# Enantioselective Total Synthesis of (+)-Heilonine

**DOI:** 10.1021/jacs.1c08756

**Published:** 2021-09-29

**Authors:** Kyle J. Cassaidy, Viresh H. Rawal

**Affiliations:** Department of Chemistry, University of Chicago, Chicago, Illinois 60637, United States

## Abstract

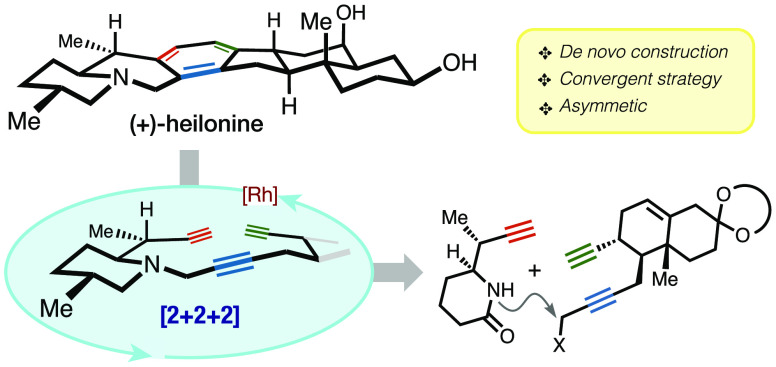

Chemical transformations that rapidly and efficiently construct a high level of molecular complexity in a single step are perhaps the most valuable in total synthesis. Among such transformations is the transition metal catalyzed [2 + 2 + 2] cycloisomerization reaction, which forges three new C–C bonds and one or more rings in a single synthetic operation. We report here a strategy that leverages this transformation to open *de novo* access to the *Veratrum* family of alkaloids. The highly convergent approach described herein includes (i) the enantioselective synthesis of a diyne fragment containing the steroidal A/B rings, (ii) the asymmetric synthesis of a propargyl-substituted piperidinone (F ring) unit, (iii) the high-yielding union of the above fragments, and (iv) the intramolecular [2 + 2 + 2] cycloisomerization reaction of the resulting carbon framework to construct in a single step the remaining three rings (C/D/E) of the hexacyclic cevanine skeleton. Efficient late-stage maneuvers culminated in the first total synthesis of heilonine (**1**), achieved in 21 steps starting from ethyl vinyl ketone.

Steroidal alkaloids have been shown to possess a wide range of biological activities that are relevant to human health.^1^ One such class of compounds is derived from the *Veratrum* genus of liliaceous plants. These highly intricate alkaloids all share a common C-*nor*-D-*homo* steroid skeleton and can be categorized into three different structural types based on the connectivity to the piperidine (F) ring (Figure 1A).^2^ The cevanine-type consists of an entirely fused hexacyclic scaffold, wherein rings E and F comprise a basic nitrogen-containing quinolizidine unit. Members of this group are adorned with varying levels of oxygenation, as exemplified by heilonine (**1**) and germine (**2**). Despite being the largest subclass of the *Veratrum* family—more than 70 members in total have been isolated to date—only one of them has yielded to chemical synthesis (*vide infra*). As opposed to their cevanine counterparts, the veratramine and jervine types are more investigated in terms of their synthesis and therapeutic potential.^3−5^ These members can be characterized by either the absence of an E ring or its inclusion as a spirofuran motif (cf. namesake compounds veratramine (**5**) and jervine (**6**), respectively).

**Figure 1:**
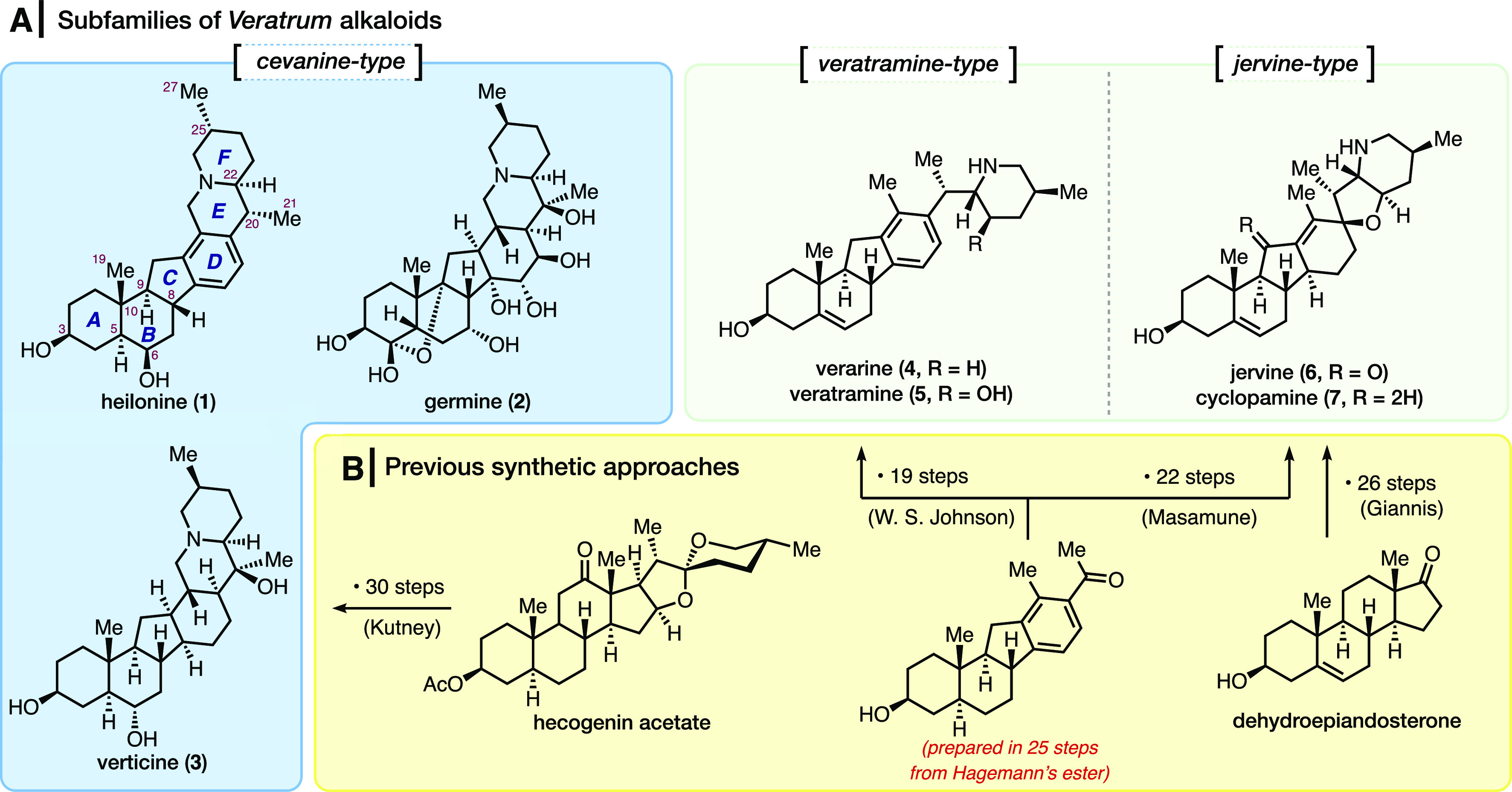
(A) Representative members of the *Veratrum* alkaloids of the three structural subtypes and (B) Previous total syntheses of the *Veratrum* alkaloids.

The early synthetic interest in these alkaloids culminated in total syntheses of veratramine (**5**) and jervine (**6**) by the groups of Johnson and Masamune, respectively (Figure 1B).^3a,3b^ Shortly after these breakthrough achievements, Kutney published a total synthesis of verarine (**4**) along with an improved route to the above-mentioned congeners.^3c−3e^ All of these strategies involved the coupling of a C-*nor*-D-*homo* steroid system with a heterocyclic piperidine unit or synthon thereof. A decade later, in 1977, Kutney reported the semisynthesis of verticine (**3**), the first and only chemical synthesis reported to date of a cevanine-type alkaloid. This approach also capitalized on the convergent nature of the aforementioned strategies; however, 30 steps were needed (starting from hecogenin acetate) to reach the final target.^3f,3g^ The only other member to succumb to synthesis in the past 40 years was reported in 2009, when Giannis published a semisynthetic strategy to cyclopamine (**7**), through a route requiring 26 steps from dehydroepiandosterone.^3h,6^ Notably, despite decades of effort from numerous laboratories,^4^ the *de novo* chemical synthesis of these alkaloids remains an unmet challenge.^7^ We have developed a general strategy to construct the C-*nor*-D-*homo* steroid skeleton of these alkaloids via a transition metal catalyzed [2 + 2 + 2] cycloisomerization reaction and have incorporated this chemistry to complete a convergent synthesis of heilonine (**1**), a member of the cevanine subfamily possessing the conspicuous aromatic D ring, which is also found in a handful of related alkaloids (see Figure 1A).

The isolation of heilonine (**1**) was reported in 1989 from *Fritillaria ussuriensis* Maxim., collected in the Hei-Long-Jiang province in China, from which its name was derived.^8^ The complex hexacyclic structure of the natural product along with its nine stereogenic centers was elucidated by NMR spectroscopy and X-ray crystallographic analysis. Heilonine is believed to be a constituent in the important Chinese herbal drug “Bei-mu”, which has traditionally been used as a sedative, antitussive, and expectorant.^9^

In our retrosynthetic plan toward **1**, we envisioned hexacycle **A** as a key intermediate that could arise from an intramolecular alkyne trimerization of **B** (Scheme 1). Since the initial discovery by Reppe in 1948 that transition metals can catalyze the cyclotrimerization of alkynes and pioneering applications by Vollhardt in several classic steroid syntheses,^10,11^ the [2 + 2 + 2] cycloaddition has seen relatively few applications in complex molecule synthesis despite its ability to forge multiple C–C bonds and rings in a single step.^12,13^ This powerful simplifying transform leads to a plausible point of divergence, as triyne **B** could be traced back to two fragments of similar complexity: bicyclic diyne **C** and propargyl-substituted piperidinone **D**. The stereochemistry of **D** would be set during an asymmetric Evans aldol reaction between a propionyl unit **H** and aldehyde **I**. Stereoinvertive azidation of the resultant syn-aldol adduct followed by Staudinger-type reductive cyclization would form the piperidinone ring. The preparation of fragment **C** was anticipated to be achieved by a Robinson annulation to form the A ring. Functional group interconversions and nucleophilic displacement with an acetylide to introduce the propargyl unit led us to intermediate **E**, which was expected to arise from an enantioselective Diels–Alder reaction between **F** and **G**. With convergent access to the hexacyclic scaffold of **A**, we anticipated that a diastereoselective methylation and several late-stage redox adjustments would allow us to complete the total synthesis of heilonine.

**Scheme 1:**
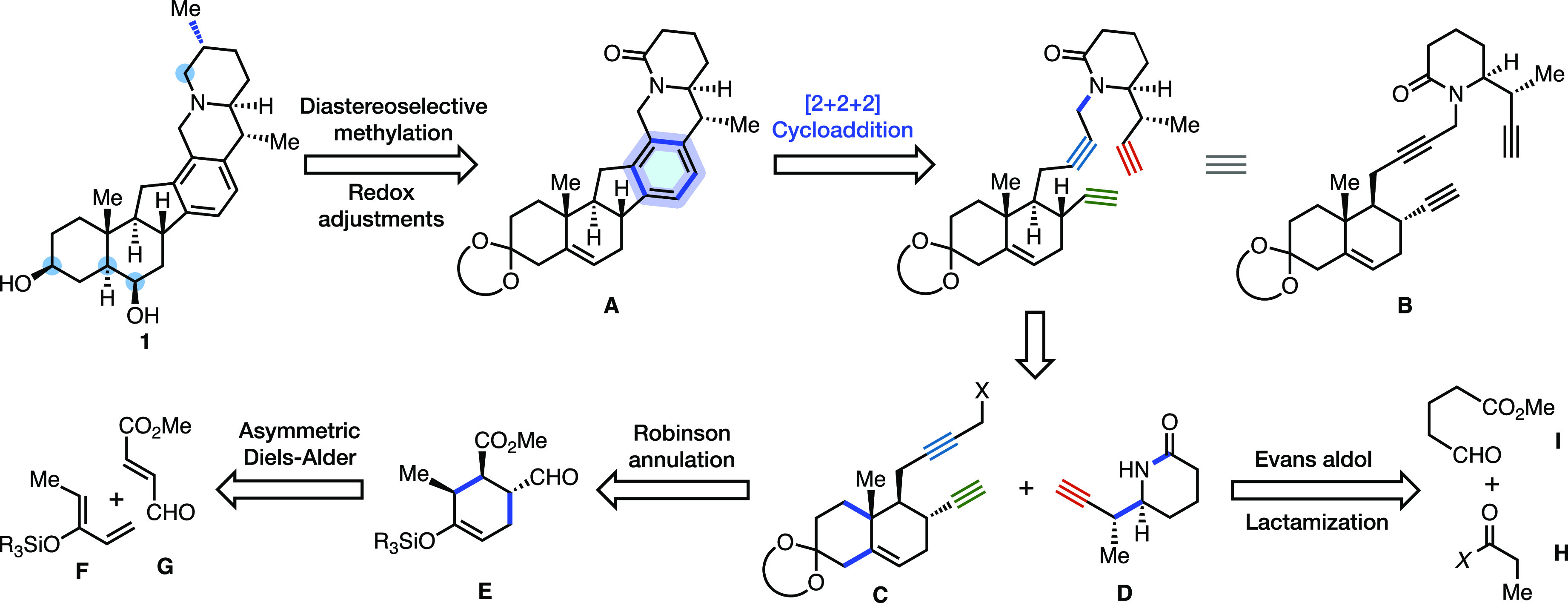
Retrosynthetic Strategy to Heilonine (**1**) Utilizing a Convergent Fragment Coupling and [2 + 2 + 2] Cycloaddition

Preparation of propargylic bromide **17** began with siloxydiene **8** and commercially available dienophile **9** through the use of an organocatalytic, enantioselective Diels–Alder reaction employing diarylprolinol-derived catalyst **18** (Scheme 2).^14^ Developed by Yang and co-workers for the preparation of a similar starting material en route to their landmark total synthesis of (+)-propindilactone G, this transformation is reported to be highly efficacious, atom-economic, and scalable, so it presented a convenient starting point for building the A/B ring fragment **17**.^15^ Indeed, the Diels–Alder reaction proceeded well and afforded exo cycloadduct **10** in 67% yield and 90% *ee* on a decagram scale.^16^ The structure assigned to **10** was consistent with ^1^H NMR coupling constants and NOE analysis of a downstream intermediate (see Supporting Information).

**Scheme 2:**
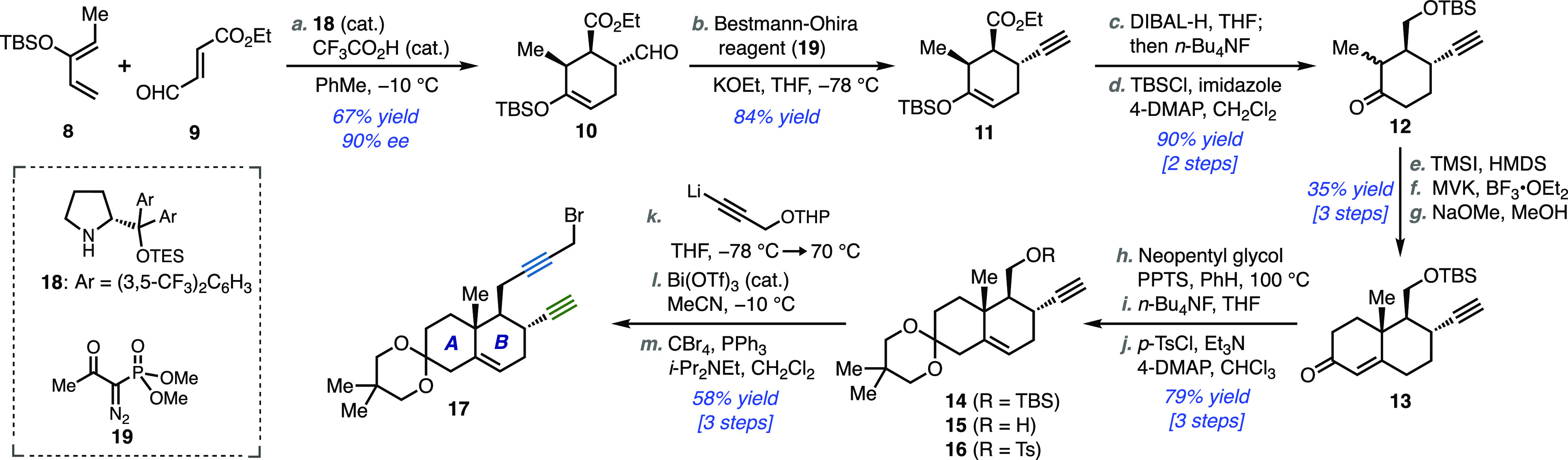
Synthesis of Propargyl Bromide Fragment (**17**) Reagents and conditions: (a) **8** (1.2 equiv), **9** (1 equiv), **18** (10 mol %), CF_3_CO_2_H (20 mol %), −10 °C, PhMe, 16 h (67%, 90% *ee*); (b) KOEt (3.2 equiv), **19** (3.6 equiv), −78 °C, THF, 1.5 h (84%); (c) DIBAL-H (3.3 equiv), −78 °C to rt, THF, 3 h *then add* MeOH (1.2 equiv), *n*-Bu_4_NF (3 equiv), 0 °C to rt, 3 h (97%, dr = 2.3:1); (d) TBSCl (1.1 equiv), imidazole (1.5 equiv), 4-DMAP (0.1 equiv), 0 °C to rt, CH_2_Cl_2_, 24 h (93%); (e) TMSI (1.2 equiv), HMDS (1.6 equiv), 0 °C to rt, CH_2_Cl_2_, 4 h (97%); (f) Methyl vinyl ketone (2 equiv), MeNO_2_ (3 equiv), *i*-PrOH (3 equiv), BF_3_·OEt_2_ (2.3 equiv), −78 °C to −65 °C, CH_2_Cl_2_, 24 h (55%, dr = 3:1); (g) NaOMe (1.6 equiv), rt to 40 °C, MeOH, 8 h (66%); (h) 2,2-Dimethylpropane-1,3-diol (5 equiv), PPTS (0.2 equiv), 100 °C, PhH, 20 h (63% **14** + 24% **15**); (i) *n*-Bu_4_NF (2 equiv), rt, THF, 20 h (93%); (j) *p*-TsCl (3 equiv), Et_3_N (10 equiv), 4-DMAP (2 equiv), 0 °C to rt, CHCl_3_, 24 h (96%); (k) Tetrahydro-2-(2-propynyloxy)-2*H*-pyran (2.5 equiv), *n*-BuLi (2.4 equiv), −78 °C to rt, THF, 2 h *then add***16** (1 equiv), – 78 to 70 °C, 18 h (75%); (l) Bi(OTf)_3_ (5 mol %), 2,2-Dimethylpropane-1,3-diol (5 equiv), −10 °C, MeCN, 2 h (87%); (m) CBr_4_ (1.2 equiv), PPh_3_ (1.5 equiv), *i*-Pr_2_NEt (3 equiv), 0 °C to rt, CH_2_Cl_2_, 2 h (89%).

Aldehyde **10** underwent Gilbert–Seyferth homologation to provide alkyne **11** in 84% yield through the use of the Bestmann–Ohira reagent.^17^ As extensive epimerization was observed under the standard protocol (i.e., K_2_CO_3_ in methanol), the reaction conditions were carefully examined to overcome this complication. Ultimately, it was found that preforming the diazo anion species in situ prior to addition of the substrate furnished the homologated product cleanly and in good yield.^18^ A sequence involving one-pot ester reduction—silyl enol ether hydrolysis (DIBAL-H; then *n*-Bu_4_NF) and silylation of the primary alcohol (TBSCl, imidazole, 4-DMAP)—afforded **12** as an inconsequential mixture of diastereomers in 90% yield over two steps.

Next, a three-step Robinson annulation protocol was employed to furnish octalone **13**.^19^ Thus, **12** was smoothly converted to its thermodynamic silyl enol ether (TMSI, HMDS), which underwent a Mukaiyama–Michael reaction with methyl vinyl ketone (BF_3_·OEt_2_, *i*-PrOH) and subsequent aldol closure/dehydration (NaOMe, MeOH) to provide **13**. An inseparable 3:1 mixture of diastereomers was formed during the Mukaiyama–Michael reaction; however, only the major (desired) stereoisomer underwent subsequent aldol cyclization/dehydration, permitting clean isolation of **13**. Although the isolated yield for this annulation sequence was moderate, due to competing silyl enol ether hydrolysis back to **12** during the Mukaiyama–Michael step, this material could be smoothly recycled to afford a 61% yield of **13** (over three steps) after three sequences of recycling hydrolyzed material.

Octalone **13** was transformed to tosylate **16** in 79% yield over three steps: ketalization with concomitant olefin isomerization, silyl deprotection, and sulfonylation. Treatment of **16** with the lithio derivative of propargyl tetrahydropyranyl (THP) ether in THF and heating the resultant solution at reflux for 18 h afforded the desired diyne (not shown) in 75% yield. Attempts to perform this reaction at lower temperature with or without the use of polar aprotic additives (e.g., DMPU, HMPA) gave inferior results. Chemoselective deprotection of the THP acetal (catalytic Bi(OTf)_3_, 2,2-dimethylpropane-1,3-diol, MeCN) and bromination (CBr_4_, PPh_3_, *i*-Pr_2_NEt) gave the requisite propargyl bromide fragment **17** in 77% yield over two steps.

The synthesis of the piperidinone fragment commenced with an Evans aldol reaction between commercially available **20** and known aldehyde **21** (Scheme 3).^20,21^ The desired syn-aldol adduct was formed in excellent yield as a single diastereomer. A Mitsonobu reaction (PPh_3_, DIAD, DPPA) was used to install an azide, and the Evans auxiliary was subsequently removed (LiOBn) to afford diester **23** in 66% yield over two steps. Staudinger reduction of the azide resulted in spontaneous lactamization under the reaction conditions to cleanly form **24** in 89% yield. Of note, removal of the chiral auxiliary *prior* to the reductive cyclization was found to be necessary due to competing cyclization occurring exclusively on the imide moiety. Once the piperidinone ring had been formed, the ester was converted to aldehyde **25** in two steps, followed by modified Gilbert–Seyferth homologation to furnish the requisite fragment **26**.

**Scheme 3:**
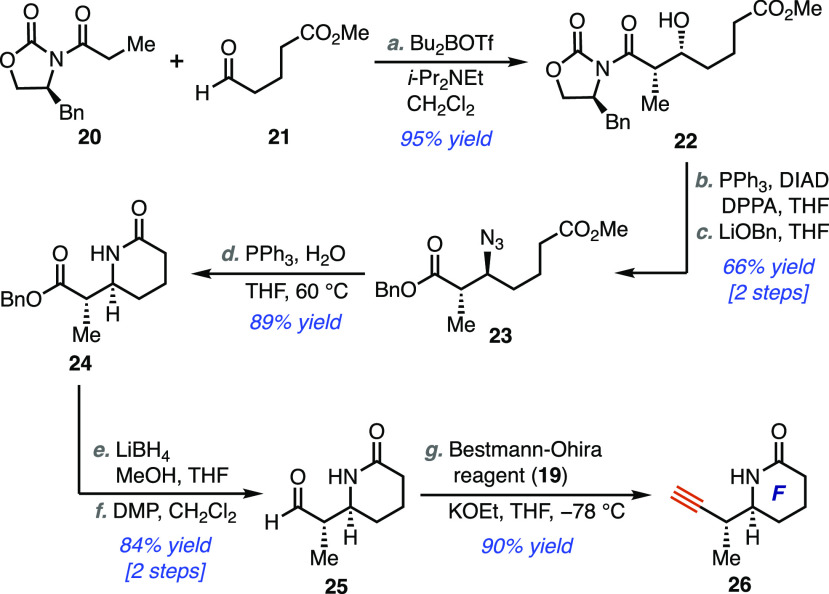
Synthesis of Piperidinone Fragment (**26**) Reagents and conditions: (a) **20** (1 equiv), *n*-Bu_2_BOTf (1.1 equiv), *i*-Pr_2_NEt (1.4 equiv), −78 to 0 °C, CH_2_Cl_2_, 3.5 h *then add***21** (1.1 equiv), −78 °C to rt, 20 h *then add* MeOH/pH 7 buffer, 30% aq. H_2_O_2_, 0 °C, 2 h (95%); (b) PPh_3_ (1.5 equiv), DIAD (1.5 equiv), DPPA (1.5 equiv), 0 °C to rt, 10 h (85%); (c) Benzyl alcohol (2 equiv), *n*-BuLi (1.8 equiv), −78 to 0 °C, THF, 30 min, *then* −40 °C to −20 °C, 3.5 h (78%); (d) PPh_3_ (1.2 equiv), H_2_O (10 equiv), 60 °C, THF, 24 h (89%); (e) LiBH_4_ (5 equiv), MeOH (6 equiv), 0 °C to rt, THF 24 h (94%); (f) Dess-Martin periodinane (1.3 equiv), rt, CH_2_Cl_2_, 3 h *then add* solid NaHCO_3_ (98%); (g) KOEt (3.3 equiv), **19** (4 equiv), −78 °C to −50 °C, THF, 1.5 h (90%).

The two chiral fragments were conjoined by alkylation of piperidinone **26** (NaH, DMF) with propargyl bromide **17** to provide triyne **27** in excellent yield (Scheme 4). Significantly, only 1 equiv of each fragment was necessary in this highly efficient reaction. The critical [2 + 2 + 2] cycloisomerization required some optimization to provide synthetically useful yields (see Supporting Information for a brief summary). Ultimately, we found that RhCl(PPh_3_)_3_ (Wilkinson’s catalyst) in refluxing ethanol smoothly effected the alkyne trimerization, affording the desired cevanine framework in 89% yield.^22^ In accordance with a previous report,^22b^ it was found that a polar solvent was crucial to achieve a high yield of the cycloisomerized product. This transformation can also be performed using conditions developed by Yamamoto (Cp*Ru(cod)Cl, DCE),^23^ but it required higher catalyst loadings in order to obtain useful yields. With a robust set of conditions leading to hexacyclic intermediate **28**, all that remained was installation of the C-27 methyl group and some redox adjustments. We found the lactam methylation to be quite challenging, and many standard conditions failed to provide any conversion to the methylated product.^24^ It was not until strongly basic conditions (*n*-BuLi, *t*-BuLi) were employed that any methylation was observed, delivering **29** in moderate yield and as a ca. 2:1 mixture of C-25 epimers. Upon further investigation, we found lithium 2,2,6,6-tetramethylpiperidine (LiTMP) in the presence of hexamethylphosphoramide (HMPA) as a cosolvent led to an extremely rapid trapping of the intermediate enolate by methyl iodide. After a thorough optimization of the reaction conditions, it was found that 3 equiv of LiTMP were necessary to achieve complete enolization (as indicated by full conversion of **28** upon ensuing treatment with MeI). Furthermore, quenching of the reaction mixture with methanol at −78 °C gave the desired diastereomer in high selectivity (dr = 7:1) and permitted clean isolation of **29** in 63% yield, while a side product containing an additional methyl group was also recovered (10–15% yields). Precise control of reagent stoichiometry was deemed crucial, as a significant amount of this dimethylated side product (40–45% yields) was formed when too large of an excess of base was used. An analysis of the 2-D NMR of the side-product indicates that the additional methyl group is incorporated at the benzylic carbon (C-18), giving an inseparable 4:1 mixture of diastereomers of undetermined relative stereochemistry. The formation of this byproduct necessitates the intermediacy of the C-18-lithio species, presumably through benzylic lithiation of the initially formed enolate.^25^

**Scheme 4:**
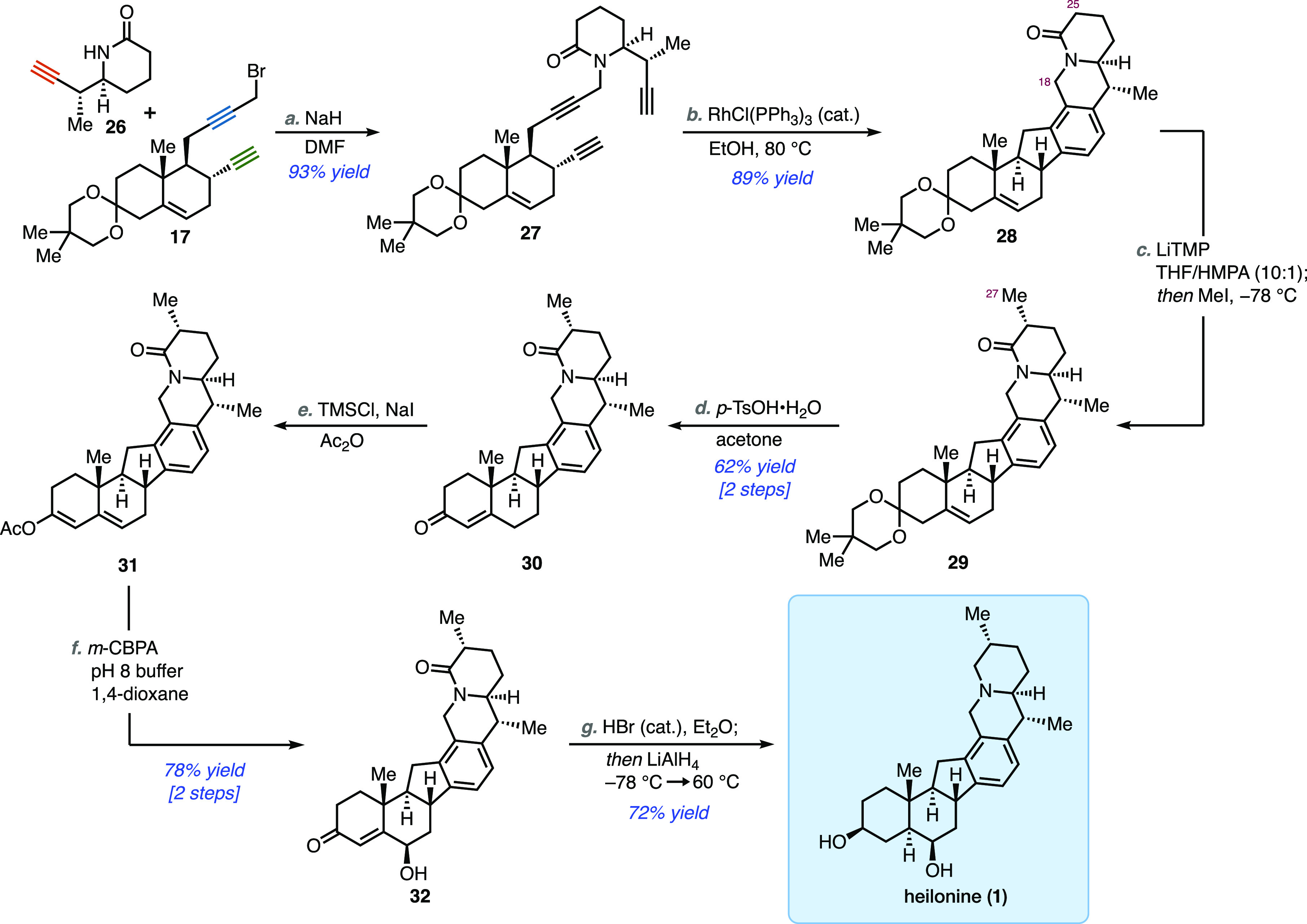
Completion of the Total Synthesis of Heilonine (**1**) Reagents and conditions: (a) NaH (1.5 equiv), DMF, 0 °C to rt, 18 h (93%); (b) RhCl(PPh_3_)_3_ (10 mol %), EtOH, 80 °C, 30 min (89%); (c) Lithium 2,2,6,6-tetramethylpiperidine (3 equiv), HMPA/THF (1:10), −78 °C, 20 min *then add* MeI (8 equiv), −78 °C, 1 min *then add* MeOH (quench), −78 °C (72%, dr = 7:1); (d) *p*-TsOH·H_2_O (2.5 equiv), H_2_O, acetone, 0 °C to rt, 15 h (98%); (e) TMSCl (4 equiv), NaI (4 equiv), Ac_2_O, 0 °C, 4 h (100%); (f) *m*-CPBA (1.2 equiv), KH_2_PO_4_–Na_2_HPO_4_ buffer (pH 8), 1,4-dioxane, 0 °C to rt, 20 h (78%); (g) HBr (0.1 equiv), Et_2_O, rt, 1.5 h *then add* LiAlH_4_ (3 equiv), −78 °C to rt, 1 h *then add* LiAlH_4_ (4 equiv), rt to 60 °C, 18 h (72%).

Intermediate **29**, which now possesses the entire carbon skeleton of the natural product, was treated with *p*-toluenesulfonic acid in acetone to afford enone **30** in near-quantitative yield. This material was transformed (Ac_2_O, TMSCl, NaI) to dienol acetate **31**,^26^ the oxidation of which with *m*-chloroperoxybenzoic acid furnished γ-hydroxyenone **32** in 78% yield over two steps.^27^ Treatment of **32** with catalytic hydrobromic acid promoted its clean isomerization to a γ-diketone (not shown),^28^ an intermediate that was directly subjected to global reduction, which accomplished the diastereoselective reduction of both ketones and the total reduction of the lactam functionality to afford heilonine (**1**). For the purpose of full characterization, this material was converted to its diacetate, which was found to be spectroscopically identical to the diacetate derivative of naturally isolated heilonine.^8^

In summary, we have achieved the first total synthesis of heilonine (**1**), representing the first *de novo* synthesis of a cevanine-type alkaloid. The key to the success of our strategy was the utilization of an intramolecular [2 + 2 + 2] cycloisomerization to forge the central aromatic D ring, along with C and E rings concomitantly. Other indispensable features to our strategy include an organocatalytic enantioselective Diels–Alder reaction, a challenging late-stage diastereoselective methylation of a heptacyclic intermediate, and a one-pot acid-catalyzed isomerization–global reduction. The modularity and convergent nature of the approach described herein will grant expedient access to unnatural analogs and facilitate evaluation of the biological activity of related members of the ceveratrum family. Efforts are currently underway in our laboratory to modify this strategy to allow access to the other subclasses of *Veratrum* alkaloids.

## Supporting Information Available

Experimental procedures and spectroscopic data for all new compounds (PDF)
