# Supplementary material for: Enantioselective Total Synthesis of (+)-Heilonine
Source: J Am Chem Soc. 2021 Sep 29;143(40):16394–400. doi: 10.1021/jacs.1c08756 (PMC8517970; doi:10.1021/jacs.1c08756)
Supplement: Supplementary file 1 — ja1c08756_si_001.pdf [file ja1c08756_si_001.pdf]

SUPPORTING INFORMATION

Enantioselective Total Synthesis of (+)-Heilonine

Kyle J. Cassaidy† and Viresh H. Rawal*,†

†Department of Chemistry
University of Chicago
5735 S Ellis Ave, Chicago, IL 60637, United States

Email: vrawal@uchicago.edu

<u>Table of Contents</u> **<u>Page</u>**

Section	Page
General Experimental	S2
Abbreviations	S3
Literature Preparations, Commercial Vendors, and Reagent Purification	S4
Experimental Procedures	S9
Miscellaneous Information	S45
Experimental Spectra	S48
Schemes of Previous Syntheses	S175

General Experimental:

All reactions were performed in oven-dried (>12 h at 150 °C) and/or flame-dried glassware equipped with a Teflon-coated magnetic stir bar under an atmosphere of nitrogen which had been pre-dried by passage through a Drierite column ($CaSO_4$ ≥ 98% + $CoCl_2$ <2%) unless otherwise specified. Reaction solvents dichloromethane (CH_2Cl_2; unstabilized HPLC grade), tetrahydrofuran (THF; HPLC grade), toluene (PhMe; ACS grade), and diethyl ether (Et_2O; ACS grade, stabilized with BHT) were dried by passage through an activated alumina column purification system (Innovative Technology Inc. Pure-Solv^{TM}). Anhydrous methanol (MeOH), ethanol (EtOH), 2-propanol (*i*-PrOH), and benzene (PhH) were purchased from Sigma-Aldrich and used as received. Anhydrous acetonitrile (MeCN) and *N*,*N*-dimethylformamide (DMF) was purchased from ACROS Organics and used as received. Commercially obtained reagents were used as received, unless stated otherwise. Ambient temperature refers to 20 – 24 °C. Higher than ambient temperature was maintained using pre-heated oil baths. Lower temperatures were maintained using a cooling bath of acetone/dry ice (-78 °C), water/ice (0 °C), or NESLAB CB-80 Cryobath for all other temperatures in between. All reaction temperatures refer to external bath temperatures monitored using a thermometer, unless otherwise stated.

Thin-layer chromatography (TLC) was performed using EMD Millipore silica gel 60 Å plates and visualization was achieved with either UV fluorescence quenching (254 nm), potassium permanganate stain ($KMnO_4$) with heat, or Seebach's stain ($Ce(SO_4)_2$ in phosphomolybdic acid) with heat. Flash column chromatography was performed on SiliCycle SiliaFlash P60 (40-63 µm particle size) using ACS grade solvents purchased from Fisher Scientific. Nuclear magnetic resonance (NMR) data were acquired on either a 400 MHz Bruker Avance-III-HD nanobay spectrometer equipped with a BBFO SmartProbe and 60-sample SampleCase autosampler or a 500 MHz Bruker Avance-III-HD spectrometer equipped with a BBFO SmartProbe, using Topspin 3.6.2. ^{1}H NMR spectra were calibrated from internal standard (TMS: δ 0.0 ppm) or solvent (CD_3OD: δ 3.31 ppm) resonance and ^{13}C NMR spectra from solvent ($CDCl_3$: δ 77.16 ppm, CD_3OD: δ 49.00 ppm) resonance. Chemical shifts (δ) are reported in parts per million (ppm) relative to the residual solvent resonance and coupling constants (*J*) are reported in hertz (Hz). NMR peak pattern abbreviations are as follows: s = singlet, d = doublet, t = triplet, q = quartet, pent = pentet, sept = septet, dd = doublet of doublets, dt = doublet of triplets, td = triplet of doublets, tt = triplet of triplets, qd = quarter of doublets, ddd = doublet of doublet of doublets, ddt = doublet of doublet of triplets, tdd = triplet of doublet of doublets, m = multiplet, br = broad (i.e., signal is broadened), app = apparent (i.e., signal appears as). All ^{1}H and ^{13}C are corroborated by 2-D experiments (e.g., COSY, HSQC). High-resolution mass spectral (HRMS) analysis was measured on Agilent Technologies 6224 TOF LC/MS using electrospray ionization (ESI) at the University of Chicago Mass Spectroscopy Core Facility. Optical rotations were measured on a Jasco DIP-1000 polarimeter using a 100 mm-path-length cell, *c* = g/100 mL. Infrared (IR) spectra were recorded on a Thermo Scientific Nicolet iS50 FT-IR spectrometer and are reported as a frequency of absorption (cm^{-1}). Chiral high-performance liquid chromatography (HPLC) analysis was performed using an Agilent analytical

chromatography system with a commercial Chiralcel® column (OD-H) equipped with a guard.

Abbreviations:

KHMDS = potassium hexamethyldisilazide; DIBAL-H = diisobutylaluminum hydride; TMS = trimethylsilyl; TES = triethylsilyl; TBS = *tert*-butyldimethylsilyl; PCC = pyridinium chlorochromate; *m*-CPBA = *meta*-chloroperoxybenzoic acid; HMDS = hexamethyldisilazane; 4-DMAP = 4-dimethylaminopyridine; MVK = methyl vinyl ketone; THP = tetrahydropyranyl; PPTS = pyridinium *p*-toluenesulfonate; Ts = *p*-toluenesulfonyl; DIAD = diisopropyl azodicarboxylate; DPPA = diphenylphosphoryl azide; Bn = benzyl; DMP = Dess-Martin periodinane; TMP = 2,2,6,6-tetramethylpiperidine; HMPA = hexamethylphosphoramide.

Literature Preparations, Commercial Vendors, and Reagent Purification:

Scheme S1. Detailed synthetic route to Hayashi ligand (**18**).[1]

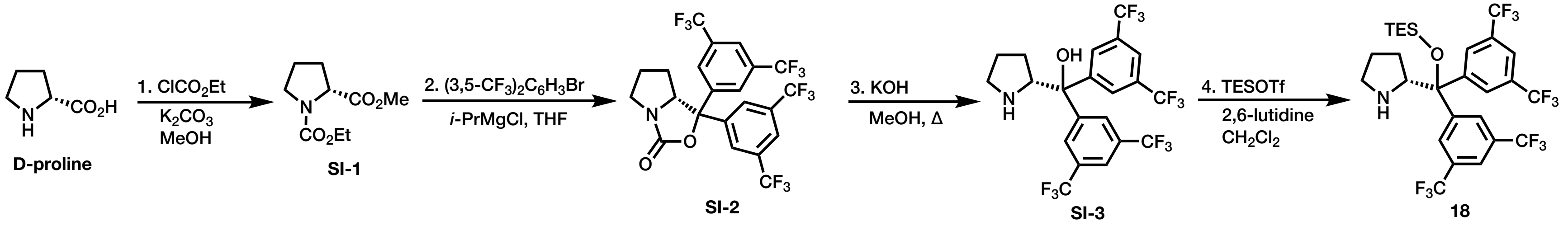

Scheme S2. Detailed synthetic route to **21**.[2]

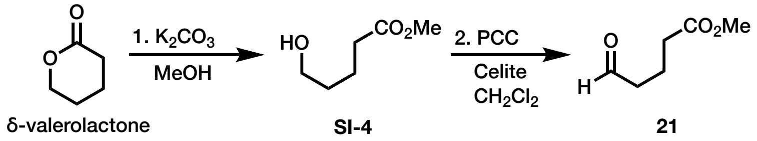

Commercial Sources of Starting Materials and Selected Reactants/Reagents

- δ-Valerolactone: Sigma-Aldrich, tech. grade
- Ethyl vinyl ketone: Sigma-Aldrich, ≥97%, stabilized with BHT
- Ethyl trans-4-oxo-2-butenoate: Alfa Aesar, 96%
- D-proline: Oakwood Chemical, 99%
- 3-Buten-2-one: Sigma Aldrich, tech. grade, 90%
- Tetrahydro-2-(2-propynyloxy)-2*H*-pyran: Sigma-Aldrich, 98%
- 2,2-Dimethyl-1,3-propanediol: Sigma-Aldrich, 99%
- Chlorotris(triphenylphosphine)rhodium(I): Strem Chemicals, 99%
- Potassium ethoxide: Sigma-Aldrich, 95%
- Boron trifluoride diethyl etherate: Sigma-Aldrich, purified by redistillation, ≥46.5% BF_3 basis
- Trimethylsilyl iodide: Oakwood Chemical, stabilized over Cu, 95%
- Dess-Martin periodinane: Oakwood Chemical, 95%

[1] Lin, Q.; Meloni, D.; Pan, Y.; Xia, M.; Rodgers, J.; Shepard, S.; Li, M.; Galya, L.; Metcalf, B.; Yue, T.; Liu, P.; Zhou, J. Enantioselective Synthesis of Janus Kinase Inhibitor INCB018424 via an Organocatalytic Aza-Michael Reaction. *Org. Lett.* **2009**, *11*, 1999−2002.
[2] Huang, J.; Bolm, C. Microwave-Assisted Synthesis of Heterocycles by Rhodium(III)-Catalyzed Annulation of *N*-Methoxyamides with α-Chloroaldehydes. *Angew. Chem. Int. Ed.* **2017**, *56*, 15921−15925.

Purification of Reagents

- Ethyl vinyl ketone: short-path distillation (bp 112 °C at 760 mm Hg)
- Ethyl trans-4-oxo-2-butenoate: flash column chromatography (25:1 → 10:1 hexanes:EtOAc)
- 3-Buten-2-one: stirred over K_2CO_3 and $CaCl_2$ at 0 °C for 1 h, then short-path distillation (bp 80 – 81 °C at 760 mm Hg)
- Tetrahydro-2-(2-propynyloxy)-2*H*-pyran: dried over Na_2CO_3, then short-path distillation (bp 38 – 40 °C at 1.5 mm Hg)
- *N*,*N*-Diisopropylethylamine: short-path distillation over CaH_2 (bp 80 – 83 °C at 250 mm Hg)
- Hexamethyldisilazane: short-path distillation over CaH_2 (bp 79 – 81 °C at 250 mm Hg)
- Hexamethylphosphoramide: short-path distillation over CaH_2 (bp 75 – 77 °C at 1.5 mm Hg)
- 2,2,6,6-Tetramethylpiperidine: short-path distillation over CaH_2 (bp 110 – 112 °C at 250 mm Hg)
- *p*-Toluenesulfonyl chloride: dissolved in $CHCl_3$ (10 g / ca. 25 mL), filtered, diluted filtrate with petroleum ether (5 volumes), clarified filtrate with charcoal, then concentrated in vacuo
- Bestmann-Ohira reagent: flash column chromatography (100% EtOAc)
- Nitromethane: short-path distillation over CaH_2 (bp 102 – 103 °C at 760 mm Hg)
- Di-*n*-butyl boron trifluoromethanesulfonate: short-path distillation (bp 56 – 58 °C at 1.5 mm Hg)
- Chlorotrimethylsilane: short-path distillation over CaH_2 (bp 56 – 58 °C at 760 mm Hg)
- Benzyl alcohol: short-path distillation (discarding forerun; bp 62 – 63 °C at 1.5 mm Hg)

Scheme S3. Detailed synthetic route to **17**.

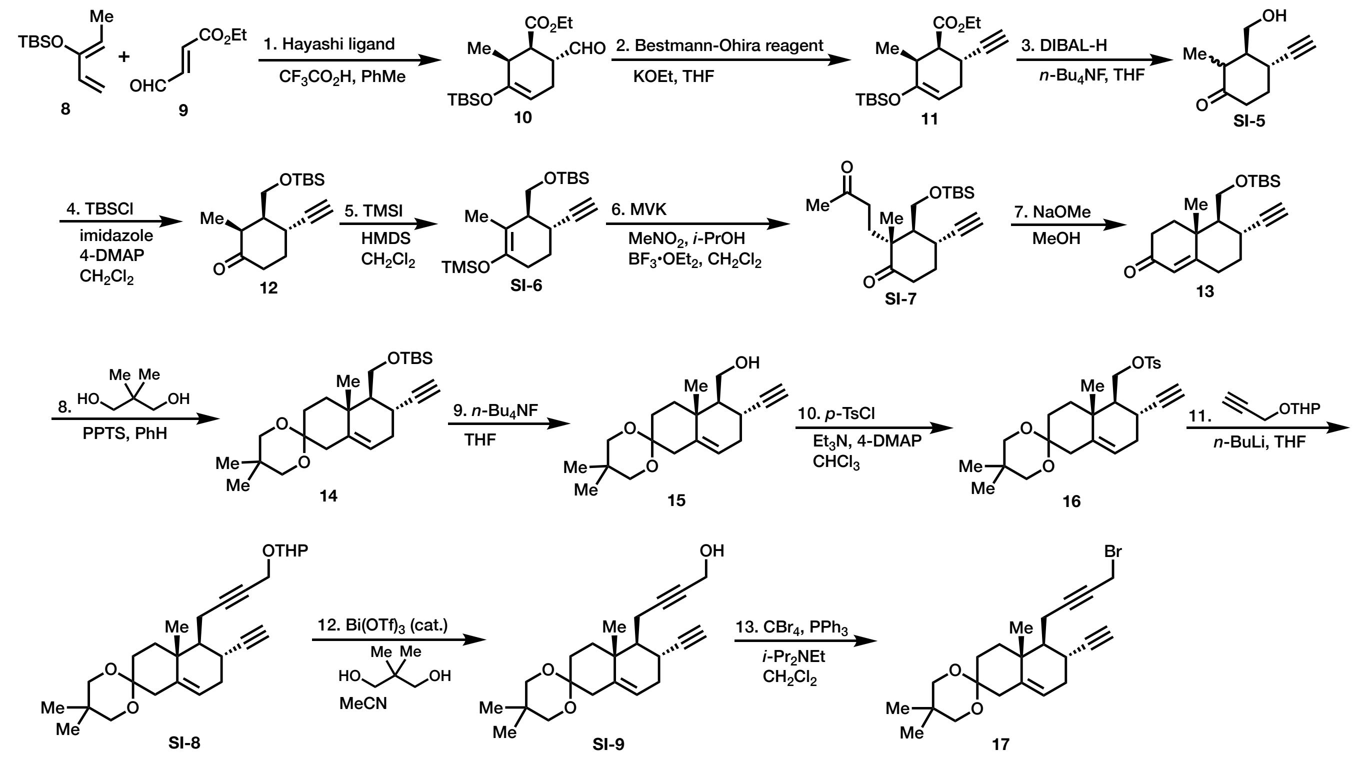

Scheme S4. Detailed synthetic route to **26**.

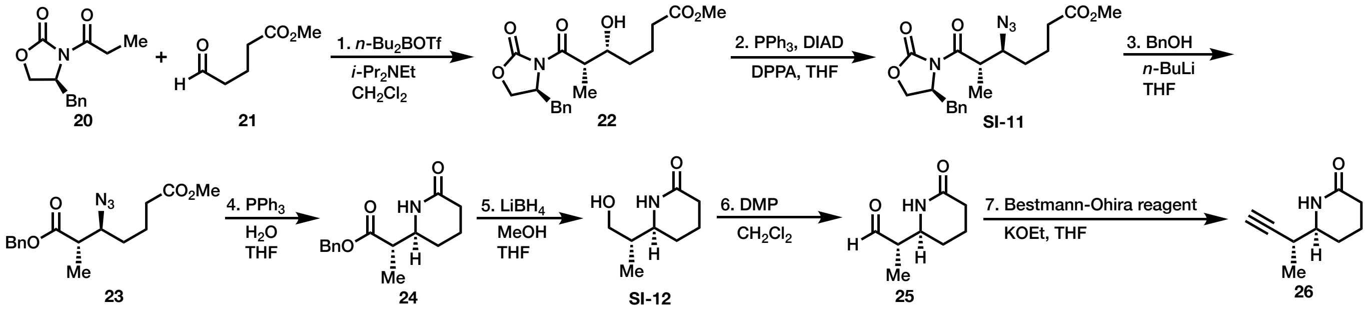

Scheme S5. Detailed synthetic route to **heilonine** and **heilonine diacetate**.

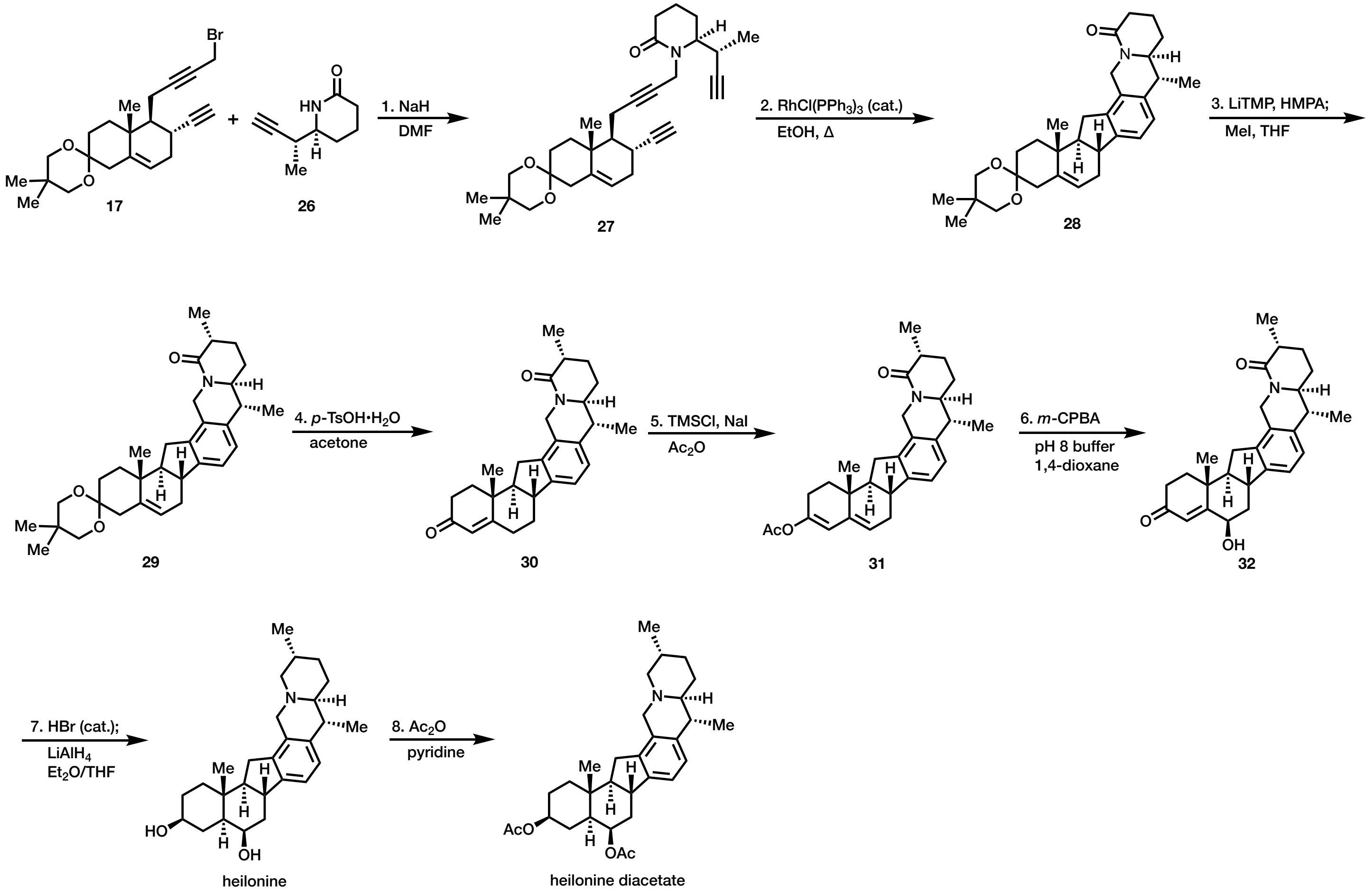

Experimental Procedures:

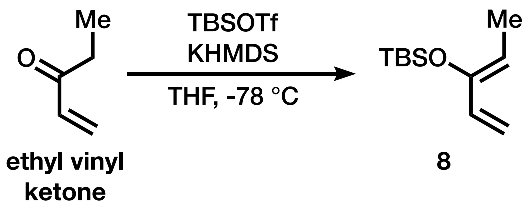

Siloxydiene 8.[3] To a pre-cooled solution of ethyl vinyl ketone (11.0 g, 131 mmol, 1.0 equiv) in THF (750 mL) was added *tert*-butyldimethylsilyl trifluoromethanesulfonate (30 mL, 131 mmol, 1.0 equiv) slowly at −78 °C. The resultant solution was treated with a 1.0 M solution of KHMDS in THF (160 mL, 160 mmol, ca. 1.2 equiv), which was added via cannula dropwise over 30 minutes at −78 °C. Following complete addition, the pale-yellow mixture was stirred at −78 °C for 45 min, then the cold bath was removed and reaction mixture was stirred for an additional 1 h upon warming to ambient temperature. The mixture was cooled to 0 °C and quenched with a saturated aqueous $NaHCO_3$ solution (300 mL), then concentrated in vacuo until the total volume was approximately 500 mL. The residual mixture was extracted with 10:1 hexanes:Et_2O (3 x 300 mL), then the combined organic extracts were washed with brine (1 x 400 mL) and dried over Na_2SO_4. Concentration of the dried extracts provided a crude yellow oil, which was purified via flash column chromatography (100% petroleum ether) to afford siloxydiene **8** (18.6 g, 72% yield; >50:1 *Z*:*E*) as a clear, colorless oil.

^{1}H NMR (500 MHz, $CDCl_3$): δ 6.16 (dd, *J* = 17.1, 10.8 Hz, 1H), 5.27 (d, *J* = 17.1 Hz, 1H), 4.94 (d, *J* = 10.8 Hz, 1H), 4.87 (q, *J* = 7.0 Hz, 1H), 1.64 (d, *J* = 7.0 Hz, 3H), 1.01 (s, 9H), 0.12 (s, 6H).

^{13}C NMR (101 MHz, $CDCl_3$): δ 149.6, 135.7, 111.8, 110.2, 26.1, 18.6, 11.9, -3.5.

IR (thin film) ν_{max} (cm^{-1}): 3102 (w), 3024 (w), 2957 (s), 2930 (s), 2859 (s), 1648 (m), 1607 (m), 1474 (m), 1465 (w), 1385 (m), 1364 (w), 1340 (s), 1290 (m), 1255 (s), 1203 (m), 1082 (m), 1050 (s), 1015 (w), 1006 (w), 983 (w), 939 (m), 897 (m), 839 (s), 803 (m), 779 (s), 740 (w), 695 (m).

***R_f* =** 0.46 (100% hexanes; visualized with UV and $KMnO_4$ stain).

---

[3] Known compound; this procedure was adapted from the following: Liu, P.; Sirois, L. E.; Cheong, P. H.; Yu, Z.; Hartung, I. V.; Rieck, H.; Wender, P. A.; Houk, K. N. Electronic and Steric Control of Regioselectivities in Rh(I)-Catalyzed (5+2) Cycloadditions: Experiment and Theory. *J. Am. Chem. Soc.* **2010**, *132*, 10127−10135.

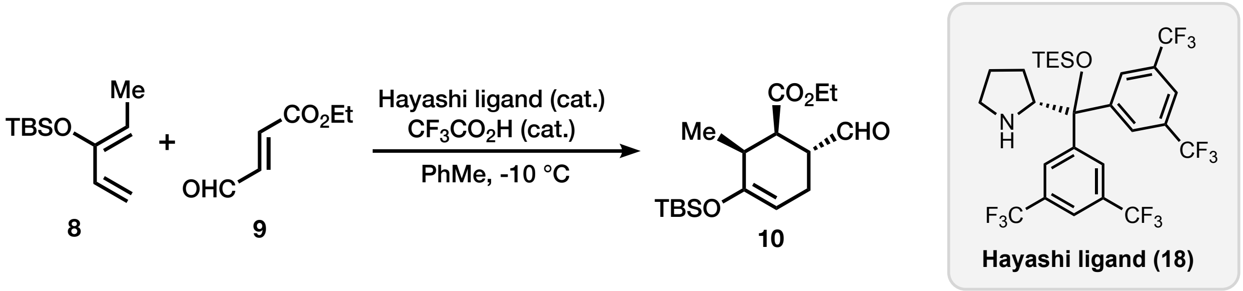

TBS enol ether 10. A solution of ethyl trans-4-oxo-2-butenoate[4] (8.6 mL, 71.3 mmol, 1.0 equiv) in PhMe (4.0 mL) was treated dropwise with solution of Hayashi ligand (4.56 g, 7.13 mmol, 10 mol%) in PhMe (11 mL) at ambient temperature. To the resultant pale-yellow solution was added a solution of trifluoroacetic acid (1.10 mL, 14.3 mmol, 20 mol%) in PhMe (30 mL) and the mixture was cooled to −10 °C. The clear, yellow solution was next treated with siloxydiene **8** (16.9 g, 85.2 mmol, 1.2 equiv) dropwise over 15 minutes at −10 °C. The resultant solution was stirred at −10 °C for 16 h, then diluted with hexanes (80 mL) and neutralized with a saturated aqueous $NaHCO_3$ solution (40 mL) at −10 °C. The mixture was brought to ambient temperature and extracted with 5:1 hexanes:Et_2O (2 x 50 mL), then the combined organic extracts were washed with brine (1 x 100 mL) and dried over Na_2SO_4. Concentration of the dried extracts[5] afforded a crude yellow residue that was purified via flash column chromatography (100:0 hexanes:EtOAc → 13:1 hexanes:EtOAc) to afford TBS enol ether **10** (15.6 g, 67% yield) as a pale-yellow oil.

^{1}H NMR (500 MHz, $CDCl_3$): δ 9.81 (d, J = 1.8 Hz, 1H), 4.75 (dd, J = 5.3, 2.4 Hz, 1H), 4.18 (q, J = 7.1 Hz, 2H), 3.01 (dd, J = 11.8, 5.6 Hz, 1H), 2.92 (tdd, J = 11.6, 5.7, 1.8 Hz, 1H), 2.54 (app pent, J = 6.6 Hz, 1H), 2.30 (dt, J = 16.7, 5.5 Hz, 1H), 1.98 (app ddt, J = 16.7, 11.4, 2.0 Hz, 1H), 1.27 (t, J = 7.1 Hz, 3H), 1.00 (d, J = 7.0 Hz, 3H), 0.93 (s, 9H), 0.15 (6H).

^{13}C NMR (101 MHz, $CDCl_3$): δ 203.1, 172.9, 153.6, 99.3, 60.9, 45.3, 42.6, 35.2, 25.8, 24.0, 18.1, 15.0, 14.4, -4.2, -4.5.

COSY/HSQC: see **Figure S5** / **Figure S6**.

HRMS: (ESI) m/z $[M+H]^+$ calcd for $C_{17}H_{30}O_4Si$: 327.1991, found: 327.1994.

$[\alpha]_D^{21.9}$ = −105.4 (c = 1.0, $CHCl_3$).

[4] This material (commercially available, Alfa Aesar) had a tendency to decompose upon storing for several months in a refrigerator. The purity was always checked via ^{1}H NMR prior to use and, if necessary, purified via flash column chromatography, see: Harmand, T. J.; Murar, C. E.; Takano, H.; Bode, J. W. Enantioselective Synthesis of *(S)*-Ethyl 2-((*tert*-butoxycarbonyl)((*tert*-butyldimethylsilyl)oxy)amino)-4-oxobutanoate. *Org. Synth.* **2018**, *95*, 142−156.

[5] Bath temperature was maintained below 30 °C, as product decomposition was observed at higher temperatures.

IR (thin film) ν_{max} (cm^{-1}): 2956 (m), 2929 (m), 2856 (m), 1727 (s), 1669 (s), 1472 (m), 1463 (m), 1377 (m), 1302 (m), 1278 (m), 1251 (m), 1196 (s), 1097 (w), 1027 (m), 838 (s), 778 (s), 665 (w).

***R_f* =** 0.32 (10:1 hexanes:EtOAc; visualized with $KMnO_4$ stain).

HPLC: (Chiralcel® OD-H column, see method details below, 210 nm)

Time (min)	% Hexanes	% i-PrOH	Flow Rate (mL/min)
0	99.8	0.2	0.500
3.0	99.7	0.3	0.500
5.0	99.5	0.5	0.500
8.0	99.2	0.8	0.500
15	99.0	1.0	0.500

t_{major} = 21.5 min, t_{minor} = 21.9 min (90% *ee*). The chromatographic traces are shown in a separate SI section.

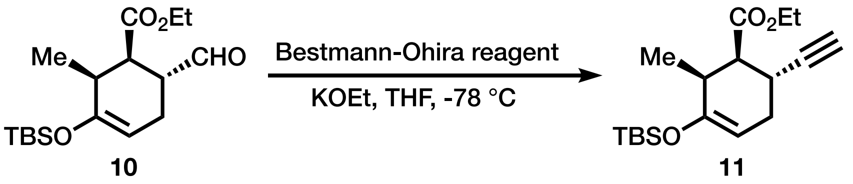

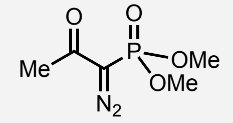

Bestmann-Ohira reagent (19)

Alkyne 11. To a flame-dried 1-L, three-necked round-bottomed flask charged with a 4 cm Teflon-coated magnetic oval stir bar and equipped with an inert gas inlet (Ar), thermometer, and rubber septum was added potassium ethoxide (6.95 g, 82.6 mmol, 3.2 equiv) and THF (500 mL). The resultant mixture was cooled to −78 °C (internal temperature per thermometer) and treated with solution of Bestmann-Ohira reagent[6] (17.8 g, 92.9 mmol, 3.6 equiv) in THF (120 mL) via cannula transfer at a rate such that the internal reaction temperature did not exceed −74 °C. After complete addition, the empty flask and cannula were rinsed with THF (3 x 3 mL) and the resultant opaque, bright yellow reaction mixture was stirred at −78 °C for 25 min. A solution of TBS enol ether **10** (8.42 g, 25.8 mmol, 1 equiv) in THF (70 mL) was then added to the reaction mixture via cannula transfer at a rate such that the internal reaction temperature did not exceed −72 °C. After complete addition, the empty flask and cannula were rinsed with THF (3 x 3 mL) and the resultant effervescing yellowish-orange reaction mixture was

[6] Bestmann-Ohira reagent was prepared according to the following reference: Pietruszka, J.; Witt, A. *Synthesis* **2006**, 4266−4268.

stirred at −78 °C for 1 h. The cold bath was next removed and, upon warming to −5 °C (internal temperature), the reaction mixture was quenched with a saturated aqueous NH_4Cl solution (300 mL) and diluted with hexanes (400 mL). The biphasic mixture was separated and the aqueous extracts were washed with 5:1 hexanes:Et_2O (2 x 450 mL). All organic extracts were combined, washed with brine (1 x 500 mL), and dried over Na_2SO_4. Concentration of the dried extracts provided a crude yellow residue, which was purified via flash column chromatography (20:1 hexanes:Et_2O) to afford alkyne **11** (6.98 g, 84% yield) as a clear, light-yellow oil.

^{1}H NMR (400 MHz, $CDCl_3$): δ 4.69 (dd, *J* = 5.1, 2.1 Hz, 1H), 4.21 (q, *J* = 7.2 Hz, 2H), 2.83 (dd, *J* = 11.7, 5.3 Hz, 1H), 2.77 (m, 1H), 2.48 – 2.37 (m, 2H), 2.18 (ddt, *J* = 17.0, 10.6 Hz, 1H), 2.03 (d, 2.1 Hz, 1H), 1.29 (t, *J* = 7.2 Hz, 3H), 1.00 (d, *J* = 7.0 Hz), 0.92 (s, 9H), 0.14 (6H).

^{13}C NMR (101 MHz, $CDCl_3$): δ 172.4, 152.8, 100.1, 86.8, 69.0, 60.5, 49.9, 35.5, 30.3, 25.8, 23.3, 18.1, 14.8, 14.4, -4.3, -4.5.

COSY/HSQC: see **Figure S10** / **Figure S11**.

HRMS: (ESI) *m/z* $[M+H]^+$ calcd for $C_{18}H_{30}O_3Si$: 323.2042, found: 323.2046.

$[\alpha]_D^{23.2}$ = −102.7 (*c* = 1.0, $CHCl_3$).

IR (thin film) ν_{max} (cm^{-1}): 3312 (s), 2956 (m), 2929 (m), 2856 (m), 2111 (w), 1739 (s), 1670 (s), 1472 (m), 1463 (m), 1374 (m), 1253 (s), 1196 (s), 1152 (s), 1032 (m), 1005 (m), 910 (s), 838 (s), 799 (m), 778 (s), 671 (m), 634 (s).

***R_f* =** 0.46 (10:1 hexanes:EtOAc; visualized with $KMnO_4$ stain).

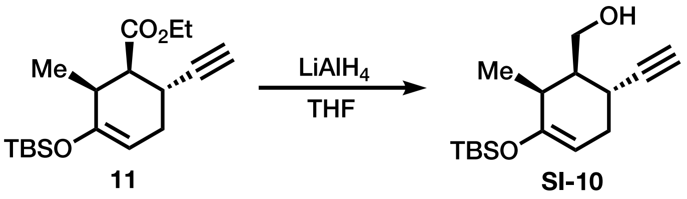

Primary Alcohol SI-10. To a pre-cooled solution of alkyne **11** (6.81 g, 21.1 mmol, 1.0 equiv) in THF (200 mL) was added lithium aluminum hydride solution (2.0 M in THF; 13.5 mL, 27.0 mmol, 1.3 equiv) dropwise at 0 °C. The resultant mixture was allowed to warm to ambient temperature and stirred for 2 h. Following complete consumption of starting material as indicated by TLC, the reaction mixture was re-cooled to 0 °C and diluted with Et_2O (200 mL). The reaction was carefully quenched by sequential addition

of H_2O (1.0 mL), 2 M NaOH aqueous solution (1.0 mL), and H_2O (3.0 mL) and vigorously stirred for 15 min upon warming to ambient temperature. Anhydrous $MgSO_4$ was then added and the mixture was stirred for an additional 15 minutes. The mixture was next passed through a layer of Celite, washing the filter cake thoroughly with Et_2O (ca. 400 mL). Concentration of the filtrate provided primary alcohol **SI-10** (5.62 g, 95% yield) as a clear, colorless oil which solidified to white crystals upon refrigeration. The crude concentrate was typically clean enough to be carried forward without purification, however flash column chromatography (8:1 hexanes:EtOAc) was used to obtain spectroscopically pure material.

^{1}H NMR (400 MHz, $CDCl_3$): δ 4.69 (dd, *J* = 5.1, 2.4 Hz, 1H), 3.96 (dd, *J* =11.3, 6.3 Hz , 1H), 3.65 (dd, *J* = 11.3, 6.9 Hz, 1H), 2.51 – 2.42 (tdd, *J* = 10.8, 5.3, 2.3 Hz, 1H), 2.40 – 2.28 (m, 2H), 2.24 – 2.17 (m, 1H), 2.16 (d, *J* = 2.4 Hz, 1H), 2.05 – 1.94 (m, 2H), 0.99 (d, *J* = 7.0 Hz, 3H), 0.92 (s, 9H), 0.13 (6H).

^{13}C NMR (101 MHz, $CDCl_3$): δ 154.4, 100.5, 87.1, 70.6, 64.1, 44.3, 35.7, 31.3, 25.8, 24.4, 18.1, 13.1, -4.3, -4.5.

COSY/HSQC: see **Figure S14** / **Figure S15**.

HRMS: (ESI) *m*/*z* $[M+H]^+$ calcd for $C_{16}H_{28}O_2Si$: 281.1937, found: 281.1942.

$[\alpha]_D^{22.6}$ = −98.7 (*c* = 1.0, $CHCl_3$).

IR (thin film) ν_{max} (cm^{-1}): 3310 (br), 2956 (s), 2929 (s), 2896 (m), 2857 (m), 2110 (w), 1668 (s), 1472 (m), 1463 (m), 1361 (w), 1254 (s), 1196 (s), 1168 (m), 1075 (m), 903 (s), 836 (s), 778 (s), 631 (m).

***R_f* =** 0.42 (4:1 hexanes:EtOAc; visualized with $KMnO_4$ stain).

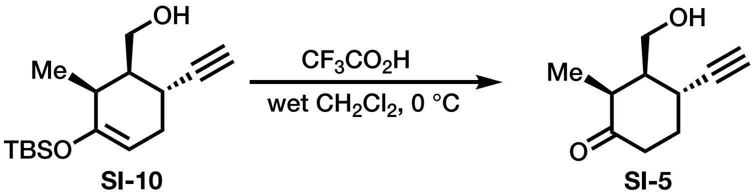

Ketone SI-5. To a pre-cooled solution of primary alcohol **SI-10** (5.59 g, 19.9 mmol, 1 equiv) in water-saturated CH_2Cl_2[7] (100 mL) was added trifluoroacetic acid (3.0 mL, 39.2

[7] Using a separatory funnel, the CH_2Cl_2 had been shaken vigorously with ca. 10 mL of distilled water for 30 seconds, then separated from the aqueous layer and used directly.

mmol, *ca.* 2 equiv) dropwise at 0 °C. The resultant mixture was stirred at 0 °C for 20 minutes and, following complete consumption of starting material by as indicated TLC, neutralized with a saturated aqueous $NaHCO_3$ solution (150 mL) at 0 °C. The biphasic mixture was stirred vigorously for 10 minutes as it was allowed to warm to ambient temperature, then the mixture was extracted with CH_2Cl_2 (6 x 80 mL) and the combined organic extracts were dried over Na_2SO_4. The dried extracts were concentrated in vacuo and the resultant crude residue was purified via flash column chromatography (6:2:1 hexanes:CH_2Cl_2:acetone) to afford ketone **SI-5** (3.07 g, 95% yield) as a clear, viscous waxy oil which solidified upon refrigeration.

^{1}H NMR (500 MHz, $CDCl_3$): δ 3.74 (app dt, *J* = 11.2, 4.4 Hz, 1H), 3.55 (m, 1H), 3.11 (m, 2H), 2.66 (m, 1H), 2.39 – 2.32 (m, 2H), 2.25 – 2.17 (m, 1H), 2.20 (d, *J* = 2.4 Hz, 1H), 2.01 (m, 1H), 1.33 (t, 4.7 Hz, 1H), 1.09 (d, *J* = 7.0 Hz, 3H).

^{13}C NMR (101 MHz, $CDCl_3$): δ 212.7, 86.3, 70.4, 61.1, 49.1, 43.1, 37.8, 28.1, 28.0, 11.7.

COSY/HSQC: see **Figure S18** / **Figure S19**.

HRMS: (ESI) *m/z* $[M+H]^+$ calcd for $C_{10}H_{14}O_2$: 167.1072, found: 167.1069.

$[\alpha]_D^{24.3}$ = +12.4 (*c* = 1.0, $CHCl_3$).

IR (thin film) ν_{max} (cm^{-1}): 3400 (br), 3285 (s), 2936 (m), 2111 (w), 1699 (s), 1452 (m), 1424 (m), 1379 (w), 1322 (m), 1286 (w), 1218 (m), 1141 (w), 1113 (m), 1049 (s), 960 (s), 640 (s).

***R_f* =** 0.47 (1:1 hexanes:EtOAc; visualized with $KMnO_4$ stain).

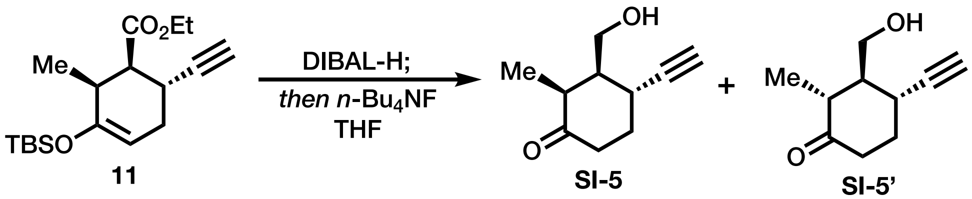

Ketone SI-5/SI-5'. To a pre-cooled solution of alkyne **11** (1.32 g, 4.09 mmol, 1.0 equiv) in THF (50 mL) was added diisobutylaluminum hydride solution (1.0 M in PhMe; 13.5 mL, 13.5 mmol, 3.3 equiv) dropwise at −78 °C. The resultant clear and colorless solution was stirred at −78 °C for 1 h, then brought to ambient temperature and stirred for an additional 2 h. The reaction mixture was cooled to 0 °C and treated slowly with anhydrous methanol (0.65 mL; to quench unreacted DIBAL-H), followed by slow addition of tetrabutylammonium fluoride solution (1.0 M in THF; 12.0 mL, 12.0 mmol, ca.

3 equiv). The resultant pale-yellow solution was next allowed to warm to ambient temperature and stirred for 3 h. The reaction mixture was then treated with saturated aqueous $NaHCO_3$ solution (25 mL) and saturated aqueous potassium sodium tartrate solution (100 mL) and extracted with 1:1 hexanes:EtOAc (4 x 80 mL). The organic extracts were combined and washed with brine (1 x 150 mL), then dried over Na_2SO_4. Concentration of the dried extracts afforded a crude colorless oil, which was purified via flash column chromatography (6:2:1 hexanes:CH_2Cl_2:acetone) to afford ketone **SI-5** and **SI-5'** (0.659 g, 97% yield) as a 1.0:2.3 mixture of inseparable C–2 epimers.

^{1}H NMR (400 MHz, $CDCl_3$): (*Major diastereomer*) δ 4.16 (dd, *J* = 11.3, 1.9 Hz, 1H), 3.81 (dd, *J* = 11.3, 2.8 Hz, 1H), 2.98 – 2.88 (tt, *J* = 11.5, 2.7 Hz, 1H), 2.64 – 2.55 (m, 1H), 2.48 – 2.32 (m, 3H), 2.19 (d, *J* = 2.4 Hz, 1H), 2.06 – 1.93 (m, 1H), 1.91 – 1.77 (m, 1H), 1.55 – 1.47 (tt, *J* = 11.4, 2.4 Hz, 1H), 1.11 (d, *J* = 6.5 Hz, 3H). (*Minor diastereomer*) see ketone **SI-5**.

^{13}C NMR (101 MHz, $CDCl_3$): (*Major diastereomer*) δ 212.1, 85.4, 70.8, 61.2, 51.2, 44.3, 40.5, 32.2, 30.1, 11.5. (*Minor diastereomer*) see ketone **SI-5**.

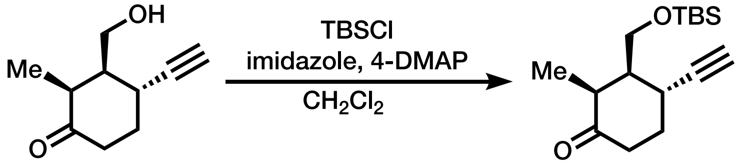

SI-5 **12**

TBS ether 12. To a pre-cooled solution of ketone **SI-5** (3.00 g, 18.5 mmol, 1.0 equiv) in CH_2Cl_2 (200 mL) was added imidazole (1.89 g, 27.8 mmol, 1.5 equiv), 4-dimethylaminopyridine (0.226 g, 1.85 mmol, 0.1 equiv), and *tert*-butyldimethylsilyl chloride (3.07 g, 20.4 mmol, 1.1 equiv) sequentially and in single, separate portions at 0 °C. The resultant opaque mixture was stirred at 0 °C for 4 h, then allowed to warm to ambient temperature and stirred for an additional 20 h. The mixture was quenched with a saturated aqueous $NaHCO_3$ solution (150 mL) and extracted with CH_2Cl_2 (3 x 150 mL). The combined organic extracts were dried over Na_2SO_4, concentrated in vacuo, and the residue purified via flash column chromatography (15:1 hexanes:Et_2O) to afford TBS ether **12** (4.80 g, 93% yield) as clear, viscous oil which froze into a white, waxy solid upon refrigeration.

^{1}H NMR (400 MHz, $CDCl_3$): δ 3.64 (dd, *J* = 10.6, 4.4 Hz, 1H), 3.47 (dd, *J* = 10.6, 8.2 Hz, 1H), 3.11 – 3.01 (m, 2H), 2.70 – 2.59 (m, 1H), 2.36 – 2.27 (m, 2H), 2.24 – 2.14 (m, 1H), 2.17 (d, *J* = 2.5 Hz, 1H), 2.01 – 1.93 (m, 1H), 1.05 (d, *J* = 6.9 Hz, 3H), 0.87 (s, 9H), 0.02 (6H).

^{13}C NMR (101 MHz, $CDCl_3$): δ 211.8, 86.8, 70.1, 61.4, 49.0, 42.8, 37.8, 28.5, 27.7, 25.9, 18.3, 11.7, -5.5, -5.5.

COSY/HSQC: see **Figure S23** / **Figure S24**.

HRMS: (ESI) *m/z* $[M+H]^+$ calcd for $C_{16}H_{28}O_2Si$: 281.1937, found: 281.1940.

$[\alpha]_D^{22.6}$ = +0.4 (*c* = 1.0, $CHCl_3$).

IR (thin film) ν_{max} (cm^{-1}): 3310 (m), 2952 (m), 2928 (s), 2884 (m), 2856 (m), 2111 (w), 1712 (s), 1471 (m), 1463 (m), 1389 (w), 1361 (w), 1252 (s), 1092 (s), 1005 (w), 988 (w), 897 (m), 834 (s), 775 (s), 634 (s).

***R_f* =** 0.35 (10:1 hexanes:Et_2O; visualized with $KMnO_4$ stain).

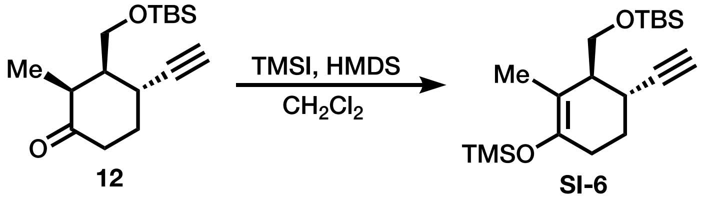

12

SI-6

TMS enol ether SI-6. To a pre-cooled solution of TBS ether **12**[8] (5.26 g, 18.8 mmol, 1.0 equiv) in CH_2Cl_2 (60 mL) was added freshly distilled hexamethyldisilazane (5.2 mL, 24.8 mmol, 1.3 equiv) at 0 °C. The resultant solution was stirred for 0.5 min, then treated with iodotrimethylsilane (3.1 mL, 22.5 mmol, 1.2 equiv) dropwise over 10 minutes at 0 °C. The thick, orange mixture was stirred for 20 minutes, then allowed to slowly warm to ambient temperature. Upon stirring at ambient temperature for 2 h, additional hexamethyldisilazane (1.4 mL, 6.2 mmol, 0.33 equiv) was added and stirring was continued for an additional 2 h. Upon consumption of starting material as indicated by TLC, the mixture was diluted with petroleum ether (250 mL) and the resultant cloudy, white mixture filtered through a layer of Florisil® (washing with ca. 200 mL of 10:1 petroleum ether:Et_2O). Concentration of the filtrate in vacuo afforded TMS enol ether **SI-6** (6.44 g, 97% yield; 97:3 thermodynamic: kinetic) as a clear, colorless oil.[9,10]

---

[8] Azeotroped with benzene (3 x 10 mL) prior to the reaction.
[9] A white solid precipitate occasionally remained following initial filtration and subsequent concentration. In this case, the residue was resuspended in pentane and re-filtered through a layer of Florisil® in order to remove this impurity. The crude concentrate was clean enough to be carried forward without purification; moreover, attempts to purify the product by column chromatography often resulted in considerable hydrolysis to return starting TBS ether **12** as a 1:1 mixture of diastereomers.
[10] Bath temperature was maintained at or below 20 °C, as product decomposition was observed at higher temperatures.

^{1}H NMR (400 MHz, CDCl$_3$): δ 3.77 (dd, J = 10.3, 3.2 Hz, 1H), 3.48 (dd, J = 10.3, 7.6 Hz, 1H), 2.85 (m, 1H), 2.25 (m, 2H), 2.02 – 1.93 (m, 1H), 1.96 (d, J = 2.2 Hz, 1H), 1.87 (m, 1H), 1.74 – 1.66 (m, 1H), 1.60 (s, 3H), 0.89 (s, 9H), 0.17 (s, 9H), 0.05 (6H).

^{13}C NMR (101 MHz, CDCl$_3$): δ 145.1, 110.4, 88.1, 67.7, 62.8, 48.1, 27.9, 26.1, 24.8, 18.4, 14.9, 0.8, -5.2, -5.3.

COSY/HSQC: see **Figure S27** / **Figure S28**.

HRMS: (ESI) m/z [M+H]$^+$ calcd for $C_{19}H_{36}O_2Si_2$: 353.2332, found: 353.2326.

$[\alpha]_D^{22.0}$ = +17.5 (c = 1.0, $CHCl_3$).

IR (thin film) ν_{max} (cm^{-1}): 3310 (s), 2980 (s), 2929 (m), 2883 (m), 2113 (w), 1682 (m), 1472 (m), 1381 (s), 1252 (s), 1161 (m), 1092 (m), 939 (m), 841 (s), 775 (w), 739 (s), 703 (m), 630 (w).

***R_f* =** 0.65 (20:1 hexanes:Et_2O; visualized with $Ce(SO_4)_2$ in phosphomolybdic acid stain).

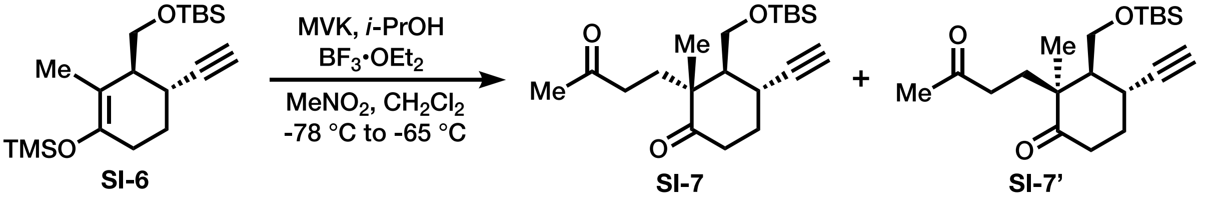

1,5-Diketone SI-7/SI-7’. A solution of TMS enol ether **SI-6** (6.42 g, 18.2 mmol, 1.0 equiv) in dry CH_2Cl_2 (120 mL) was cooled to −78 °C. Nitromethane (3.0 mL, 54.6 mmol, 3.0 equiv), methyl vinyl ketone (3.1 mL, 36.4 mmol, 2.0 equiv), and 2-propanol (4.2 mL, 54.6 mmol, 3.0 equiv) were added sequentially and all dropwise at −78 °C. The resultant mixture was stirred for 5 minutes before $BF_3{\cdot}OEt_2$ (3.4 mL, 27.3 mmol, 1.5 equiv) was added dropwise at −78 °C via syringe pump over a period of 2 h. The reaction mixture was then stirred at −78 °C for 4 h. At this point, additional $BF_3{\cdot}OEt_2$ (1.8 mL, 14.6 mmol, 0.8 equiv) was added dropwise over 20 minutes. The reaction mixture was then brought to −65 °C and stirred for an additional 20 hours at this same temperature. Upon consumption of starting material as indicated by TLC, the reaction mixture was allowed to warm slowly to 0 °C and quenched with saturated aqueous $NaHCO_3$ solution (100 mL). The mixture was extracted with CH_2Cl_2 (3 x 100 mL) and the combined organic extracts were washed with brine (1 x 250 mL) and dried over Na_2SO_4. Concentration of the dried extracts provided a crude oil, which was purified via

flash column chromatography (25:1 hexanes:EtOAc → 6:1 hexanes:EtOAc)[11] to afford 1,5-diketone **SI-7** and **SI-7'** (3:1 dr; 3.51 g, 55% yield [80% brsm]) as a clear, colorless oil.

^{1}H NMR (400 MHz, CDCl$_3$): (*Major diastereomer* — **SI-7**) δ 3.96 (dd, *J* = 10.7, 4.5 Hz, 1H), 3.84 (dd, *J* = 10.7, 2.1 Hz, 1H), 3.06 – 2.98 (m, 1H), 2.58 – 2.41 (m, 2H), 2.39 – 2.27 (m, 3H), 2.15 (s, 3H), 2.13 (d, *J* = 2.3 Hz, 1H), 2.12 – 2.02 (m, 1H), 1.87 – 1.74 (m, 3H), 1.16 (s, 3H), 0.89 (s, 9H), 0.07 (6H). (*Minor diastereomer* — **SI-7'**) δ 4.07 (dd, *J* = 10.8, 4.3 Hz, 1H), 3.91 (dd, *J* = 10.8, 1.3 Hz, 1H), 3.19 – 3.09 (m, 1H), 2.58 – 2.41 (m, 1H), 2.39 – 2.27 (m, 3H), 2.12 – 2.02 (m, 1H), 2.10 (d, *J* = 2.4 Hz, 1H), 2.09 (s, 3H), 2.02 – 1.92 (m, 2H), 1.76 – 1.71 (m, 1H), 1.55 – 1.49 (m, 1H), 1.13 (s, 3H), 0.92 (s, 9H), 0.09 (6H).

^{13}C NMR (101 MHz, CDCl$_3$): (*Major diastereomer* — **SI-7**) δ 213.4, 208.7, 86.5, 70.3, 61.6, 50.8, 48.5, 39.1, 37.1, 31.1, 30.1, 29.9, 26.7, 26.0, 22.0, 18.3, -5.6. (*Minor diastereomer* — **SI-7'**) δ 213.5, 207.7, 86.1, 70.2, 61.5, 53.9, 51.5, 38.5, 37.7, 32.2, 30.1, 28.1, 26.4, 26.0, 20.3, 18.3, -5.5.

COSY/HSQC: see **Figure S31** / **Figure S32**.

HRMS: (ESI) *m/z* $[M+H]^+$ calcd for $C_{20}H_{34}O_3Si$: 351.2355, found: 351.2359.

IR (thin film) ν_{max} (cm^{-1}): 2953 (m), 2928 (s), 2883 (m), 2855 (m), 2112 (w), 1707 (s), 1471 (m), 1427 (w), 1360 (m), 1252 (s), 1165 (m), 1110 (s), 1069 (w), 1005 (m), 956 (m), 879 (w), 836 (s), 807 (w), 777 (s), 667 (m), 634 (m).

***R_f* =** 0.31 (5:1 hexanes:EtOAc; visualized with $Ce(SO_4)_2$ in phosphomolybdic acid stain).

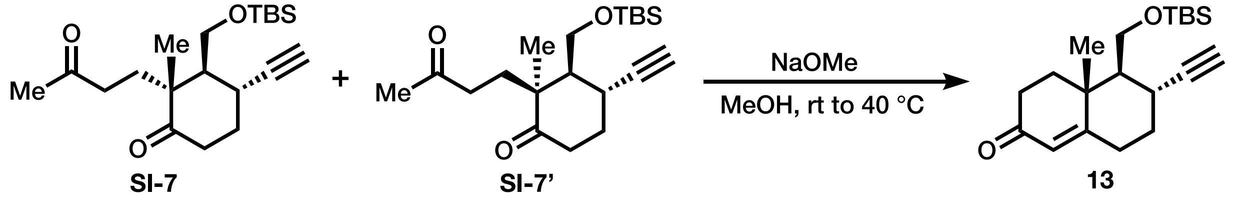

Octalone 13. To a solution of 1,5-diketone **SI-7/SI-7'**, a 3:1 mixture of diastereomers obtained from the previous step, (3.12 g, 8.90 mmol, 1.0 equiv) in MeOH (100 mL) was added sodium methoxide (0.627 g, 11.6 mmol, 1.3 equiv) in a single portion. The resultant mixture was stirred at ambient temperature for 5 h, then an additional portion of sodium methoxide (0.144 g, 2.67 mmol, 0.3 equiv) was added. The yellow solution

[11] This particular gradient was used to recover the less-polar TBS ether **12** (1.58 g, 31% yield)

was then heated to 40 °C and stirred at the same temperature for 3 h. Upon consumption of starting material as indicated by TLC, the reaction mixture was brought back to ambient temperature, diluted with Et_2O (80 mL), and quenched with saturated aqueous $NaHCO_3$ solution (50 mL). The mixture was extracted first with 1:1 hexanes:Et_2O (2 x 100 mL), then with EtOAc (1 x 100 mL). The combined organic extracts were washed with brine (1 x 200 mL) and dried over Na_2SO_4. Concentration of the dried extracts provided a crude yellow oil, which was purified via flash column chromatography (9:1 hexanes:EtOAc) to afford octalone **13** (1.94 g, 66% yield) as a white, waxy solid.

^{1}H NMR (400 MHz, $CDCl_3$): δ 5.72 (s, 1H), 4.06 – 4.00 (dd, *J* = 10.9, 4.6 Hz, 1H), 4.00 – 3.94 (dd, *J* = 10.9, 1.4 Hz, 1H), 2.88 – 2.79 (m, 1H), 2.52 – 2.32 (m, 3H), 2.30 – 2.15 (m, 3H), 2.09 (d, *J* = 2.3 Hz, 1H), 1.88 – 1.77 (td, *J* = 13.7, 4.8 Hz, 1H), 1.66 – 1.53 (m, 1H), 1.40 – 1.33 (ddd, *J* = 11.6, 4.6, 1.4 Hz, 1H), 1.28 (s, 3H), 0.91 (s, 9H), 0.08 (6H).

^{13}C NMR (101 MHz, $CDCl_3$): δ 199.3, 169.4, 124.1, 86.6, 69.9, 60.5, 53.3, 39.1, 36.7, 33.9, 33.1, 32.3, 27.4, 26.0, 19.7, 18.3, -5.5, -5.6.

COSY/HSQC: see **Figure S35** / **Figure S36**.

HRMS: (ESI) *m*/*z* $[M+H]^+$ calcd for $C_{20}H_{32}O_2Si$: 333.2250, found: 333.2246.

$[\alpha]_D^{23.2}$ = +77.4 (*c* = 1.0, $CHCl_3$).

IR (thin film) ν_{max} (cm^{-1}): 2927 (s), 2883 (m), 2855 (m), 2113 (w), 1672 (s), 1619 (m), 1471 (m), 1348 (m), 1252 (s), 1234 (m), 1193 (w), 1115 (s), 1094 (s), 1063 (m), 1005 (s), 964 (m), 952 (w), 882 (w), 835 (s), 809 (w), 775 (s), 666 (m), 630 (s), 560 (m).

***R_f* =** 0.35 (5:1 hexanes:EtOAc; visualized with UV, $KMnO_4$ stain, and/or $Ce(SO_4)_2$ in phosphomolybdic acid stain).

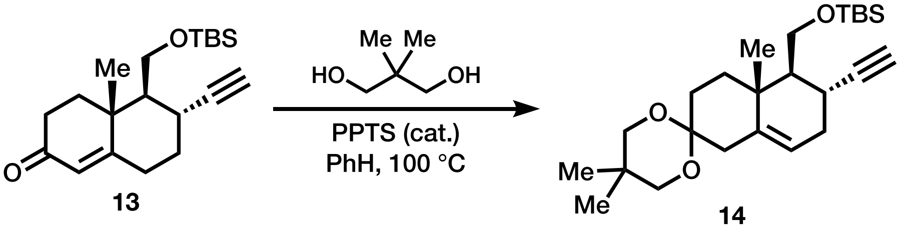

Neopentyl glycol ketal 14. To a solution of octalone **13** (1.93 g, 5.80 mmol, 1.0 equiv) in anhydrous benzene (120 mL) was added 2,2-dimethylpropane-1,3-diol (3.02 g, 29.0 mmol, 5 equiv) and pyridinium *p*-toluenesulfonate (0.292 g, 1.16 mmol, 0.2 equiv) at ambient temperature. A Dean-Stark apparatus was assembled, then the entire reaction

setup was put under an atmosphere of argon and heated to reflux. The initially clear and colorless reaction mixture was stirred at reflux for 20 h, then the resultant pale-yellow mixture was brought back to ambient temperature, diluted with 1:1 hexanes:EtOAc (100 mL) and quenched with saturated aqueous $NaHCO_3$ solution (100 mL). The mixture was extracted with 1:1 hexanes:EtOAc (3 x 100 mL), then the organic extracts were combined and washed with brine (1 x 200 mL) and dried over Na_2SO_4. Concentration of the dried extracts provided a crude oil, which was purified via flash column chromatography (40:1 hexanes:EtOAc → 4:1 hexanes:EtOAc)[12] to afford neopentyl glycol **14** (1.53 g, 63% yield [71% brsm]) as a white solid.

^{1}H NMR (400 MHz, $CDCl_3$): δ 5.31 (m, 1H), 4.04 – 3.98 (dd, *J* = 10.6, 2.1 Hz, 1H), 3.98 – 3.91 (dd, *J* = 10.6, 5.6 Hz, 1H), 3.56 (d, *J* = 11.4 Hz, 1H), 3.50 (d, *J* = 11.4 Hz, 1H), 3.46 (s, 1H), 3.44 (s, 1H), 2.68 – 2.59 (tdd, *J* = 11.3, 5.4, 2.3 Hz, 1H), 2.59 – 2.53 (dd, *J* = 14.0, 3.1 Hz, 1H), 2.40 – 2.17 (m, 4H), 2.05 (d, *J* = 2.3 Hz, 1H), 1.99 – 1.91 (dt, *J* = 13.2, 3.5 Hz, 1H), 1.64 – 1.54 (td, *J* = 14.0, 4.0 Hz, 1H), 1.49 – 1.42 (qd, *J* = 5.8, 2.1 Hz, 1H), 1.34 – 1.23 (m, 1H), 1.10 (s, 3H), 0.99 (s, 3H), 0.92 (s, 3H), 0.89 (s, 9H), 0.07 (s, 3H), 0.05 (s, 3H).

^{13}C NMR (101 MHz, $CDCl_3$): δ 140.0, 120.8, 98.4, 87.86, 70.4, 70.0, 69.6, 61.3, 50.6, 39.6, 37.9, 35.8, 33.3, 30.3, 27.8, 26.1, 25.0, 22.9, 22.8, 19.7, 18.3, -5.4, -5.5.

COSY/HSQC: see **Figure S39** / **Figure S40**.

HRMS: (ESI) *m/z* $[M+H]^+$ calcd for $C_{25}H_{42}O_3Si$: 419.2981, found: 419.2982.

$[\alpha]_D^{21.2}$ = −41.7 (*c* = 1.0, $CHCl_3$).

IR (thin film) ν_{max} (cm^{-1}): 2952 (s), 2927 (m), 2855 (m), 2113 (w), 1471 (s), 1393 (m), 1362 (m), 1252 (s), 1211 (w), 1105 (s), 1036 (m), 1006 (m), 985 (w), 966 (w), 938 (w), 873 (w), 858 (w), 836 (s), 811 (w), 775 (m), 666 (w), 629 (m).

***R_f* =** 0.43 (10:1 hexanes:EtOAc; visualized with $Ce(SO_4)_2$ in phosphomolybdic acid stain).

[12] This particular gradient was used to also isolate the more-polar primary alcohol **15** (0.424 g, 24% yield) and a small amount of unreacted octalone **13** (0.212 g, 11% yield).

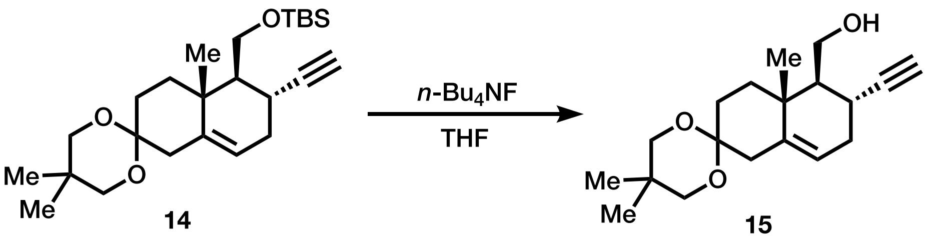

Primary alcohol 15. To a solution of neopentyl glycol **14** (1.53 g, 3.65 mmol, 1.0 equiv) in THF (25 mL) was added tetrabutylammonium fluoride solution (1.0 M in THF; 7.3 mL, 7.30 mmol, 2.0 equiv) slowly at ambient temperature. The resultant pale burgundy solution was stirred at ambient temperature for 20 h. Upon consumption of starting material as indicated by TLC, the mixture was diluted with 1:1 hexanes:EtOAc (50 mL) and quenched with saturated aqueous $NaHCO_3$ solution (50 mL). The layers were partitioned and the aqueous collected and further extracted with 1:1 hexanes:EtOAc (3 x 50 mL). The combined organic extracts were washed with brine (1 x 100 mL) and dried over Na_2SO_4. Concentration of the dried extracts provided a crude, off-white solid, which was purified via flash column chromatography (15:5:2 hexanes:CH_2Cl_2:acetone) to afford primary alcohol **15** (1.03 g, 93% yield) as a white solid.

^{1}H NMR (400 MHz, $CDCl_3$): δ 5.35 (m, 1H), 3.93 – 3.85 (m, 1H), 3.85 – 3.78 (m, 1H), 3.58 (d, J = 11.3 Hz, 1H), 3.48 (d, J = 11.3 Hz, 1H), 3.45 (s, 2H), 2.69 – 2.59 (tdd, J = 11.2, 5.4, 2.5 Hz, 1H), 2.58 – 2.49 (m, 2H), 2.38 – 2.19 (m, 4H), 2.23 (d, J = 2.4 Hz), 1.87 (dt, J = 13.0, 3.5 Hz, 1H), 1.60 – 1.50 (m, 2H), 1.43 – 1.33 (td, J = 13.6, 3.7 Hz, 1H), 1.01 (s, 3H), 0.99 (s, 3H) 0.91 (s, 3H).

^{13}C NMR (101 MHz, $CDCl_3$): δ 139.6, 120.9, 98.2, 87.9, 70.9, 70.4, 70.0, 63.0, 52.6, 39.8, 37.7, 34.5, 33.1, 30.2, 27.2, 26.5, 22.9, 22.7, 18.5.

COSY/HSQC: see **Figure S43** / **Figure S44**.

HRMS: (ESI) m/z $[M+H]^+$ calcd for $C_{19}H_{28}O_3$: 305.2116, found: 305.2119.

$[\alpha]_D^{23.0}$ = −37.3 (c = 1.0, $CHCl_3$).

IR (thin film) ν_{max} (cm^{-1}): 3461 (br), 3301 (s), 2951 (s), 2866 (m), 2110 (w), 1671 (w), 1471 (m), 1394 (m), 1363 (s), 1269 (m), 1097 (s), 1058 (w), 1030 (s), 975 (m), 948 (w), 907 (w), 801 (w), 735 (s), 702 (m), 630 (s).

***R_f* =** 0.64 (1:1 hexanes:EtOAc; visualized with $Ce(SO_4)_2$ in phosphomolybdic acid stain).

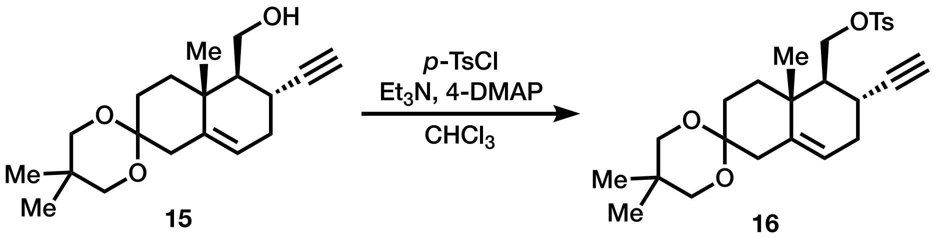

Tosylate 16. To a pre-cooled solution of primary alcohol **15** (0.729 g, 2.39 mmol, 1 equiv) in $CHCl_3$ (35 mL) was added *p*-toluenesulfonyl chloride (1.37 g, 7.17 mmol, 3 equiv), triethylamine (3.3 mL, 23.7 mmol, ca. 10 equiv), and 4-dimethylaminopyridine (0.584 g, 4.78 mmol, 2 equiv) sequentially at 0 °C. The resultant mixture was brought to ambient temperature and stirred for 24 h. Upon consumption of starting material as indicated by TLC, the reaction mixture was treated with 1:1 saturated aqueous $NaHCO_3$: H_2O solution (50 mL) and stirred vigorously for 30 minutes to hydrolyze unreacted *p*-TsCl. The mixture was then extracted with $CHCl_3$ (5 x 40 mL) and the combined organic extracts dried over Na_2SO_4. Concentration of the dried extracts provided a crude yellow oil, which was purified via flash column chromatography (20:2:1 hexanes:EtOAc:CH_2Cl_2 → 20:3:2 hexanes:EtOAc:CH_2Cl_2) to afford tosylate **16** (1.05 g, 96% yield) as a white solid.

^{1}H NMR (400 MHz, $CDCl_3$): δ 7.82 (d, *J* = 8.3 Hz, 2H), 7.34 (d, *J* = 8.3 Hz, 2H), 5.31 (m, 1H), 4.48 (dd, *J* = 10.1, 2.3 Hz, 1H), 4.28 (dd, *J* = 10.1, 5.7 Hz, 1H), 3.57 (d, *J* = 11.2 Hz, 1H), 3.47 (d, *J* = 11.2 Hz, 1H), 3.42 (s, 2H), 2.64 – 2.56 (tdd, *J* = 11.4, 5.5, 2.3 Hz, 1H), 2.56 – 2.50 (dd, *J* = 14.2, 3.1 Hz, 1H), 2.45 (s, 3H), 2.39 – 2.13 (m, 4H), 1.86 (d, *J* = 2.3 Hz, 1H), 1.83 – 1.76 (dt, *J* = 13.0, 3.5 Hz, 1H), 1.68 – 1.62 (ddd, *J* = 11.6, 5.7, 2.4 Hz, 1H), 1.59 – 1.49 (td, *J* = 14.1, 3.8 Hz, 1H), 1.34 – 1.24 (td, *J* = 13.6, 3.8 Hz, 1H), 1.05 (s, 3H), 1.00 (s, 3H), 0.90 (s, 3H).

^{13}C NMR (101 MHz, $CDCl_3$): δ 144.8, 139.2, 133.0, 130.0, 128.4, 120.9, 98.0, 85.4, 70.8, 70.4, 70.1, 69.0, 48.6, 39.9, 37.7, 35.4, 33.0, 30.2, 27.2, 25.1, 22.9, 22.7, 21.8, 19.3.

COSY/HSQC: see **Figure S47** / **Figure S48**.

HRMS: (ESI) *m*/*z* $[M+H]^+$ calcd for $C_{26}H_{34}O_5S$: 459.2205, found: 459.2205.

$[\alpha]_D^{22.1}$ = −59.6 (*c* = 1.0, $CHCl_3$).

IR (thin film) ν_{max} (cm^{-1}): 3276 (s), 2952 (s), 2867 (m), 1598 (m), 1467 (m), 1361 (s), 1269 (w), 1189 (m), 1176 (s), 1104 (s), 1022 (m), 941 (m), 857 (m), 814 (m), 711 (w), 664 (s), 555 (s).

***R_f* =** 0.45 (3:1 hexanes:EtOAc; visualized with UV and $Ce(SO_4)_2$ in phosphomolybdic acid stain).

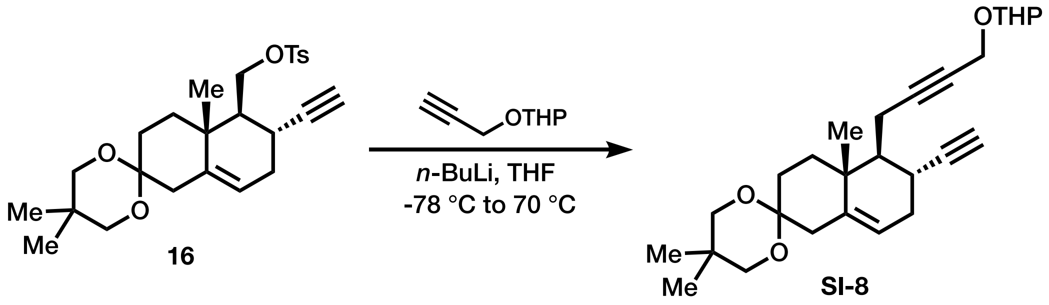

Diyne SI-8. To a solution of tetrahydro-2-(2-propynyloxy)-2*H*-pyran (0.80 mL, 5.66 mmol, 2.5 equiv) in dry THF (16 mL) was added *n*-BuLi solution (1.60 M in hexanes; 3.3 mL, 5.28 mmol, 2.4 equiv) dropwise at −78 °C. The cold bath was removed and the mixture was allowed to warm to ambient temperature, then the pale-yellow solution was stirred at room temperature for 2 h. After re-cooling to −78 °C, the reaction mixture was treated dropwise with a solution of tosylate **16**[13] (1.01 g, 2.21 mmol, 1 equiv) in dry THF (7.5 mL; rinsed 3 x 0.5 mL). Following complete addition, the cooling bath was removed and the mixture was allowed to warm to ambient temperature and stirred for an additional 30 minutes before heating to 70 °C in a pre-heated oil bath. The reaction mixture was stirred at this temperature for 18 h, then cooled to ambient temperature, quenched with a saturated aqueous $NaHCO_3$ solution (40 mL), and extracted with 1:1 hexanes:EtOAc (3 x 40 mL). The combined organic extracts were washed with brine (1 x 100 mL), dried over Na_2SO_4, then concentrated in vacuo. The resulting crude product, a light-brown oil, was purified via flash column chromatography (18:1 hexanes:EtOAc → 9:1 hexanes:EtOAc) to afford diyne **SI-8** (0.708 g, 75% yield) as a white, amorphous solid.

^{1}H NMR (400 MHz, $CDCl_3$): δ 5.33 (m, 1H), 4.85 (m, 1H), 4.24 (m, 2H), 3.89 – 3.80 (m, 1H), 3.57 (d, *J* = 11.2 Hz, 1H), 3.47 (d, *J* = 11.2 Hz, 1H), 3.56 – 3.48 (m, 1H), 3.45 (s, 1H), 3.44 (s, 1H), 2.72 – 2.52 (m, 4H), 2.40 – 2.17 (m, 4H), 2.11 (d, *J* = 2.2 Hz, 1H), 2.00 – 1.92 (dt, *J* = 13.1, 3.1 Hz, 1H), 1.88 – 1.78 (m, 1H), 1.78 – 1.68 (m, 1H), 1.68 – 1.46 (m, 6H), 1.38 – 1.27 (td, *J* = 13.7, 3.7 Hz, 1H), 1.10 (s, 3H), 1.00 (s, 3H), 0.91 (s, 3H).

^{13}C NMR (101 MHz, $CDCl_3$): δ 139.5, 120.9, 98.2, 96.5, 87.1, 86.4, 77.3, 70.4, 70.0, 62.3, 62.2, 54.7, 48.0, 39.9, 38.6, 35.3, 33.2, 30.5, 30.2, 27.6, 27.5, 25.6, 22.9, 22.7, 19.4, 18.9, 18.3.

COSY/HSQC: see **Figure S51** / **Figure S52**.

HRMS: (ESI) *m*/*z* $[M+H]^+$ calcd for $C_{27}H_{38}O_4$: 427.2848, found: 427.2847.

$[\alpha]_D^{22.1}$ = −45.0 (*c* = 1.0, $CHCl_3$).

---

[13] Azeotroped with benzene (3 x 3 mL) prior to use.

IR (thin film) ν_{max} (cm^{-1}): 3303 (s), 2948 (s), 2867 (m), 2113 (w), 1673 (w), 1453 (m), 1394 (m), 1362 (m), 1266 (m), 1200 (m), 1077 (s), 1104 (w), 1020 (s), 968 (m), 902 (m), 869 (m), 811 (m), 735 (s), 702 (w), 633 (m).

***R_f* =** 0.46 (4:1 hexanes:EtOAc; visualized with $KMnO_4$ stain).

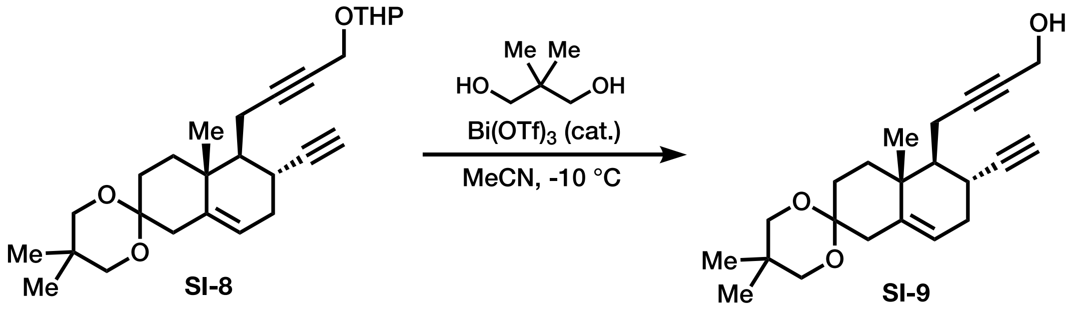

Ketalized propargyl alcohol SI-9. To a pre-cooled solution of diyne **SI-8** (0.565 g, 1.32 mmol, 1 equiv) and 2,2-dimethylpropane-1,3-diol (0.690 g, 6.62 mmol, 5 equiv) in anhydrous MeCN (16 mL) was added bismuth(III) trifluoromethanesulfonate (0.043 g, 0.066 mmol, 5 mol%) in a single portion at −10 °C. The resultant clear and colorless solution was stirred at −10 °C for 2 h, then quenched with a saturated aqueous $NaHCO_3$ solution (10 mL) and subsequently extracted with 1:1 hexanes:EtOAc (3 x 25 mL). The combined organic extracts were washed with brine (1 x 40 mL), dried over Na_2SO_4, and concentrated in vacuo. The resulting crude product, a pale-yellow oil, was purified via flash column chromatography (100% $CHCl_3$ → 20:1 $CHCl_3$:acetone)[14] to afford ketalized propargyl alcohol **SI-9** (0.394 g, 87% yield [100% brsm]) as a white solid.

^{1}H NMR (500 MHz, $CDCl_3$): δ 5.33 (m, 1H), 4.24 (s, 2H), 3.57 (d, *J* = 11.4 Hz, 1H), 3.49 (d, *J* = 11.4 Hz, 1H), 3.45 (s, 1H), 3.44 (s, 1H), 2.69 – 2.54 (m, 4H), 2.41 – 2.20 (m, 4H), 2.13 (d, *J* = 2.3 Hz, 1H), 1.97 – 1.91 (dt, *J* = 13.1, 3.5 Hz, 1H), 1.69 – 1.60 (m, 2H), 1.61 – 1.52 (td, *J* = 14.1, 3.9 Hz, 1H), 1.39 – 1.30 (td, *J* = 13.8, 3.8 Hz, 1H), 1.10 (s, 3H), 1.00 (s, 3H), 0.92 (s, 3H).

^{13}C NMR (101 MHz, $CDCl_3$): δ 139.4, 121.0, 98.2, 87.2, 86.5, 79.7, 70.5, 70.4, 70.0, 51.7, 48.0, 39.8, 38.6, 35.3, 33.2, 30.2, 27.6, 27.5, 22.9, 22.7, 18.9, 18.3.

COSY/HSQC: see **Figure S55** / **Figure S56**.

HRMS: (ESI) *m/z* $[M+H]^+$ calcd for $C_{22}H_{30}O_3$: 343.2273, found: 343.2275.

---

[14] This particular gradient was used to recover unreacted diyne **SI-8** (75 mg, 13% yield).

$[\alpha]_D^{20.2}$ = −56.7 (c = 1.0, $CHCl_3$).

IR (thin film) ν_{max} (cm^{-1}): 3301 (br), 2953 (s), 2868 (m), 2359 (m), 2341 (m), 2113 (w), 1455 (w), 1427 (w), 1395 (w), 1364 (m), 1310 (w), 1265 (s), 1140 (w), 1104 (s), 1022 (w), 968 (m), 847 (w), 805 (w), 736 (s), 703 (m), 640 (w).

***R_f* =** 0.40 (1:1 hexanes:EtOAc; visualized with $KMnO_4$ stain).

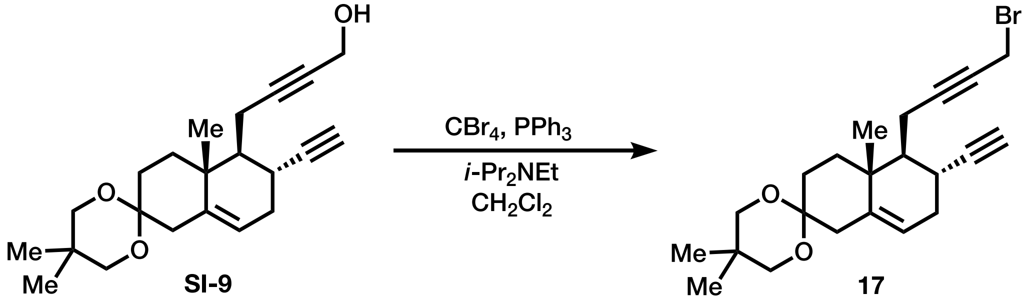

Ketalized propargyl bromide 17. To a solution of ketalized propargyl alcohol **SI-9** (0.362 g, 1.06 mmol, 1 equiv) in dry CH_2Cl_2 (25 mL) was added *N*,*N*-diisopropylethylamine (0.45 mL, 3.17 mmol, 3 equiv) at ambient temperature. The resultant clear solution was cooled to 0 °C and treated with a single portion of tetrabromomethane (0.422 g, 1.27 mmol, 1.2 equiv). The resultant clear, pale-yellow solution was stirred for 5 minutes at 0 °C, then treated portionwise with triphenylphosphine (0.417 g, 1.59 mmol, 1.5 equiv). The reaction mixture subsequently brought to ambient temperature and stirred for an additional 2 h. Upon complete consumption of the starting material as indicated by TLC, the mixture was diluted with CH_2Cl_2 (25 mL), treated with a saturated aqueous $NaHCO_3$ solution (20 mL), and extracted with CH_2Cl_2 (3 x 20 mL). The organic extracts were combined and dried over Na_2SO_4. Concentration of the dried extracts[15] provided a crude yellow oil, which was purified via flash column chromatography (12:1 hexanes:EtOAc) to afford propargyl bromide **17** (0.382 g, 89% yield) as an off-white, waxy oil.

^{1}H NMR (500 MHz, $CDCl_3$): δ 5.33 (m, 1H), 3.93 (t, *J* = 2.3 Hz, 2H), 3.58 (d, *J* = 11.2 Hz, 1H), 3.49 (d, *J* = 11.2 Hz, 1H), 3.45 (s, 2H), 2.72 – 2.59 (m, 3H), 2.59 – 2.54 (dd, *J* = 14.1, 3.2 Hz, 1H), 2.40 – 2.20 (m, 4H), 2.14 (d, *J* = 2.3 Hz, 1H), 1.96 – 1.90 (dt, *J* = 13.1, 3.5 Hz, 1H), 1.68 – 1.61 (ddd, *J* = 11.6, 6.5, 3.7 Hz, 1H), 1.61 – 1.52 (td, *J* = 14.1,

[15] Bath temperature was maintained at or below 25 °C, as product decomposition was observed at higher temperatures.

3.8 Hz, 1H), 1.38 – 1.28 (td, J = 13.7, 3.7 Hz, 1H), 1.10 (s, 3H), 1.00 (s, 3H), 0.91 (s, 3H).

^{13}C NMR (101 MHz, $CDCl_3$): δ 139.4, 120.9, 98.1, 88.3, 86.9, 76.4, 70.6, 70.4, 70.0, 48.1, 39.8, 38.5, 35.3, 33.1, 30.2, 27.5, 27.4, 22.9, 22.7, 18.9, 18.5, 16.2.

COSY/HSQC: see **Figure S59** / **Figure S60**.

HRMS: (ESI) *m/z* $[M+H]^+$ calcd for $C_{22}H_{29}BrO_2$: 405.1429, found: 405.1412.

$[\alpha]_D^{21.3}$ = −46.8 (c = 1.0, $CHCl_3$).

IR (thin film) ν_{max} (cm^{-1}): 3297 (s), 2923 (s), 2853 (s), 2359 (m), 2161 (w), 1552 (m), 1461 (m), 1393 (w), 1364 (w), 1259 (w), 1210 (w). 1106 (s), 1025 (s), 967 (m), 907 (w), 814 (w), 748 (m), 668 (s).

***R_f* =** 0.43 (6:1 hexanes:EtOAc; visualized with $KMnO_4$ stain).

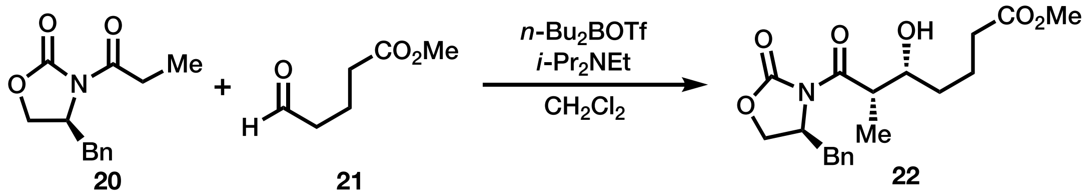

Alcohol 22. To a pre-cooled solution of (S)-(+)-4-Benzyl-3-propionyl-2-oxazolidinone (**20**) (5.77 g, 24.7 mmol, 1 equiv) in dry CH_2Cl_2 (120 mL) was added dropwise at −78 °C a solution of freshly distilled di-*n*-butylboron trifluoromethanesulfonate[16] (6.8 mL, 27.0 mmol, 1.1 equiv) in dry CH_2Cl_2 (13 mL). The resultant mixture was then slowly treated with freshly distilled *N*,*N*-diisopropylethylamine (6.0 mL, 34.4 mmol, 1.4 equiv) at −78 °C. The reaction mixture was stirred at −78 °C for 30 minutes, subsequently brought to 0 °C and stirred at this same temperature for 3 h, before re-cooling to −78 °C. A separate solution containing aldehyde **21** (3.54 g, 27.2 mmol, 1.1 equiv) in dry CH_2Cl_2 (30 mL) was cooled to −78 °C, then added dropwise to the reaction mixture via cannula transfer over 15 minutes. The resultant mixture was allowed to warm to ambient temperature and stirred for 20 h. Upon consumption of starting material as indicated by TLC, the reaction mixture was cooled to 0 °C, diluted with MeOH (120 mL) and 1.0 M

[16] Prepared from tri-*n*-butylborane (1 equiv) and trifluoromethanesulfonic acid (1 equiv) according to the following procedures: (a) Tan, I.; Mukaiyama, T. Regio- and Stereoselective Cross-aldol Reactions *via* Dialkylboryl Triflates. *Bull. Chem. Soc. Jpn.* **1980**, *53*, 174−178. (b) Evans, D. A.; Nelson, J. V.; Vogel, E.; Taber, T. R. Stereoselective aldol condensations via boron enolates. *J. Am. Chem. Soc.* **1981**, *103*, 3099−3111.

aqueous K_2HPO_4 — H_3PO_4 buffer solution (pH 7; 30 mL), and finally slowly treated with a 1:1 MeOH / 30% (w/w) aqueous H_2O_2 solution (60 mL). The resultant mixture was vigorously stirred at 0 °C for 2 h. The mixture was subsequently extracted with CH_2Cl_2 (4 x 150 mL) and the organic extracts were combined and dried over Na_2SO_4. Concentration of the dried extracts provided a crude orange oil, which was purified via flash column chromatography (3:2 hexanes:EtOAc) to afford alcohol **22** (8.57 g, 95% yield) as a pale-yellow oil.

^{1}H NMR (500 MHz, $CDCl_3$): δ 7.34 (m, 2H), 7.28 (m, 1H), 7.21 (m, 2H), 4.71 (m, 1H), 4.28 – 4.17 (m, 2H), 3.97 (m, 1H), 3.75 (qd, *J* = 7.0, 2.7 Hz, 1H), 3.67 (s, 3H), 3.25 (dd, *J* = 13.4, 3.2 Hz, 1H), 2.93 (br s, 1H), 2.79 (dd, *J* = 13.4, 9.5 Hz, 1H), 2.42 – 2.31 (m, 2H), 1.90 – 1.80 (m, 1H), 1.75 – 1.65 (m, 1H), 1.63 – 1.52 (m, 1H), 1.50 – 1.41 (m, 1H), 1.26 (d, *J* = 7.1 Hz, 3H).

^{13}C NMR (125 MHz, $CDCl_3$): δ 177.5, 174.1, 153.2, 135.1, 129.6, 129.1, 127.6, 71.2, 66.3, 55.2, 51.7, 42.4, 37.9, 33.9, 33.3, 21.5, 10.7.

COSY/HSQC: see **Figure S63** / **Figure S64**.

HRMS: (ESI) *m/z* $[M+H]^+$ calcd for $C_{19}H_{25}NO_6$: 364.1760, found: 364.1771.

$[\alpha]_D^{23.6}$ = +44.2 (*c* = 1.0, $CHCl_3$).

IR (thin film) ν_{max} (cm^{-1}): 3512 (br), 2949 (m), 1772 (s), 1731 (s), 1692 (m), 1454 (w), 1383 (m), 1350 (m), 1207 (s), 1108 (m), 971 (m), 762 (w), 750 (w), 702 (s).

***R_f* =** 0.44 (1:1 hexanes:EtOAc; visualized with UV and $KMnO_4$ stain).

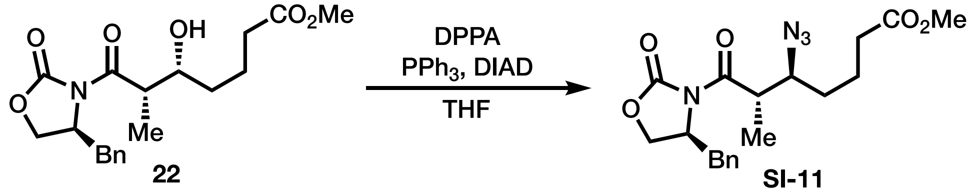

Azide SI-11. To a solution of triphenylphosphine (4.96 g, 18.9 mmol, 1.5 equiv) in dry THF (120 mL) was added diisopropyl azodicarboxylate (3.7 mL, 18.9 mmol, 1.5 equiv) dropwise at 0 °C. The resultant mixture was stirred at 0 °C for 15 minutes, whereupon a solution of alcohol **22** (4.58 g, 12.5 mmol, 1 equiv) in dry THF (100 mL) was added slowly over 15 minutes at 0 °C. This mixture was stirred at 0 °C for 20 minutes, followed by subsequent treatment with diphenylphosphoryl azide (4.1 mL, 19.0 mmol, 1.5 equiv) dropwise, and stirred at 0 °C for an additional 30 minutes. The resultant turbid beige

mixture was then allowed to warm to ambient temperature and stirred for 10 h, slowly changing to a clear, pale yellow solution. Upon consumption of starting material as indicated by TLC, the reaction mixture was treated with a saturated aqueous $NaHCO_3$ solution (200 mL) and extracted with 1:1 hexanes:EtOAc (3 x 200 mL). The organic extracts were then combined, washed with brine (1 x 250 mL), and dried over Na_2SO_4. Concentration of the dried extracts[17] provided a crude yellow oil, which was purified via flash column chromatography (4:1:0 hexanes:CH_2Cl_2:EtOAc → 4:1:0.2 hexanes:CH_2Cl_2:EtOAc → 4:1:0.5 hexanes:CH_2Cl_2:EtOAc → 4:1:1 hexanes:CH_2Cl_2:EtOAc) to afford azide **SI-11** (4.13 g, 85% yield) as a clear, colorless oil.

^{1}H NMR (400 MHz, $CDCl_3$): δ 7.37 – 7.27 (m, 3H), 7.22 (m, 2H), 4.74 (m, 1H), 4.27 (dd, *J* = 8.9, 7.9 Hz, 1H), 4.20 (dd, *J* = 9.1, 2.6 Hz, 1H), 3.92 – 3.83 (m, 1H), 3.78 – 3.70 (td, *J* = 9.1, 2.8 Hz, 1H), 3.69 (s, 3H), 3.26 (dd, *J* = 13.4, 3.4 Hz, 1H), 2.80 (dd, *J* = 13.4, 9.5 Hz, 1H), 2.38 (m, 2H), 1.94 – 1.82 (m, 1H), 1.79 – 1.67 (m, 2H), 1.56 – 1.48 (m, 1H), 1.23 (d, *J* = 6.9 Hz, 3H).

^{13}C NMR (125 MHz, $CDCl_3$): δ 174.7, 173.6, 153.1, 135.2, 129.6, 129.1, 127.6, 66.4, 64.3, 55.4, 51.8, 42.1, 38.0, 33.7, 30.6, 21.1, 14.7.

COSY/HSQC: see **Figure S67** / **Figure S68**.

HRMS: (ESI) *m/z* $[M+H]^+$ calcd for $C_{19}H_{24}N_4O_5$: 389.1825, found: 389.1824.

$[\alpha]_D^{24.8}$ = +72.0 (*c* = 1.0, $CHCl_3$).

IR (thin film) ν_{max} (cm^{-1}): 2951 (m), 2099 (s), 1775 (s), 1733 (s), 1693 (s), 1454 (w), 1386 (m), 1349 (w), 1209 (s), 1110 (m), 972 (m), 762 (m), 749 (m), 703 (s).

***R_f* =** 0.54 (2:1 hexanes:EtOAc; visualized with $KMnO_4$).

[17] Bath temperature was maintained at or below 30 – 35 °C, as extensive product decomposition was observed at higher temperatures.

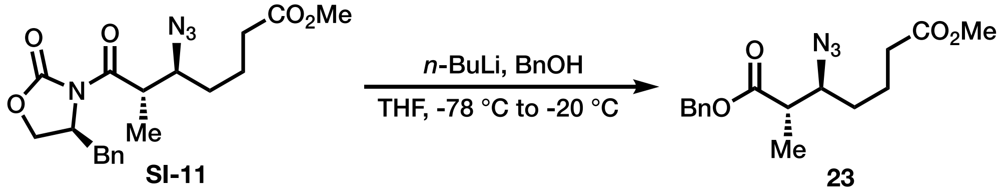

Benzyl ester 23. To pre-cooled a solution of freshly distilled benzyl alcohol (2.0 mL, 19.2 mmol, 2.0 equiv) in dry THF (30 mL) was added *n*-butyllithium solution (1.60 M in hexanes; 10.5 mL, 16.8 mmol, *ca.* 1.8 equiv) dropwise at −78 °C. The resultant mixture was stirred at −78 °C for 15 minutes, then allowed to warm to 0 °C, stirred at this same temperature for 10 minutes, and subsequently re-cooled to −40 °C. A solution of azide **SI-11** (3.70 g, 9.50 mmol, 1 equiv) in dry THF (50 mL) was added dropwise over 15 minutes at −40 °C to the solution containing LiOBn. The resultant yellow solution was stirred at −40 °C for 30 minutes, then allowed to slowly warm to −20 °C and stirred at this same temperature for an additional 3 h. Upon consumption of starting material as indicated by TLC, the reaction mixture was quenched with a saturated aqueous NH_4Cl solution (50 mL) at −20 °C and allowed to warm to ambient temperature. The mixture was extracted with 1:1 hexanes:EtOAc (3 x 150 mL) and the organic extracts were combined, washed with brine (1 x 200 mL), and dried over Na_2SO_4. Concentration of the dried extracts provided a crude yellow oil, which was purified via flash column chromatography (8:1 hexanes:EtOAc) to afford benzyl ester **23** (2.38 g, 78% yield) as a clear, colorless oil.

^{1}H NMR (400 MHz, $CDCl_3$): δ 7.41 – 7.30 (m, 5H), 5.19 (d, *J* = 12.3 Hz, 1H), 5.14 (d, *J* = 12.3 Hz, 1H), 3.67 (s, 3H), 3.60 (m, 1H), 2.72 – 2.63 (pent, *J* = 7.3 Hz, 1H), 2.40 – 2.26 (m, 2H), 1.88 – 1.75 (m, 1H), 1.74 – 1.44 (m, 3H), 1.19 (d, *J* = 7.1 Hz, 3H).

^{13}C NMR (125 MHz, $CDCl_3$): δ 173.8, 173.6, 135.8, 128.8, 128.5, 128.4, 66.8, 64.4, 51.8, 44.2, 33.6, 30.6, 21.4, 13.7.

COSY/HSQC: see **Figure S71** / **Figure S72**.

HRMS: (ESI) *m/z* $[M+H]^+$ calcd for $C_{16}H_{21}N_3O_4$: 320.1610, found: 320.1598.

$[\alpha]_D^{20.7}$ = +5.7 (*c* = 1.0, $CHCl_3$).

IR (thin film) ν_{max} (cm^{-1}): 2951 (m), 2098 (s), 1731 (s), 1455 (m), 1436 (w), 1249 (s), 1167 (s), 1074 (w), 1028 (w), 911 (w), 752 (s), 698 (s).

***R_f* =** 0.45 (4:1 hexanes:EtOAc; visualized with $KMnO_4$ stain).

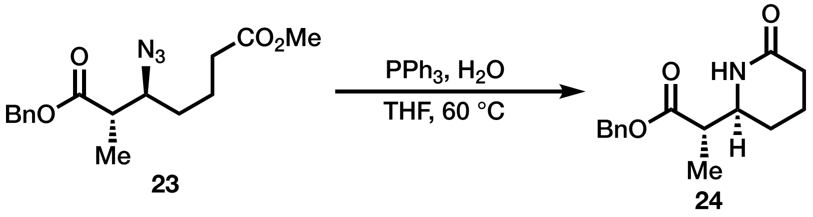

Lactam 24. To a solution of azide **23** (2.38 g, 7.45 mmol, 1 equiv) in THF (220 mL) was added distilled water (1.35 mL, 75 mmol, 10 equiv) and triphenylphosphine (2.34 g, 8.94 mmol, 1.2 equiv) at ambient temperature. The resultant mixture was heated to 60 °C and stirred at that same temperature for 24 h. Upon consumption of starting material, as indicated by TLC, the mixture was cooled to ambient temperature and concentrated in vacuo to afford a crude yellow solid, which was purified via flash column chromatography (1:1:0.5 hexanes:CH_2Cl_2:acetone → 1:1:1 hexanes:CH_2Cl_2:acetone) to afford lactam **24** (1.73 g, 89% yield) as an off-white solid.

^{1}H NMR (400 MHz, $CDCl_3$): δ 7.41 – 7.32 (m, 5H), 6.07 (br s, 1H), 5.17 (d, *J* = 12.3 Hz, 1H), 5.13 (d, *J* = 12.3 Hz, 1H), 3.63 (m, 1H), 2.59 – 2.51 (pent, *J* = 7.3 Hz, 1H), 2.43 – 2.35 (m, 1H), 2.30 – 2.21 (m, 1H), 1.96 – 1.84 (m, 2H), 1.75 – 1.64 (m, 1H), 1.50 – 1.40 (m, 1H), 1.20 (d, *J* = 7.3 Hz, 3H).

^{13}C NMR (101 MHz, $CDCl_3$): δ 174.1, 172.2, 135.6, 128.8, 128.6, 128.4, 66.9, 54.5, 45.5, 31.5, 25.2, 19.4, 13.5.

COSY/HSQC: see **Figure S75** / **Figure S76**.

HRMS: (ESI) *m/z* $[M+H]^+$ calcd for $C_{15}H_{19}NO_3$: 262.1443, found: 262.1453.

$[\alpha]_D^{22.9}$ = +5.5 (*c* = 1.0, $CHCl_3$).

IR (thin film) ν_{max} (cm^{-1}): 2947 (m), 1728 (s), 1656 (s), 1454 (m), 1405 (w), 1349 (m), 1309 (w), 1150 (s), 1065 (m), 958 (m), 735 (s), 697 (s).

***R_f* =** 0.50 (1:1 $CHCl_3$:acetone; visualized with $Ce(SO_4)_2$ in phosphomolybdic acid stain).

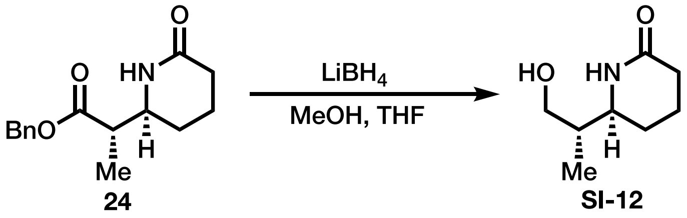

Alcohol SI-12. To a pre-cooled solution of benzyl ester **24** (1.60 g, 6.12 mmol, 1 equiv) in dry THF (60 mL) was added lithium borohydride (0.675 g, 31.0 mmol, 5 equiv) in a single portion at 0 °C, followed by anhydrous methanol (1.50 mL, 37.1 mmol, 6 equiv) dropwise at the same temperature. The resultant, cloudy white mixture was allowed to warm to ambient temperature and stirred for 24 h. Upon consumption of starting material as indicated by TLC, the mixture was diluted with Et_2O (50 mL) and slowly treated with distilled water (20 mL). The resultant biphasic mixture was separated, and the aqueous layer extracted with Et_2O (2 x 40 mL). The *aqueous* extracts were concentrated in vacuo to afford a whitish residue, which was subsequently azeotroped with benzene (3 x 5 mL) to remove remaining traces of water. The resulting white solid was resuspended in MeOH (10 mL) and filtered through a layer of Celite, washing the filter cake thoroughly with ice-cold MeOH (50 mL). Concentration of the filtrate provided a crude, off-white solid, which was purified via flash column chromatography (6% MeOH in CH_2Cl_2) to afford alcohol **SI-12** (0.908 g, 94% yield) as a white, crystalline solid.

^{1}H NMR (400 MHz, $CDCl_3$): δ 7.96 (br s, 1H), 5.13 (br s, 1H), 3.81 (dd, *J* = 10.7, 3.6 Hz, 1H), 3.53 (t, *J* = 9.8 Hz, 1H), 3.34 – 3.25 (td, *J* = 9.8, 3.6 Hz, 1H), 2.42 – 2.31 (m, 1H), 2.30 – 2.19 (m, 1H), 1.98 – 1.85 (m, 2H), 1.79 – 1.60 (m, 2H), 1.39 – 1.28 (m, 1H), 0.83 (d, *J* = 6.9 Hz).

^{13}C NMR (125 MHz, $CDCl_3$): δ 172.8, 67.9, 58.8, 40.1, 31.0, 26.6, 20.0, 13.3.

COSY/HSQC: see **Figure S79** / **Figure S80**.

HRMS: (ESI) *m/z* $[M+H]^+$ calcd for $C_8H_{15}NO_2$: 158.1181, found: 158.1188.

$[\alpha]_D^{22.8}$ = −44.3 (*c* = 1.0, MeOH).

IR (thin film) ν_{max} (cm^{-1}): 3275 (s), 2956 (m), 1625 (s), 1474 (m), 1408 (s), 1349 (m), 1329 (m), 1309 (m), 1168 (w), 1031 (s), 982 (w), 670 (s).

***R_f* =** 0.36 (10% MeOH in CH_2Cl_2; visualized with $KMnO_4$ stain).

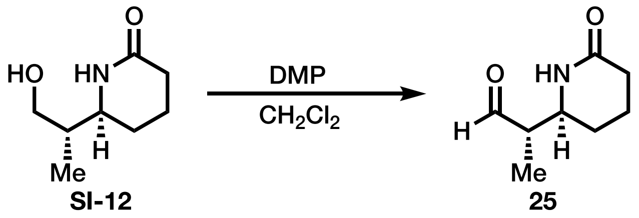

Aldehyde 25. To a solution of alcohol **SI-12** (0.311 g, 1.98 mmol, 1 equiv) in CH_2Cl_2 (70 mL) was added Dess-Martin periodinane (1.09 g, 2.57 mmol, 1.3 equiv) in a single portion at ambient temperature. The resultant cloudy white mixture was stirred at ambient temperature for 3 h. Upon consumption of the starting material as indicated by TLC, the reaction mixture was treated with solid $NaHCO_3$ (0.490 g), diluted with 1:1 hexanes:Et_2O (150 mL), then filtered through a layer of Celite while washing the filter cake thoroughly with Et_2O (ca. 100 mL). Concentration of the filtrate afforded a crude off-white solid, which was purified via flash column chromatography (4:1 $CHCl_3$:acetone) to afford aldehyde **25**[18] (0.303 g, 98% yield) as an amorphous, white solid.

^{1}H NMR (500 MHz, $CDCl_3$): δ 9.68 (s, 1H), 6.68 (br s, 1H), 3.78 – 3.70 (m, 1H), 2.60 – 2.50 (pent, J = 7.5 Hz, 1H), 2.45 – 2.35 (m, 1H), 2.32 – 2.21 (m, 1H), 1.96 – 1.84 (m, 2H), 1.79 – 1.66 (m, 1H), 1.52 – 1.40 (m, 1H), 1.18 (d, J = 7.5 Hz, 3H).

^{13}C NMR (101 MHz, $CDCl_3$): δ 203.0, 172.5, 52.8, 51.5, 31.5, 25.2, 19.6, 9.7.

COSY/HSQC: see **Figure S83** / **Figure S84**.

HRMS: (ESI) m/z $[M+H]^+$ calcd for $C_8H_{13}NO_2$: 156.1024, found: 156.1026.

IR (thin film) ν_{max} (cm^{-1}): 3213 (br), 2980 (m), 2873 (m), 1719 (s), 1659 (s), 1481 (w), 1402 (m), 1353 (s), 1319 (m), 1200 (w), 1173 (m), 1063 (w), 983 (s), 955 (w), 790 (m), 678 (w).

$[\alpha]_D^{24.7}$ = −36.7 (c = 1.0, MeOH).

R_f = 0.33 (1:2 $CHCl_3$:acetone; visualized with $Ce(SO_4)_2$ in phosphomolybdic acid stain).

---

[18] This material was found to be sparingly soluble in most organic solvents and extremely prone to epimerization. Upon exposure to protic solvents, a diastereomeric mixture of hemiacetals is readily formed.

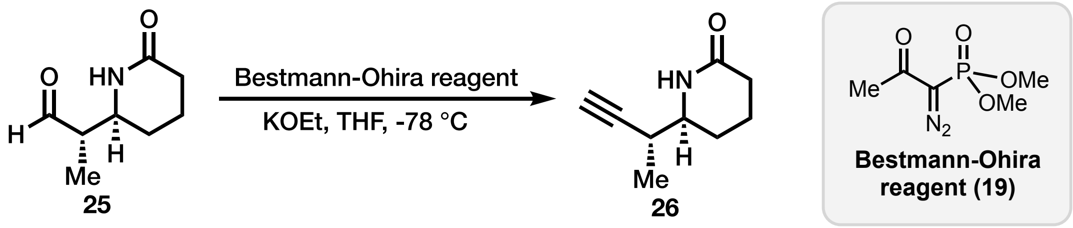

Alkyne 26. To a pre-cooled solution of potassium ethoxide (0.431 g, 5.12 mmol, 3.3 equiv) in THF (40 mL) was added dropwise over 10 minutes a solution of Bestmann-Ohira reagent (1.19 g, 6.20 mmol, 4 equiv) in THF (8.0 mL) at −78 °C. After complete addition, the resultant opaque, bright yellow mixture was stirred at −78 °C for 20 minutes. Aldehyde **25** (0.240 g, 1.55 mmol, 1 equiv) was then added to the reaction mixture in a single portion at −78 °C and the resultant mixture was vigorously stirred for 10 minutes before allowing the solution to warm to −50 °C. The reaction mixture was then stirred at −50 °C for 1.5 h before allowing to warm to 0 °C, then quenched with saturated aqueous NH_4Cl solution (50 mL) and further diluted with H_2O (50 mL). The mixture was extracted with EtOAc (6 x 30 mL). The organic extracts were combined and washed with brine (1 x 100 mL), then dried over Na_2SO_4. Concentration of the dried organic extracts provided a crude yellow residue, which was purified via flash column chromatography (100% EtOAc) to afford alkyne **26** (0.211 g, 90% yield) as a white, crystalline solid.

^{1}H NMR (400 MHz, $CDCl_3$): δ 6.27 (s, 1H), 3.26 (m, 1H), 2.48 – 2.38 (m, 2H), 2.33 – 2.23 (m, 1H), 2.19 (d, *J* = 2.3 Hz, 1H), 1.98 – 1.91 (m, 1H), 1.75 – 1.57 (m, 2H), 1.42 – 1.32 (m, 1H), 1.22 (d, *J* = 7.0 Hz, 3H).

^{13}C NMR (101 MHz, $CDCl_3$): δ 172.2, 84.7, 71.7, 56.9, 32.7, 31.4, 26.3, 19.8, 16.6.

COSY/HSQC: see **Figure S87** / **Figure S88**.

HRMS: (ESI) *m*/*z* $[M+H]^+$ calcd for $C_9H_{13}NO$: 152.1075, found: 152.1076.

IR (thin film) ν_{max} (cm^{-1}): 3221 (s), 2949 (m), 2876 (m), 2128 (w), 1648 (s), 1451 (m), 1405 (m), 1349 (m), 1335 (m), 1296 (w), 1169 (m), 1070 (m), 976 (w), 952 (w), 670 (s).

$[\alpha]_D^{23.9}$ = +34.5 (*c* = 1.0, $CHCl_3$).

***R_f* =** 0.45 (1:1 $CHCl_3$:acetone; visualized with $KMnO_4$ stain).

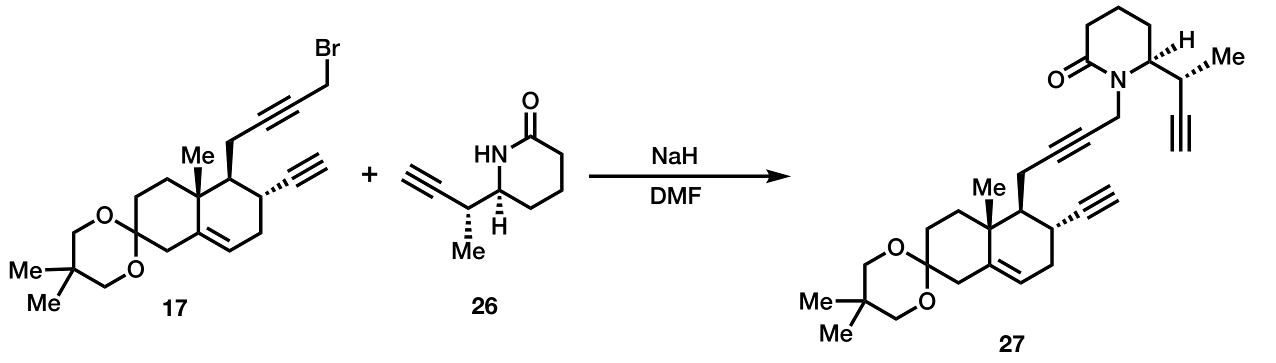

Triyne 27. To a solution of propargyl bromide **17** (0.262 g, 0.646 mmol, 1 equiv) and alkyne **26** (0.098 g, 0.650 mmol, 1 equiv) in anhydrous DMF (6.0 mL) was added sodium hydride (60% dispersion in mineral oil; 0.039 g, 0.990 mmol, 1.5 equiv) in a single portion at 0 °C. The resultant mixture was allowed to warm to ambient temperature and stirred for 18 h. The mixture was then diluted with Et_2O (15 mL) and quenched with a saturated aqueous $NaHCO_3$ solution (10 mL). Following further dilution with water (50 mL), the mixture was extracted with EtOAc (3 x 40 mL), then the organic extracts were combined, washed with brine (1 x 100 mL), and dried over Na_2SO_4. Concentration of the dried extracts provided a crude yellow oil, which was purified via flash column chromatography (35:10:6.0 hexanes:CH_2Cl_2:acetone) to afford triyne **27** (0.286 g, 93% yield) as a foamy, white solid.

^{1}H NMR (400 MHz, $CDCl_3$): δ 5.33 (m, 1H), 5.01 (dt, *J* = 17.5, 2.2 Hz, 1H), 4.01 – 3.94 (dt, *J* = 7.5, 5.6 Hz, 1H), 3.63 (app d, *J* = 17.5 Hz, 1H), 3.58 (d, *J* = 11.3 Hz, 1H), 3.49 (d, *J* = 11.3 Hz, 1H), 3.44 (s, 2H), 3.11 – 3.02 (m, 1H), 2.73 – 2.62 (m, 1H), 2.62 – 2.56 (m, 2H), 2.56 – 2.50 (m, 1H), 2.50 – 2.41 (dtd, *J* = 17.5, 5.6, 1.4 Hz, 1H), 2.41 – 2.18 (m, 5H), 2.15 – 2.06 (m, 1H), 2.13 (d, *J* = 2.4 Hz, 1H), 2.11 (d, *J* = 2.5 Hz, 1H), 1.93 – 1.84 (m, 2H), 1.84 – 1.57 (m, 3H), 1.57 – 1.51 (dd, *J* = 14.0, 3.9 Hz, 1H), 1.36 – 1.26 (td, *J* = 13.7, 3.8 Hz, 1H), 1.12 (d, *J* = 7.1 Hz, 3H), 1.10 (s, 3H), 1.00 (s, 3H), 0.91 (s, 3H).

^{13}C NMR (101 MHz, $CDCl_3$): δ 171.0, 139.4, 121.0, 98.2, 87.2, 85.8, 84.5, 75.7, 70.8, 70.4, 70.4, 70.0, 57.7, 47.9, 39.9, 38.5, 35.3, 33.8, 33.1, 32.6, 30.2, 28.2, 27.5, 27.4, 24.3, 22.9, 22.7, 19.0, 18.7, 18.2, 15.1.

COSY/HSQC: see **Figure S91** / **Figure S92**.

HRMS: (ESI) *m*/*z* $[M+H]^+$ calcd for $C_{31}H_{41}NO_3$: 476.3164, found: 476.3166.

$[\alpha]_D^{23.7}$ = −43.9 (*c* = 1.0, $CHCl_3$).

IR (thin film) ν_{max} (cm^{-1}): 3291 (s), 2950 (s), 2867 (m), 2112 (w), 1640 (s), 1452 (m), 1415 (w), 1394 (w), 1336 (m), 1308 (w), 1243 (m), 1200 (w), 1161 (w), 1140 (w), 1105 (s), 1026 (m), 967 (m), 906 (w), 805 (m), 735 (s), 700 (m), 637 (m).

***R_f* =** 0.47 (1:1 hexanes:EtOAc; visualized with $Ce(SO_4)_2$ in phosphomolybdic acid stain).

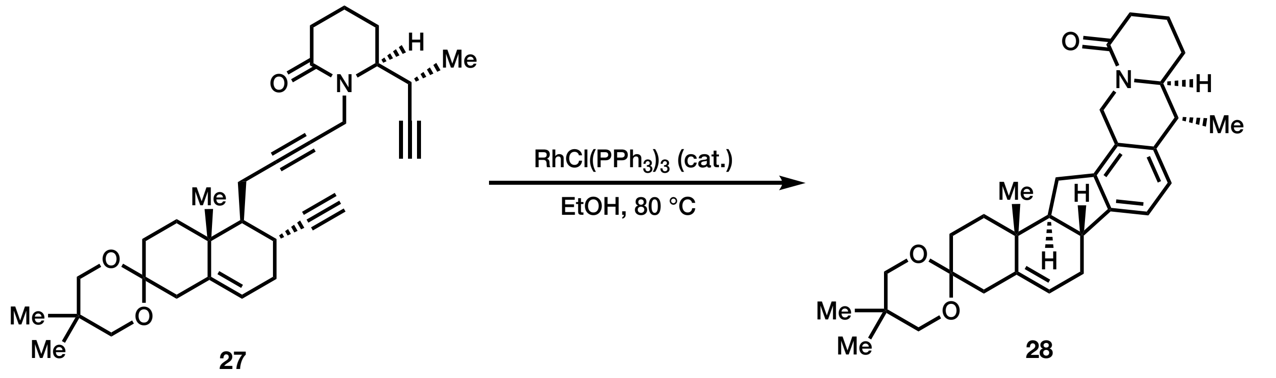

Hexacycle 28. To a solution of triyne **27** (0.286 g, 0.601 mmol, 1 equiv) in dry, degassed EtOH[19] (12 mL) was added $RhCl(PPh_3)_3$ (56 mg, 0.060 mmol, 10 mol%) at ambient temperature under an atmosphere of Ar. The initially heterogeneous mixture was heated to 80 °C, whereupon a clear, red amber solution resulted and was stirred at this temperature for 30 minutes. Upon consumption of the starting material as indicated by TLC, the mixture was brought back to ambient temperature. Concentration of the reaction mixture afforded a crude residue, which was redissolved in benzene (5 mL) and diluted with cyclohexane (40 mL) to precipitate impurities. The mixture was then filtered through a layer of Celite (washing with ca. 60 mL of 10:1 cyclohexane:benzene) and the filtrate concentrated in vacuo. The resultant light-orange solid was purified via flash column chromatography (3:1:1 hexanes:acetone:Et_2O) to afford hexacycle **28** (0.252 g, 89% yield) as a light-yellow solid.

^{1}H NMR (400 MHz, $CDCl_3$): δ 7.13 (d, *J* = 7.8 Hz, 1H), 7.04 (d, *J* = 7.8 Hz, 1H), 5.49 (m, 1H), 5.22 (d, *J* = 17.2 Hz, 1H), 4.17 (d, *J* = 17.2 Hz, 1H), 3.63 (d, *J* = 11.5 Hz, 1H), 3.50 (d, *J* = 11.5 Hz, 1H), 3.47 (s, 2H), 3.31 – 3.22 (m, 1H), 3.03 – 2.91 (td, *J* = 11.5, 5.4 Hz, 1H), 2.91 – 2.81 (m, 1H), 2.72 (dd, *J* = 15.1, 7.5 Hz, 1H), 2.68 – 2.54 (m, 3H), 2.54 – 2.29 (m, 4H), 2.23 – 2.04 (m, 2H), 1.99 – 1.86 (m, 2H), 1.84 – 1.57 (m, 4H), 1.48 – 1.37 (m, 1H), 1.34 (d, *J* = 6.8 Hz, 3H), 1.14 (s, 3H), 1.04 (s, 3H), 0.90 (s, 3H).

^{13}C NMR (101 MHz, $CDCl_3$): δ 169.7, 144.7, 141.6, 140.5, 136.6, 129.1, 124.4, 122.4, 121.1, 98.7, 70.4, 70.1, 59.7, 56.9, 43.4, 41.0, 40.4, 38.4, 37.6, 35.9, 32.7, 30.4, 30.3, 29.3, 27.2, 27.0, 23.0, 22.7, 19.1, 18.7, 17.1.

[19] Solvent was degassed by sparging with argon (ca. 4 L) for 30 minutes.

COSY/HSQC: see **Figure S95** / **Figure S96**.

HRMS: (ESI) *m/z* $[M+H]^+$ calcd for $C_{31}H_{41}NO_3$: 476.3164, found: 476.3163.

$[\alpha]_D^{21.3}$ **=** +64.2 (*c* = 1.0, $CHCl_3$).

IR (thin film) ν_{max} (cm^{-1}): 2923 (s), 2853 (s), 1763 (m), 1621 (s), 1462 (s), 1361 (m), 1302 (w), 1260 (s), 1169 (w), 1105 (s), 1029 (m), 978 (w), 807 (s), 748 (s), 668 (w).

***R_f* =** 0.26 (1:4 hexanes:EtOAc; visualized with $Ce(SO_4)_2$ in phosphomolybdic acid stain).

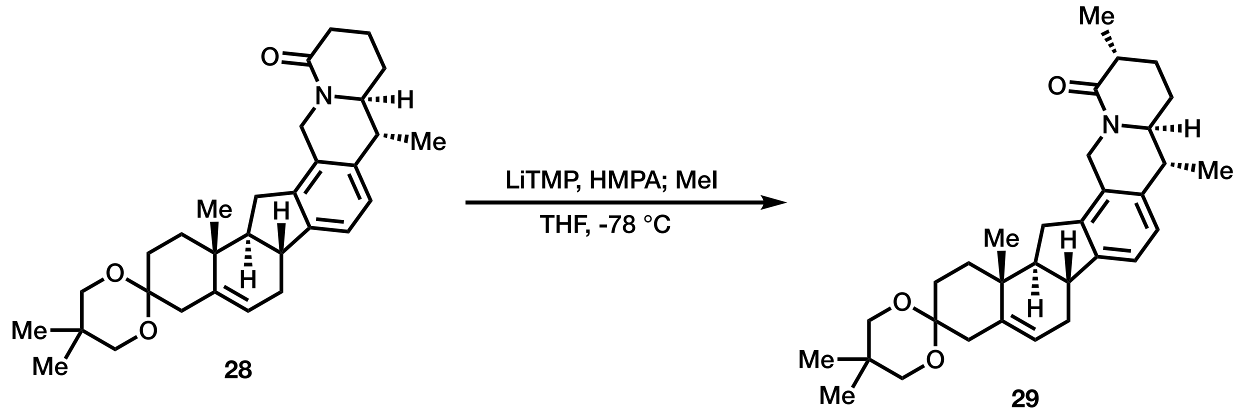

28 **29**

α-Methyl lactam 29.[20] To a pre-cooled solution of freshly distilled 2,2,6,6,-tetramethylpiperidine (1.20 mL, 7.05 mmol) in THF (3.0 mL) was added dropwise *n*-butyllithium solution (1.53 M in hexanes; 4.50 mL, 6.89 mmol) at 0 °C. The resultant pale-yellow solution was stirred at 0 °C for 30 minutes to afford a 0.81 M solution of LiTMP. A separate round-bottom flask was charged with hexacycle **28** (40 mg, 0.084 mmol, 1 equiv) and THF (4.0 mL), then cooled to −78 °C under an atmosphere of Ar. The resultant solution was first treated with freshly distilled hexamethylphosphoramide (0.35 mL) at −78 °C, followed by dropwise addition of the 0.81 M LiTMP solution (0.32 mL, 0.259 mmol, ca. 3 equiv). The orangish brown mixture was stirred at −78 °C for 20 minutes, then iodomethane (0.04 mL, 0.643 mmol, ca. 8 equiv) was slowly added. The resultant pale-yellow solution was stirred for 1 minute, then rapidly quenched with MeOH (0.06 mL) at −78 °C. The mixture was then brought to 0 °C, treated with a 1:1 H_2O:saturated aqueous $NaHCO_3$ solution (3 mL), and subsequently diluted with EtOAc

[20] In order to ensure reproducibility, this reaction was run batchwise on scales of 50 mg or less. Larger reaction scales occasionally led to slightly diminished yields owing to increased formation of dimethylated side product **SI-13/SI-13'**, as a ca. 4:1 mixture of inseparable C–18 epimers (See **Figures S101** – **S104** for spectral data).

(10 mL). The biphasic mixture was separated and aqueous layer subsequently extracted with EtOAc (3 x 15 mL). All organic extracts were then combined, washed with brine (1 x 30 mL), and dried over Na_2SO_4. Concentration of the dried extracts provided a crude colorless residue, which was purified via flash column chromatography (3:1 hexanes:EtOAc) to afford α-methyl lactam **29** (7:1 dr; 26 mg, 63% yield of single isolated diastereomer) as a white solid.

^{1}H NMR (400 MHz, $CDCl_3$): δ 7.12 (d, *J* = 7.8 Hz, 1H), 7.04 (d, *J* = 7.8 Hz, 1H), 5.49 (m, 1H), 5.10 (d, *J* = 17.5 Hz, 1H), 4.23 (d, *J* = 17.5 Hz, 1H), 3.63 (d, *J* = 11.4 Hz, 1H), 3.50 (d, *J* = 11.4 Hz, 1H), 3.47 (s, 2H), 3.26 – 3.16 (dt, *J* = 9.3, 5.6 Hz, 1H), 3.03 – 2.92 (td, *J* = 11.7, 5.2 Hz, 1H), 2.84 – 2.69 (m, 2H), 2.68 – 2.52 (m, 3H), 2.48 – 2.25 (m, 4H), 2.15 – 2.05 (m, 1H), 2.05 – 1.97 (m, 1H), 1.97 – 1.87 (td, *J* = 11.9, 7.5 Hz, 1H), 1.73 – 1.56 (m, 3H), 1.55 – 1.37 (m, 2H), 1.34 (d, *J* = 6.8 Hz, 3H), 1.28 (d, *J* = 7.1 Hz, 3H), 1.14 (s, 3H), 1.04 (s, 3H), 0.90 (s, 3H).

^{13}C NMR (101 MHz, $CDCl_3$): δ 173.2, 144.7, 141.6, 140.5, 136.6, 129.2, 123.8, 122.4, 121.0, 98.7, 70.4, 70.0, 60.2, 56.9, 43.6, 41.0, 40.4, 39.3, 37.5, 36.8, 35.9, 30.4, 30.2, 29.1, 27.7, 27.1, 26.9, 23.0, 22.7, 19.1, 17.9, 16.2.

COSY/HSQC: see **Figure S99** / **Figure S100**.

HRMS: (ESI) *m*/*z* $[M+H]^+$ calcd for $C_{32}H_{43}NO_3$: 490.3321, found: 490.3325.

$[\alpha]_D^{22.2}$ = +72.0 (*c* = 1.0, $CHCl_3$).

IR (thin film) ν_{max} (cm^{-1}): 2954 (m), 2924 (s), 2855 (s), 1641 (s), 1558 (m), 1458 (s), 1393 (w), 1361 (m), 1260 (s), 1211 (w), 1105 (s), 1030 (m), 977 (m), 807 (s), 749 (m), 668 (w).

***R_f* =** 0.49 (1:1 hexanes:EtOAc; visualized with $Ce(SO_4)_2$ in phosphomolybdic acid stain).

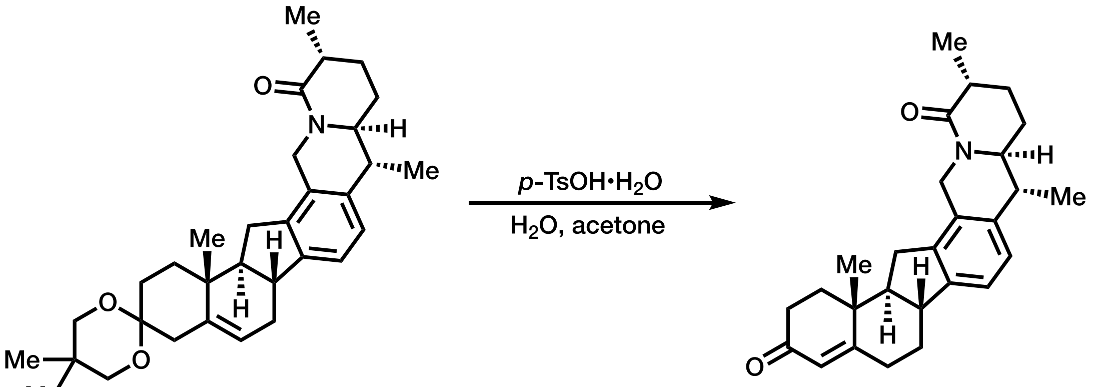

29 **30**

Enone 30. To pre-cooled solution of α-methylated lactam **29** (36 mg, 0.074 mmol, 1 equiv) in acetone (1.5 mL) was added distilled water (50 µL), followed by *p*-toluenesulfonic acid (35 mg, 0.184 mmol, 2.5 equiv) in a single portion at 0 °C. The resultant yellow solution was stirred at 0 °C for 3 h, then brought to ambient temperature and stirred for an additional 12 h. Following consumption of starting material as indicated by TLC, the clear and colorless reaction mixture was neutralized with a saturated aqueous $NaHCO_3$ solution (1 mL) and diluted with EtOAc (8 mL). The resultant biphasic mixture was separated and the aqueous layer extracted with EtOAc (3 x 10 mL). The organic extracts were combined, washed with brine (1 x 20 mL), and dried over Na_2SO_4. Concentration of the dried extracts provided enone **30** (29 mg, 98% yield) as a white solid. The crude concentrate was typically clean enough to be carried forward without further purification, however flash column chromatography (12:1 $CHCl_3$:acetone) was used to obtain spectroscopically pure material.

^{1}H NMR (400 MHz, $CDCl_3$): δ 7.15 (d, *J* = 7.8 Hz, 1H), 7.05 (d, *J* = 7.8 Hz, 1H), 5.83 (s, 1H), 5.13 (d, *J* = 17.5 Hz, 1H), 4.20 (d, *J* = 17.5 Hz, 1H), 3.25 – 3.17 (dt, *J* = 9.3, 5.5 Hz, 1H), 3.12 – 3.02 (app t, *J* = 11.7 Hz, 1H), 2.85 – 2.75 (m, 1H), 2.75 – 2.67 (dd, *J* = 14.8, 7.0 Hz, 1H), 2.67 – 2.53 (m, 3H), 2.53 – 2.36 (m, 4H), 2.35 – 2.26 (m, 1H), 2.07 – 1.97 (m, 2H), 1.92 – 1.82 (td, *J* = 14.3, 4.5 Hz, 1H), 1.83 – 1.74 (td, *J* = 12.0, 7.1 Hz, 1H), 1.73 – 1.63 (m, 1H), 1.55 – 1.44 (m, 2H), 1.34 (d, *J* = 6.8 Hz, 3H), 1.30 (s, 3H), 1.28 (d, *J* = 7.1 Hz, 3H).

^{13}C NMR (101 MHz, $CDCl_3$): δ 199.5, 173.2, 169.7, 143.5, 139.7, 137.0, 129.5, 125.4, 124.1, 120.6, 60.2, 59.9, 43.5, 43.4, 39.3, 38.6, 36.8, 36.2, 33.9, 33.3, 29.7, 28.8, 27.6, 27.0, 17.9, 17.0, 16.4.

COSY/HSQC: see **Figure S107** / **Figure S108**.

HRMS: (ESI) *m/z* $[M+H]^+$ calcd for $C_{27}H_{33}NO_2$: 404.2589, found: 404.2586.

$[\alpha]_D^{21.4}$ = +197.9 (*c* = 1.0, $CHCl_3$).

IR (thin film) ν_{max} (cm^{-1}): 2927 (s), 2858 (s), 1666 (s), 1638 (s), 1461 (m), 1432 (m), 1377 (w), 1345 (w), 1326 (w), 1260 (m), 1227 (m), 1210 (m), 1107 (w), 951 (w), 867 (w), 821 (m), 781 (w), 734 (s), 700 (w).

***R_f* =** 0.39 (8:1 $CHCl_3$:acetone; visualized with UV and/or $Ce(SO_4)_2$ in phosphomolybdic acid stain).

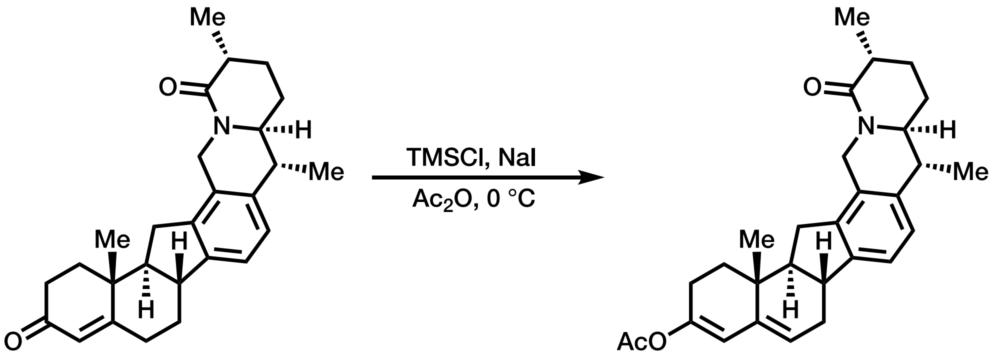

Dienol acetate 31. To a pre-cooled solution of enone **30** (29 mg, 0.072 mmol, 1 equiv) in acetic anhydride (0.90 mL) was added dropwise chlorotrimethylsilane (37 µL, 0.288 mmol, 4 equiv), followed by sodium iodide (43 mg, 0.288 mmol, 4 equiv) in a single portion at 0 °C. The resultant opaque yellow mixture was stirred at 0 °C for 4 h, then concentrated to dryness under a stream of dry N_2 and further via high vacuum. The yellow-orange residue was re-dissolved in a solution of 1:1 Et_2O:hexanes (10 mL) and washed successively with a 2% aqueous $Na_2S_2O_3$ solution (1 x 8 mL), saturated aqueous $NaHCO_3$ solution (1 x 8 mL), distilled water (1 x 10 mL), and brine (1 x 10 mL). The organic extracts were then dried over Na_2SO_4 and concentrated to afford dienol acetate **31** (32 mg, 100% yield) as light-yellow solid. The crude concentrate was typically clean enough to be carried forward without further purification, however flash column chromatography (30:1 $CHCl_3$:acetone) was used to obtain spectroscopically pure material.

^1H NMR (400 MHz, $CDCl_3$): δ 7.14 (d, *J* = 7.8 Hz, 1H), 7.06 (d, *J* = 7.8 Hz, 1H), 5.81 (s, 1H), 5.53 (s, 1H), 5.13 (d, *J* = 17.5 Hz, 1H), 4.23 (d, *J* = 17.5 Hz, 1H), 3.26 – 3.18 (td, *J* = 9.2, 5.5 Hz, 1H), 3.18 – 3.09 (td, *J* = 11.6, 5.6 Hz, 1H), 2.85 – 2.70 (m, 3H), 2.66 – 2.56 (m, 1H), 2.57 – 2.47 (m, 1H), 2.45 – 2.36 (m, 1H), 2.34 – 2.26 (m, 1H), 2.23 – 2.09 (m, 2H), 2.16 (s, 3H), 2.07 – 1.98 (m, 1H), 1.95 – 1.87 (td, *J* = 11.8, 7.2 Hz, 1H), 1.86 – 1.79 (dd, *J* = 12.6, 5.4 Hz, 1H), 1.72 – 1.59 (m, 1H), 1.59 – 1.44 (m, 2H), 1.35 (d, *J* = 6.7 Hz, 3H), 1.28 (d, *J* = 7.1 Hz, 3H), 1.14 (s, 3H).

^{13}C NMR (101 MHz, CDCl$_3$): δ 173.2, 169.5, 147.7, 144.8, 140.7, 140.2, 136.7, 129.3, 124.1, 123.9, 120.8, 116.9, 60.2, 55.5, 43.5, 40.6, 39.3, 36.8, 35.5, 35.0, 30.5, 29.2, 27.7, 27.0, 24.8, 21.3, 18.9, 17.9, 16.3.

COSY/HSQC: see **Figure S111** / **Figure S112**.

HRMS: (ESI) *m/z* [M+H]$^+$ calcd for $C_{29}H_{35}NO_3$: 446.2695, found: 446.2697.

$[\alpha]_D^{20.3}$ = +6.3 (*c* = 1.0, $CHCl_3$).

IR (thin film) ν_{max} (cm^{-1}): 2329 (s), 2853 (m), 1755 (s), 1639 (s), 1434 (m), 1365 (m), 1216 (s), 1195 (s), 1112 (m), 1015 (w), 934 (w), 810 (m), 735 (m).

***R_f* =** 0.70 (8:1 $CHCl_3$:acetone; visualized with UV and/or $Ce(SO_4)_2$ in phosphomolybdic acid stain).

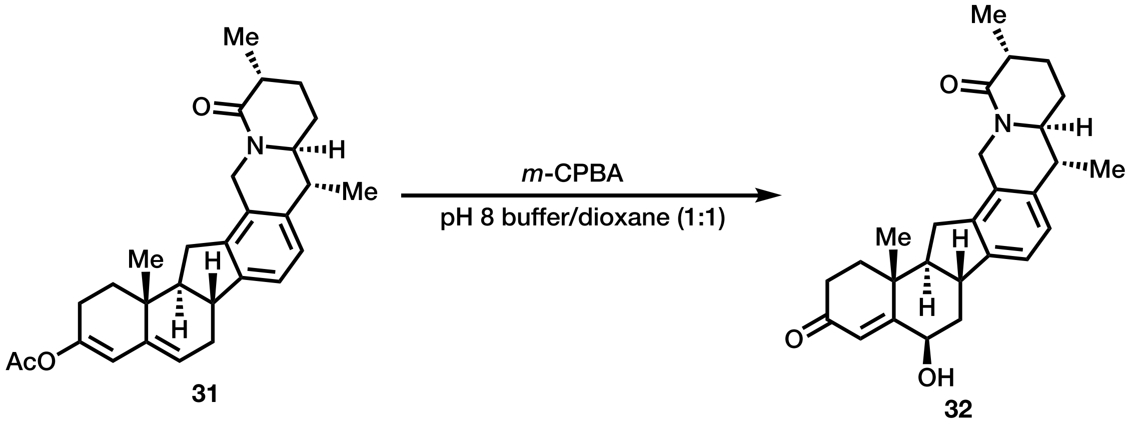

31 32

γ-Hydroxy enone 32. A solution of dienol acetate **31** (30 mg, 0.067 mmol, 1 equiv) in 1:1 1,4-dioxane: pH 8 buffer[21] (0.6 mL) was cooled to 0 °C and treated dropwise with a solution of *meta*-chloroperoxybenzoic acid (80% w/w; 18 mg, 0.081 mmol, 1.2 equiv) in 1:1 1,4-dioxane: pH 8 buffer (1.1 mL) over a period of 1 h. Following complete addition, the resultant mixture was brought to ambient temperature and stirred for 19 h. Upon consumption of the starting material as indicated by TLC, the reaction mixture was treated with solid $Na_2S_2O_3$ (15 mg) and solid $NaHCO_3$ (30 mg). The mixture was then stirred vigorously for 15 minutes and diluted with H_2O (6 mL) and $CHCl_3$ (8 mL). The biphasic mixture was separated, aqueous layer repeatedly extracted with $CHCl_3$ (6 x 6

[21] Prepared according to a modified protocol, as follows: To a solution of 0.1 M aqueous KH_2PO_4 was added a sufficient quantity of 0.1 M aqueous Na_2HPO_4 until the pH was approximately 8. See, Imuta, M.; Ziffer, H. Convenient method for the preparation of reactive oxiranes by direct epoxidation. *J. Org. Chem.* **1979**, *44*, 1351−1352.

mL), and the organic extracts were combined and dried over Na_2SO_4. Concentration of the dried extracts provided a crude tan solid, which was purified via flash column chromatography (4:1 $CHCl_3$:acetone) to afford γ-hydroxy enone **32** (22 mg, 78% yield) as an off-white solid.

^{1}H NMR (500 MHz, $CDCl_3$): δ 7.15 (d, *J* = 7.8 Hz, 1H), 7.06 (d, *J* = 7.8 Hz, 1H), 5.91 (s, 1H), 5.12 (d, *J* = 17.4 Hz, 1H), 4.54 (app s, 1H), 4.21 (d, *J* = 17.4 Hz, 1H), 3.54 (m, 1H), 3.21 (td, *J* = 9.2, 5.7 Hz, 1H), 2.84 – 2.76 (m, 1H), 2.75 – 2.69 (dd, *J* = 14.8, 7.1 Hz, 1H), 2.68 – 2.57 (m, 3H), 2.48 – 2.42 (m, 1H), 2.42 – 2.36 (m, 1H), 2.34 – 2.26 (m, 1H), 2.20 (br s, 1H), 2.06 – 1.95 (m, 2H), 1.93 – 1.84 (m, 1H), 1.83 – 1.75 (td, *J* = 11.8, 7.1 Hz, 1H), 1.74 – 1.63 (m, 2H), 1.54 – 1.45 (m, 1H), 1.49 (s, 3H), 1.34 (d, *J* = 6.8 Hz, 3H), 1.28 (d, *J* = 7.1 Hz, 3H).

^{13}C NMR (101 MHz, $CDCl_3$): δ 200.4, 173.3, 167.6, 143.7, 139.8, 137.0, 129.4, 127.7, 124.2, 120.7, 77.4, 74.2, 60.2, 59.9, 43.5, 39.3, 38.3, 38.2, 37.6, 36.8, 34.2, 28.9, 27.6, 26.9, 18.9, 17.9, 16.4.

COSY/HSQC: see **Figure S115** / **Figure S116**.

HRMS: (ESI) *m*/*z* $[M+H]^+$ calcd for $C_{27}H_{33}NO_3$: 420.2538, found: 420.2536.

$[\alpha]_D^{21.4}$ = +136.8 (*c* = 1.0, $CHCl_3$).

IR (thin film) ν_{max} (cm^{-1}): 3381 (br), 2929 (s), 2869 (m), 1674 (s), 1618 (s), 1464 (m), 1442 (s), 1378 (w), 1326 (w), 1261 (m), 1226 (m), 1210 (w), 1180 (w), 1100 (w), 1050 (m), 1010 (m), 954 (w), 931 (w), 877 (w), 829 (w), 810 (w), 733 (s), 700 (m), 641 (w).

***R_f* =** 0.31 (3:1 $CHCl_3$:acetone; visualized with UV and/or $Ce(SO_4)_2$ in phosphomolybdic acid stain).

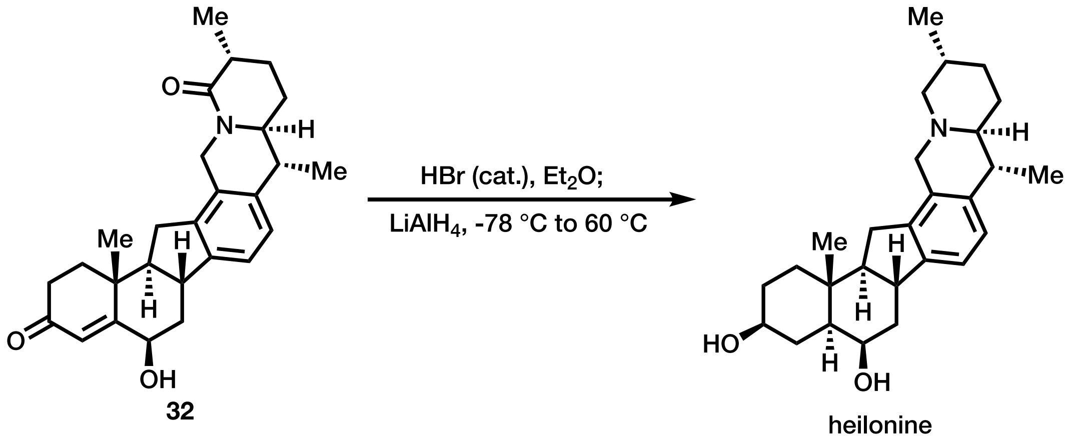

Heilonine. To a solution of γ-hydroxy enone **32** (8.7 mg, 0.021 mmol, 1 equiv) in Et_2O (1.2 mL) was added hydrobromic acid (48 wt.% in H_2O; 0.4 mg, 2.1 μmol, ca. 0.1 equiv) at ambient temperature. The resultant mixture was stirred at ambient temperature for 6 h. Upon complete consumption of starting material as indicated by TLC,[22] the reaction mixture was cooled to −78 °C and treated with a 1.05 M solution of $LiAlH_4$ in THF (0.06 mL, 0.063 mmol, 3 equiv) dropwise at −78 °C. The resultant mixture was stirred at −78 °C for 0.5 h, then brought to ambient temperature. The opaque mixture was stirred for 1 h, then treated with additional 1.05 M solution of $LiAlH_4$ in THF (0.08 mL, 0.084 mmol, 4 equiv) dropwise at ambient temperature. The resultant mixture was heated to 60 °C, whereupon the solution became beige and clear, and was stirred at the same temperature for 18 h. The reaction mixture was brought to ambient temperature, then cooled further to 0 °C and diluted with Et_2O (1.5 mL). The reaction was carefully quenched by sequential addition of H_2O (20 μL), 2 M NaOH aqueous solution (20 μL), additional H_2O (50 μL), and vigorously stirred for 15 minutes upon warming to ambient temperature. Anhydrous $MgSO_4$ was then added and the mixture was stirred for an additional 15 minutes. The mixture was filtered through a layer of Celite, which was subsequently washed thoroughly with Et_2O (ca. 25 mL). Concentration of the filtrate provided a crude whitish residue, which was purified via preparative thin-layer chromatography (88: 11: 1 $CHCl_3$: MeOH: aq. NH_4OH) to afford heilonine (6.1 mg, 72% yield) as a white solid.[23]

^{1}H NMR (500 MHz, CD_3OD): δ 7.06 (d, *J* = 7.9 Hz, 1H), 6.96 (d, *J* = 7.9 Hz, 1H), 3.93 (m, 1H), 3.72 (d, *J* = 15.0 Hz, 1H), 3.64 – 3.56 (m, 1H), 3.28 (d, *J* = 15.0 Hz, 1H), 3.24 –

[22] The data for the intermediate γ-diketone (**SI-15**) is as follows: R_f = 0.60 (3:1 $CHCl_3$:acetone; visualized with $Ce(SO_4)_2$ in phosphomolybdic acid and/or $KMnO_4$ stain); see **Figure S117** for the ^{1}H NMR spectrum of an unpurified sample.

[23] In accordance with the isolation paper (see Note 24 for reference), heilonine was found to have only modest solubility at best in organic solvents. Spectral characterization of the natural product itself was not reported by the isolation team. We found CD_3OD to be a suitable NMR solvent, whereas $CDCl_3$ led to significant peak broadening despite apparent solubilization (see **Figures S118** and **S122**, respectively, for ^{1}H NMR spectra). Solutions containing the natural product appeared to be surprisingly hygroscopic, a property that can be appreciated by the relatively large HDO peak in the NMR spectrum.

3.16 (m, 1H), 3.06 – 2.99 (m, 1H), 2.75 – 2.67 (m, 1H), 2.61 – 2.55 (dd, J = 14.5, 6.8 Hz, 1H), 2.51 – 2.44 (m, 1H), 2.43 – 2.38 (dt, J = 13.1, 3.1 Hz, 1H), 2.21 – 2.15 (dq, J = 13.5, 3.0 Hz, 1H), 1.92 – 1.75 (m, 5H), 1.75 – 1.64 (m, 2H), 1.63 – 1.53 (m, 3H), 1.53 – 1.46 (m, 1H), 1.37 – 1.30 (m, 1H), 1.27 (d, J = 6.9 Hz, 3H), 1.24 – 1.18 (m, 2H), 1.16 (s, 3H), 1.10 – 1.01 (m, 1H), 0.94 (d, J = 6.5 Hz, 3H).

^{13}C NMR (101 MHz, CD_3OD): δ 145.4, 140.6, 137.4, 130.1, 126.2, 121.5, 73.4, 72.7, 67.2, 64.9, 62.8, 57.0, 49.9, 40.1, 40.0, 40.0, 38.43, 36.8, 35.7, 33.9, 32.6, 31.9, 31.7, 29.6, 20.4, 19.8, 15.9.

COSY/HSQC: see **Figure S120** / **Figure S121**.

HRMS: (ESI) *m/z* $[M+H]^+$ calcd for $C_{27}H_{39}NO_2$: 410.3059, found: 410.3055.

$[\alpha]_D^{23.8}$ = +64.3 (c = 1.0, CH_3OH).

IR (thin film) ν_{max} (cm^{-1}): 3382 (br), 2924 (m), 1652 (w), 1558 (w), 1456 (m), 1275 (s), 1261 (s), 1032 (w), 764 (s), 750 (s), 668 (m).

***R_f* =** 0.34 (13% MeOH in $CHCl_3$; visualized with $KMnO_4$ stain).

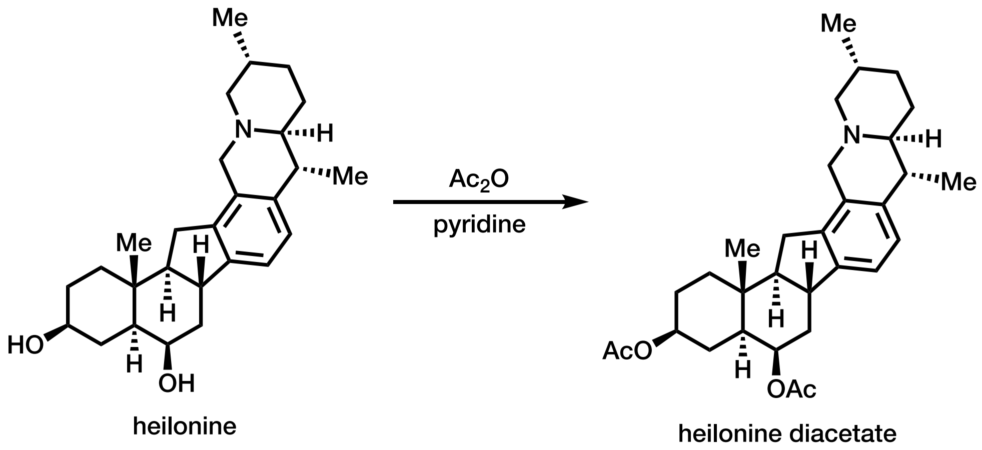

Heilonine diacetate. To a solution of heilonine (13.4 mg, 0.033 mmol, 1 equiv) in acetic anhydride (1.2 mL) was added pyridine (1.2 mL) at ambient temperature. The resultant mixture was heated to 60 °C and stirred for 6 h, then brought back to ambient temperature and stirred for an additional 18 h. Upon consumption of the starting material as indicated by TLC, the mixture was concentrated under a stream of dry N_2 and dried further under high vacuum (2 h at 1 mm Hg) to afford a crude off-white solid, which was purified via preparative thin-layer chromatography (95.5: 5.0: 0.5 $CHCl_3$: MeOH: aq. NH_4OH) to afford heilonine diacetate (12.4 mg, 77% yield) as a white solid.

^{1}H NMR (500 MHz, $CDCl_3$): δ 7.06 (d, *J* = 7.8 Hz, 1H), 6.93 (d, *J* = 7.8 Hz, 1H), 5.11 (m, 1H), 4.78 (m, 1H), 3.71 (d, *J* = 14.6, 1H), 3.24 (d, *J* = 14.6, 1H), 3.09 (td, *J* = 12.3, 2.7 Hz, 1H), 3.02 (br s, 1H), 2.72 (br s, 1H), 2.62 – 2.55 (dd, *J* = 14.4, 6.8 Hz, 1H), 2.50 – 2.43 (m, 2H), 2.16 – 2.09 (m, 1H), 2.07 (s, 3H), 2.04 (s, 3H), 1.92 – 1.77 (m, 4H), 1.77 – 1.71 (m, 1H), 1.70 – 1.61 (m, 4H), 1.61 – 1.53 (td, *J* = 13.6, 3.1 Hz, 1H), 1.46 – 1.39 (td, *J* = 12.9, 2.4 Hz, 1H), 1.37 – 1.30 (m, 1H), 1.27 (d, *J* = 6.9 Hz, 3H), 1.26 – 1.20 (m, 1H), 1.15 (s, 3H), 1.05 – 0.97 (m, 1H), 0.92 (d, *J* = 5.4 Hz, 3H); lit.: see **Table S1** for comparison.

^{13}C NMR (125 MHz, $CDCl_3$): δ 170.6, 170.6, 142.9, 139.1, 137.2, 130.1, 125.4, 120.3, 74.2, 73.6, 65.8, 64.5, 60.7, 56.4, 47.3, 39.2, 39.2, 38.6, 35.9, 34.3, 33.2, 32.1, 30.9, 30.5, 28.8, 27.2, 21.5, 21.5, 19.8, 19.8, 15.1; lit.: see **Table S2** for comparison.

COSY/HSQC: see **Figure S125** / **Figure S126**.

HRMS: (ESI) *m/z* $[M+H]^+$ calcd for $C_{31}H_{43}NO_4$: 494.3270, found: 494.3255.

$[\alpha]_D^{21.1}$ = +29.8 (*c* = 1.05, $CHCl_3$); lit.: +34 (*c* = 1.05, $CHCl_3$).

IR (thin film) ν_{max} (cm^{-1}): 2929 (s), 2853 (m), 1734 (s), 1458 (m), 1375 (m), 1240 (m), 1139 (w), 1098 (w), 1077 (w), 1025 (s), 956 (w), 829 (w), 809 (w), 750 (w), 606 (w); lit.: ν_{max} ($CHCl_3$) 2850, 1710 cm^{-1} .

***R_f* =** 0.46 (9% MeOH in $CHCl_3$; visualized with $KMnO_4$ and/or $Ce(SO_4)_2$ in phosphomolybdic acid stain).

Miscellaneous Information:

Scheme S6: Analysis of stereoselectivity in the asymmetric Diels-Alder reaction.

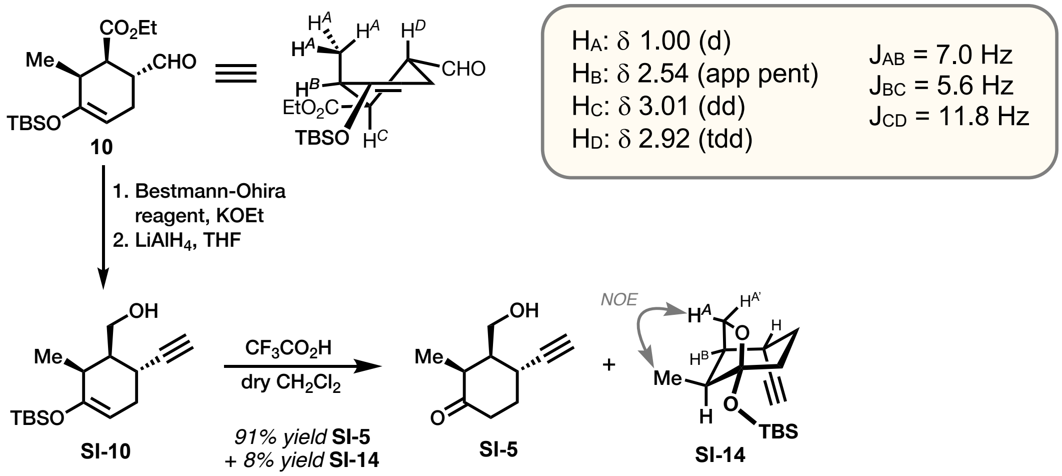

Scheme S7: Condensed summary of the optimization for the key [2+2+2] cycloaddition.

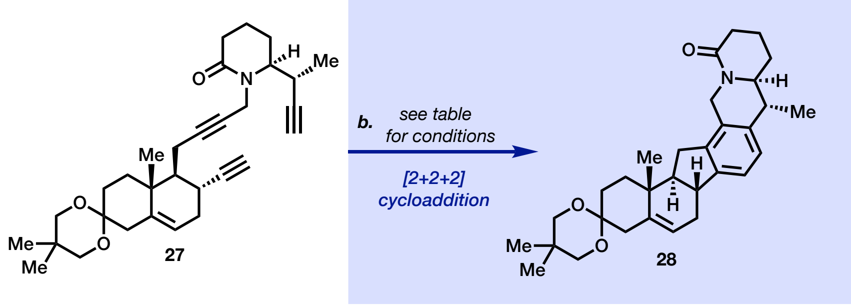

Entry	*Conditions*	*Result*
1.	$RhCl(PPh_3)_3$ (5 mol%)<br>CH_2Cl_2, RT	*18% yield*
2.	$RhCl(PPh_3)_3$ (5 mol%)<br>DCE, 80 °C	*43% yield*
3.	$RhCl(PPh_3)_3$ (5 mol%)<br>EtOH, 80 °C	*57% yield*
4.	**$RhCl(PPh_3)_3$ (10 mol%)<br>EtOH, 80 °C**	***89% yield***
5.	Cp*Ru(cod)Cl (20 mol%)<br>DCE, 80 °C	*67% yield*

Table S1. ^{1}H NMR (500 MHz, $CDCl_3$) spectral data comparison of synthetic heilonine diacetate (**Figure S123**) and natural heilonine diacetate.[24]

Synthetic	Natural [24,25]	Δδ, ppm [26]
7.06 (d, *J* = 7.8 Hz, 1H)	7.08 (d, *J* = 7.8 Hz, 1H)	0.02 (0.00)
6.93 (d, *J* = 7.8 Hz, 1H)	6.96 (d, *J* = 7.8 Hz, 1H)	0.03 (0.01)
5.11 (m, $W_{1/2}$ = 7.5 Hz, 1H)	5.10 (m, $W_{1/2}$ = 8 Hz, 1H)	0.01 (0.00)
4.78 (sept, *J* = 5.3 Hz, 1H)	4.78 (sept, *J* = 5 Hz, 1H)	0.00 (0.00)
3.71 (d, *J* = 14.6 Hz, 1H)	3.92 (d, *J* = 15.0 Hz, 1H)	0.21 (0.00)
3.24 (d, *J* = 14.6 Hz, 1H)	3.32 (d, *J* = 15.0 Hz, 1H)	0.08 (0.00)
3.02 (br s, 1H)	3.20 (br d, *J* = 10 Hz, 1H)	0.18 (0.00)
3.09 (td, 12.3, 2.7 Hz, 1H)	3.12 (dt, *J* = 12.2, 7 Hz, 1H)	0.03 (0.00)
2.72 (br s, 1H)	2.84 (pent, *J* = 7 Hz, 1H)	0.12 (−0.01)
2.58 (dd, 14.4, 6.8 Hz, 1H)	2.57 (dd, *J* = 14.0, 3.5 Hz, 1H)	−0.01 (0.00)
2.50 – 2.43 (m, 2H)	2.50 (br t, *J* = 13.6 Hz, 1H)	
	2.47 (dd, *J* = 14.0, 3.5 Hz, 1H)	
2.13 (m, 1H)	2.15 (br dd, *J* = 13.7, 2.7 Hz, 1H)	0.02 (0.00)
2.07 (s, 3H)	2.06 (s, 3H)	−0.01 (0.00)
2.04 (s, 3H)	2.04 (s, 3H)	0.00
1.92 – 1.77 (m, 4H)	1.99 (m, 1H)	
	1.89 (m, 1H)	
	1.88 (m, 1H)	
	1.87 (m, 1H)	
1.75 (m, 1H)	1.76 (m, 1H)	0.01
1.70 – 1.61 (m, 4H)	1.65 (m, 1H)	
	1.65 (m, 1H)	
	1.65 (m, 1H)	
	1.62 (m, 1H)	
1.57 (td, *J* = 13.6, 3.1 Hz, 1H)	1.57 (dt, *J* = 13.8, 3.0 Hz, 1H)	0.00
1.34 (m, 1H)	1.47 (m, 1H)	0.13 (0.01)
1.43 (td, *J* = 12.9, 2.4 Hz)	1.41 (m 1H)	−0.02
1.27 (d, *J* = 6.9 Hz, 3H)	1.29 (d, *J* = 6.9 Hz, 3H)	0.02 (0.00)
1.23 (m, 1H)	1.23 (m, 1H)	0.00
1.15 (s, 3H)	1.16 (s, 3H)	0.01 (0.01)
1.01 (m, 1H)	1.04 (qd, *J* = 11.8, 4 Hz, 1H)	0.03 (0.00)
0.92 (d, *J* = 5.4 Hz, 3H)	0.93 (d, *J* = 5.4 Hz, 3H)	0.01 (0.00)

[24] Kitamura, Y.; Nishizawa, M.; Kaneko, K, Shiro, M.; Chen, Y.; Hsu, H. New Steroidal Alkaloids from *Fritillaria Ussuriensis* Maxim. Pingbeinone and Heilonine. *Tetrahedron*, **1989**, *45*, 7281−7286.
[25] At 270 MHz in $CDCl_3$. The discrepancy in chemical shifts is most pronounced in the proximity of the tertiary amine moiety. Since the ^{1}H NMR of synthetic, *unpurified* material matches well with the isolation data we suspect that some residual acetic acid may be present and is the cause of the discrepancy in spectra of the purified material (see **Figure S127**).
[26] The parenthesized values refer to the difference in chemical shift (ppm) of unpurified material.

Table S2. ^{13}C NMR (125 MHz, $CDCl_3$) spectral data comparison of synthetic heilonine diacetate (**Figure S124**) and natural heilonine diacetate.[24]

Synthetic	Natural [24,27]	Δδ, ppm	Carbon No.
170.6	170.5	−0.1	28
170.6	170.5	−0.1	30
142.9	142.8	−0.1	12
139.1	139.0	−0.1	14
137.2	136.7	−0.5	13
130.1	129.5	−0.6	17
125.4	125.2	−0.2	16
120.3	120.2	−0.1	15
74.2	74.0	−0.2	6
73.6	73.4	−0.2	3
65.8	65.5	−0.3	22
64.5	64.0	−0.5	26
60.7	60.5	−0.2	9
56.4	55.8	−0.6	18
47.3	47.1	−0.2	5
39.2	39.0	−0.2	8
39.2	38.7	−0.5	20
38.6	38.4	−0.2	1
35.9	35.7	−0.2	10
34.3	34.2	−0.1	7
33.2	32.9	−0.3	24
32.1	31.5	−0.6	23
30.9	30.5	−0.4	25
30.5	30.4	−0.1	4
28.8	28.6	−0.2	11
27.2	27.1	−0.1	2
21.5	21.34	−0.2	31
21.5	21.27	−0.2	29
19.8	19.57	−0.2	27
19.8	19.49	−0.3	21
15.1	14.9	−0.2	19

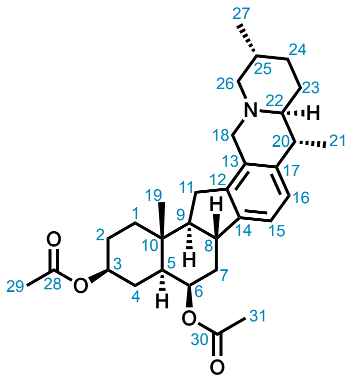

[27] At 100 MHz in $CDCl_3$.

Experimental Spectra:

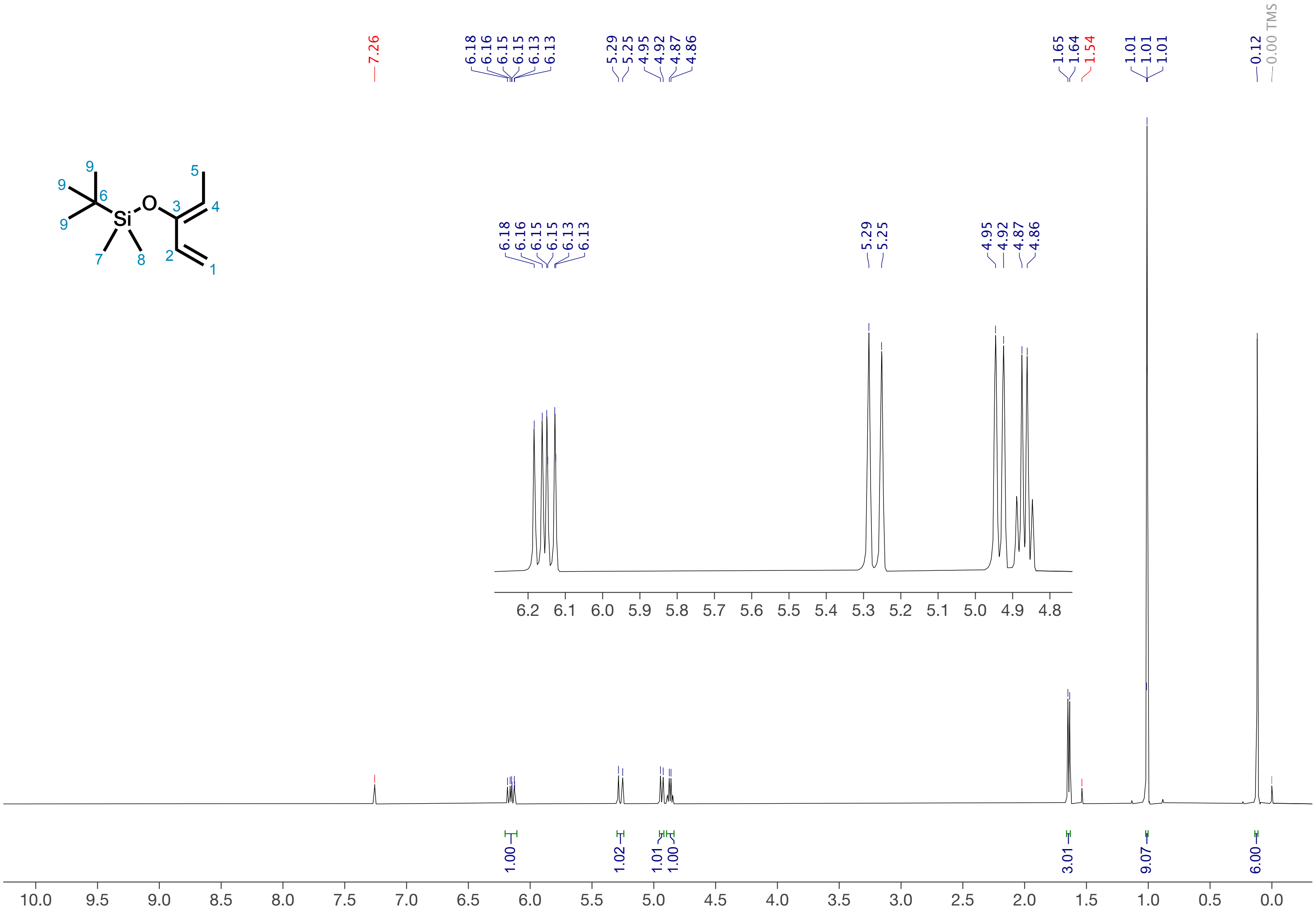

Figure S1. ^{1}H NMR (500 MHz, $CDCl_3$) of **siloxydiene 8**.

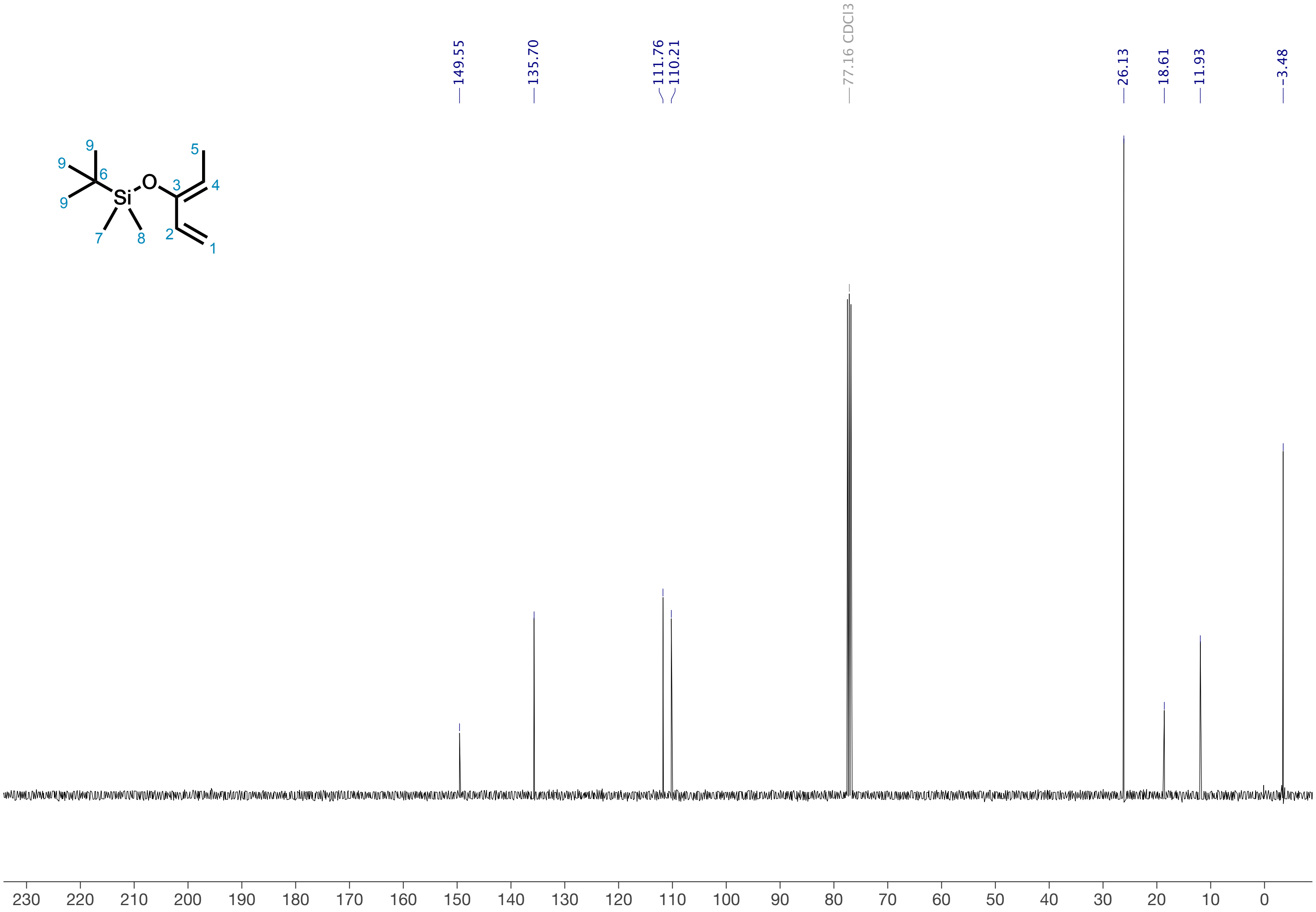

Figure S2. ^{13}C NMR (101 MHz, $CDCl_3$) of **siloxydiene 8**.

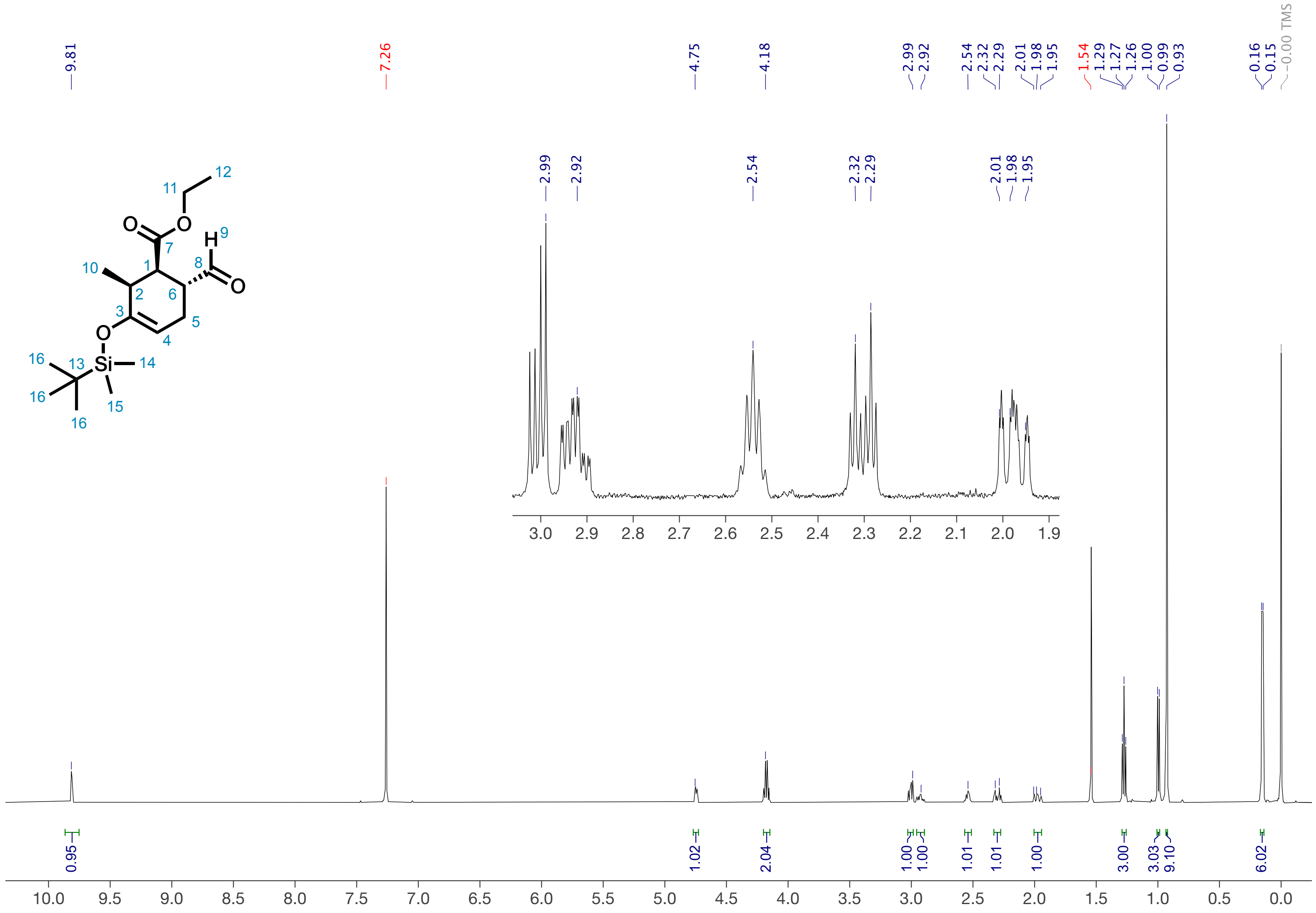

Figure S3. ^{1}H NMR (400 MHz, $CDCl_3$) of **TBS enol ether 10**.

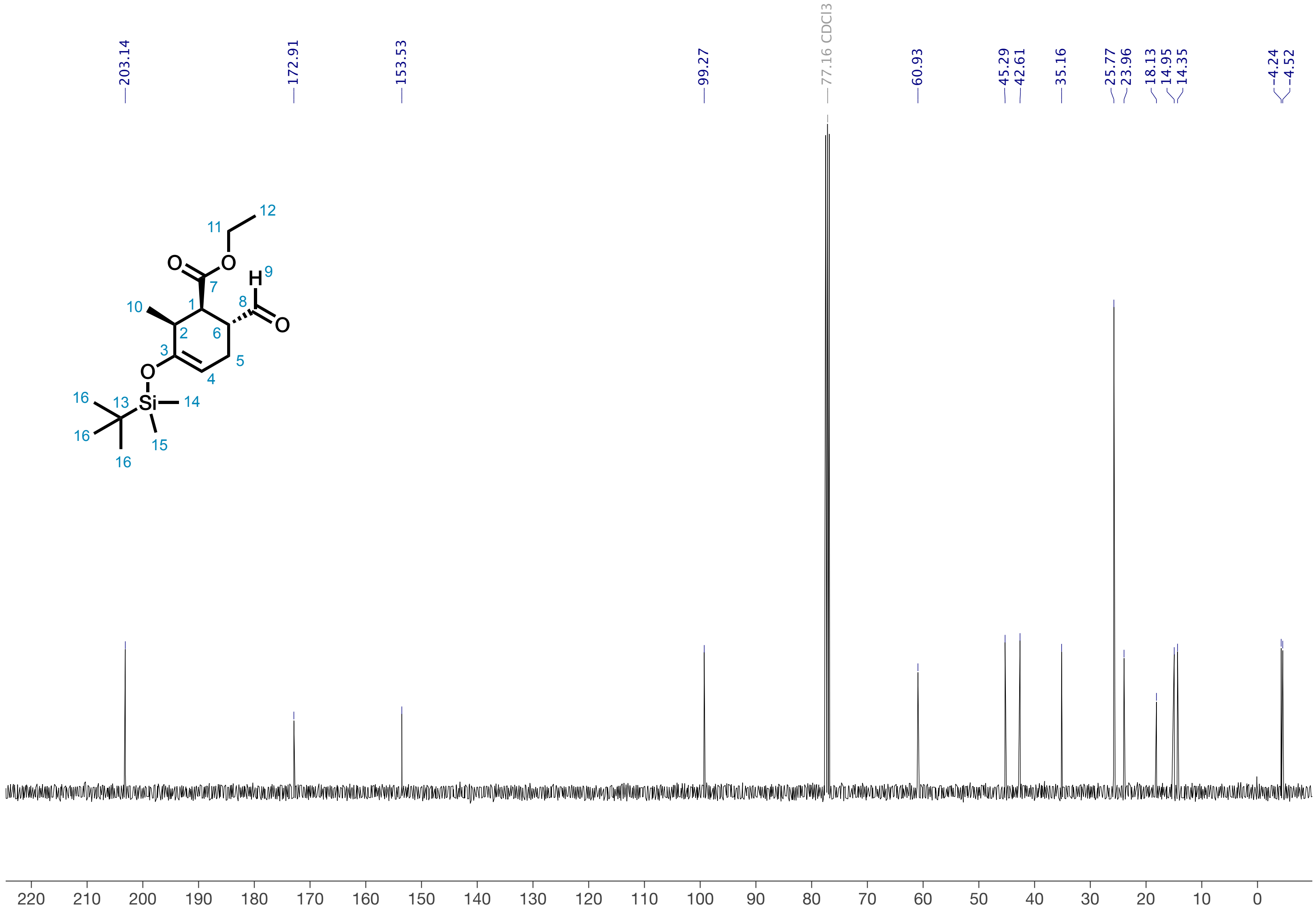

Figure S4. ^{13}C NMR (101 MHz, $CDCl_3$) of **TBS enol ether 10**.

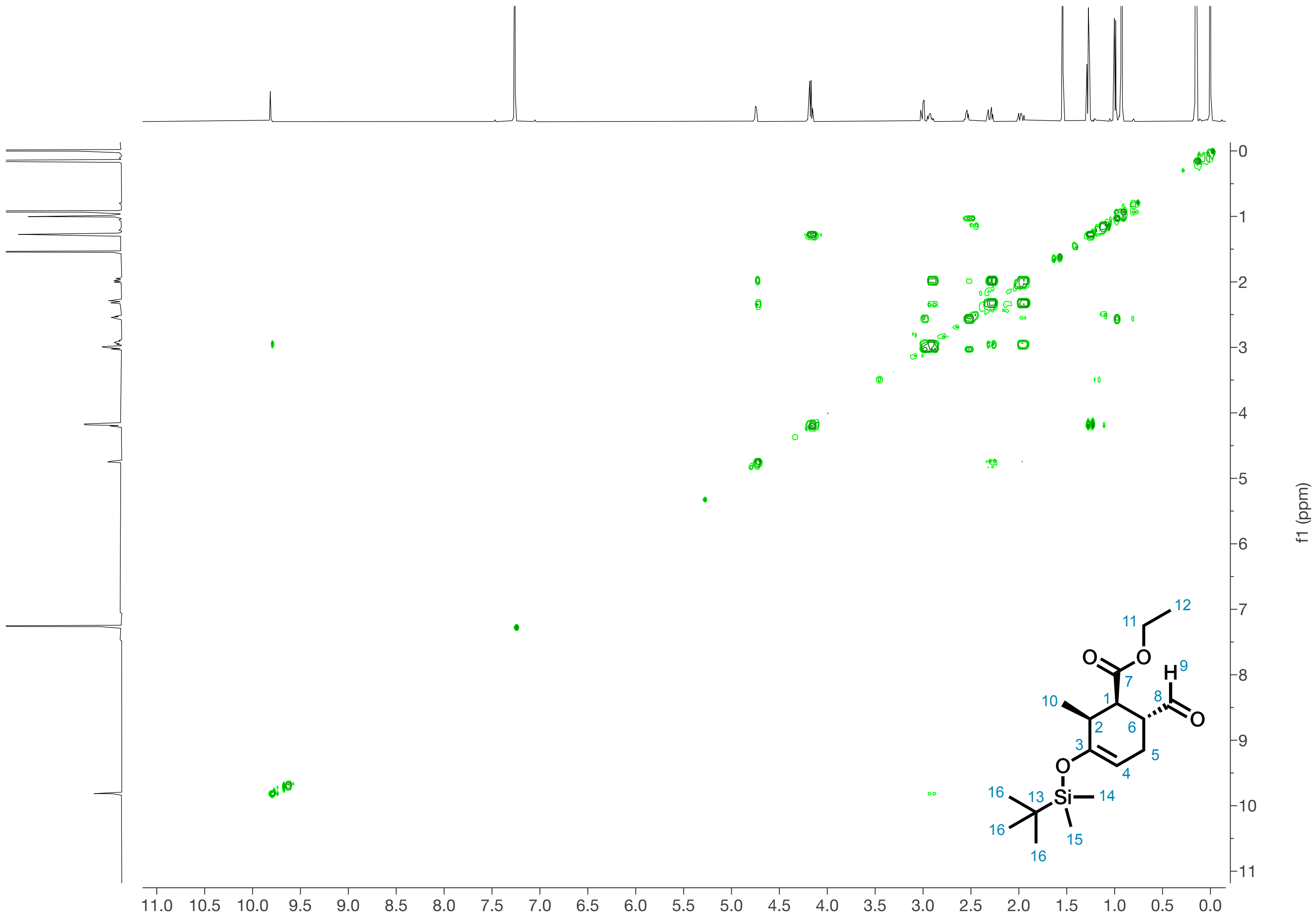

Figure S5. ^{1}H–^{1}H COSY (400 MHz, $CDCl_3$) of **TBS enol ether 10**.

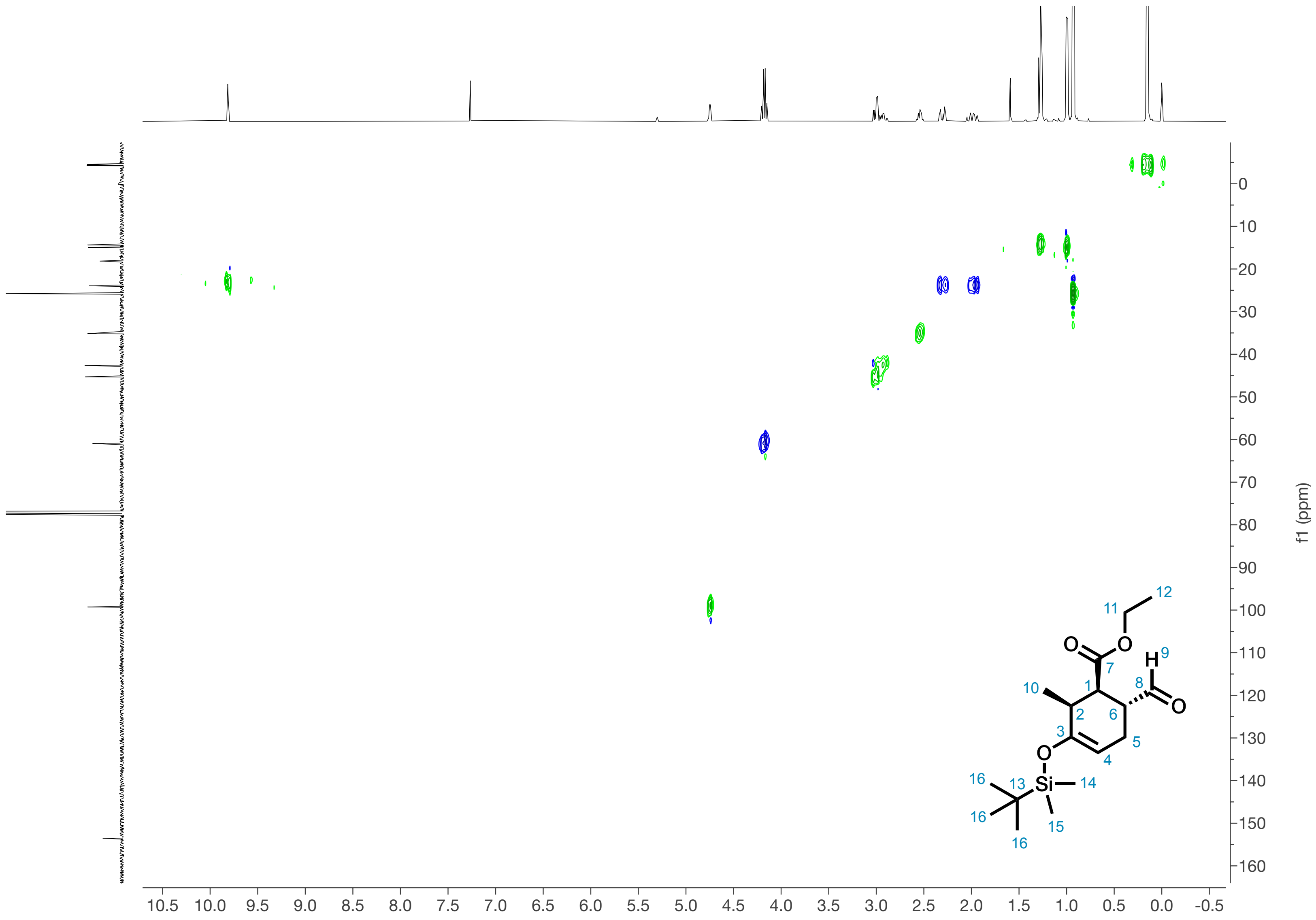

Figure S6. HSQC (400 MHz, $CDCl_3$) of **TBS enol ether 10**.

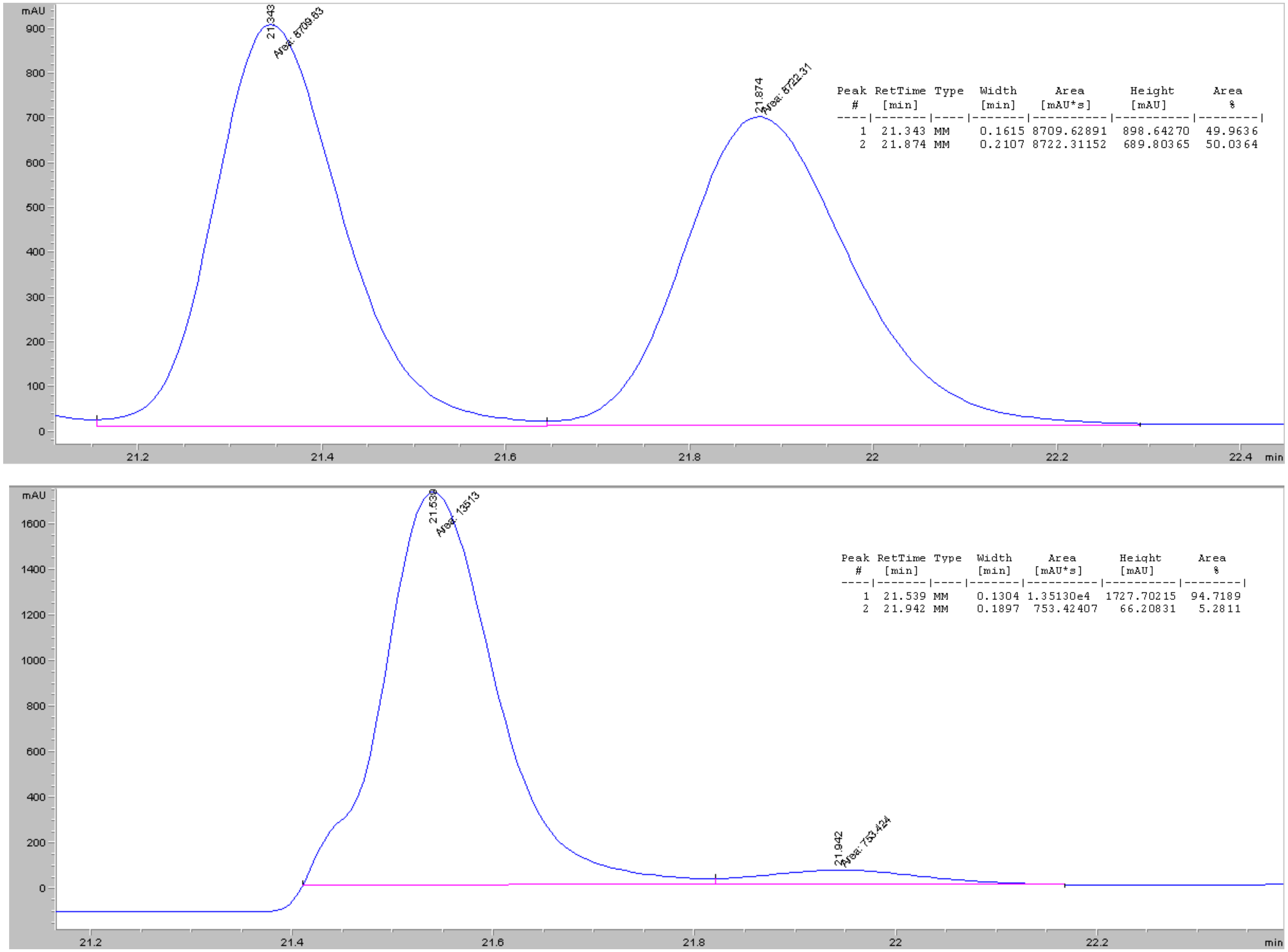

Figure S7. HPLC chromatogram of **(±)-10** (top) and **(–)-10** (bottom).

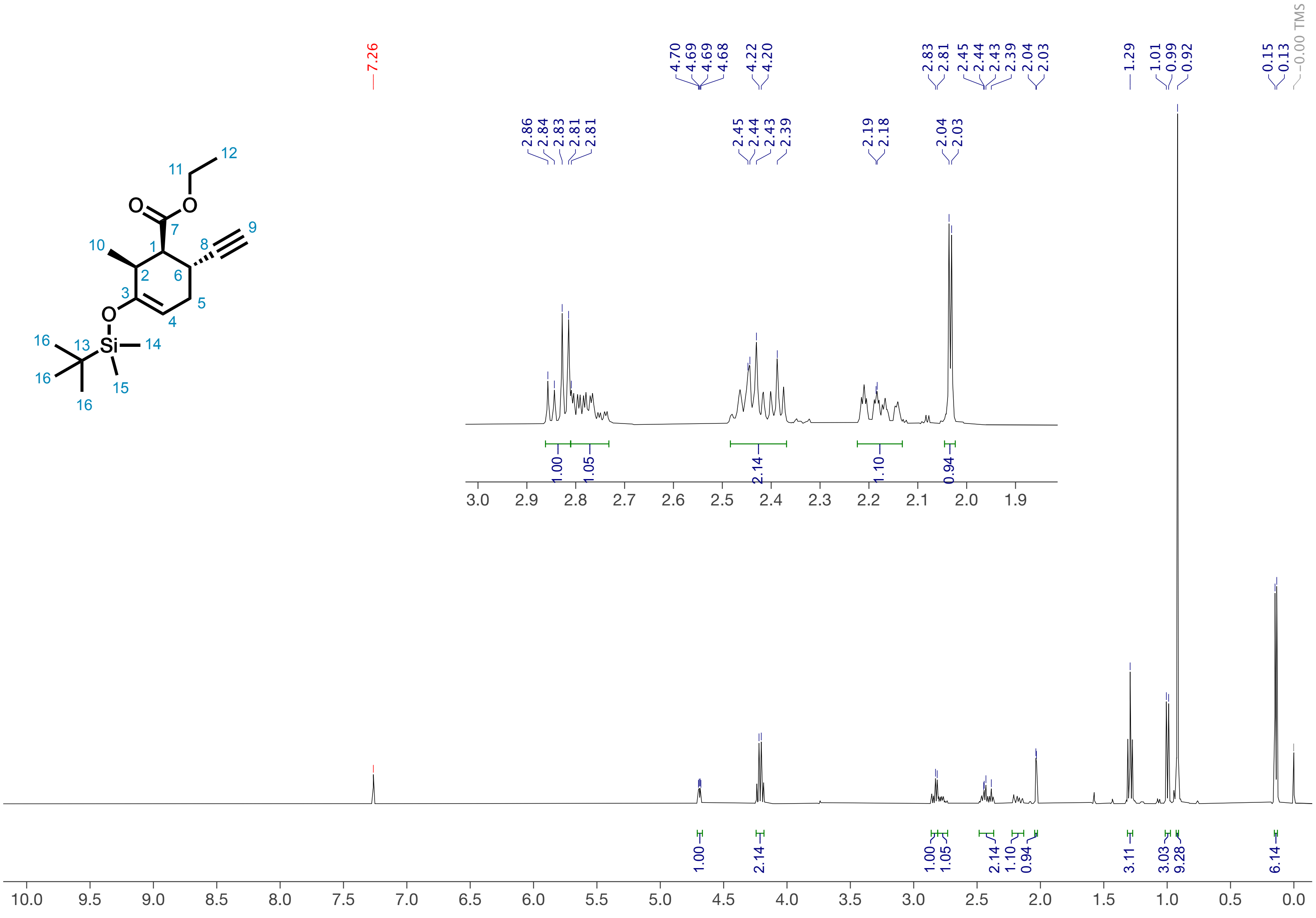

Figure S8. ^{1}H NMR (400 MHz, $CDCl_3$) of **alkyne 11**.

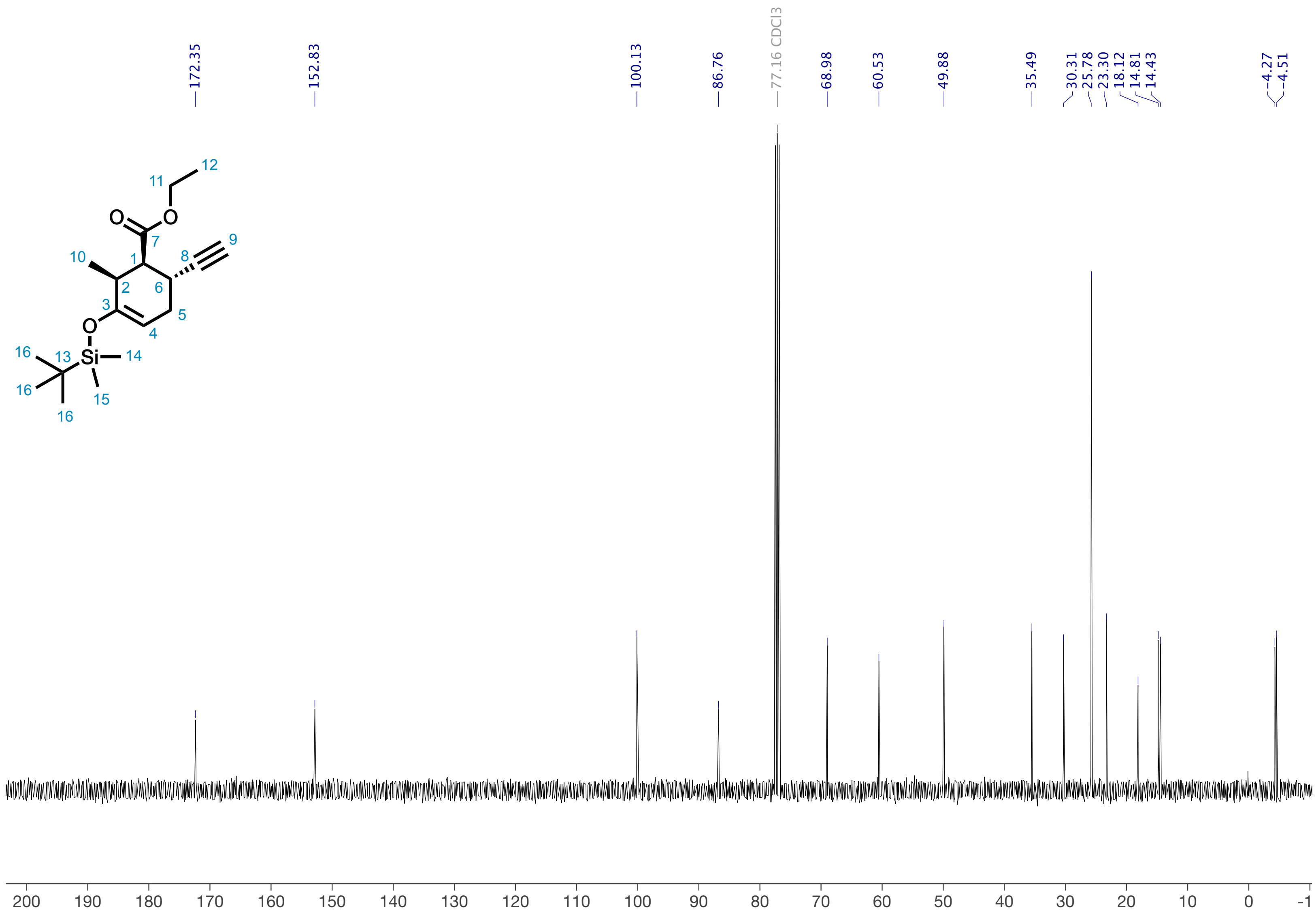

Figure S9. ^{13}C NMR (101 MHz, $CDCl_3$) of **alkyne 11**.

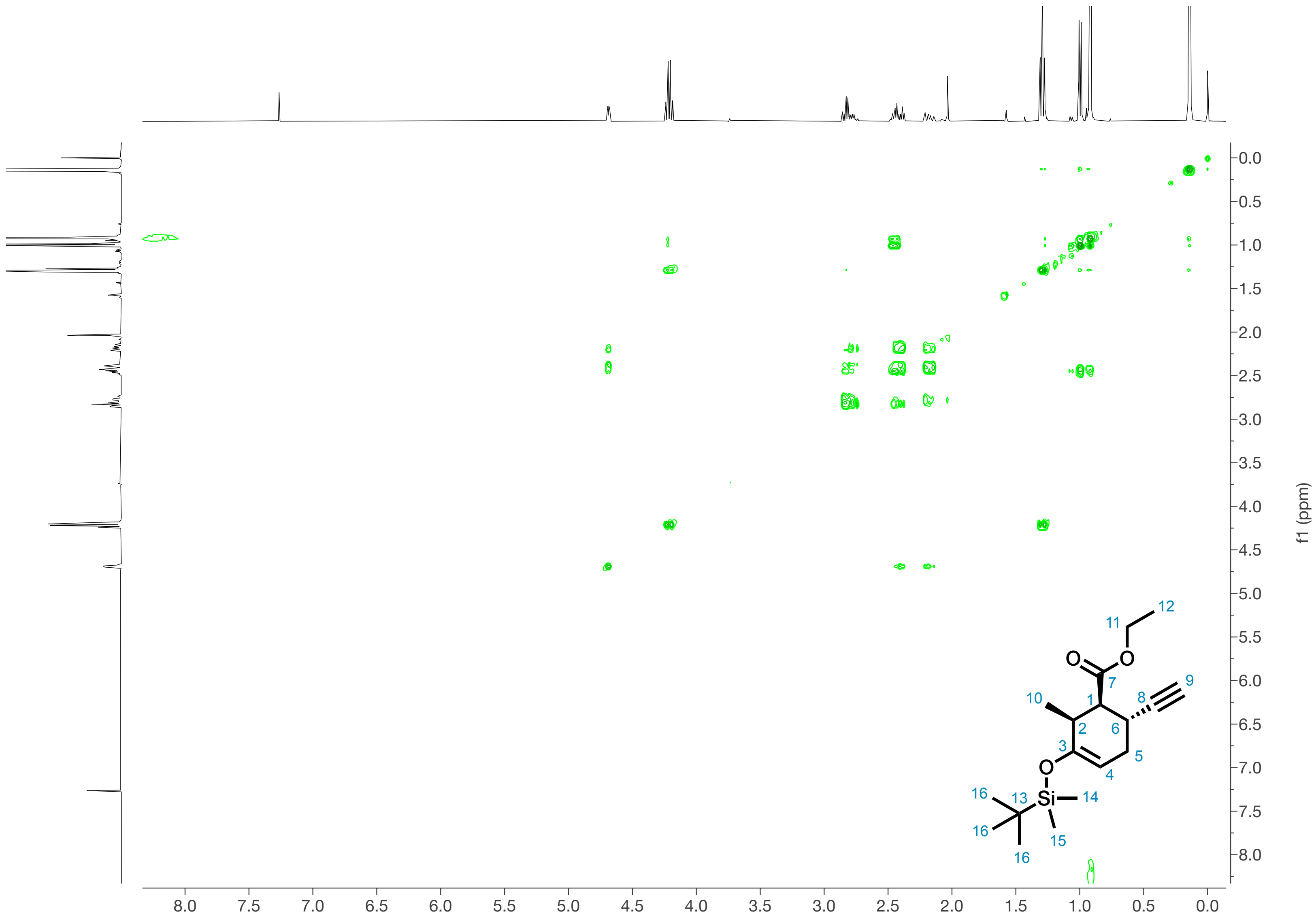

Figure S10. ^{1}H–^{1}H COSY (400 MHz, $CDCl_3$) of **alkyne 11**.

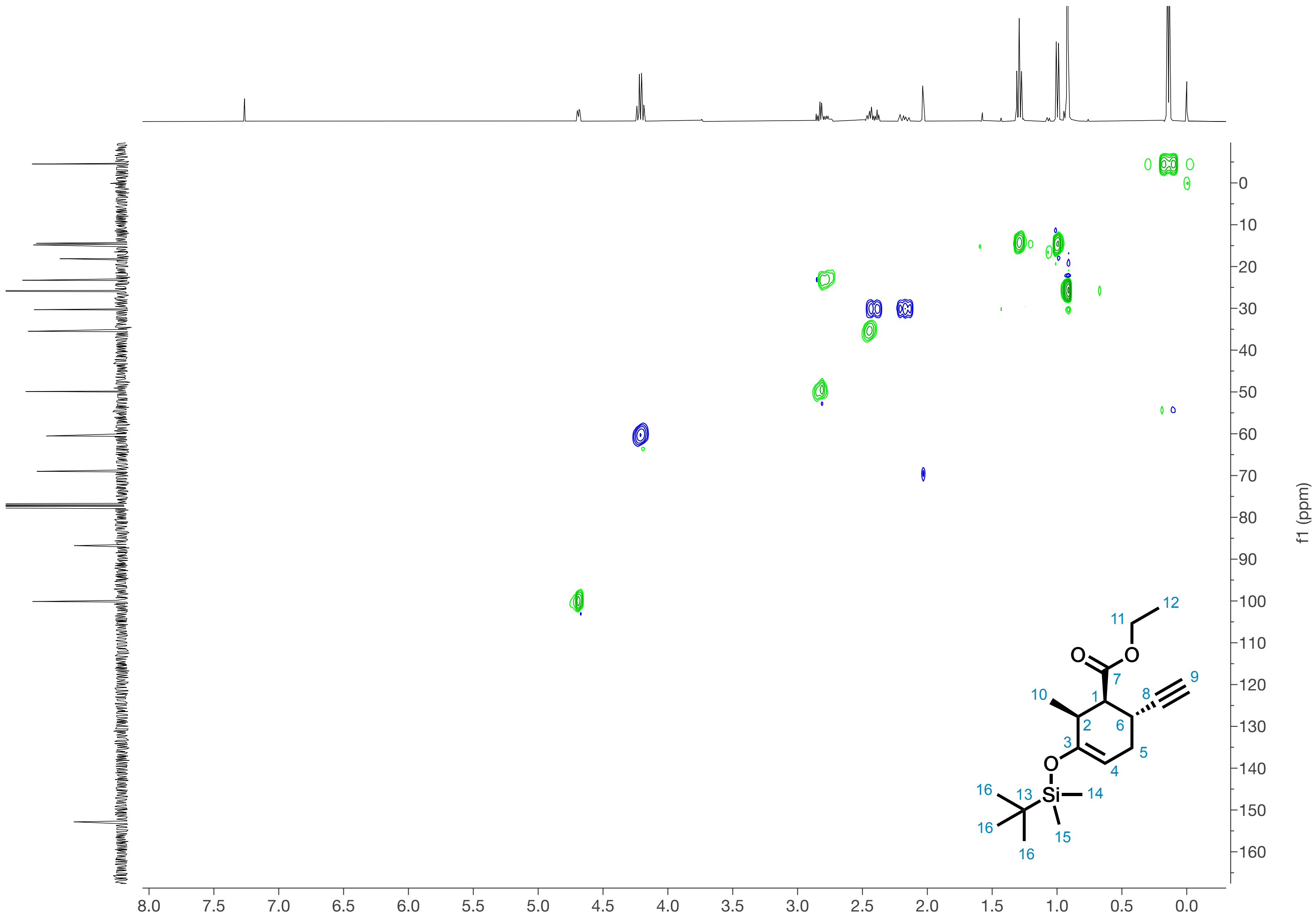

Figure S11. HSQC (400 MHz, $CDCl_3$) of **alkyne 11**.

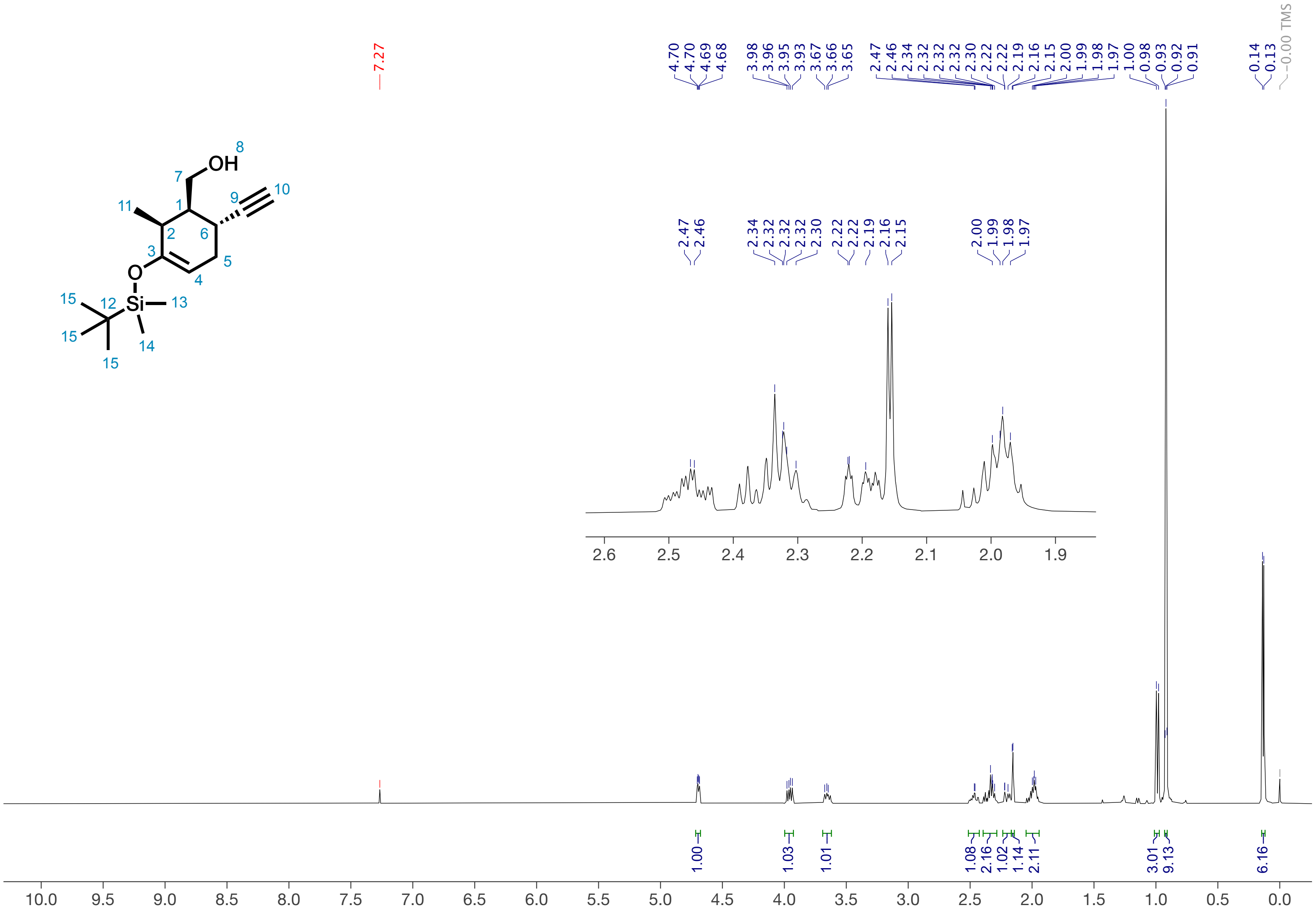

Figure S12. ^{1}H NMR (400 MHz, $CDCl_3$) of **primary alcohol SI-10**.

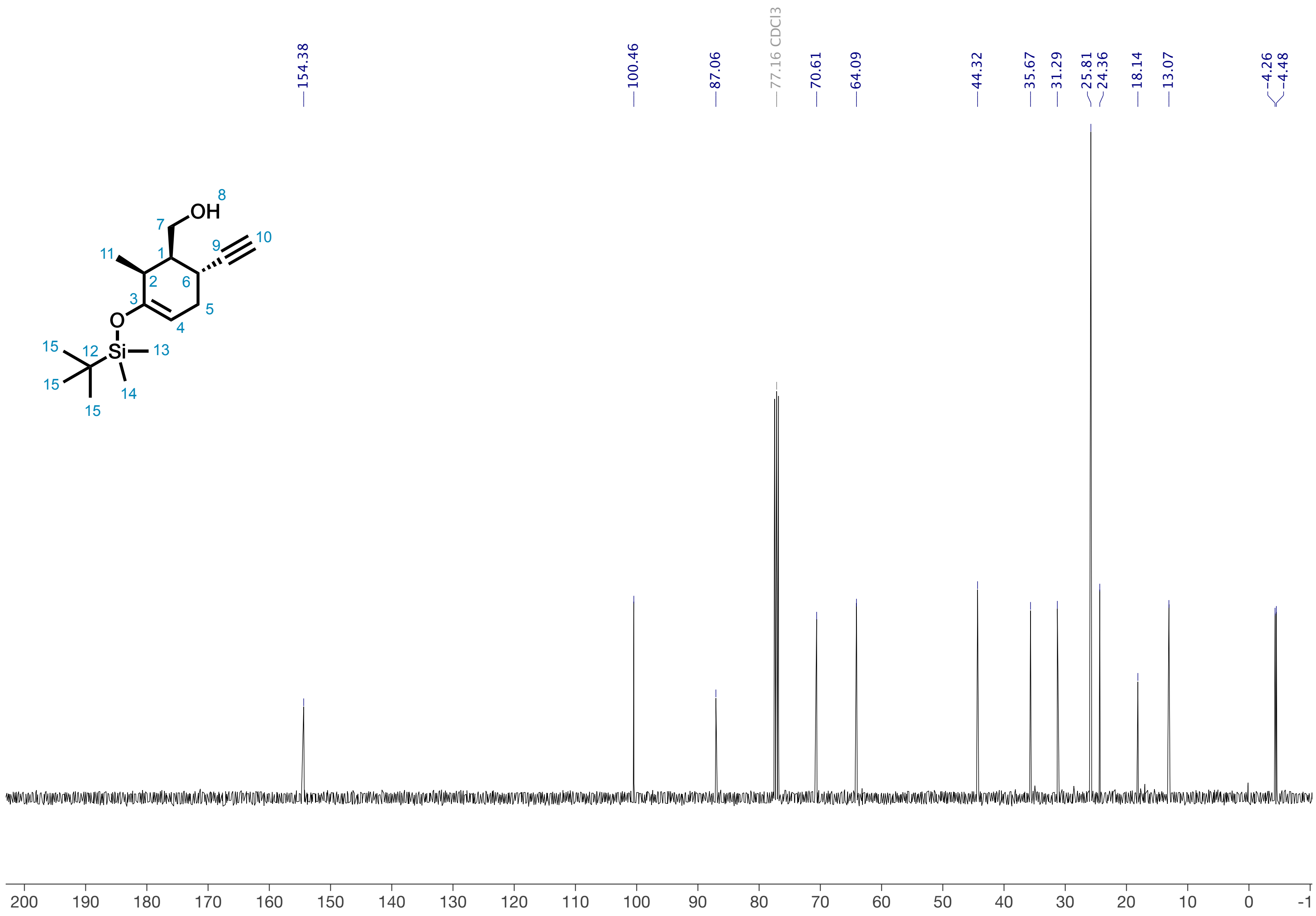

Figure S13. ^{13}C NMR (101 MHz, $CDCl_3$) of **primary alcohol SI-10**.

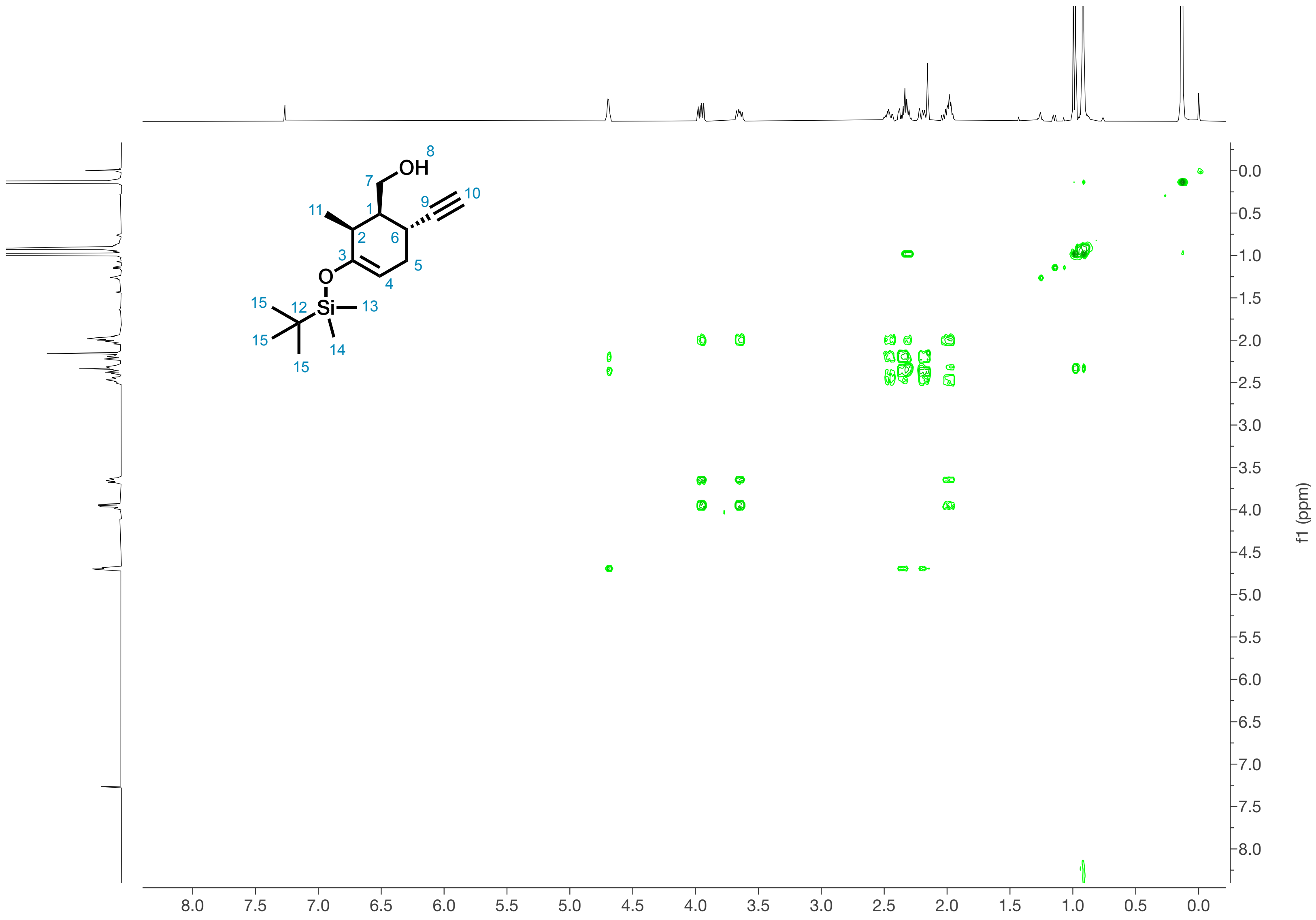

Figure S14. ^{1}H–^{1}H COSY (400 MHz, $CDCl_3$) of **primary alcohol SI-10**.

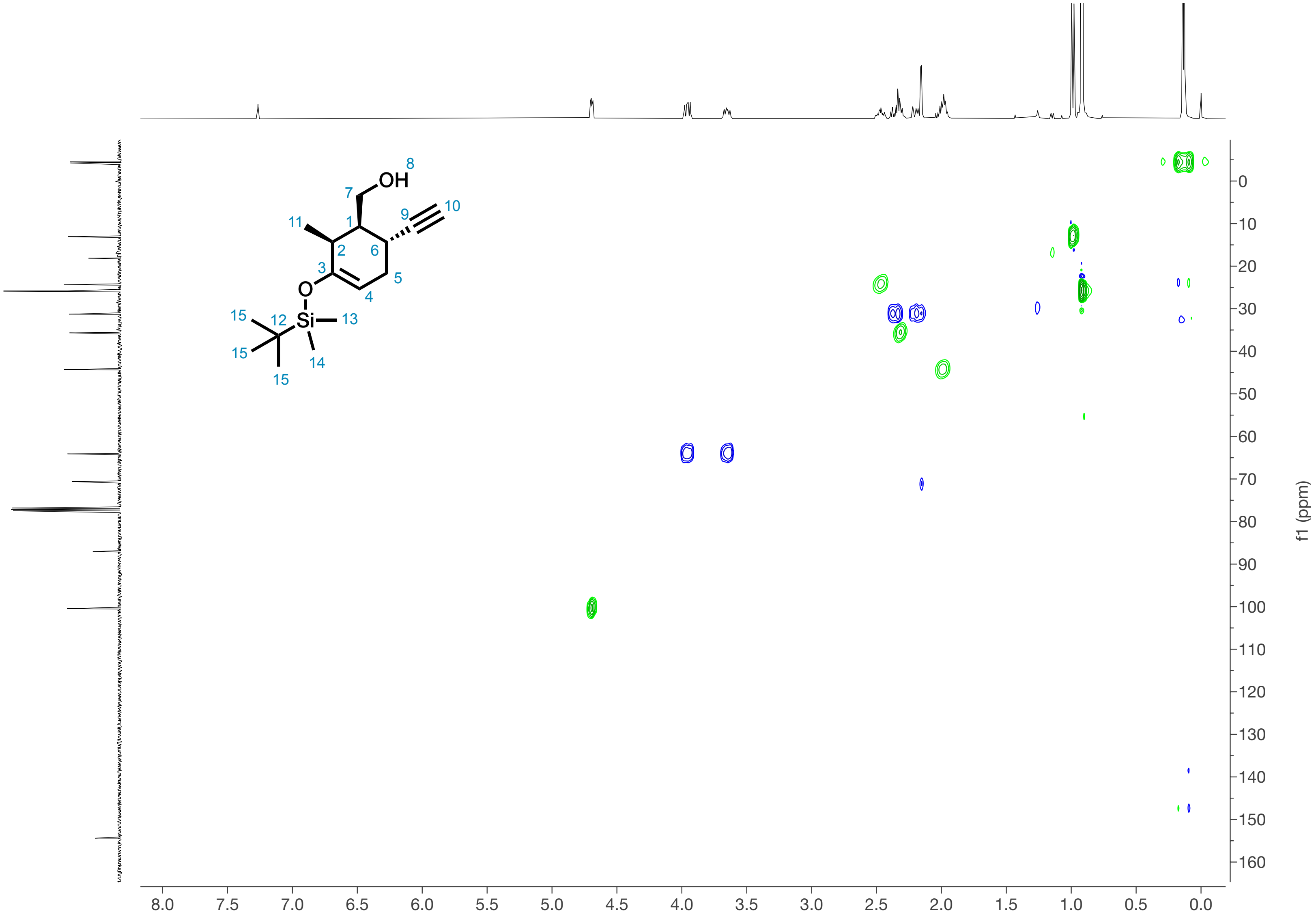

Figure S15. HSQC (400 MHz, $CDCl_3$) of **primary alcohol SI-10**.

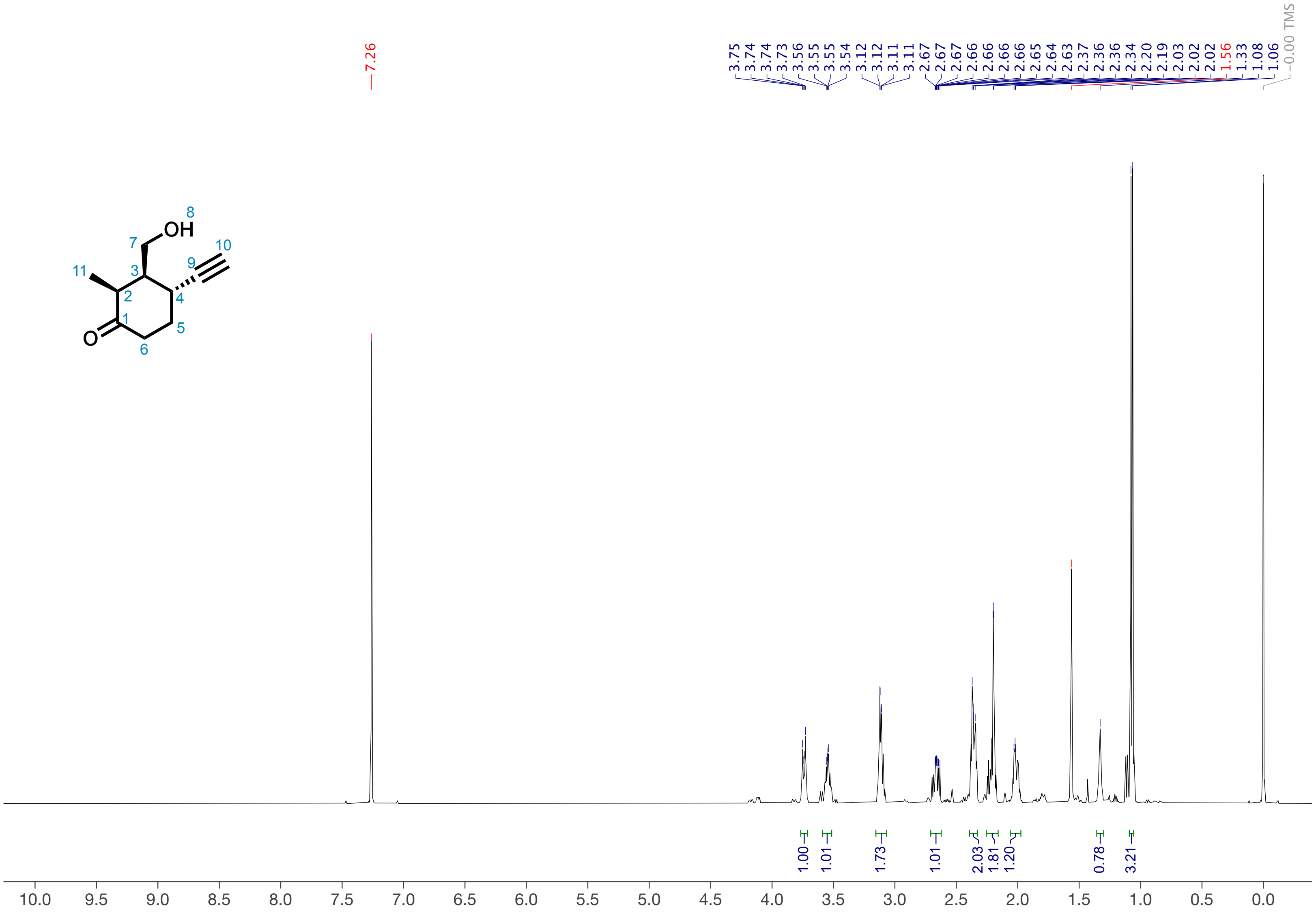

Figure S16. ^{1}H NMR (500 MHz, $CDCl_3$) of **ketone SI-5**.

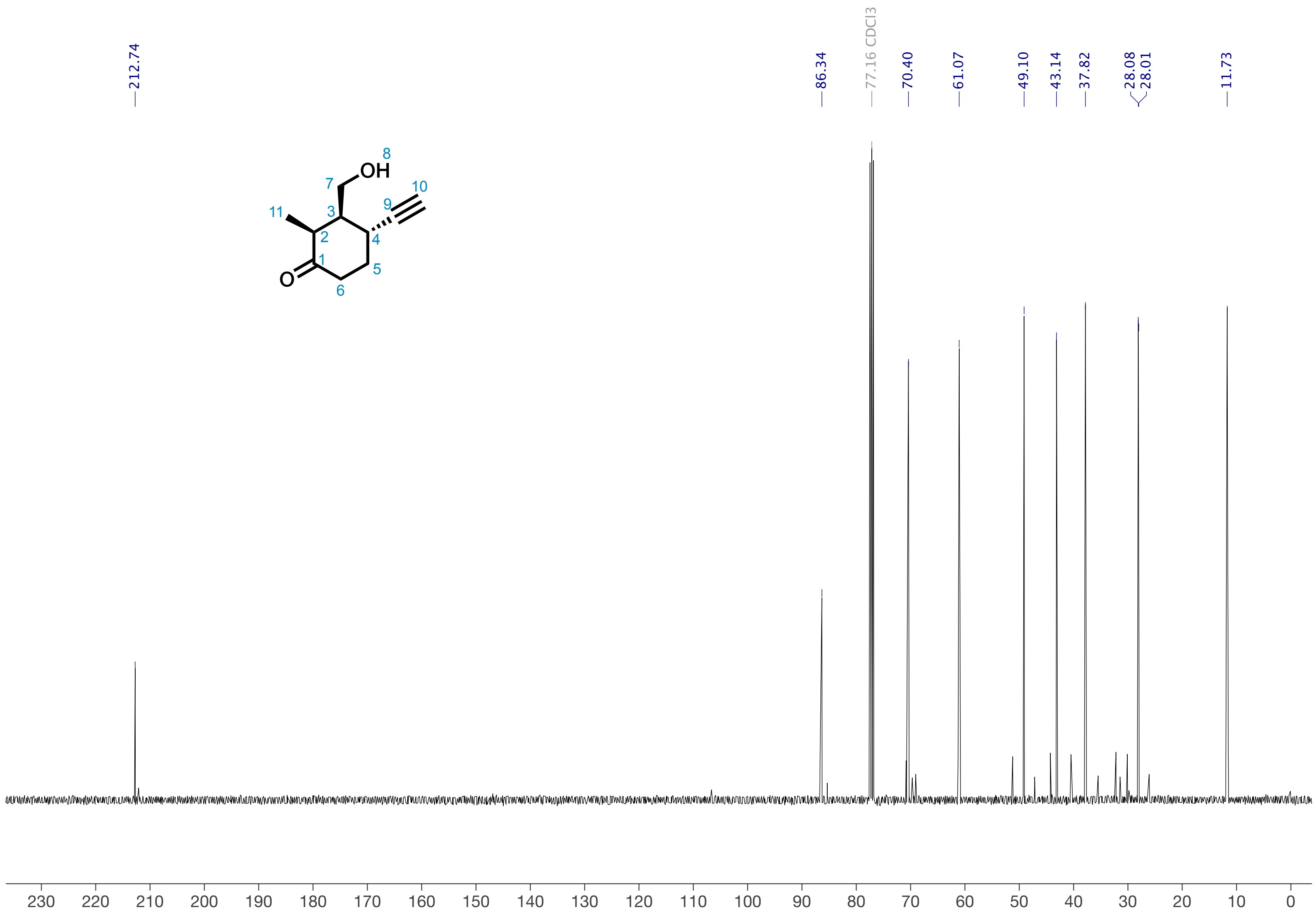

Figure S17. ^{13}C NMR (101 MHz, $CDCl_3$) of **ketone SI-5**.

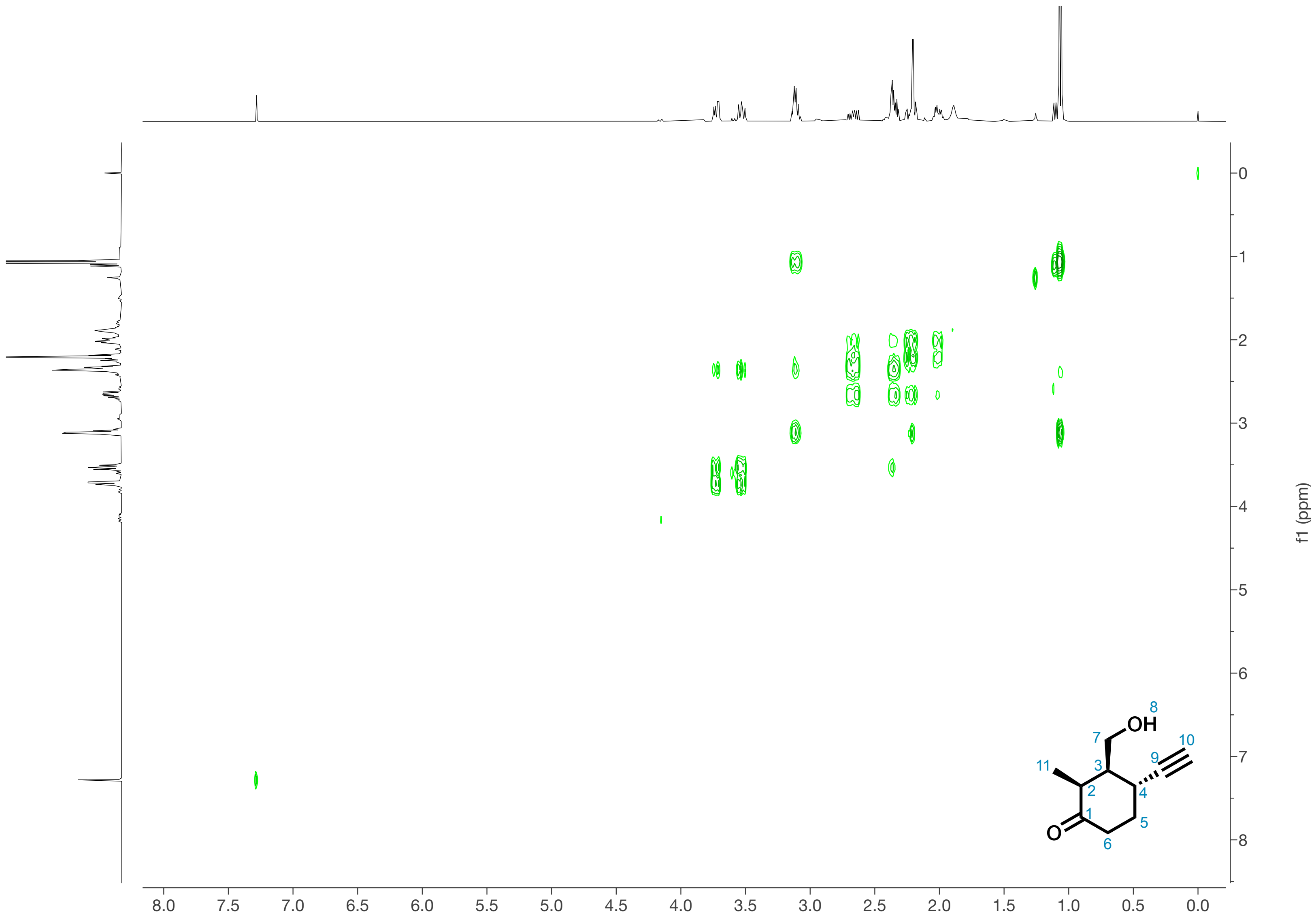

Figure S18. ^{1}H–^{1}H COSY (400 MHz, $CDCl_3$) of **ketone SI-5**.

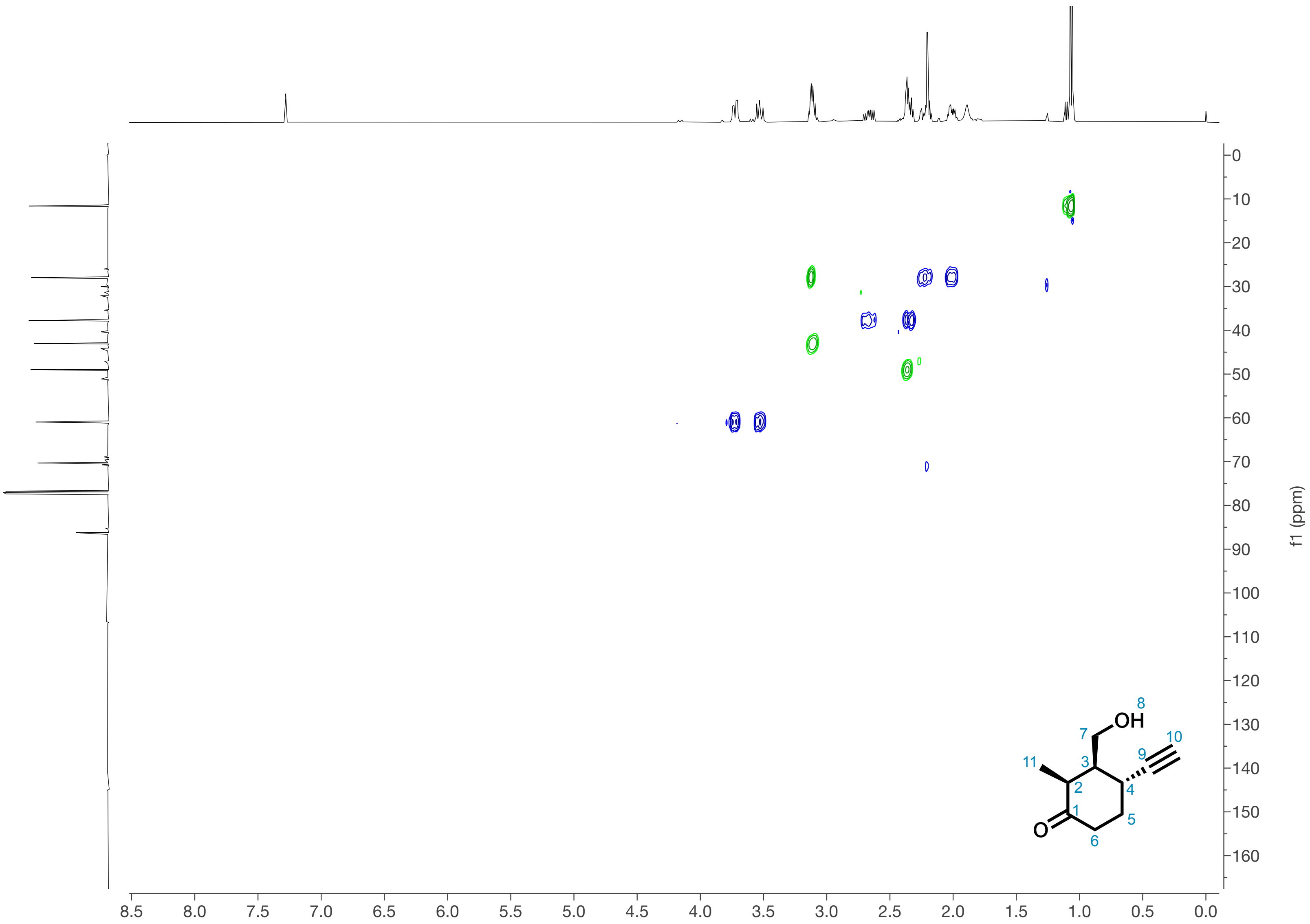

Figure S19. HSQC (400 MHz, $CDCl_3$) of **ketone SI-5**.

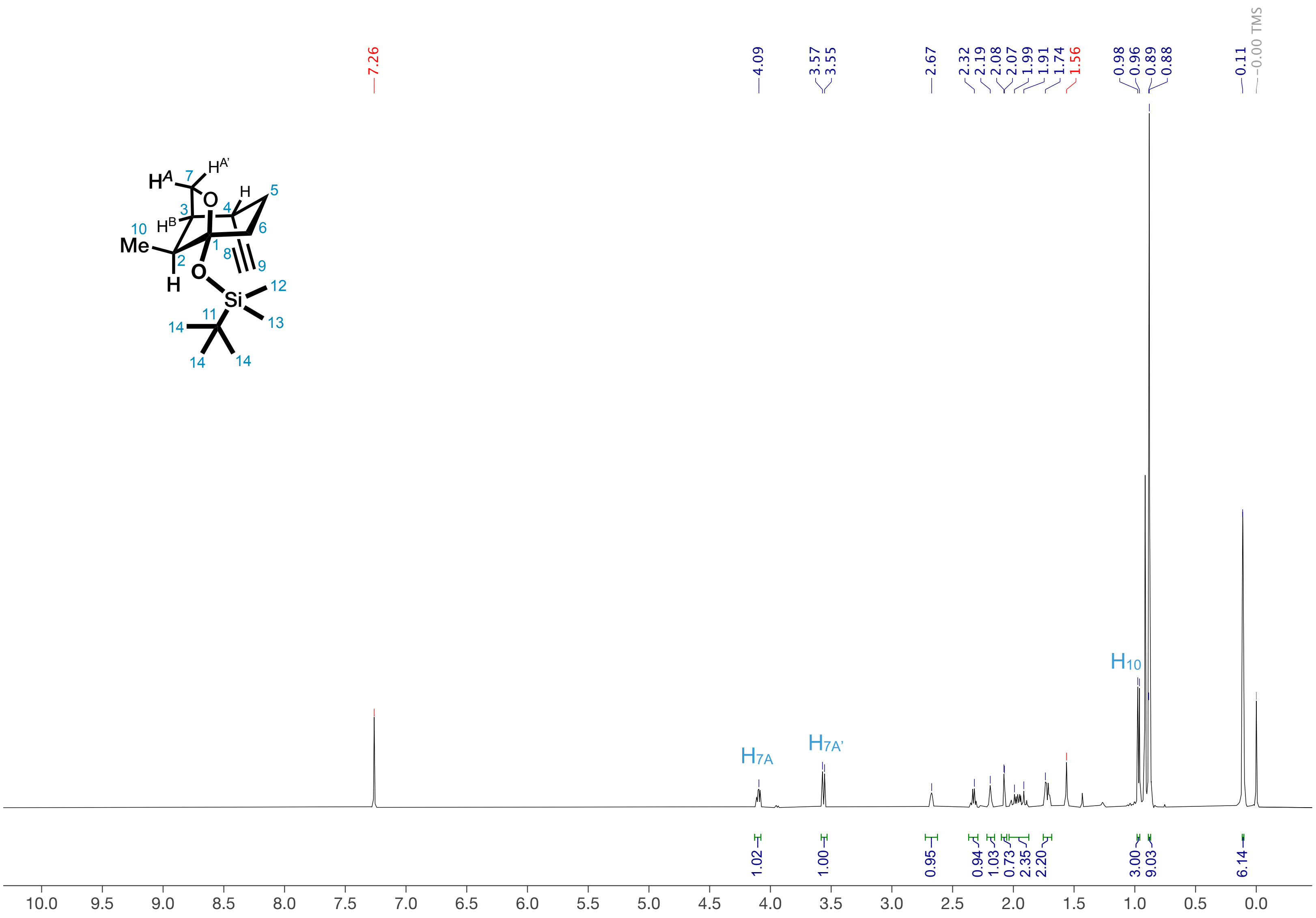

Figure S20. ^{1}H NMR (500 MHz, $CDCl_3$) of **bridged bicyclic side product SI-14**.

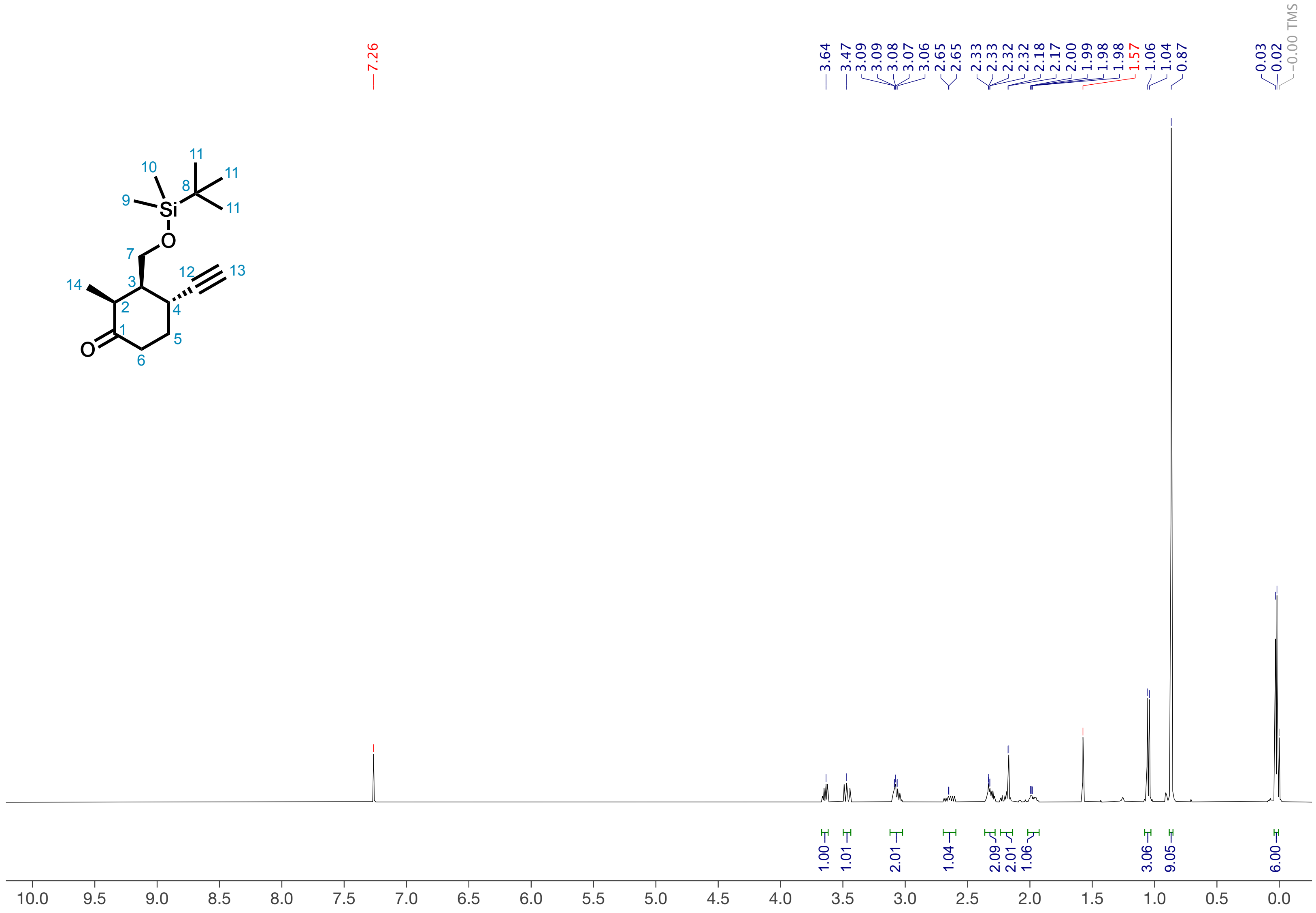

Figure S21. ^{1}H NMR (400 MHz, $CDCl_3$) of **TBS ether 12**.

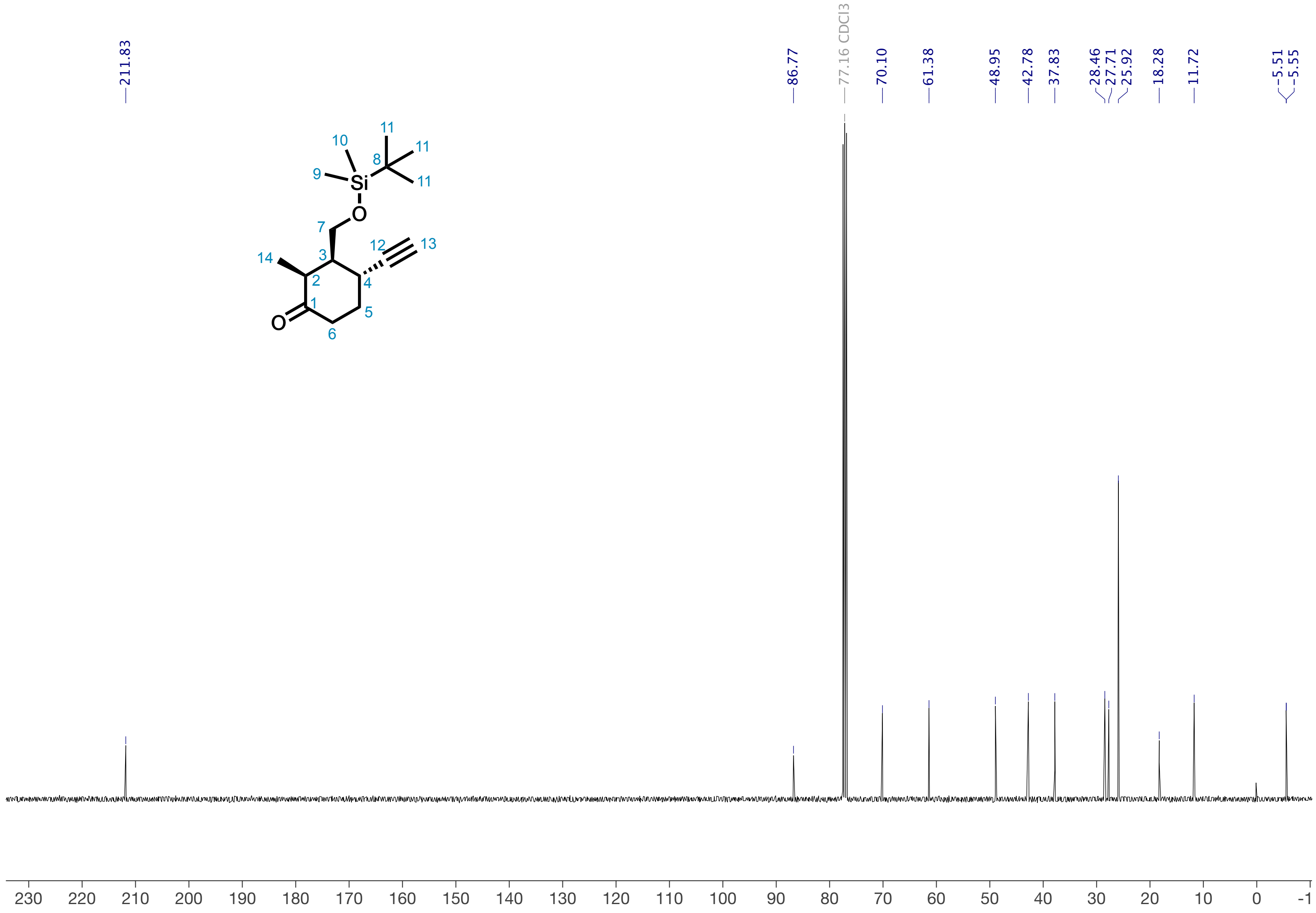

Figure S22. ^{13}C NMR (101 MHz, $CDCl_3$) of **TBS ether 12**.

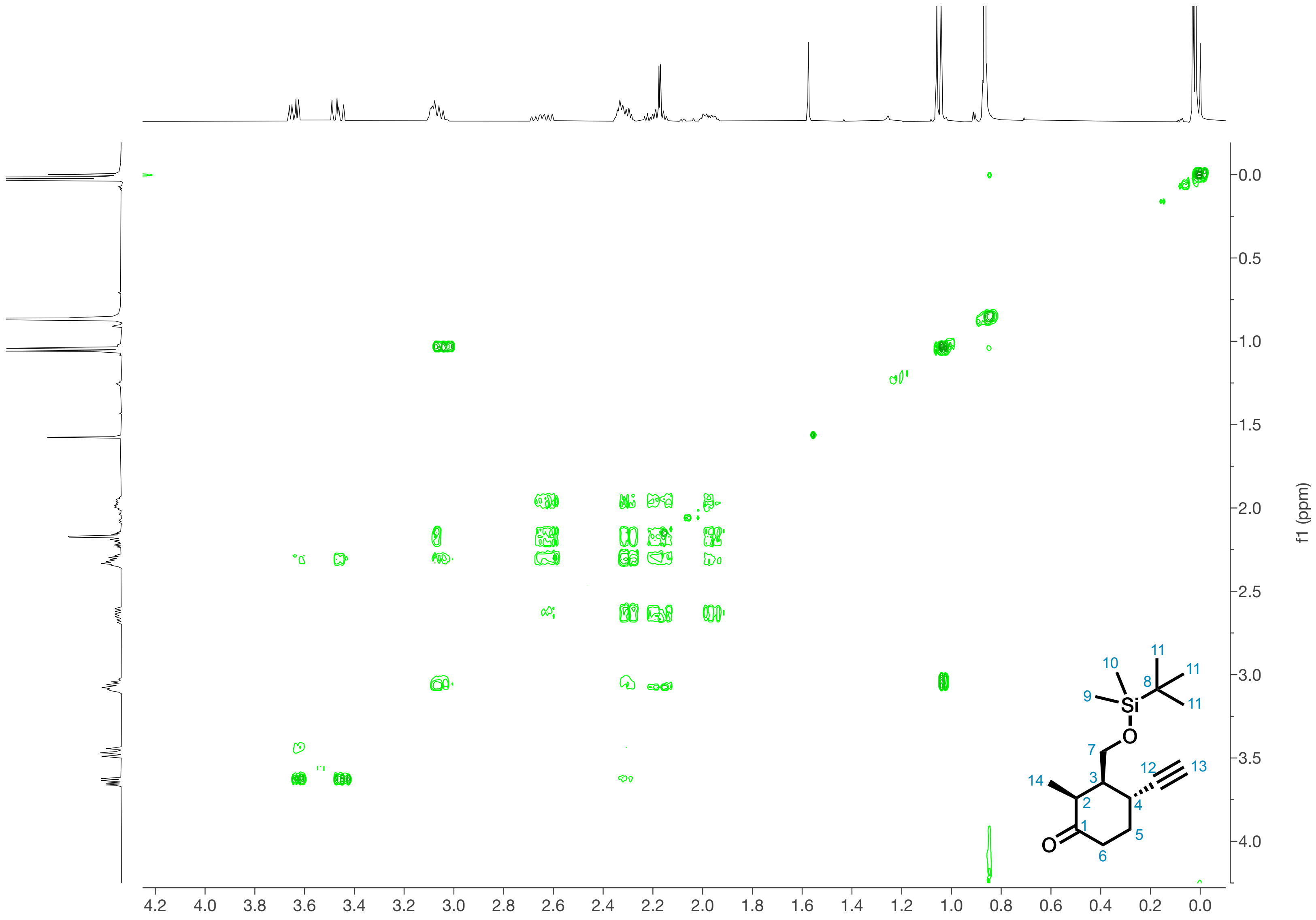

Figure S23. ^1H–^1H COSY (400 MHz, $CDCl_3$) of **TBS ether 12**.

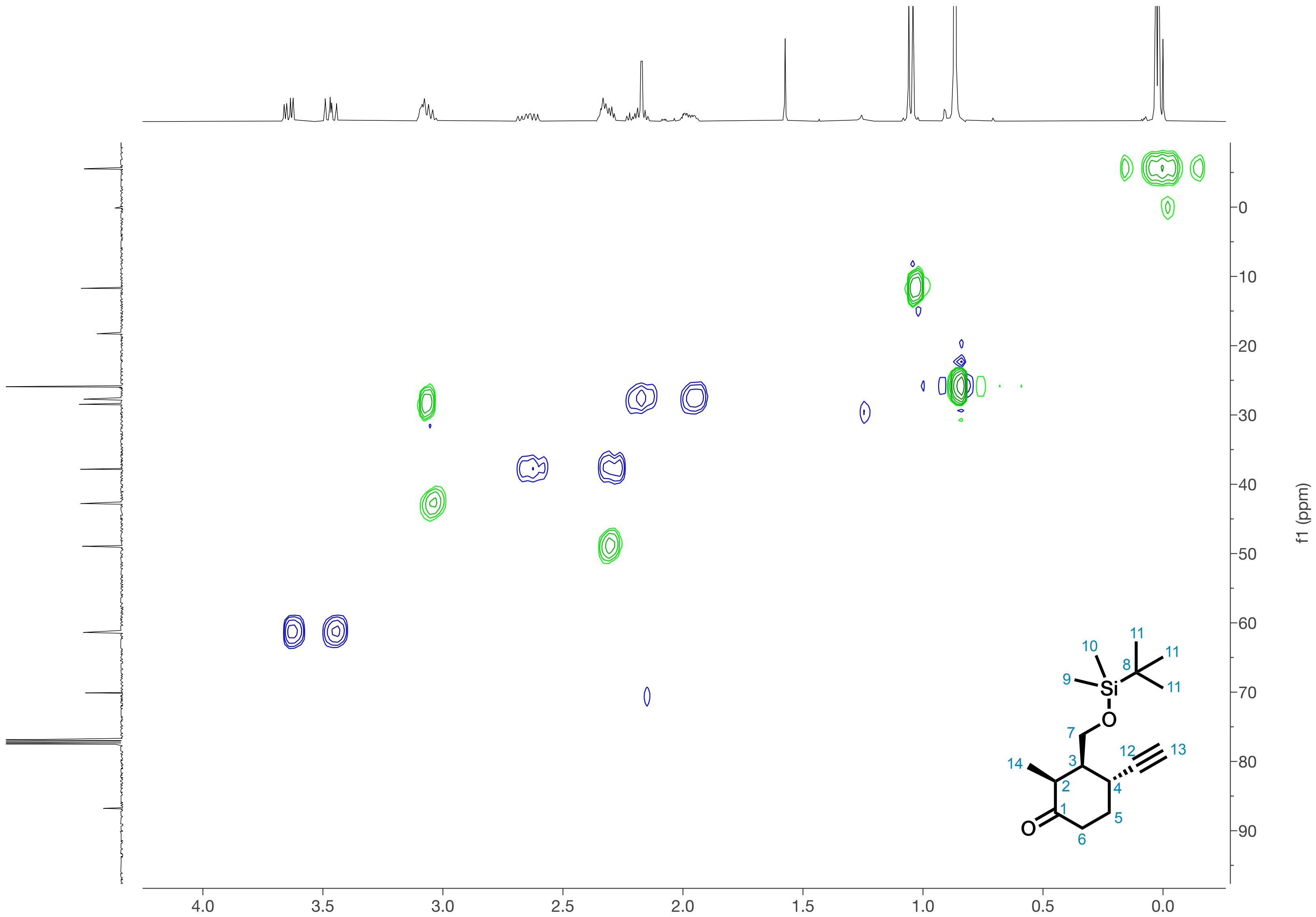

Figure S24. HSQC (400 MHz, $CDCl_3$) of **TBS ether 12**.

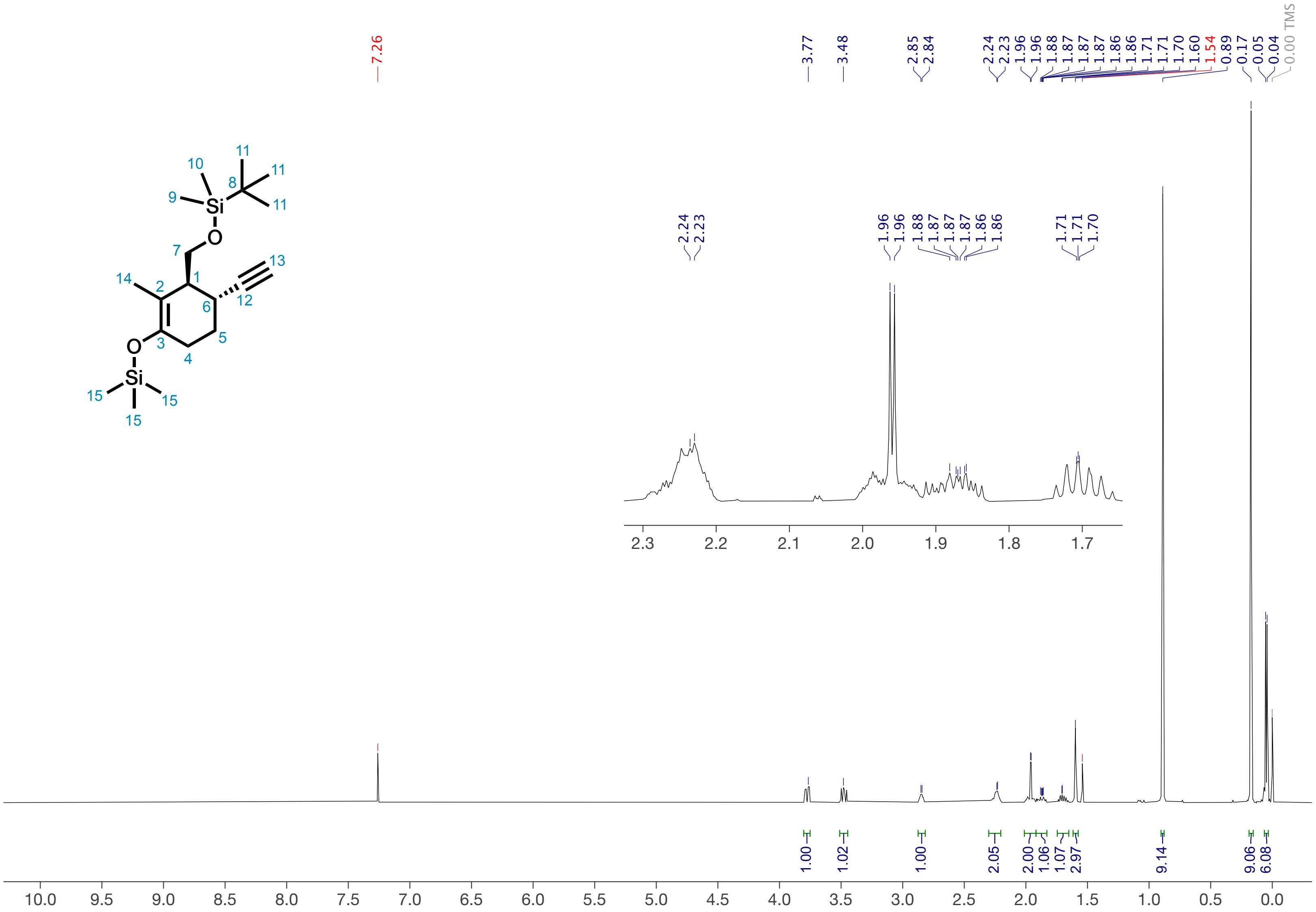

Figure S25. ^{1}H NMR (400 MHz, $CDCl_3$) of **TMS enol ether SI-6**.

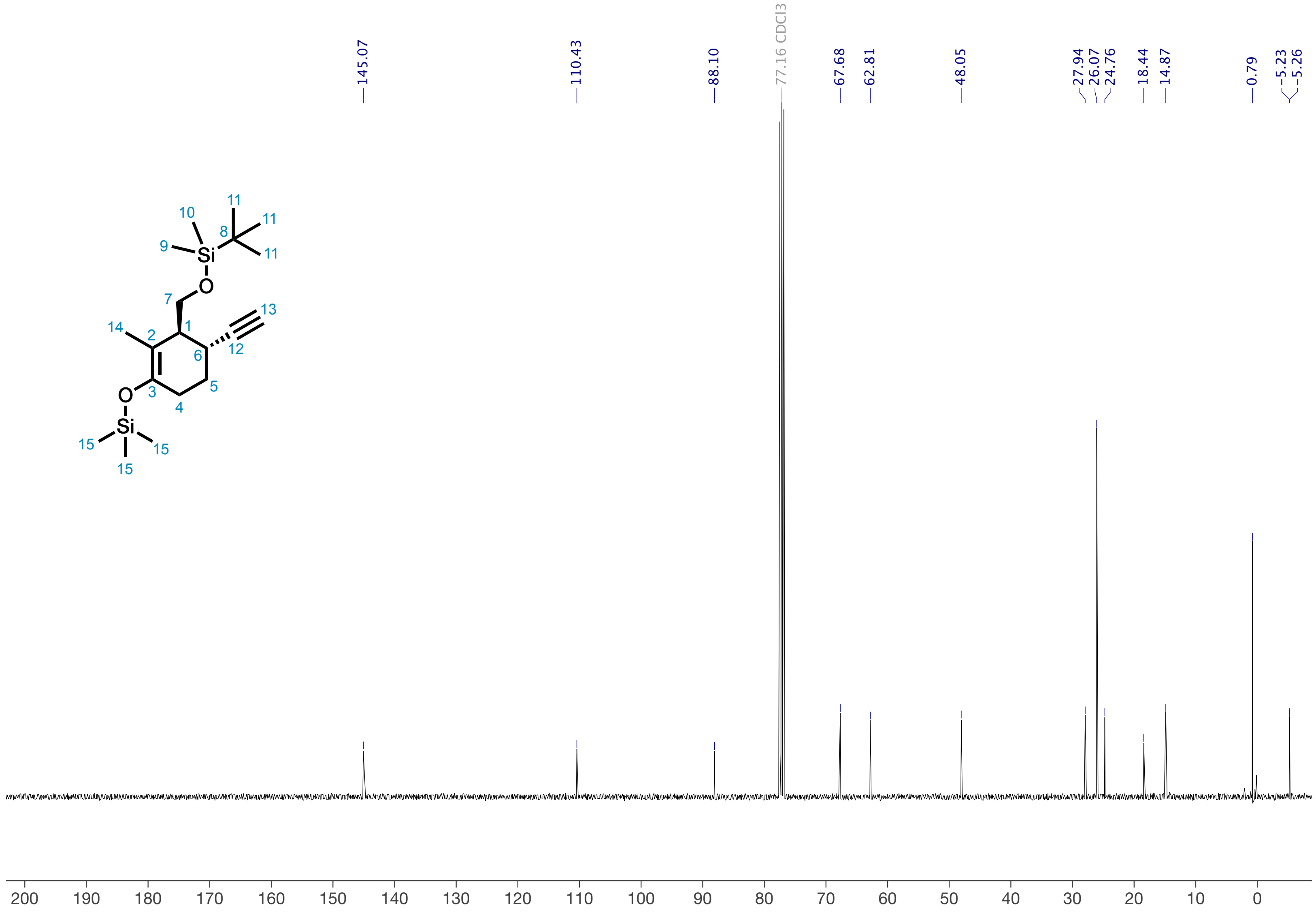

Figure S26. ^{13}C NMR (101 MHz, $CDCl_3$) of **TMS enol ether SI-6**.

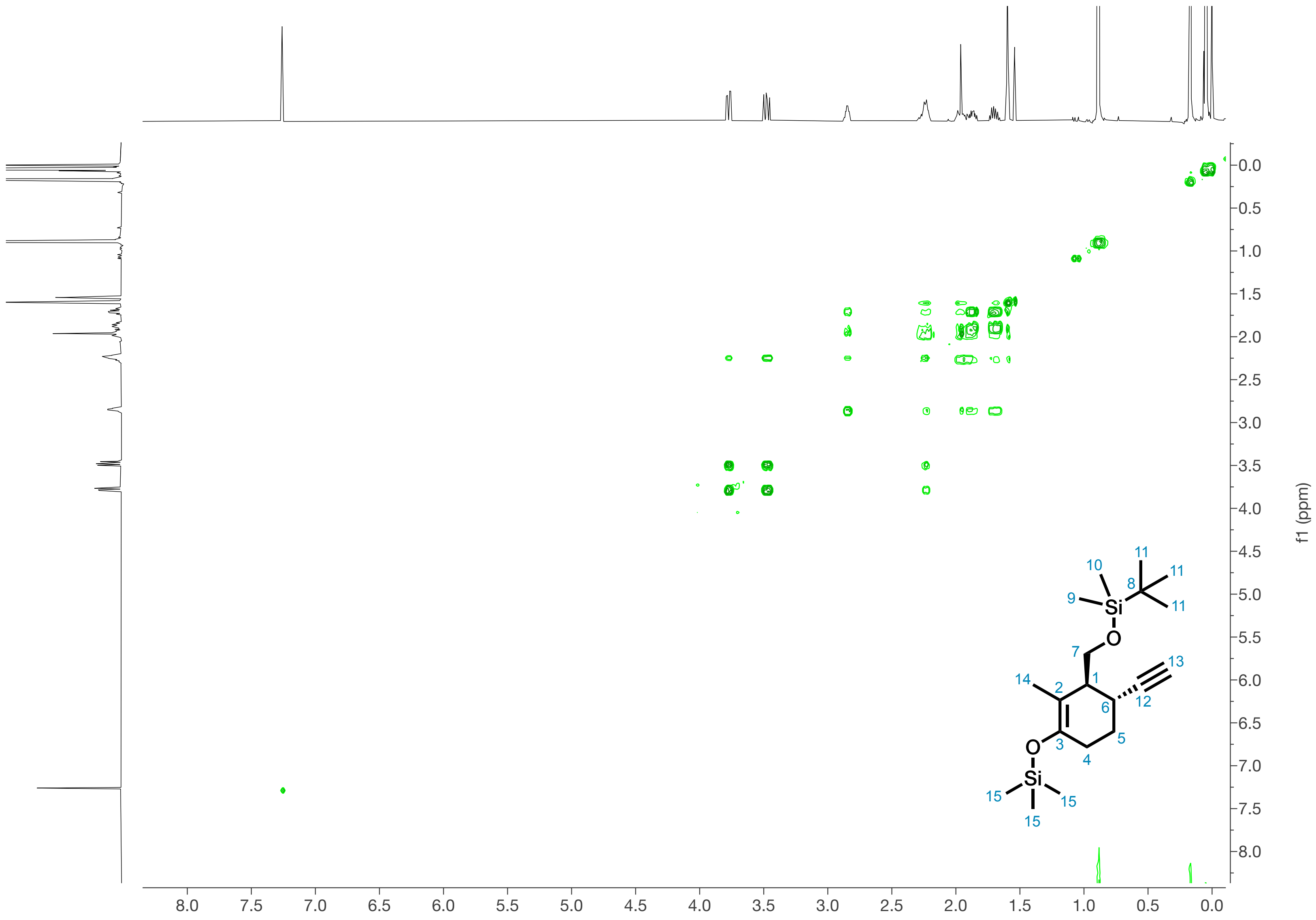

Figure S27. ^{1}H–^{1}H COSY (400 MHz, $CDCl_3$) of **TMS enol ether SI-6**.

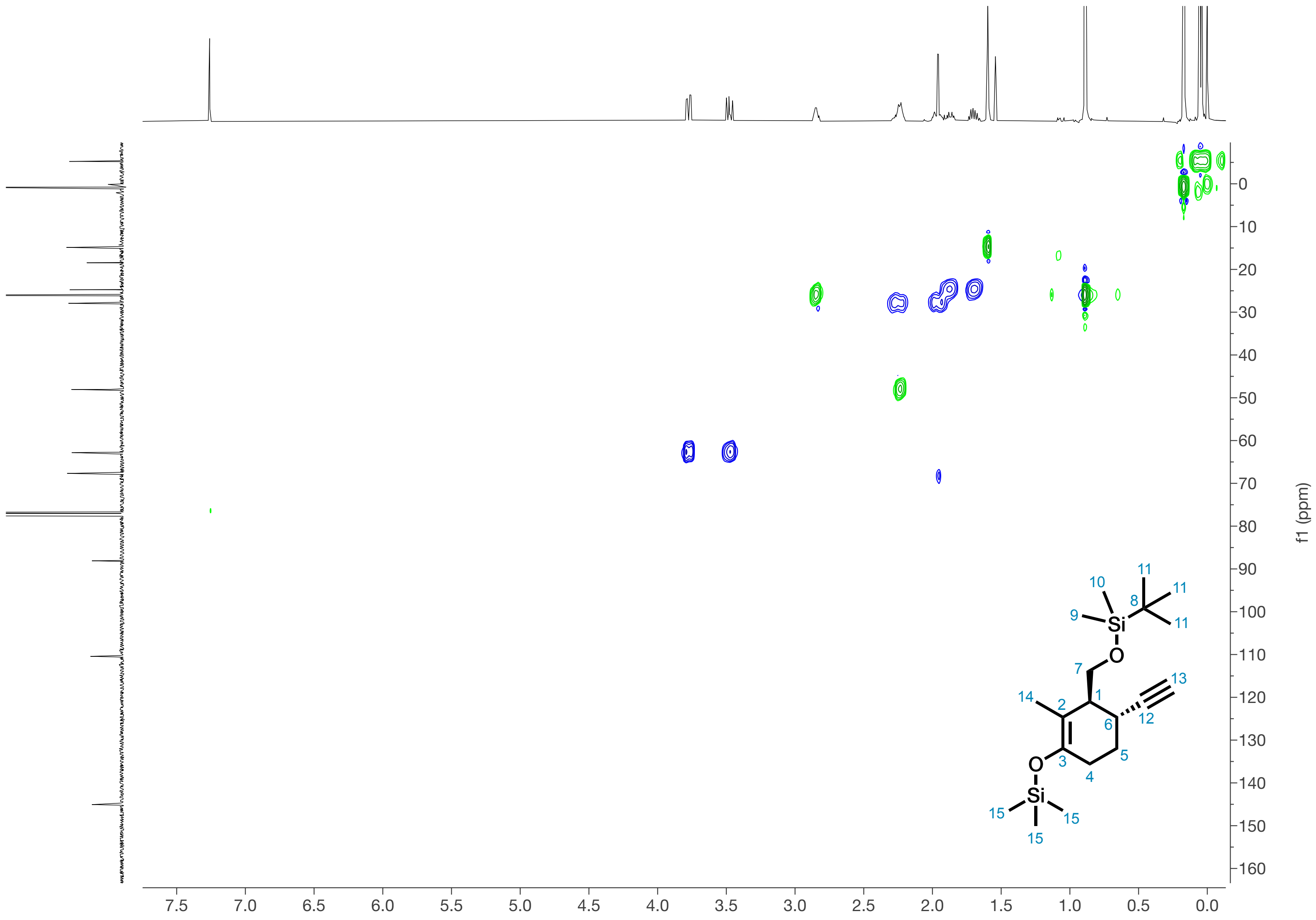

Figure S28. HSQC (400 MHz, $CDCl_3$) of **TMS enol ether SI-6**.

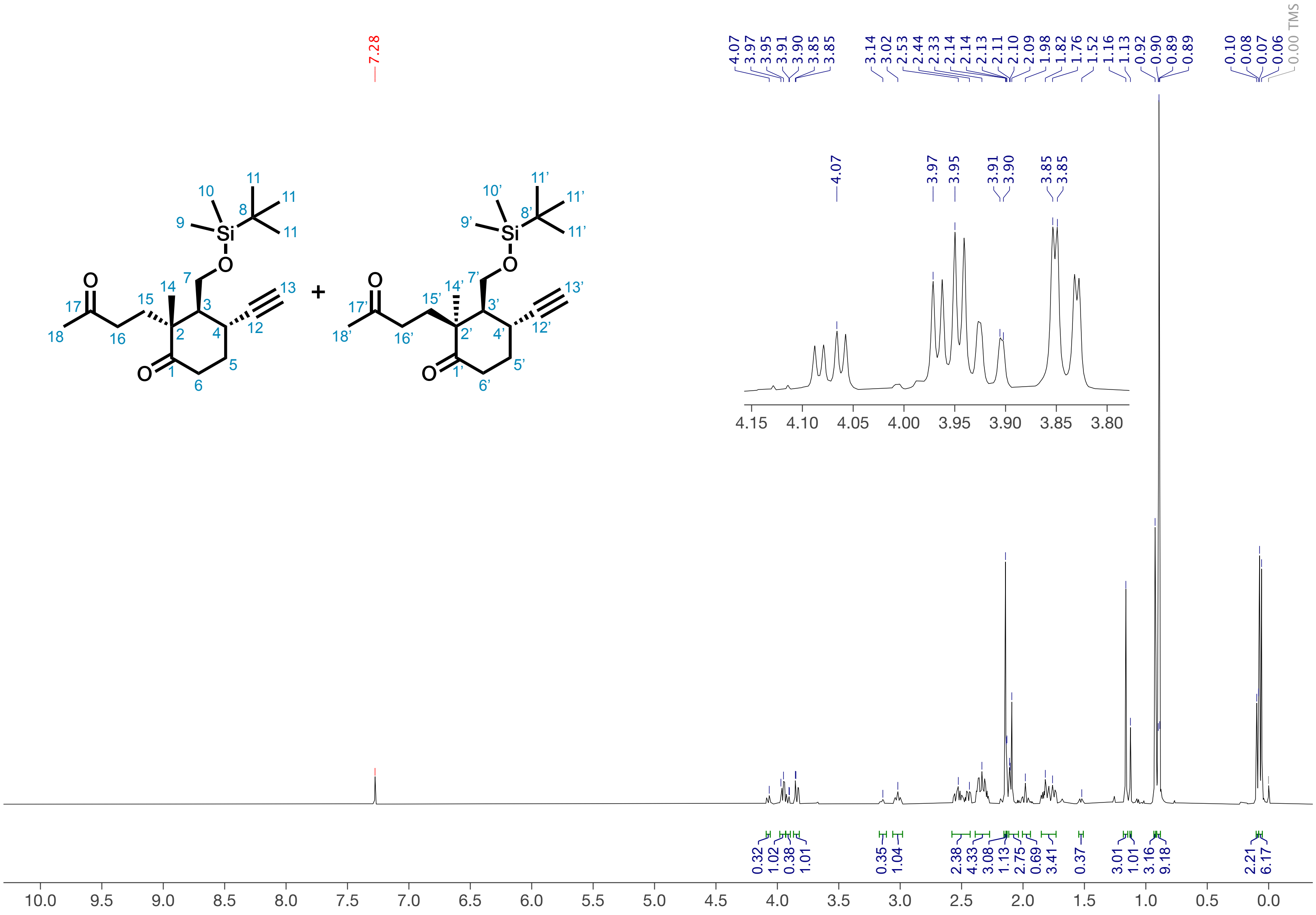

Figure S29. ^{1}H NMR (400 MHz, $CDCl_3$) of **1,5-diketone SI-7/SI-7'**.

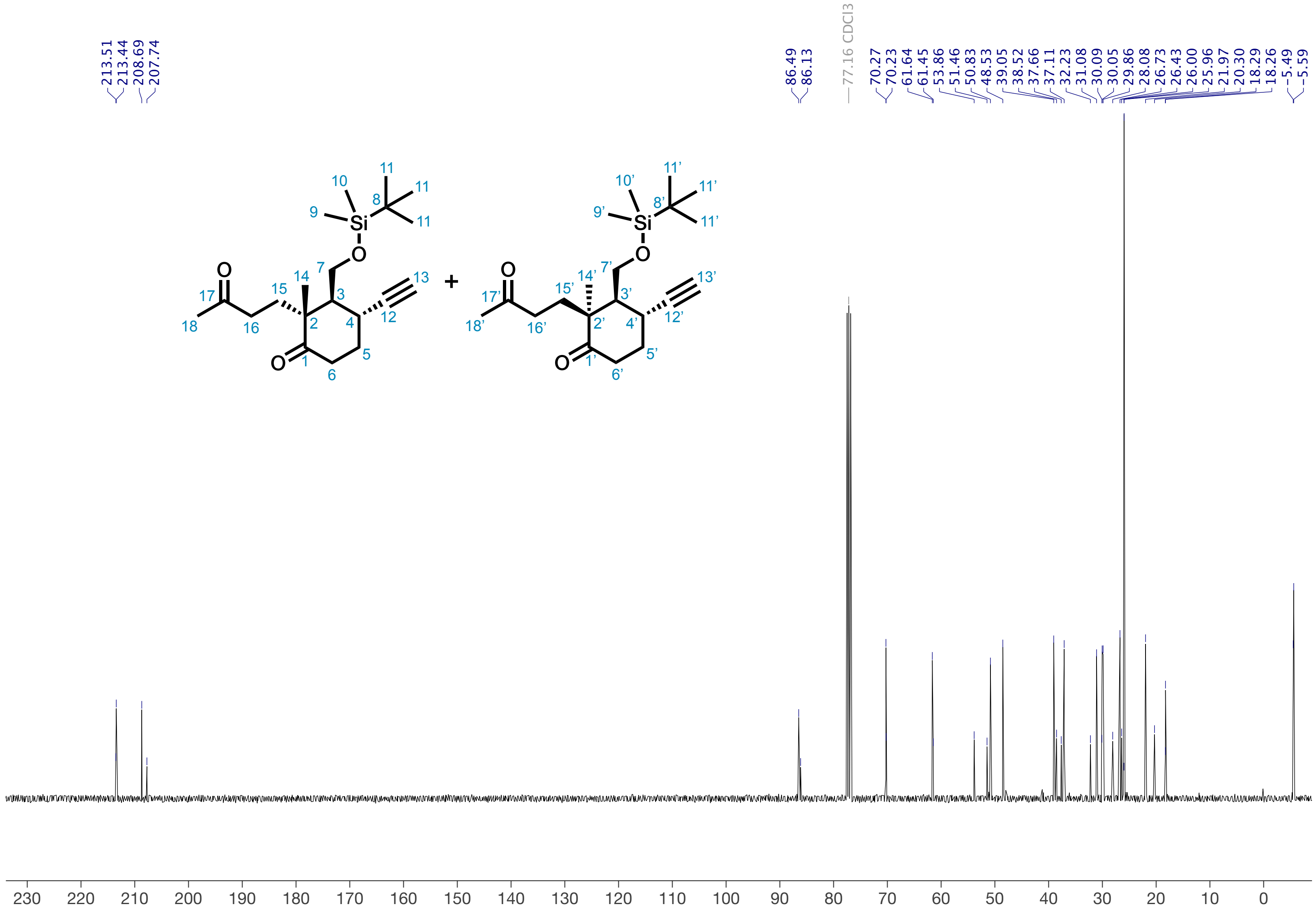

Figure S30. ^{13}C NMR (101 MHz, $CDCl_3$) of **1,5-diketone SI-7/SI-7'**.

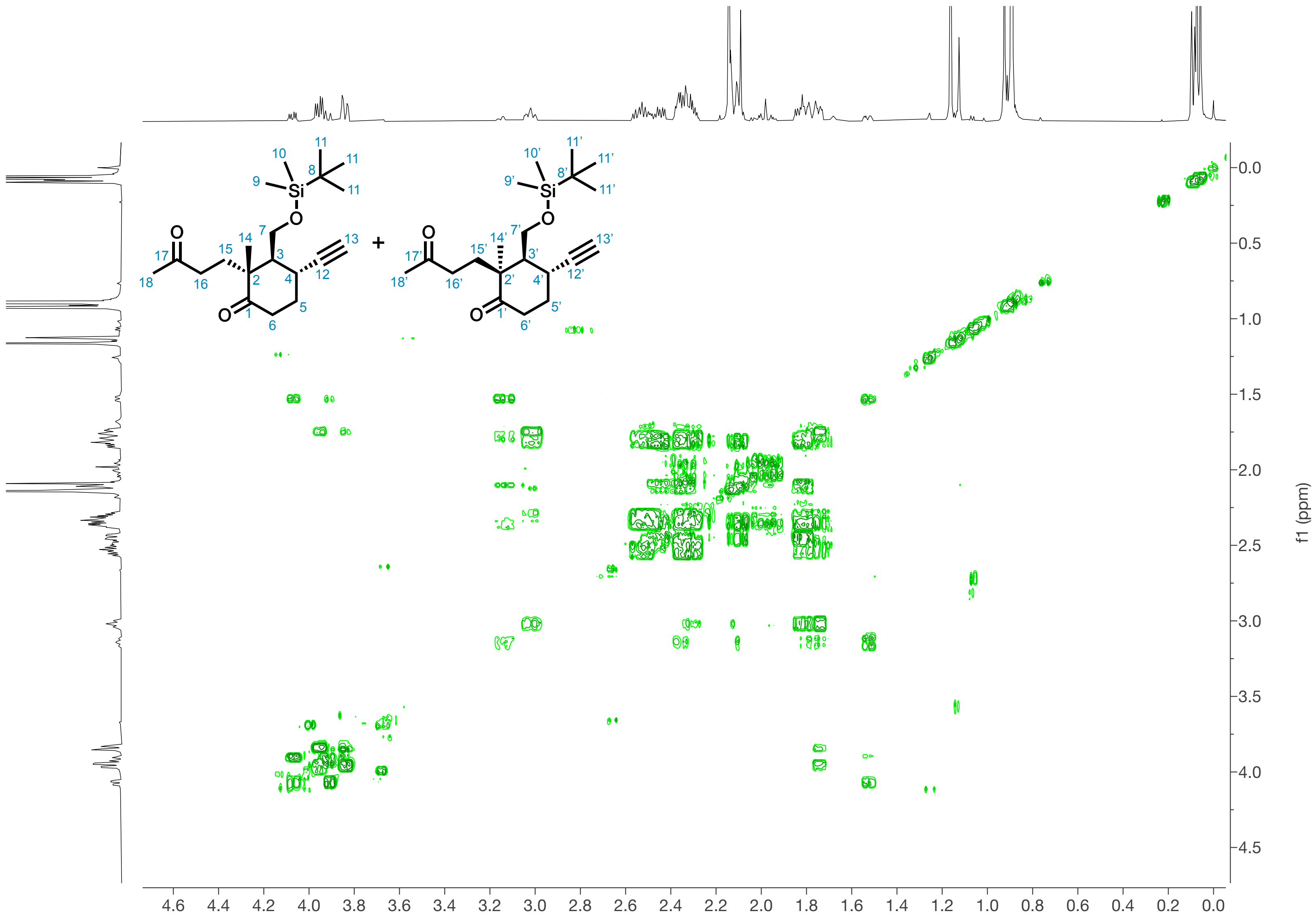

Figure S31. ^{1}H–^{1}H COSY (400 MHz, $CDCl_3$) of **1,5-diketone SI-7/SI-7’**.

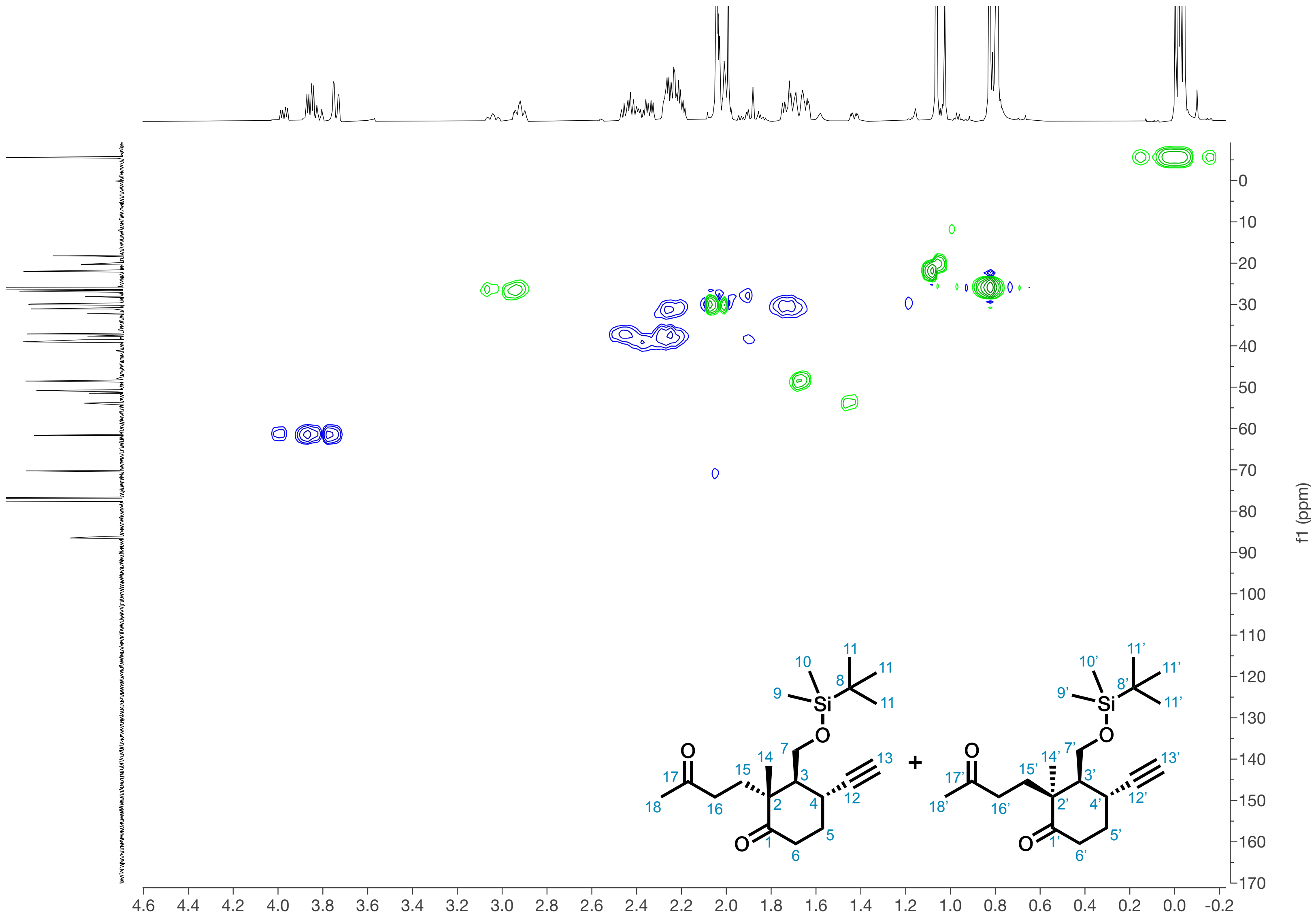

Figure S32. HSQC (400 MHz, $CDCl_3$) of **1,5-diketone SI-7/SI-7'**.

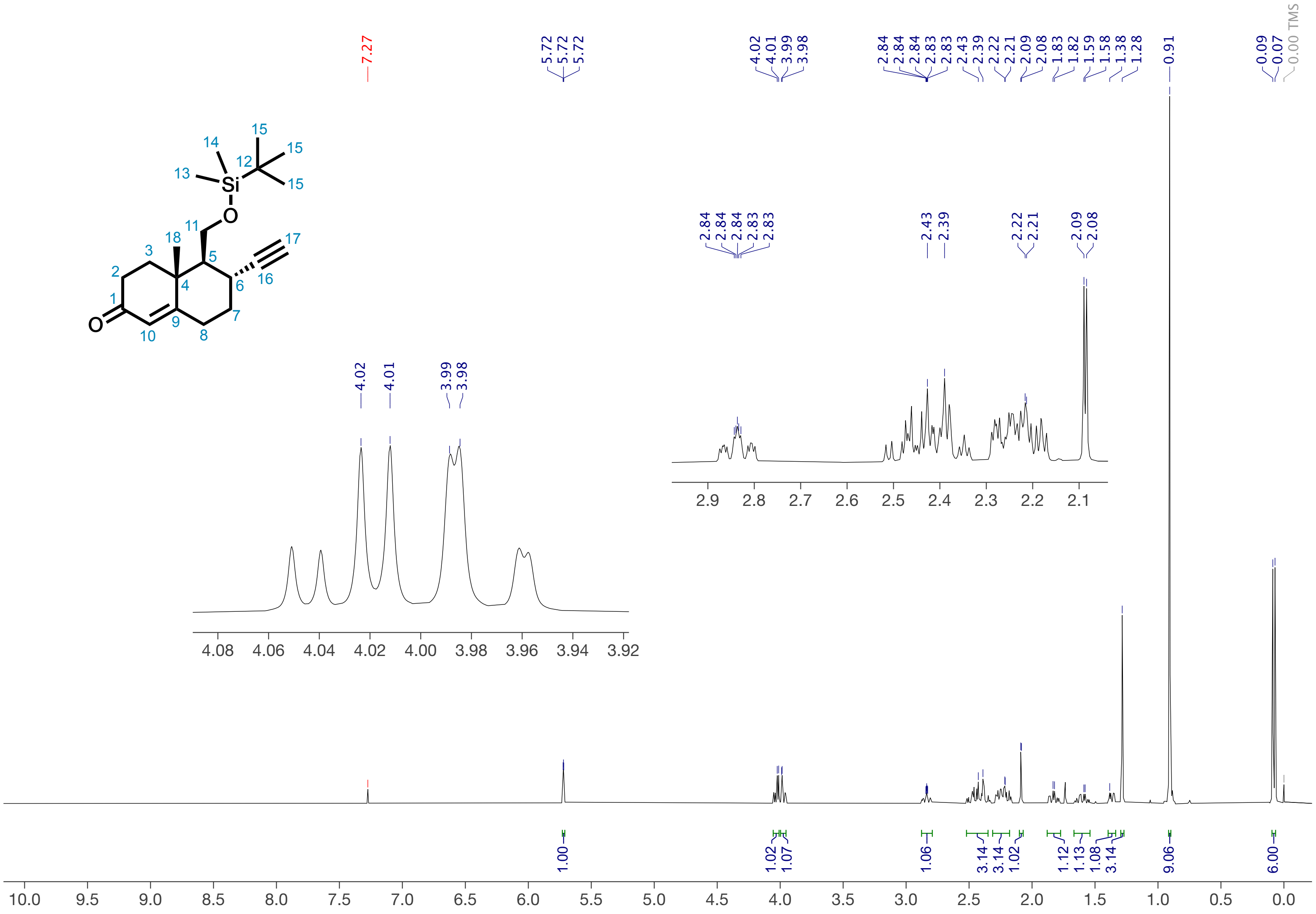

Figure S33. ^{1}H NMR (400 MHz, $CDCl_3$) of **octalone 13**.

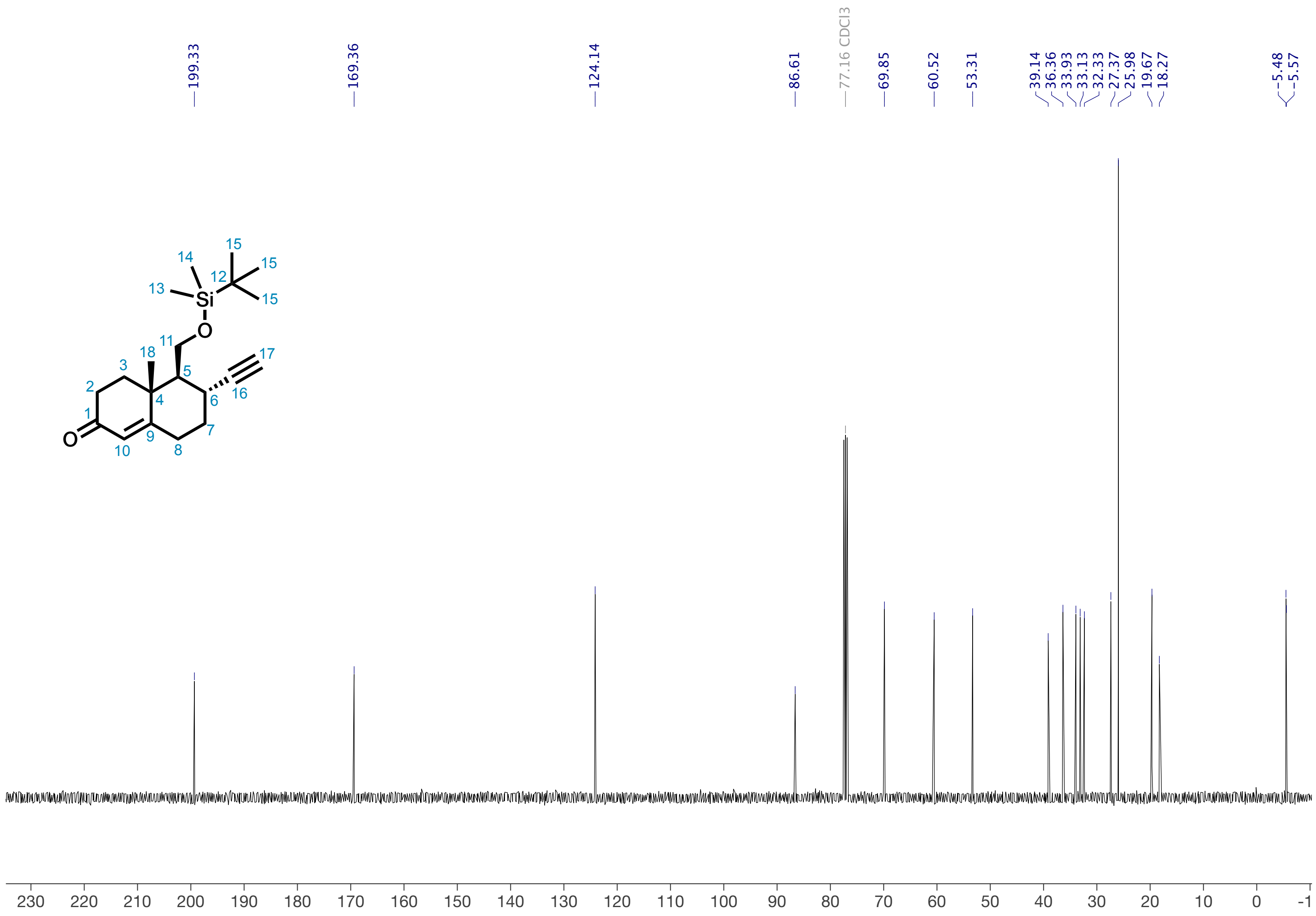

Figure S34. ^{13}C NMR (101 MHz, $CDCl_3$) of **octalone 13**.

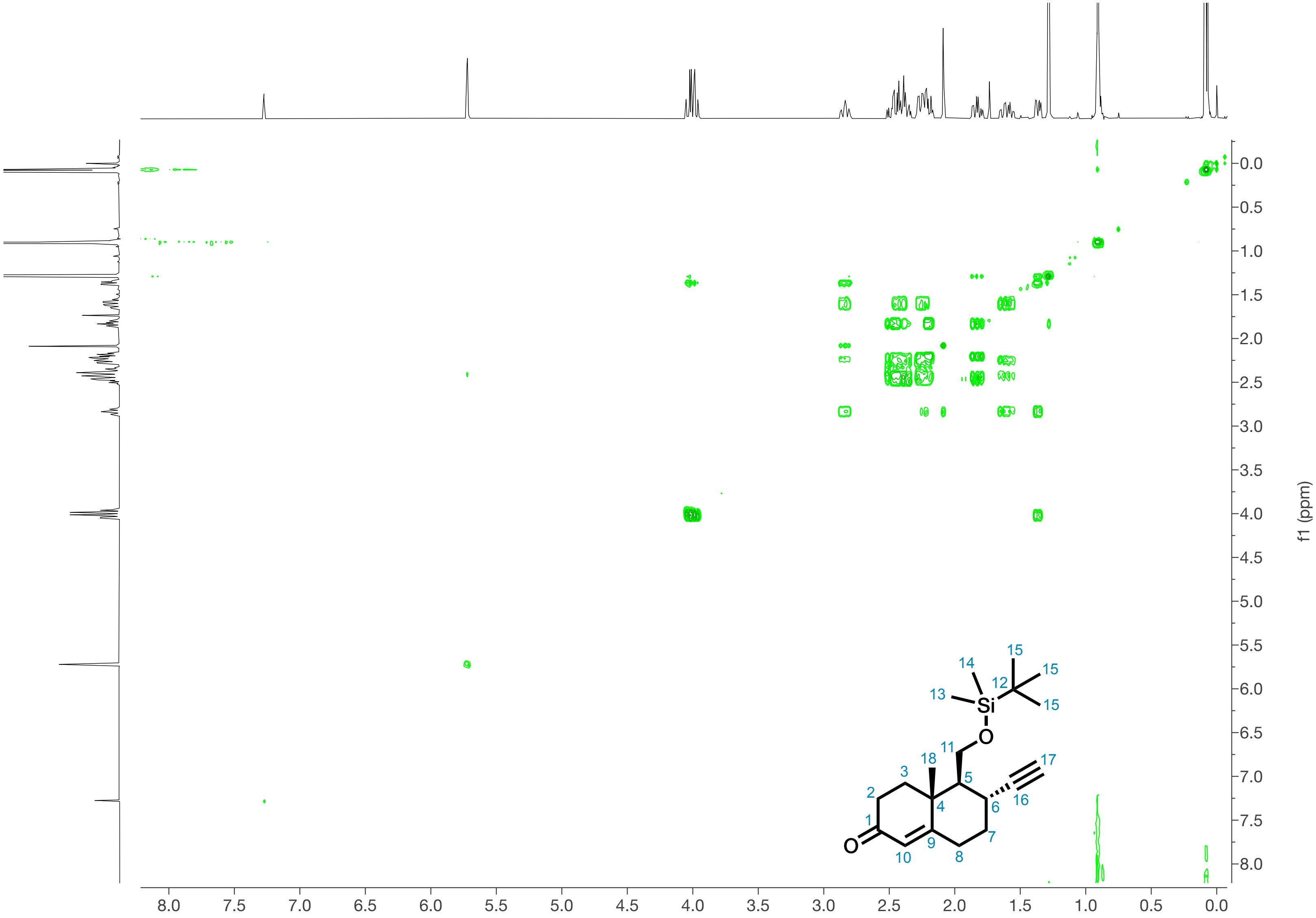

Figure S35. ^{1}H–^{1}H COSY (400 MHz, $CDCl_3$) of **octalone 13**.

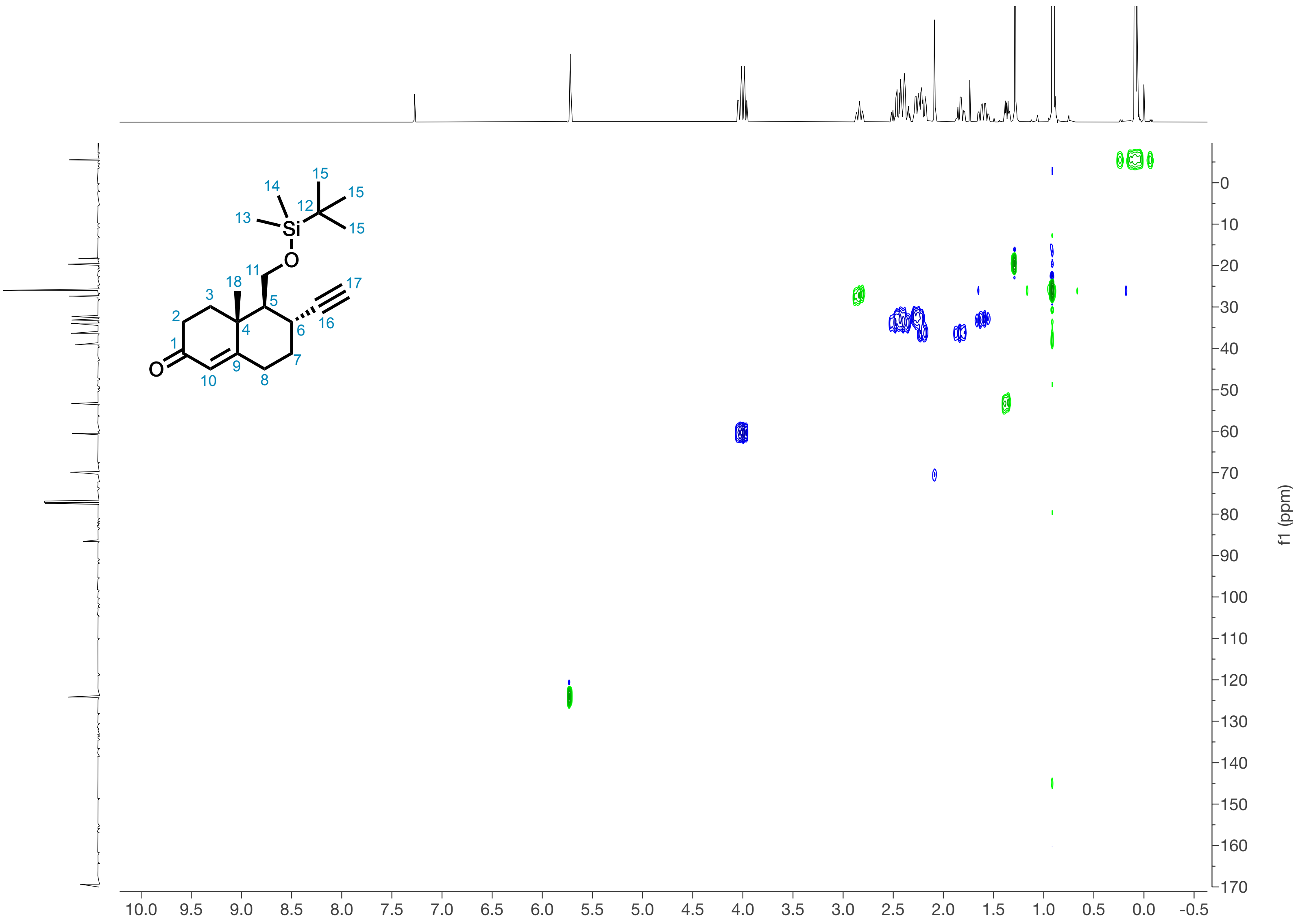

Figure S36. HSQC (400 MHz, $CDCl_3$) of **octalone 13**.

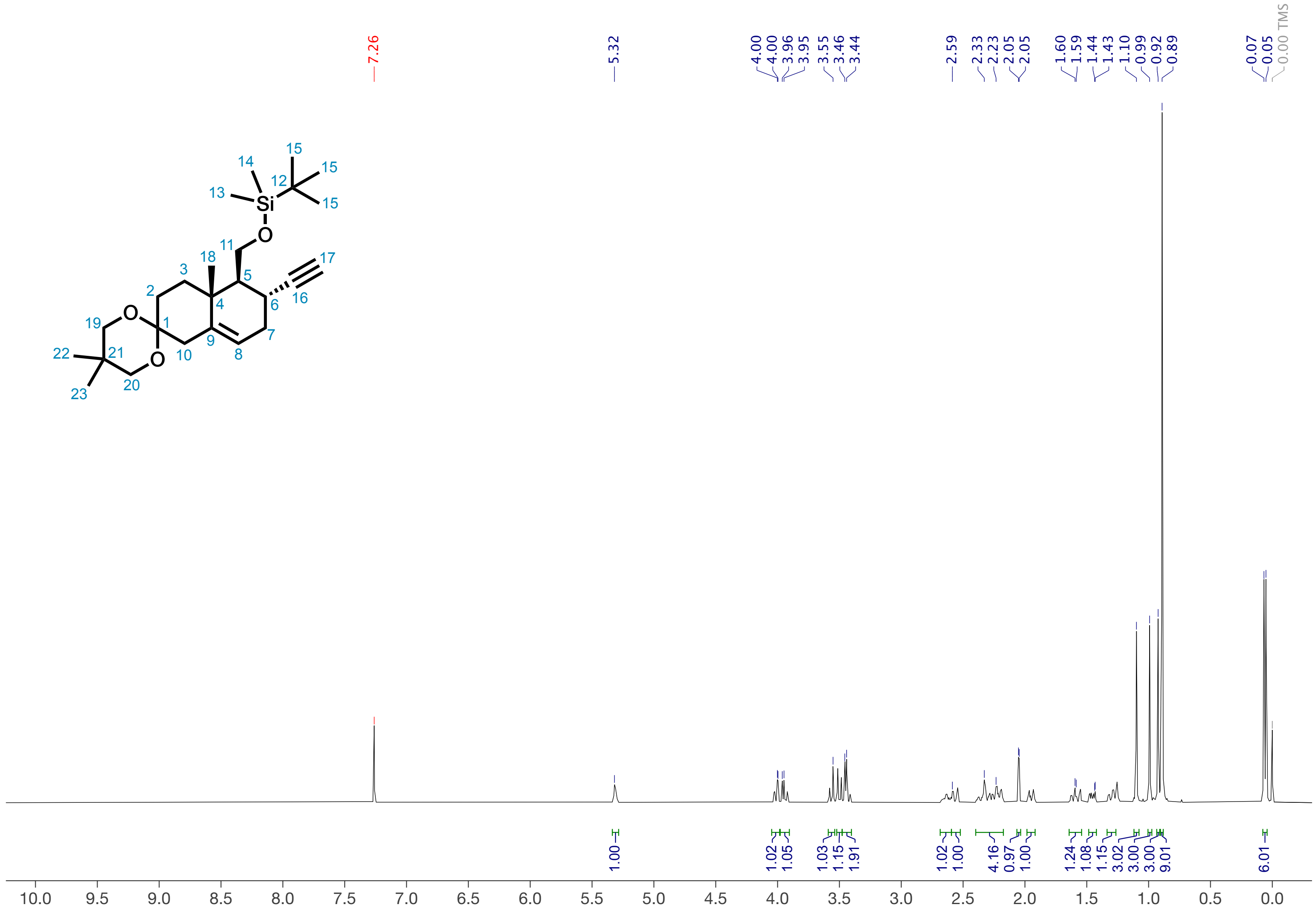

Figure S37. ^{1}H NMR (400 MHz, $CDCl_3$) of **neopentyl glycol 14**.

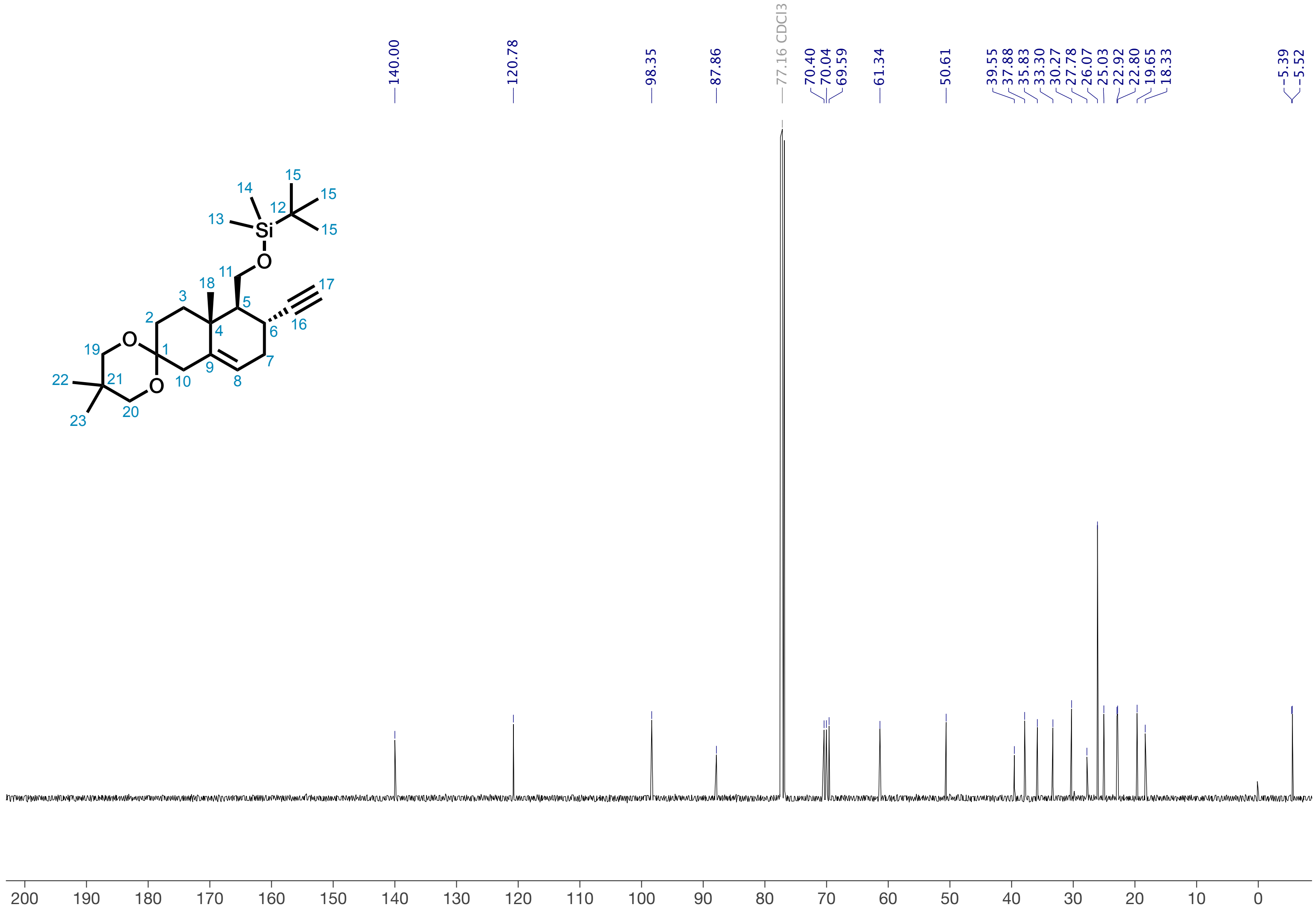

Figure S38. ^{13}C NMR (101 MHz, $CDCl_3$) of **neopentyl glycol 14**.

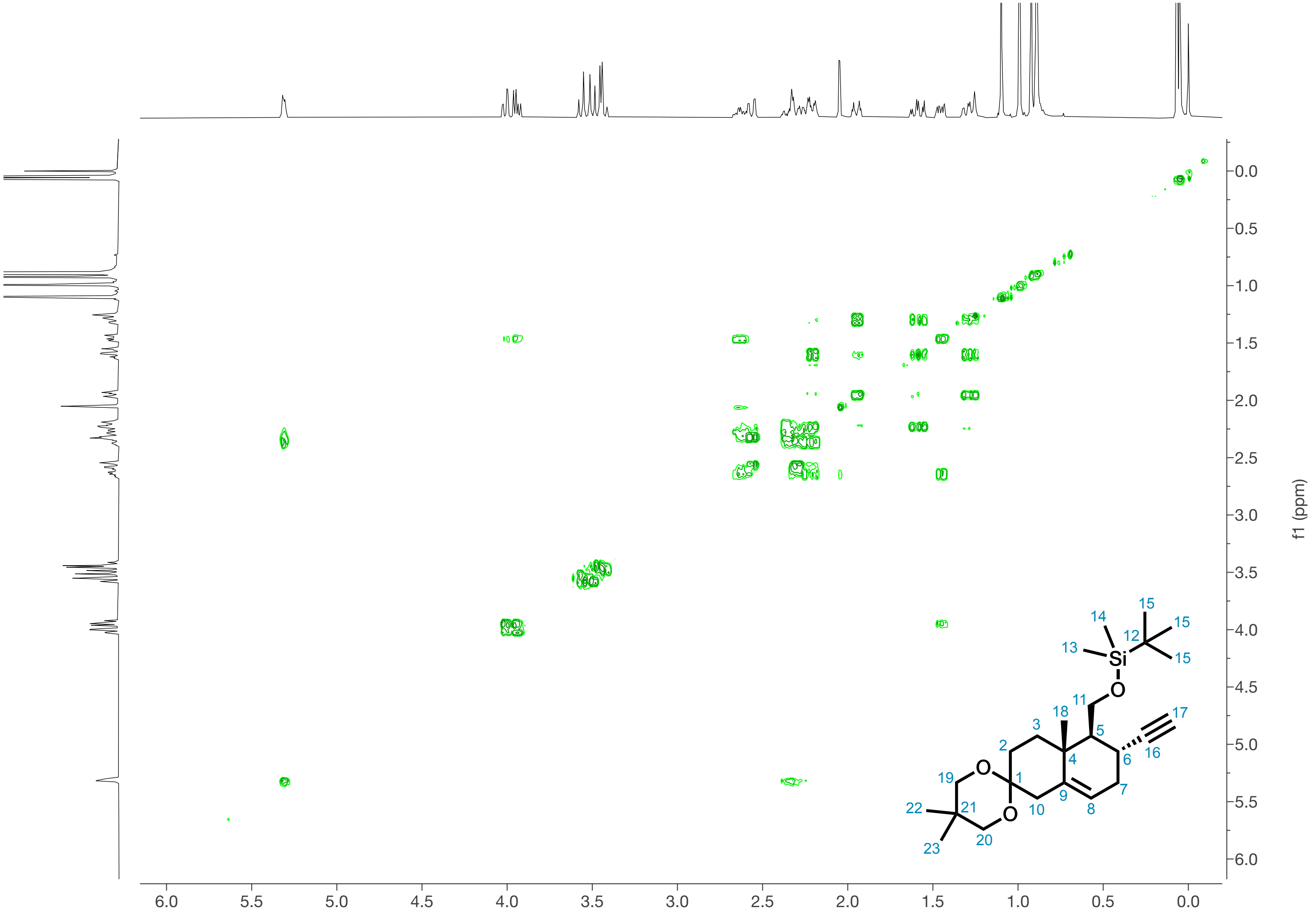

Figure S39. ^{1}H–^{1}H COSY (400 MHz, $CDCl_3$) of **neopentyl glycol 14**.

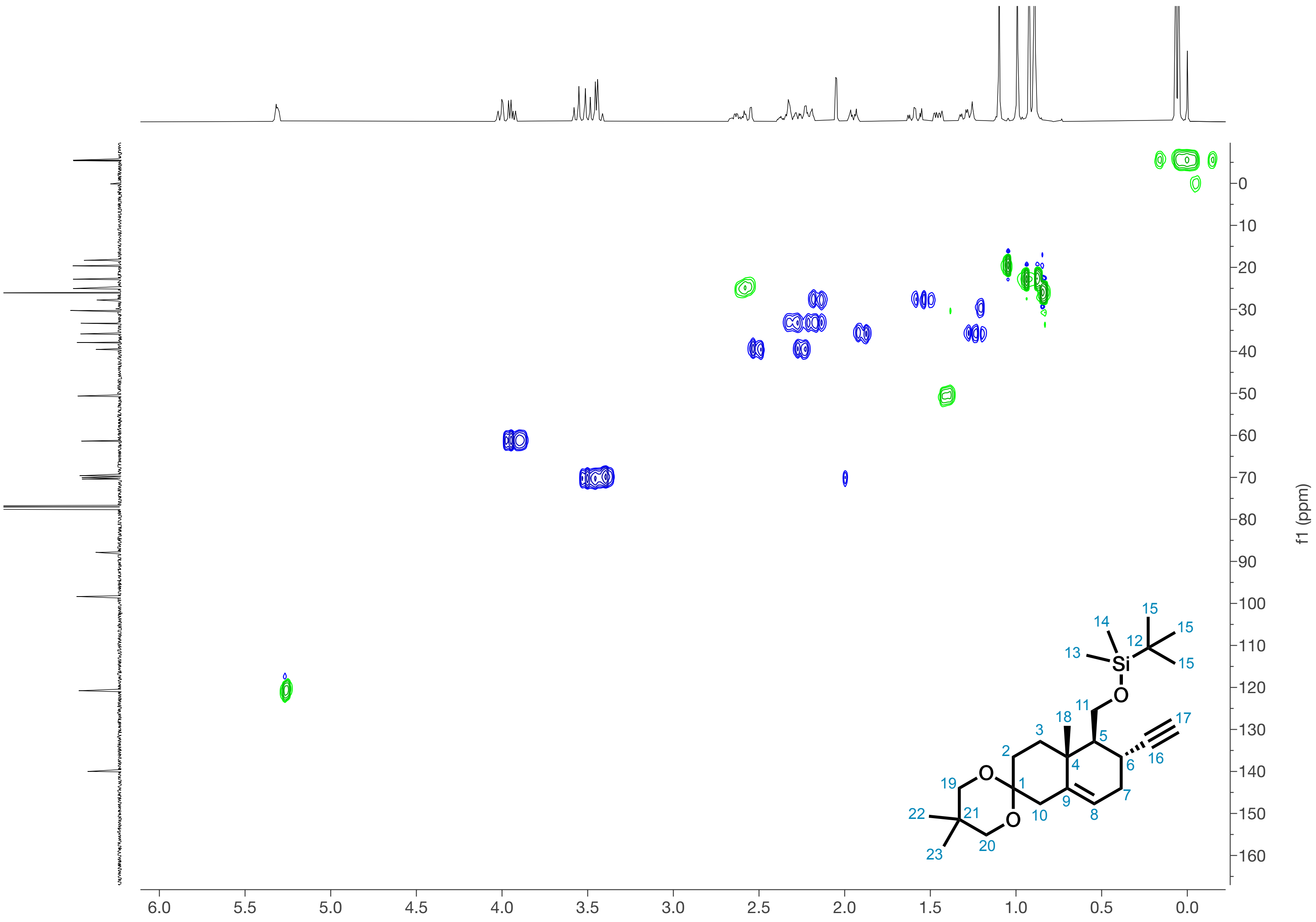

Figure S40. HSQC (400 MHz, $CDCl_3$) of **neopentyl glycol 14**.

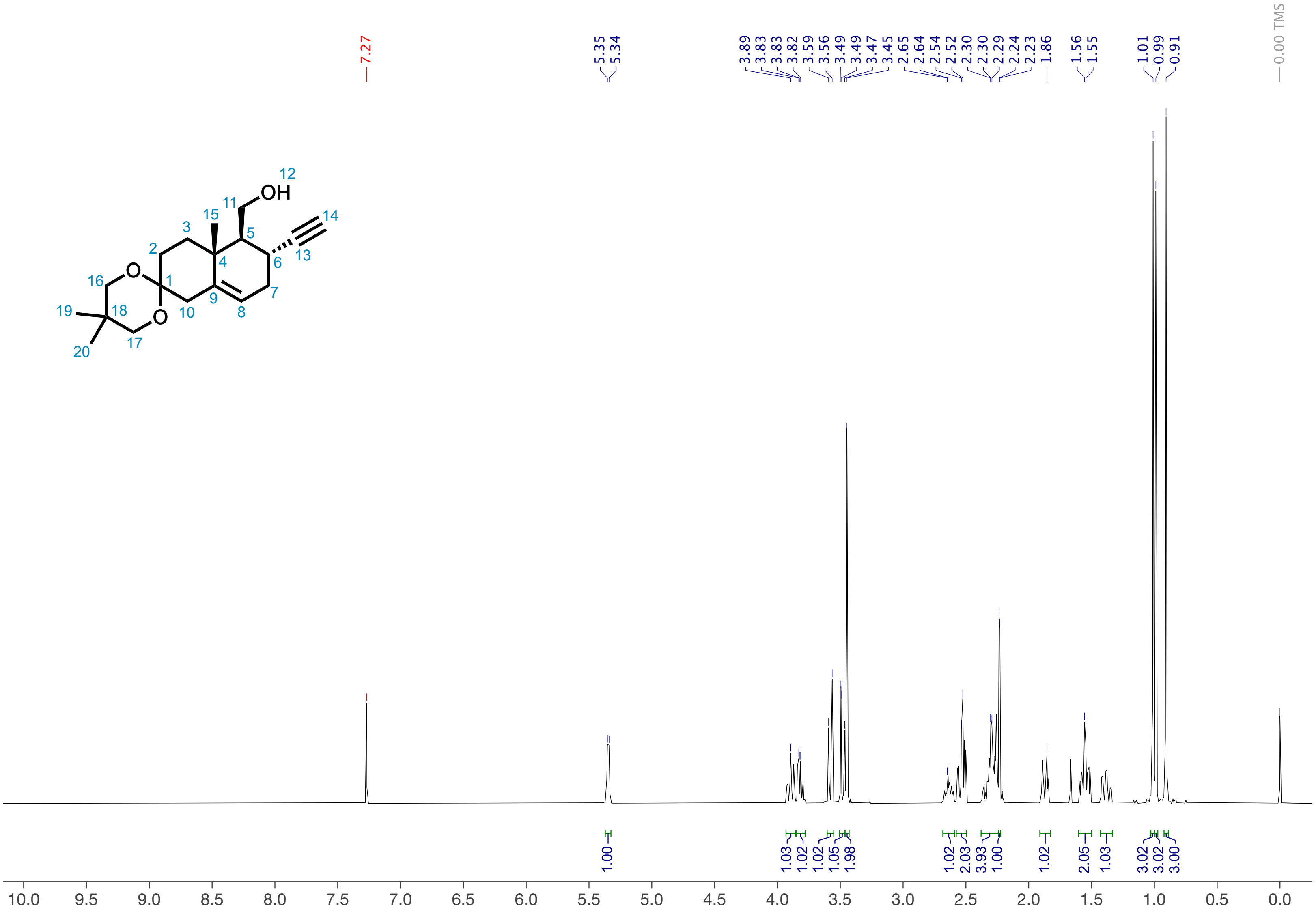

Figure S41. ^{1}H NMR (400 MHz, $CDCl_3$) of **primary alcohol 15**.

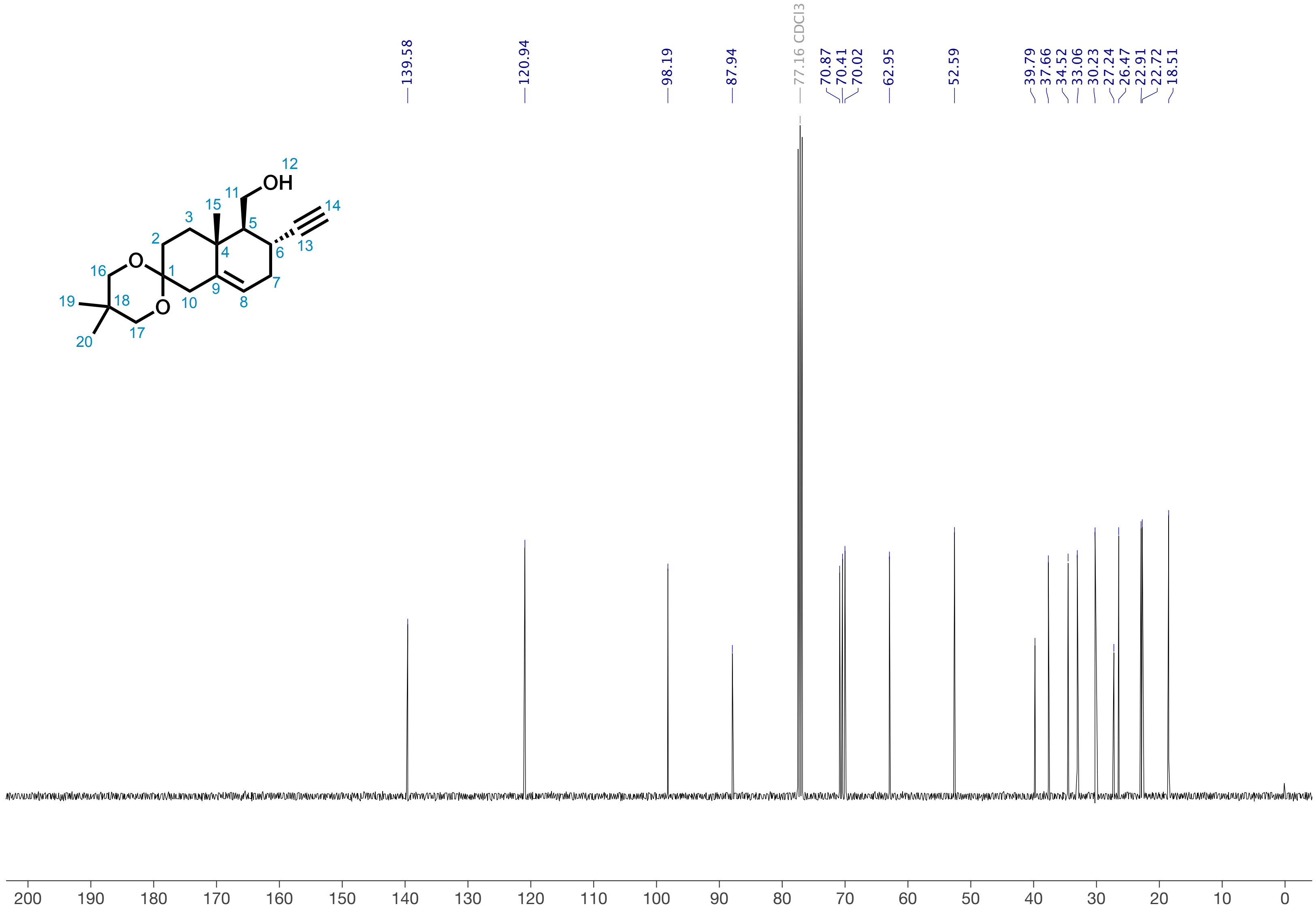

Figure S42. ^{13}C NMR (101 MHz, $CDCl_3$) of **primary alcohol 15**.

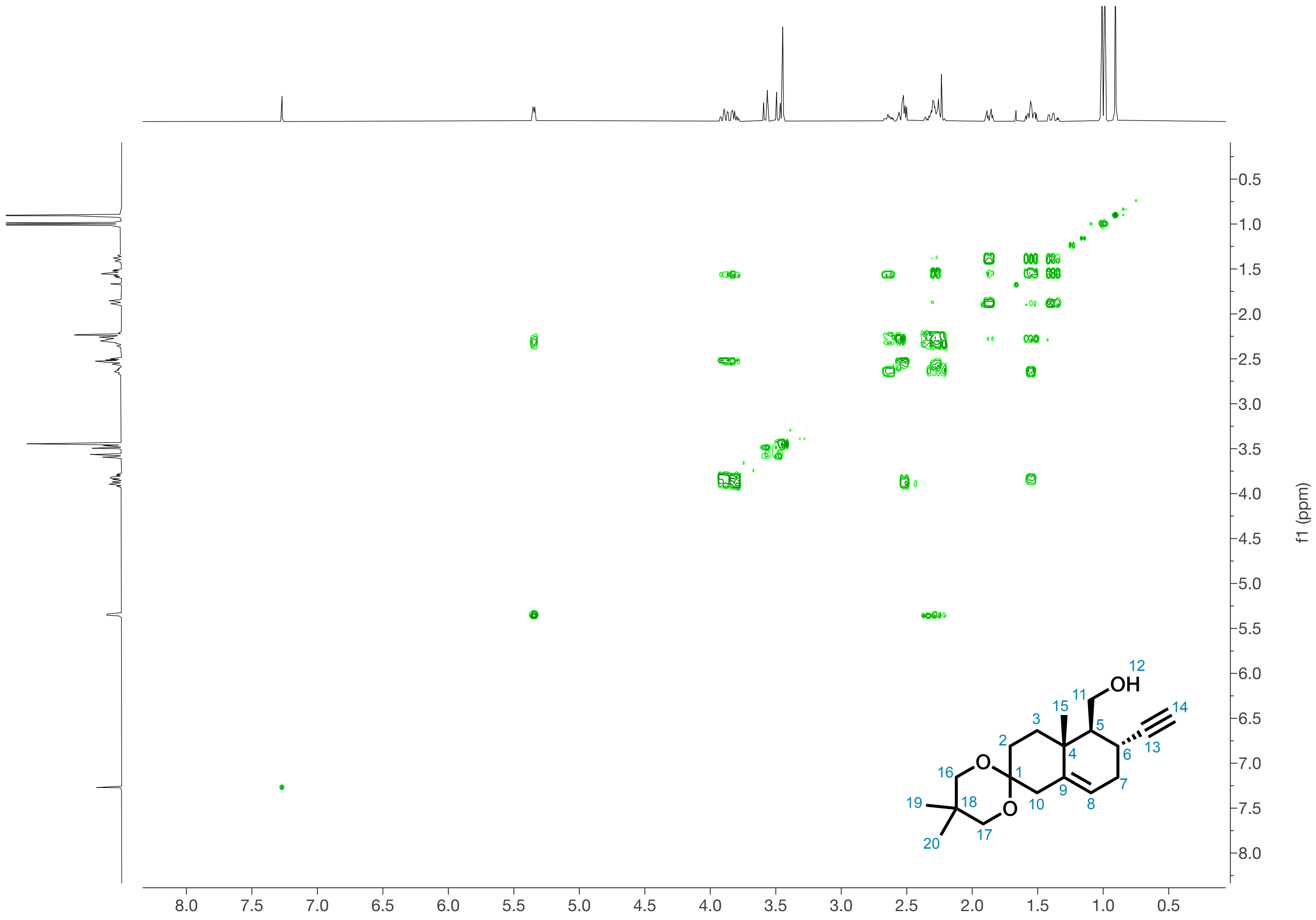

Figure S43. ^{1}H–^{1}H COSY (400 MHz, $CDCl_3$) of **primary alcohol 15**.

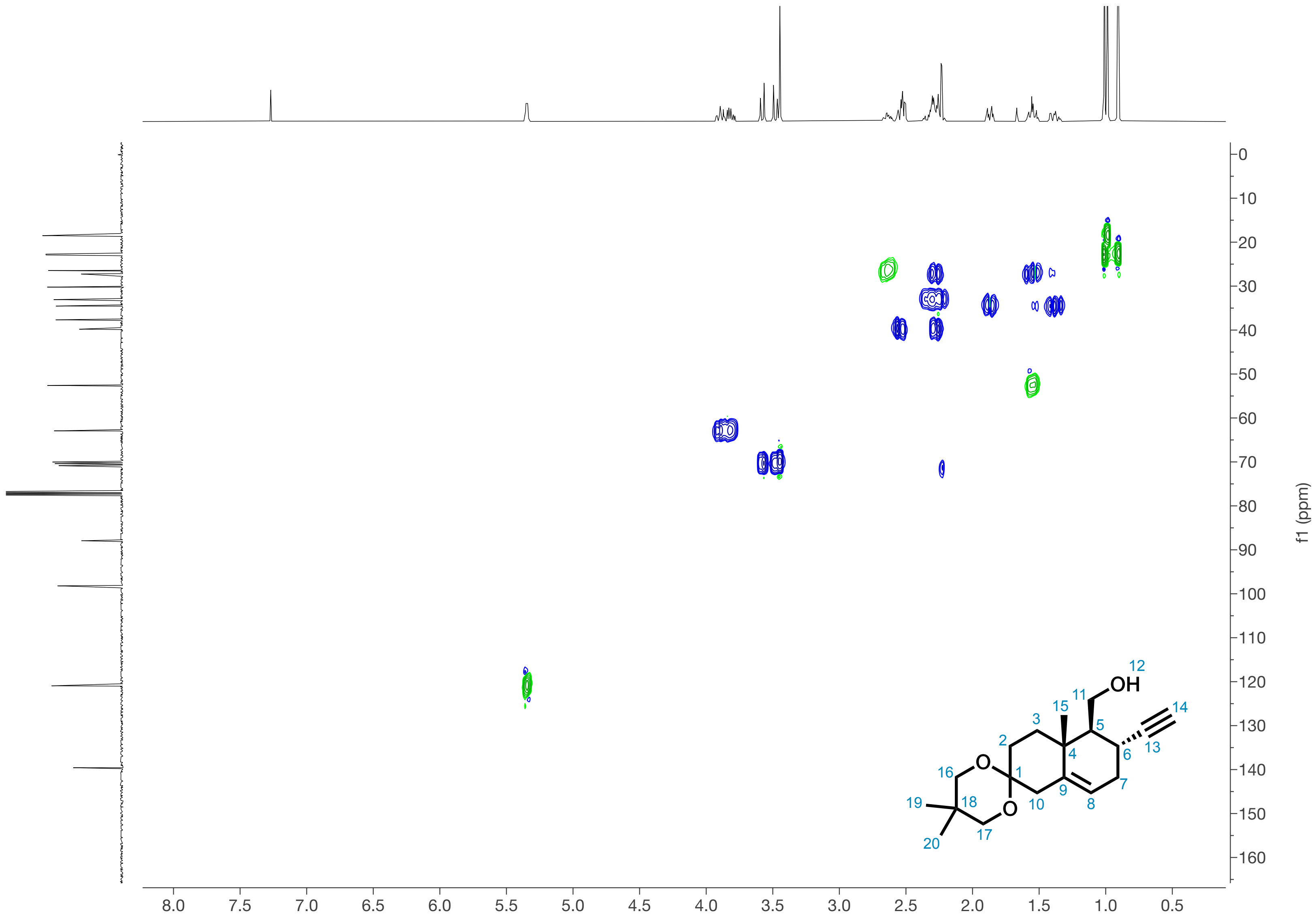

Figure S44. HSQC (400 MHz, $CDCl_3$) of **primary alcohol 15**.

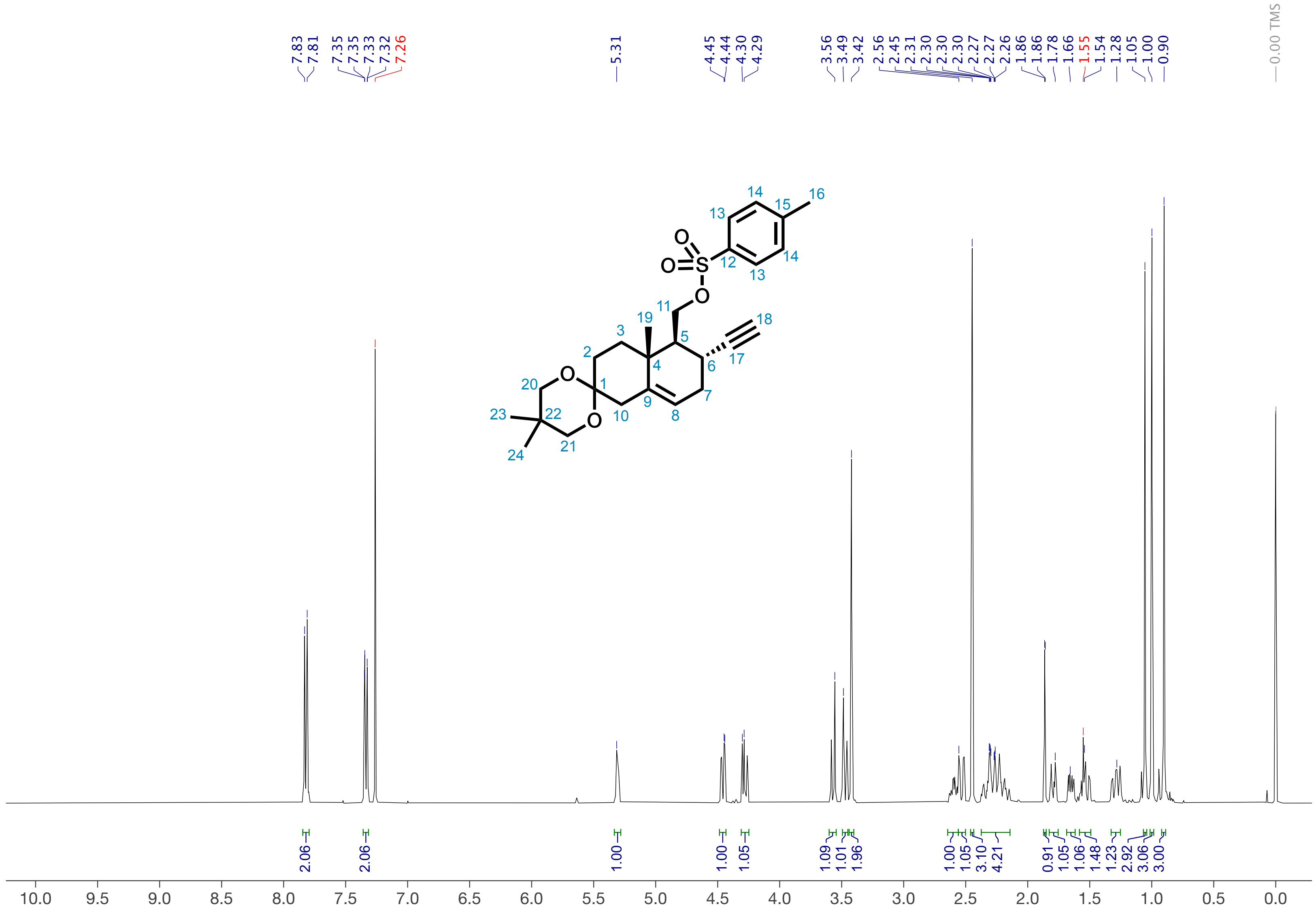

Figure S45. ^{1}H NMR (400 MHz, $CDCl_3$) of **tosylate 16**.

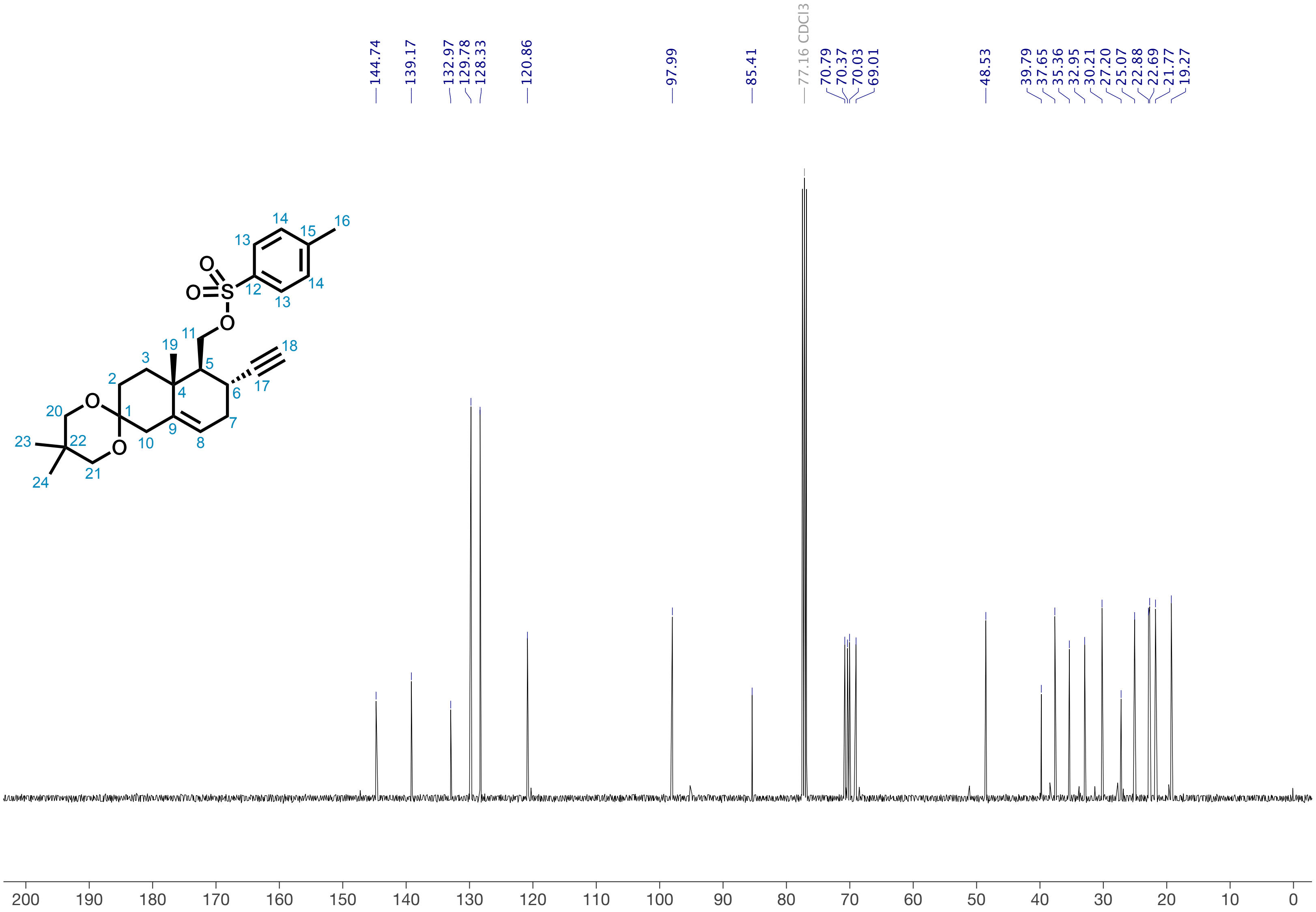

Figure S46. ^{13}C NMR (101 MHz, $CDCl_3$) of **tosylate 16**.

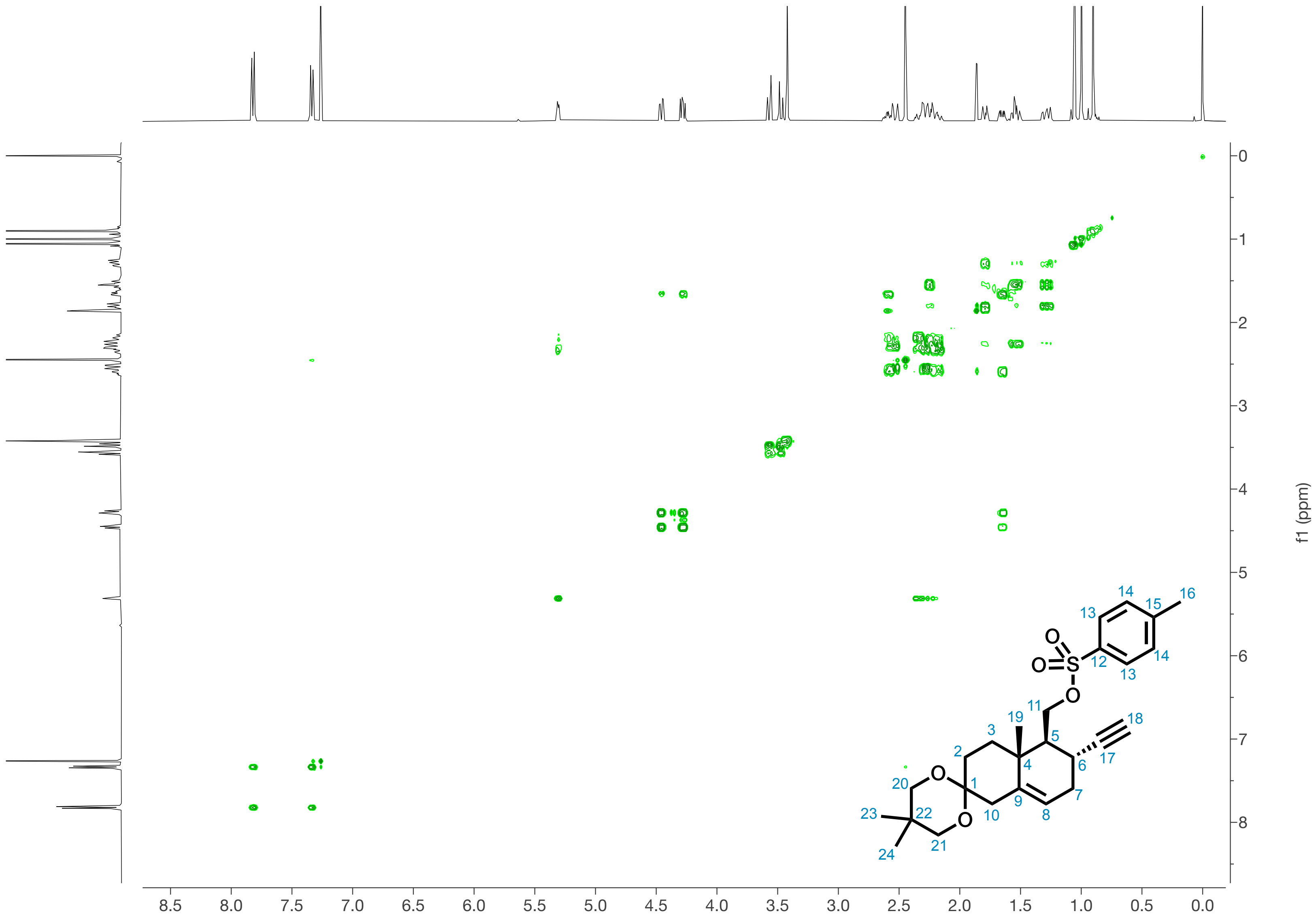

Figure S47. ^{1}H–^{1}H COSY (400 MHz, $CDCl_3$) of **tosylate 16**.

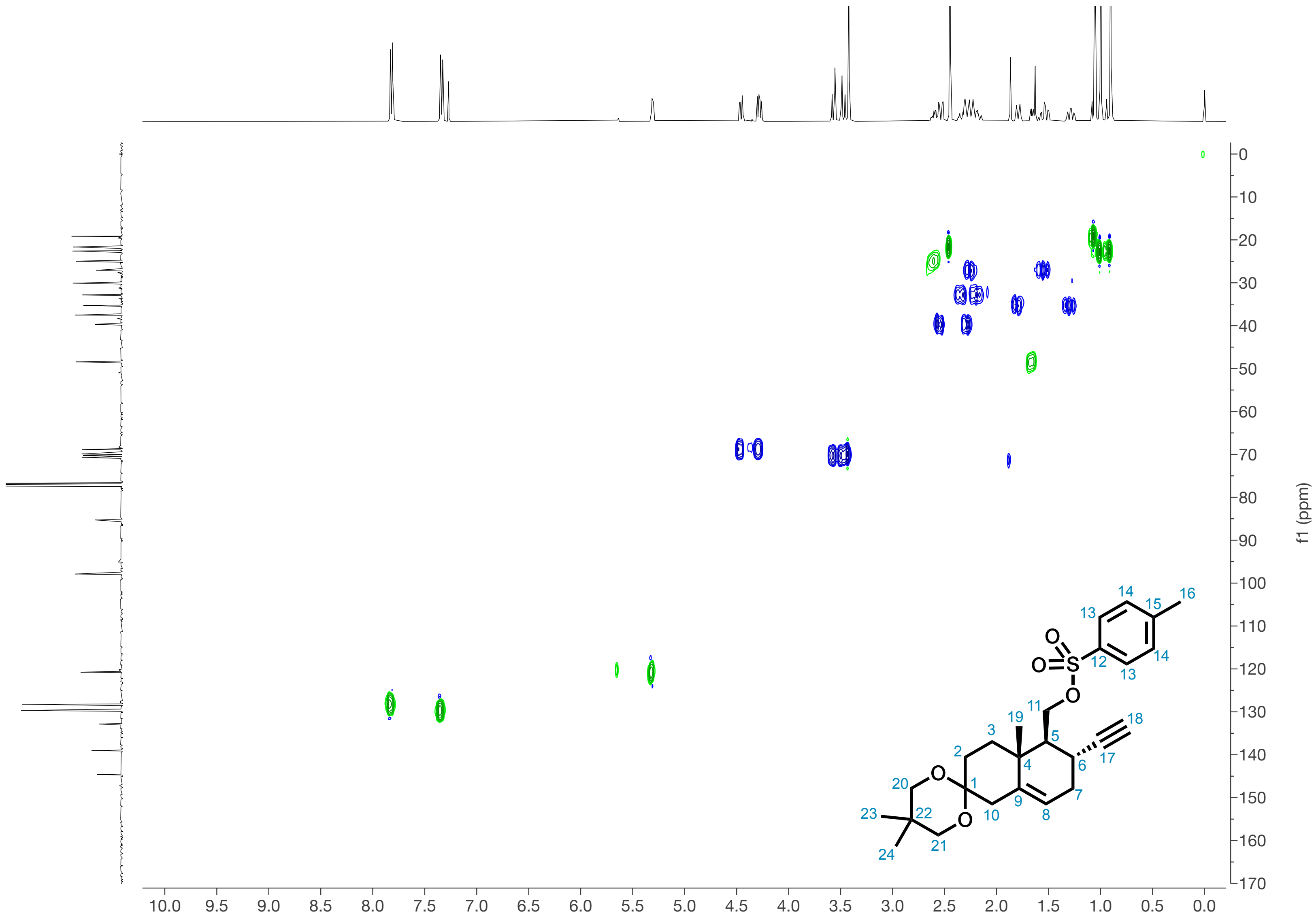

Figure S48. HSQC (400 MHz, $CDCl_3$) of **tosylate 16**.

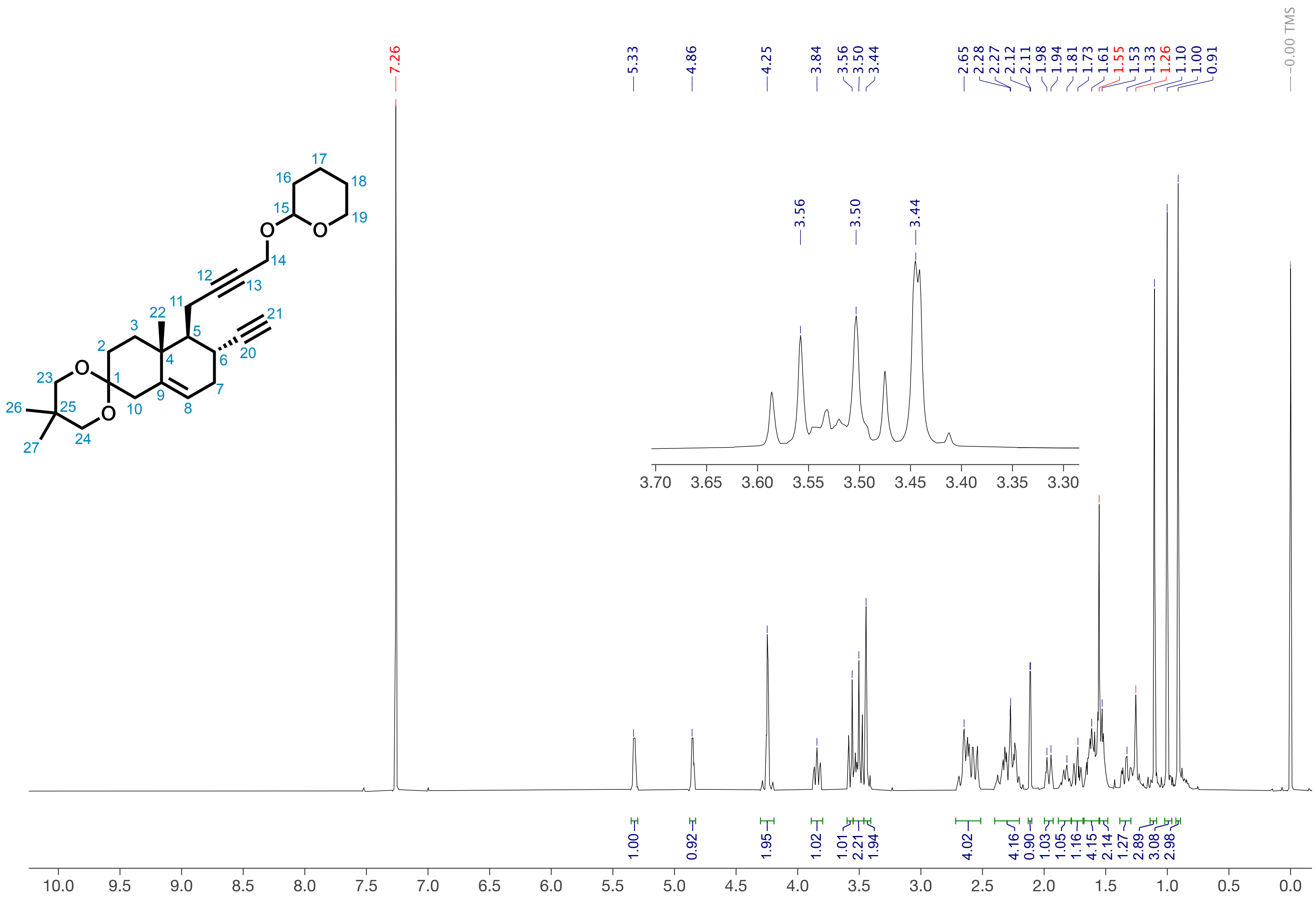

Figure S49. ^{1}H NMR (400 MHz, $CDCl_3$) of **diyne SI-8**.

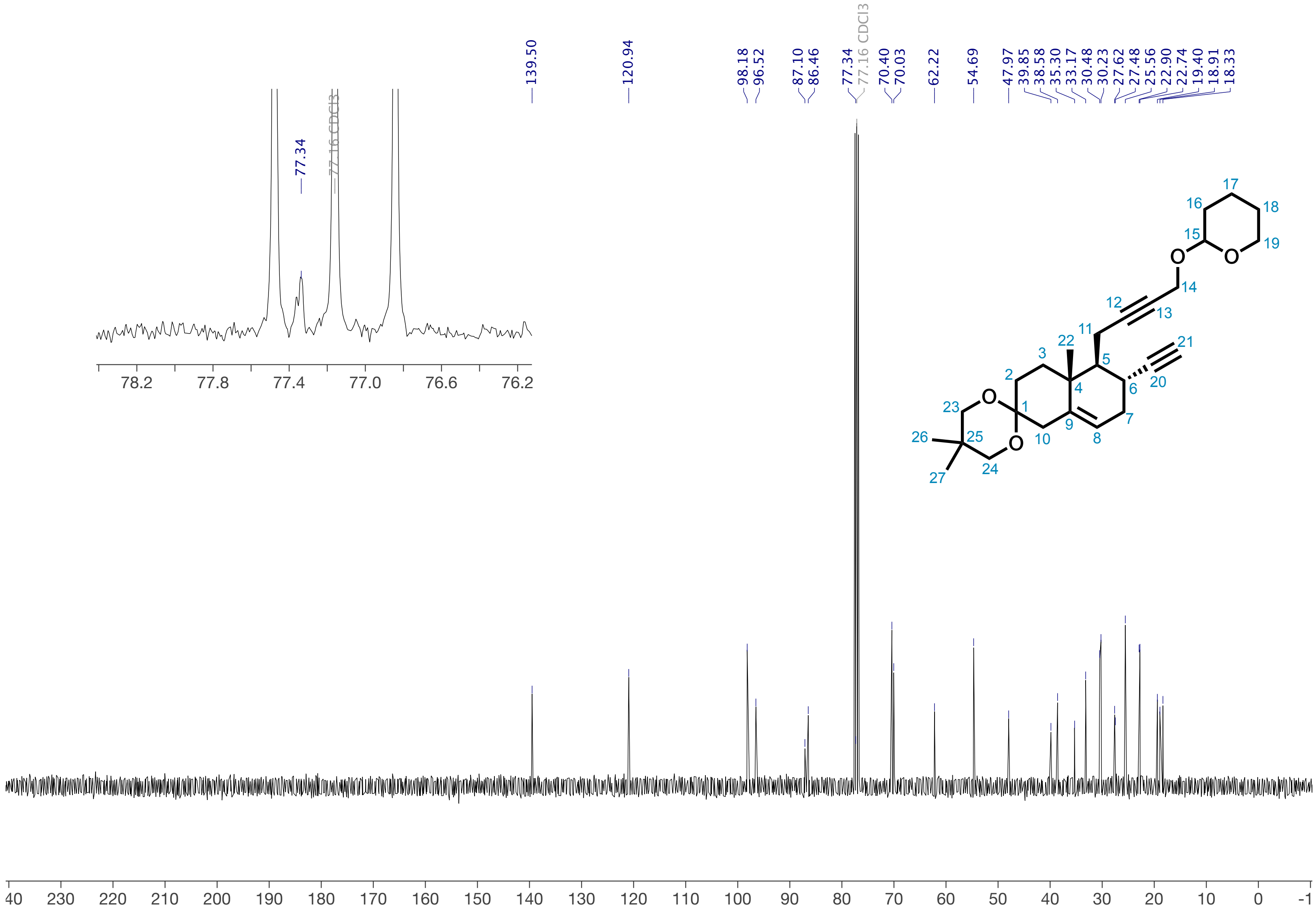

Figure S50. ^{13}C NMR (101 MHz, $CDCl_3$) of **diyne SI-8.**

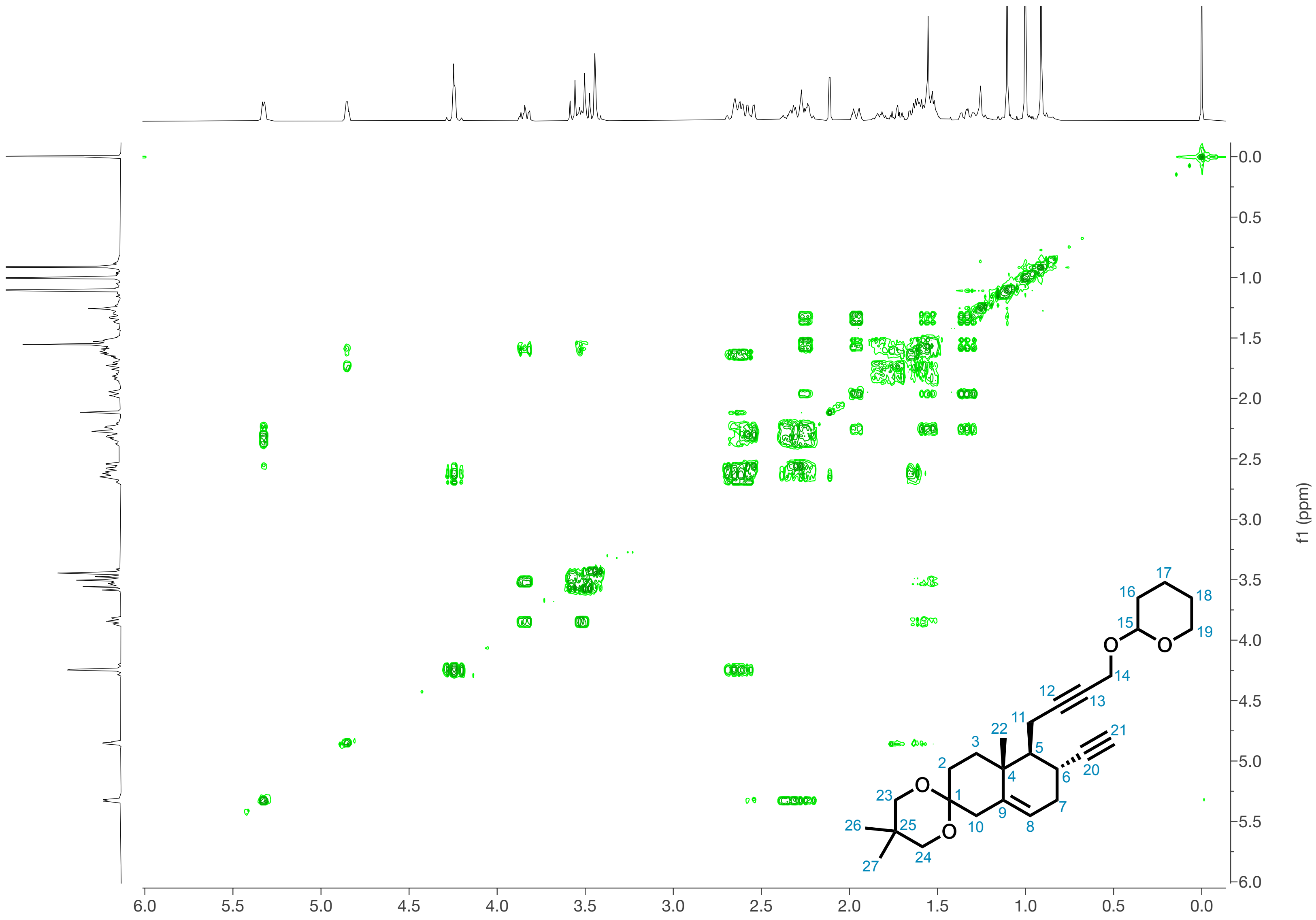

Figure S51. ^{1}H–^{1}H COSY (400 MHz, $CDCl_3$) of **diyne SI-8**.

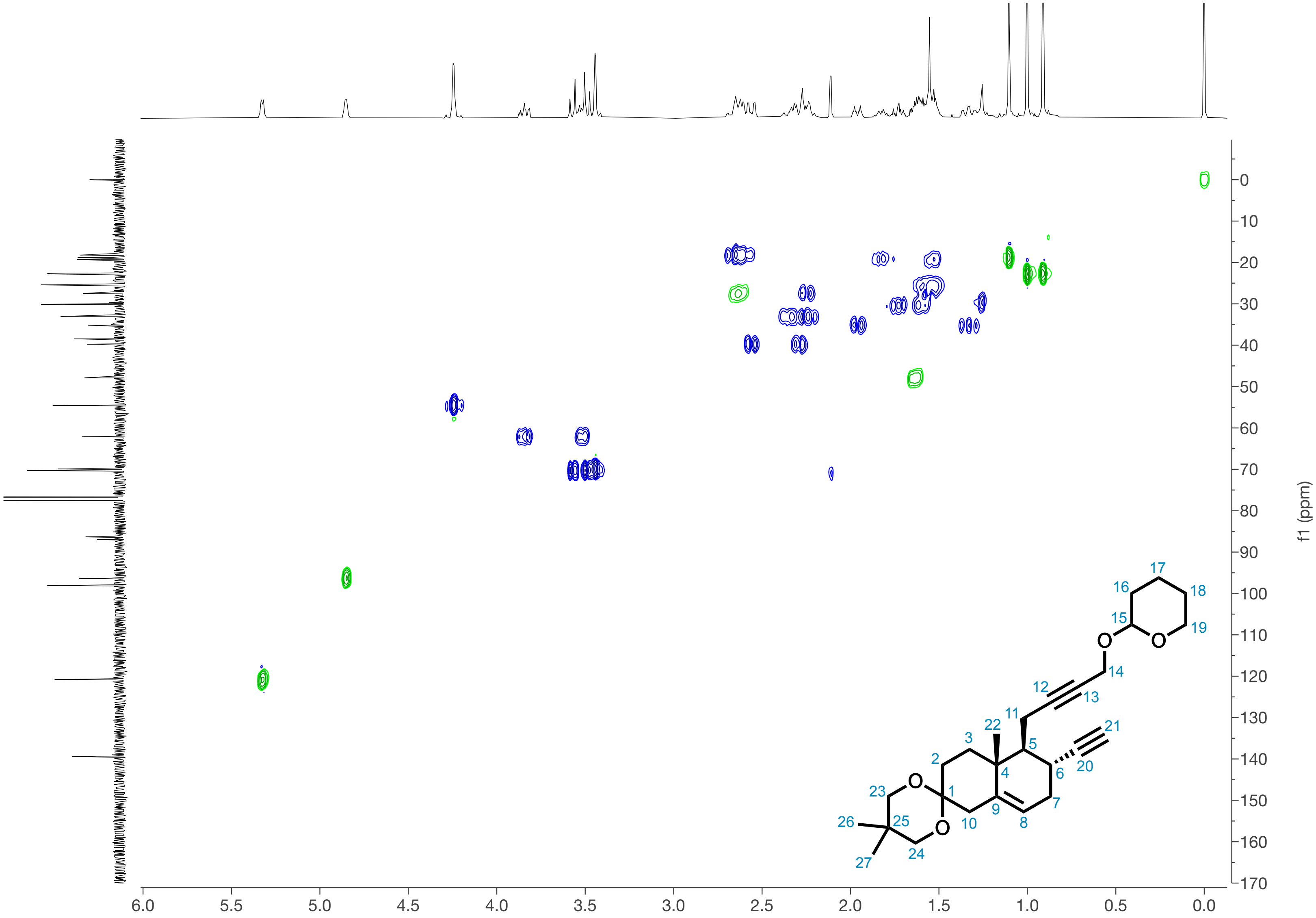

Figure S52. HSQC (400 MHz, $CDCl_3$) of **diyne SI-8**.

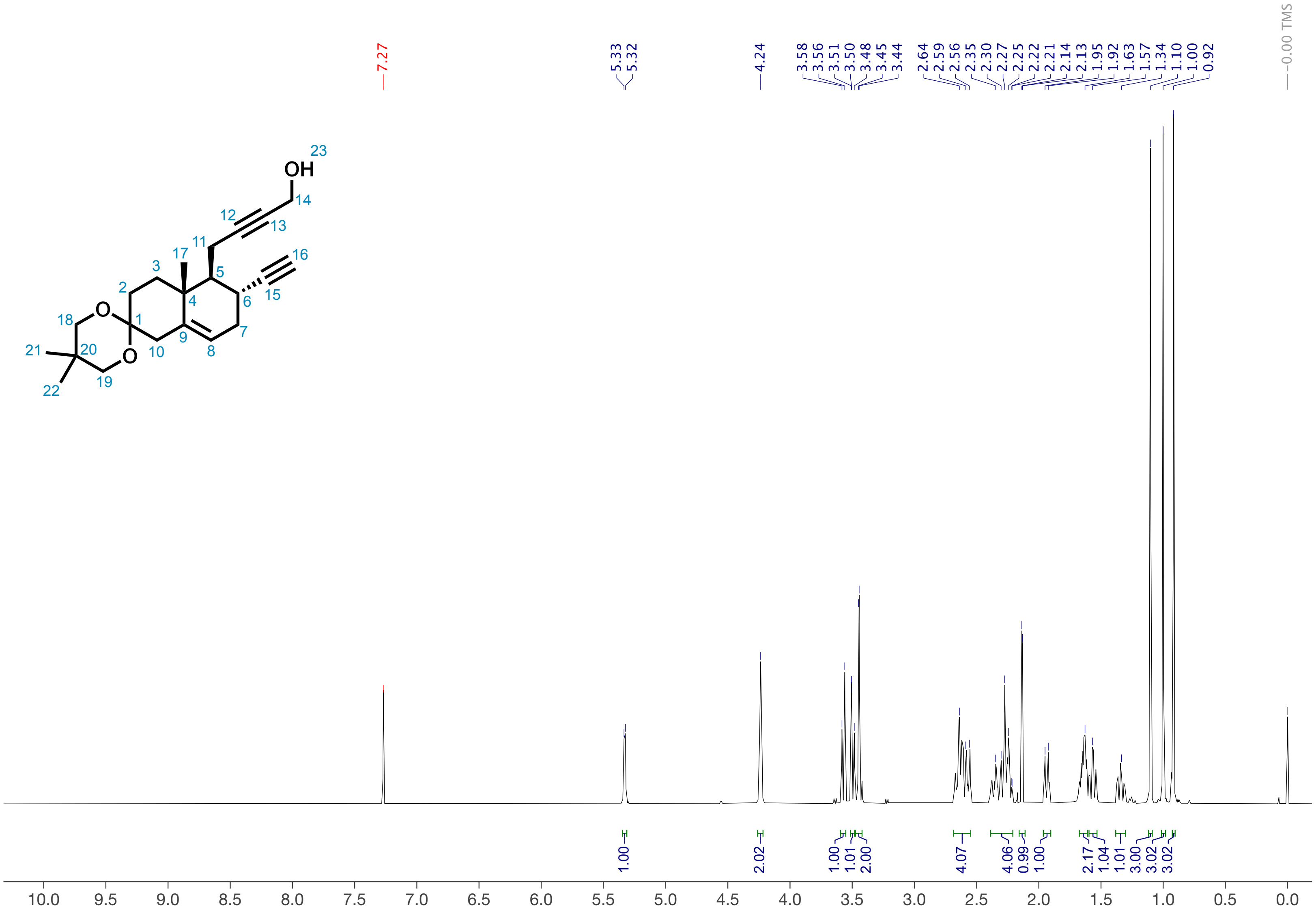

Figure S53. ^{1}H NMR (500 MHz, $CDCl_3$) of **ketalized propargyl alcohol SI-9**.

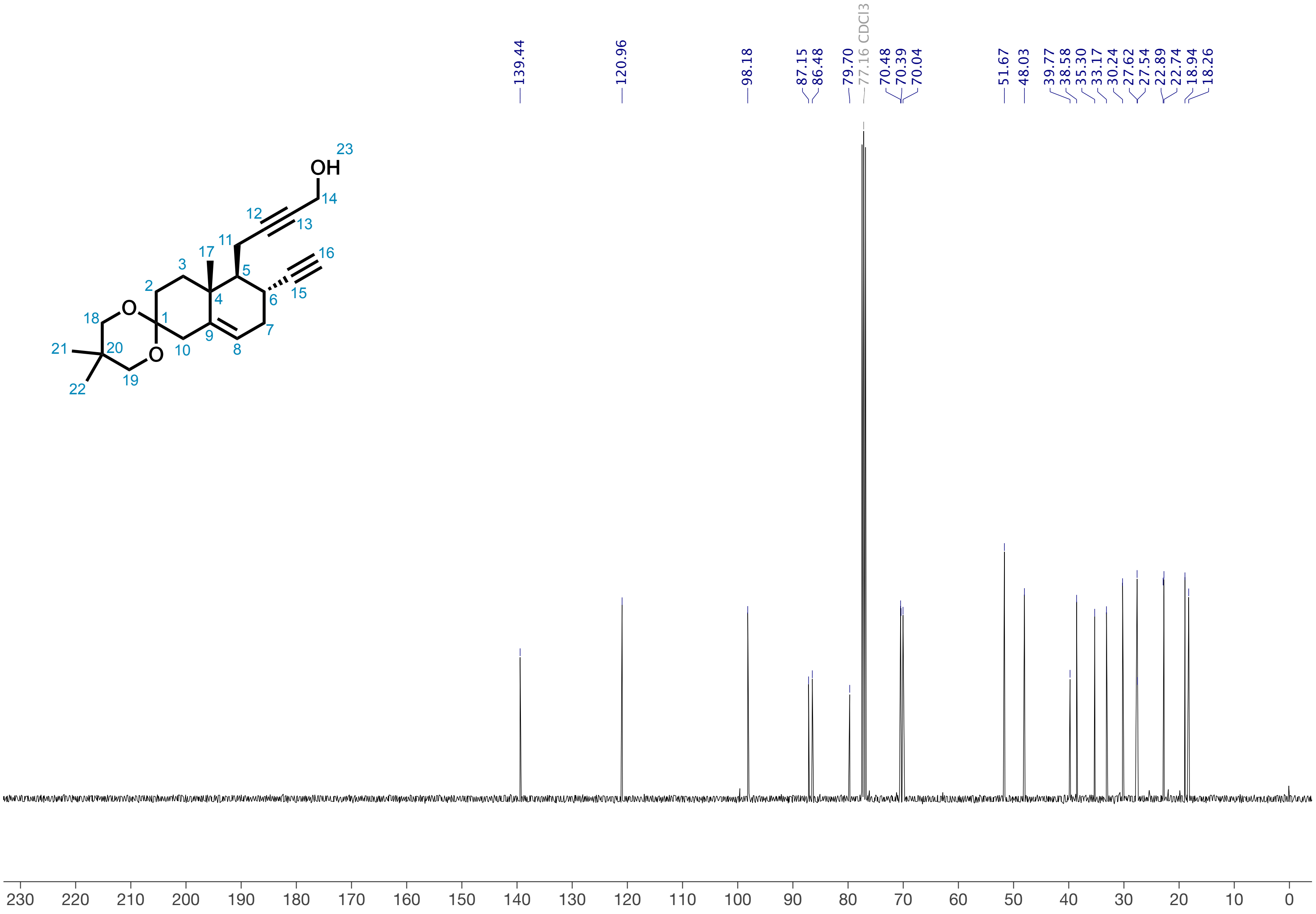

Figure S54. ^{13}C NMR (101 MHz, $CDCl_3$) of **ketalized propargyl alcohol SI-9**.

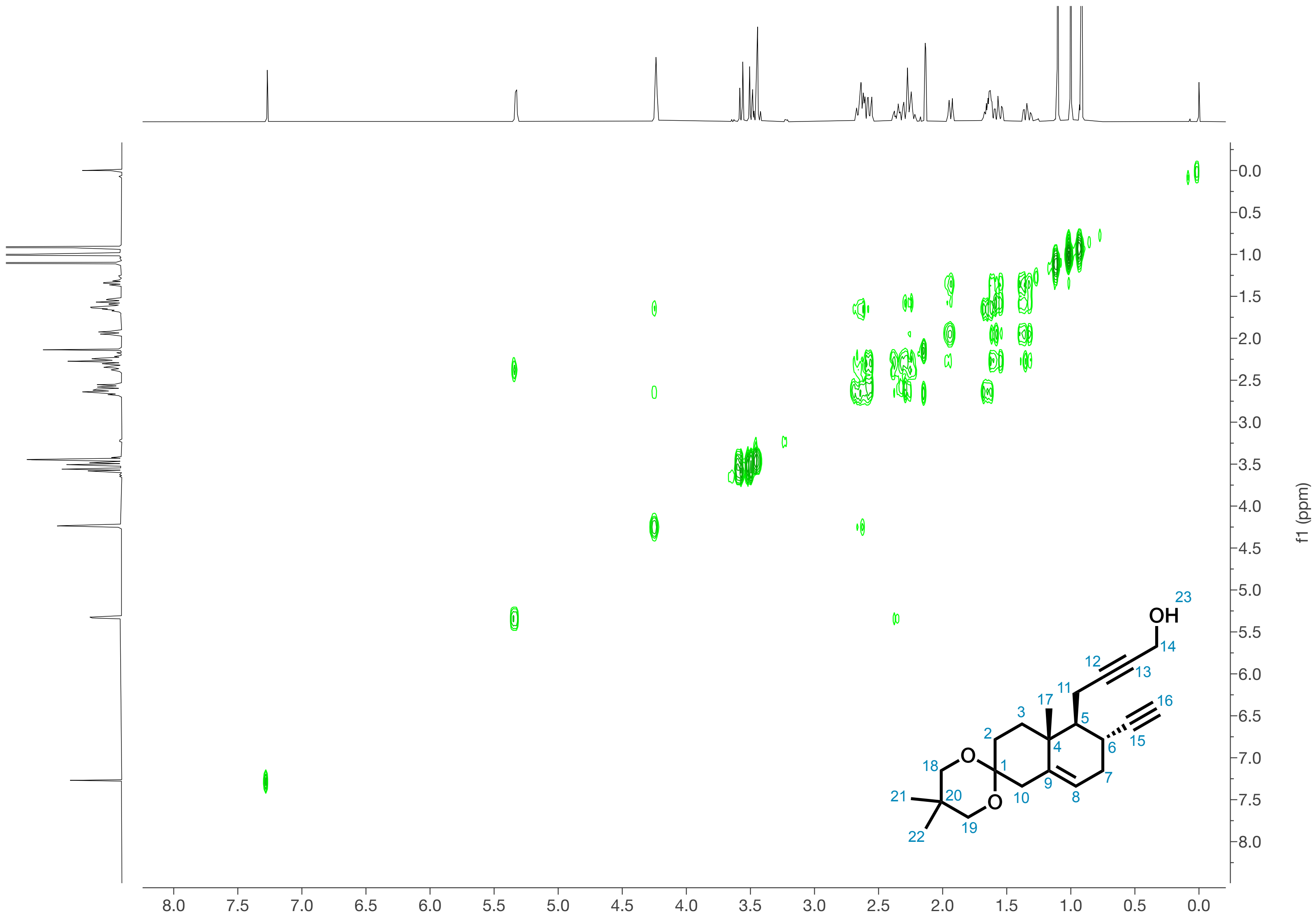

Figure S55. ^{1}H–^{1}H COSY (400 MHz, $CDCl_3$) of **ketalized propargyl alcohol SI-9**.

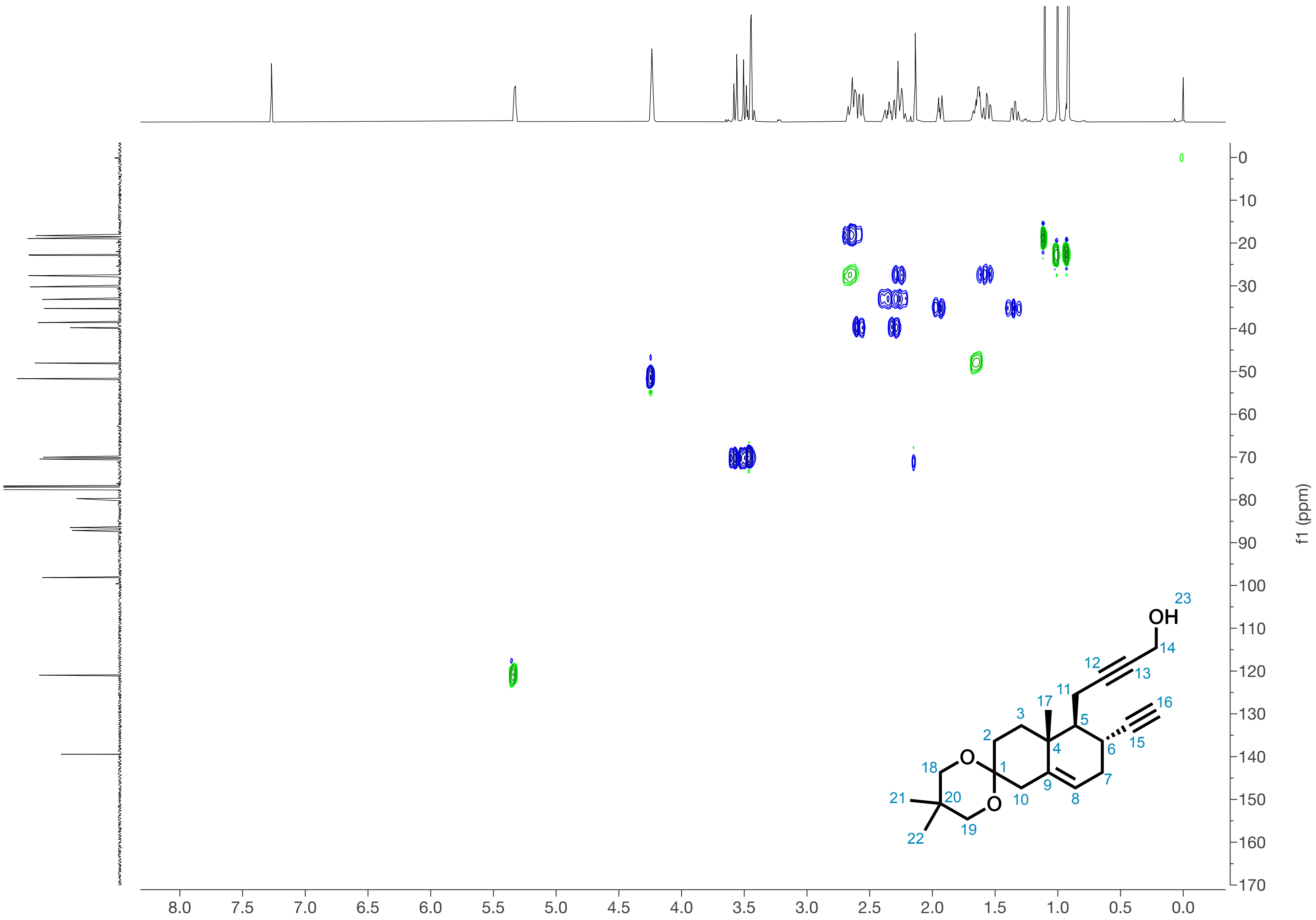

Figure S56. HSQC (400 MHz, $CDCl_3$) of **ketalized propargyl alcohol SI-9**.

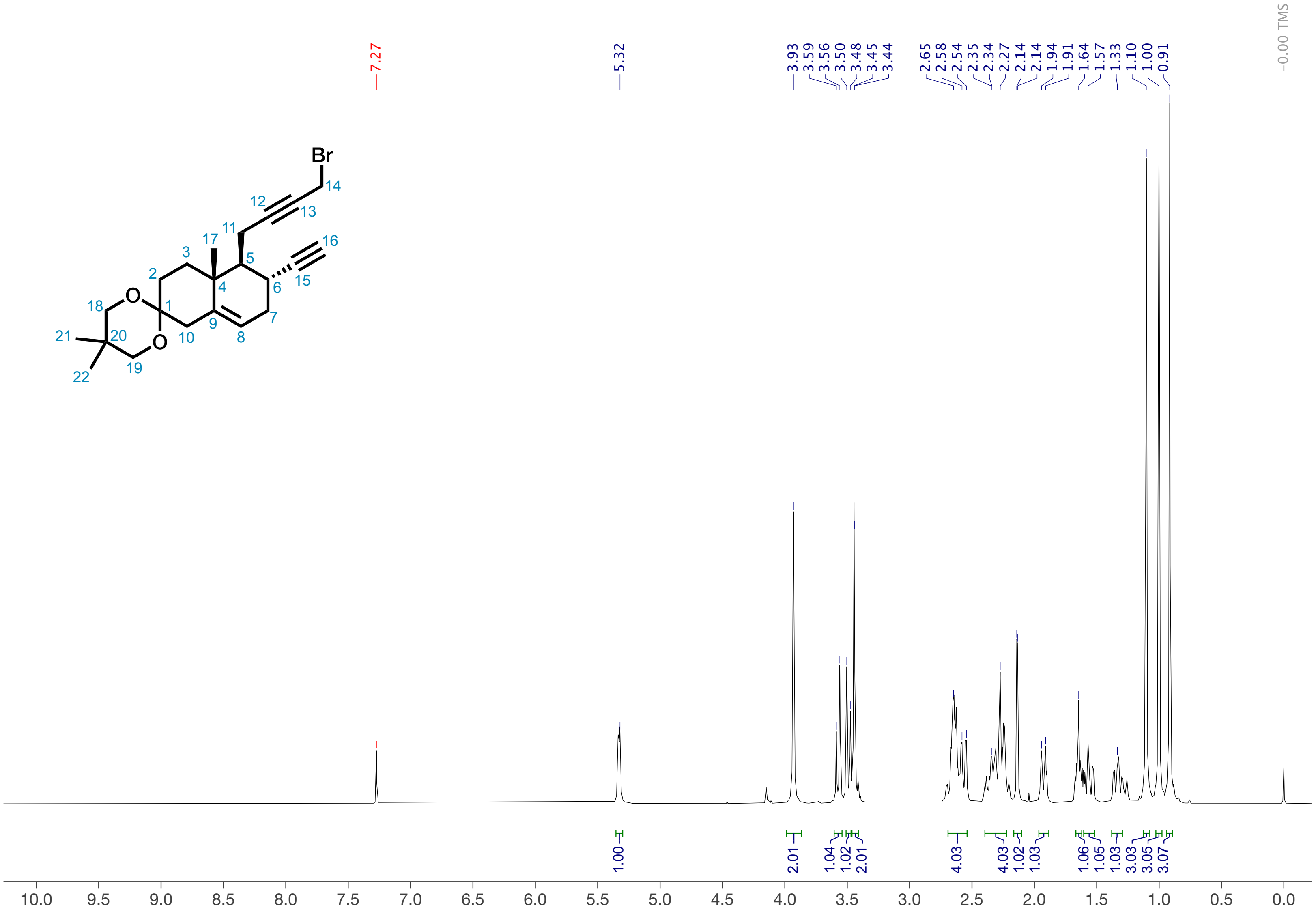

Figure S57. ^{1}H NMR (400 MHz, $CDCl_3$) of **ketalized propargyl bromide 17**.

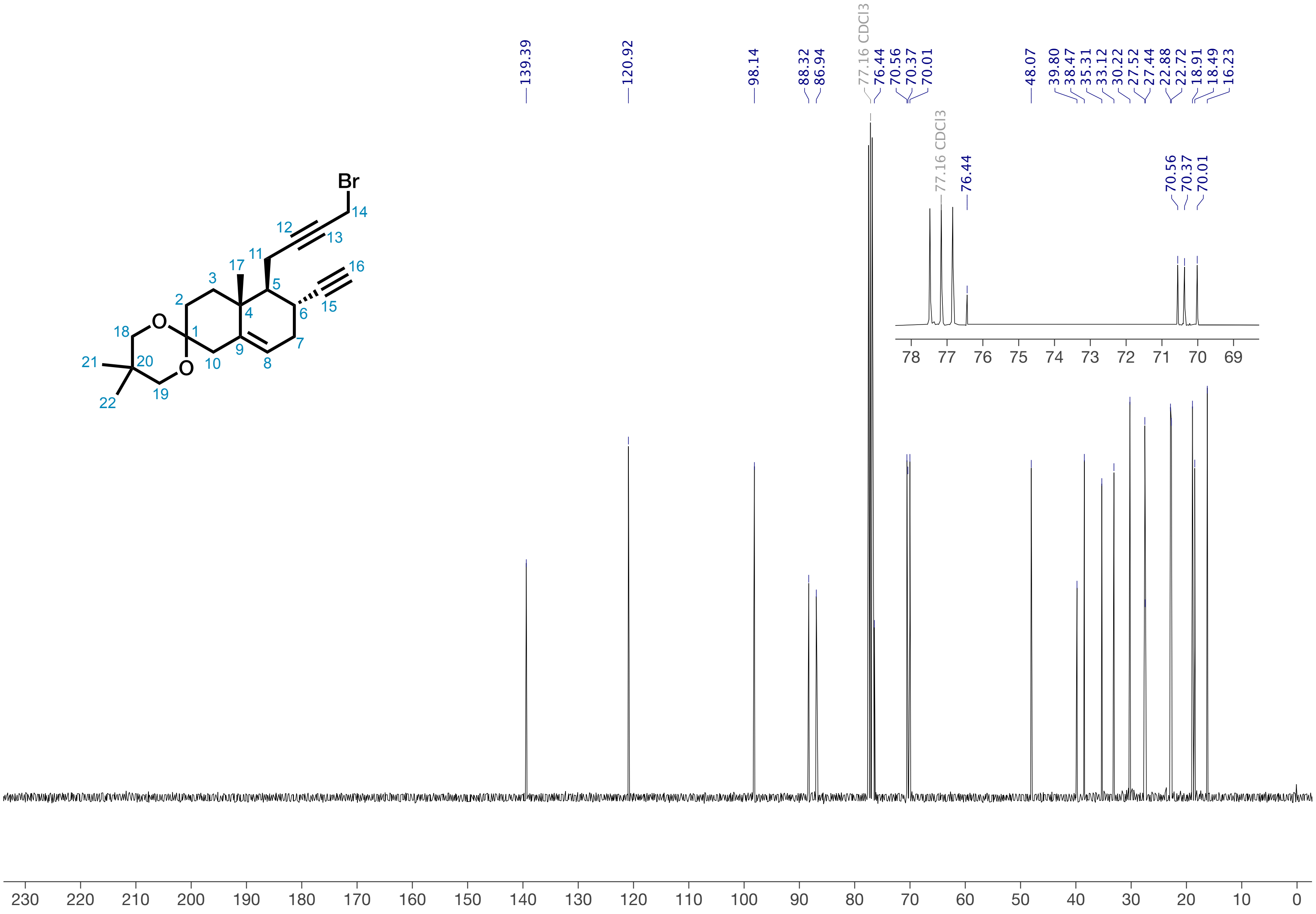

Figure S58. ^{13}C NMR (101 MHz, $CDCl_3$) of **ketalized propargyl bromide 17**.

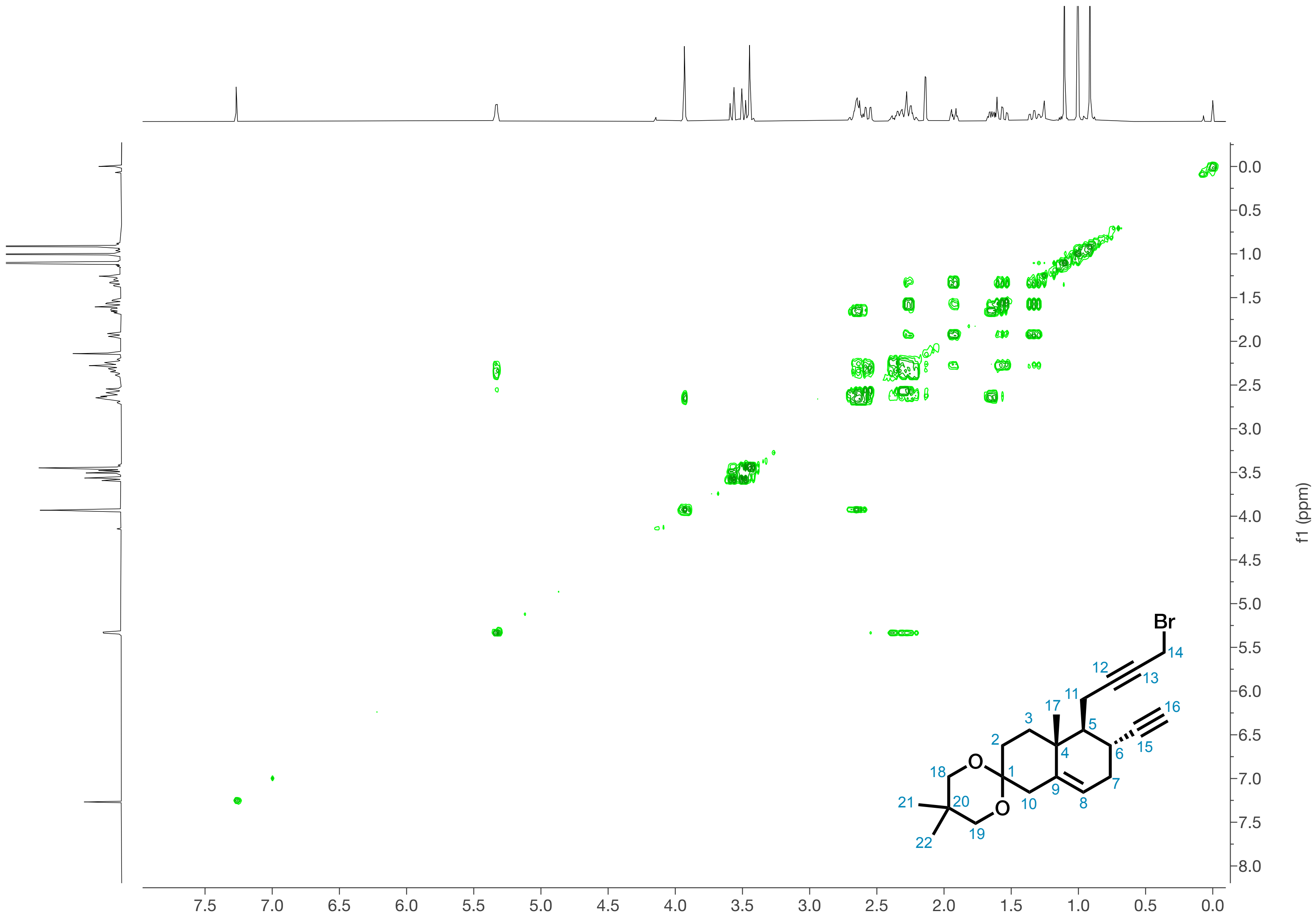

Figure S59. ^1H–^1H COSY (400 MHz, $CDCl_3$) of **ketalized propargyl bromide 17**.

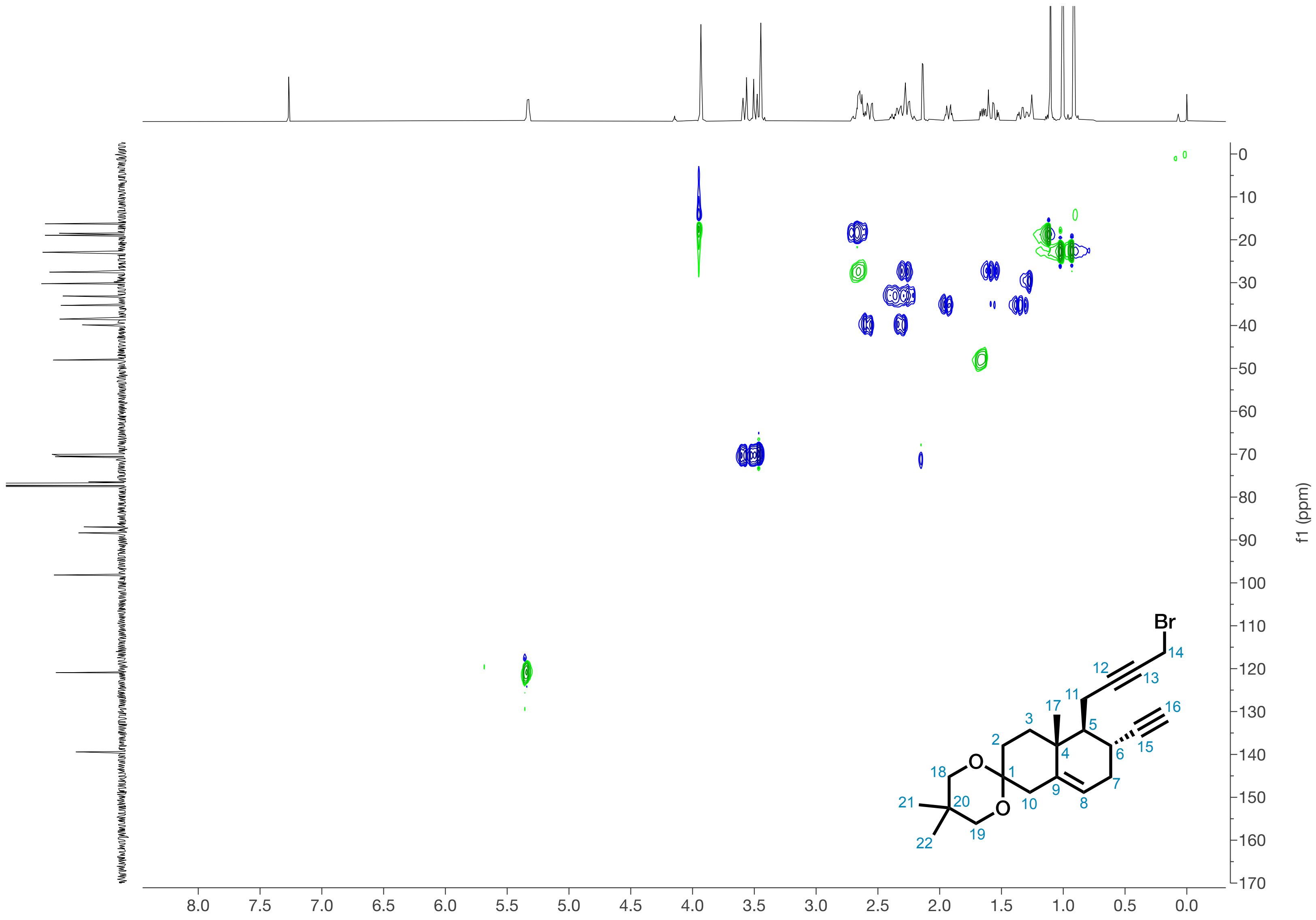

Figure S60. HSQC (400 MHz, $CDCl_3$) of **ketalized propargyl bromide 17**.

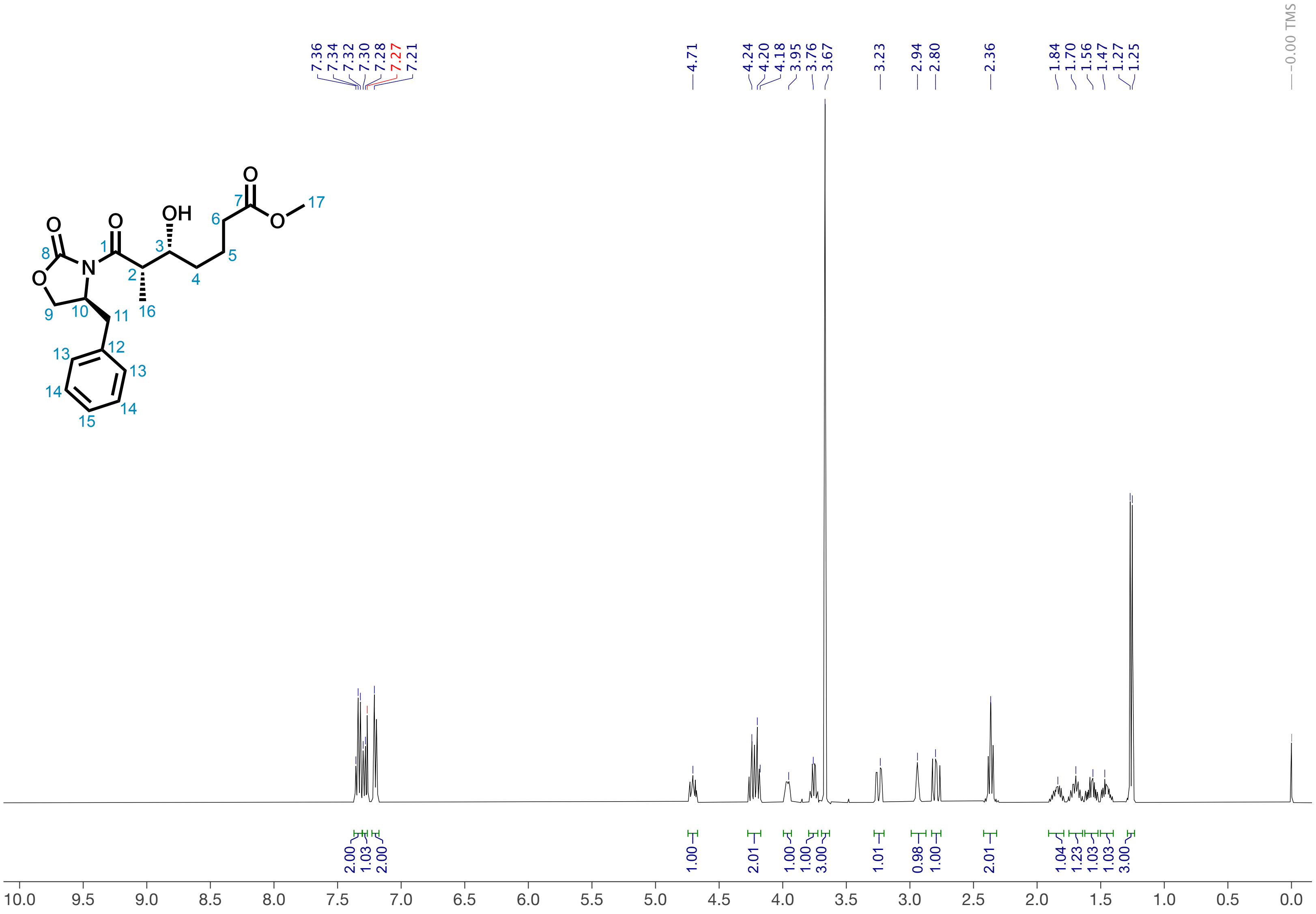

Figure S61. ^{1}H NMR (400 MHz, $CDCl_3$) of **alcohol 22**.

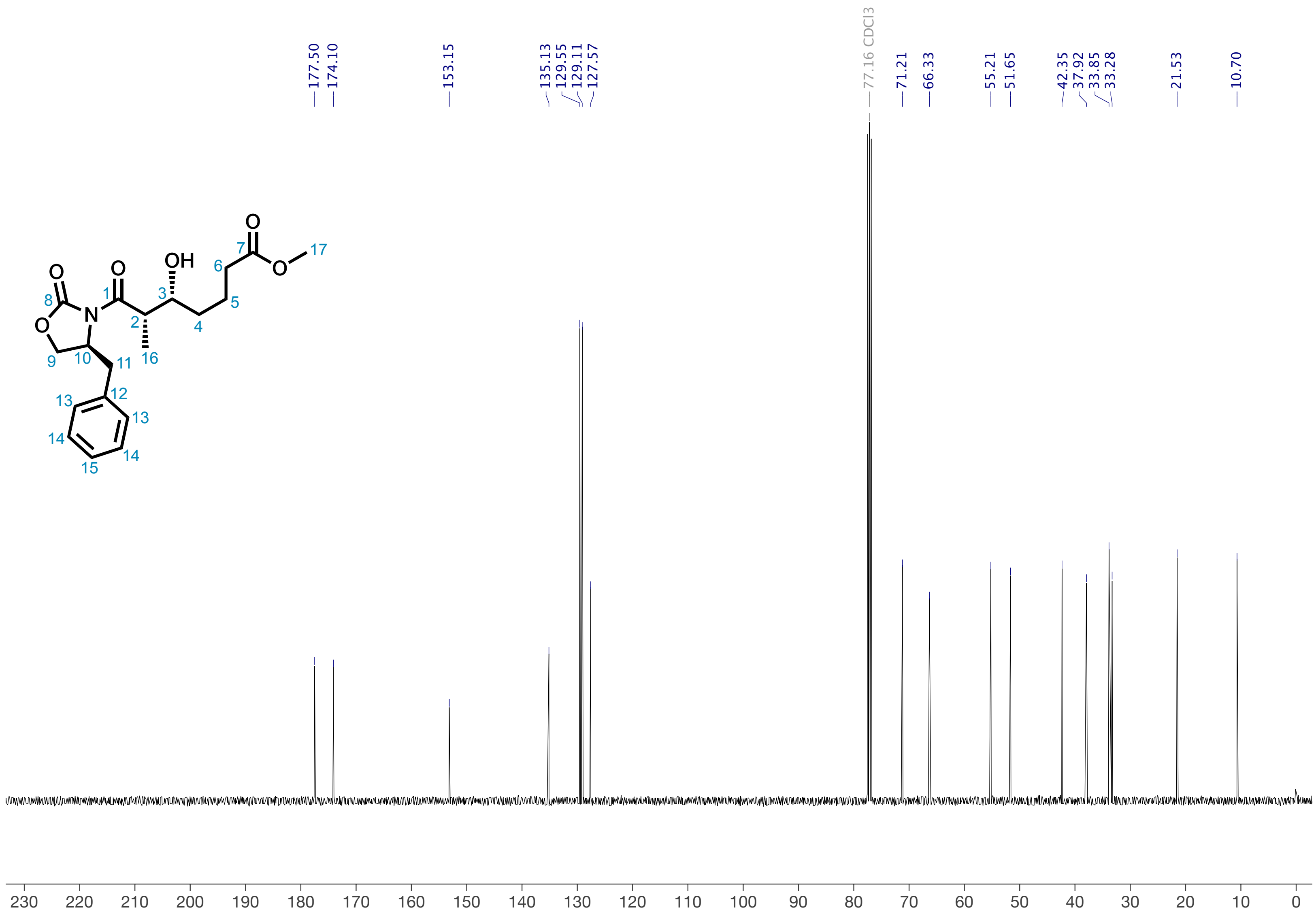

Figure S62. ^{13}C NMR (101 MHz, $CDCl_3$) of **alcohol 22**.

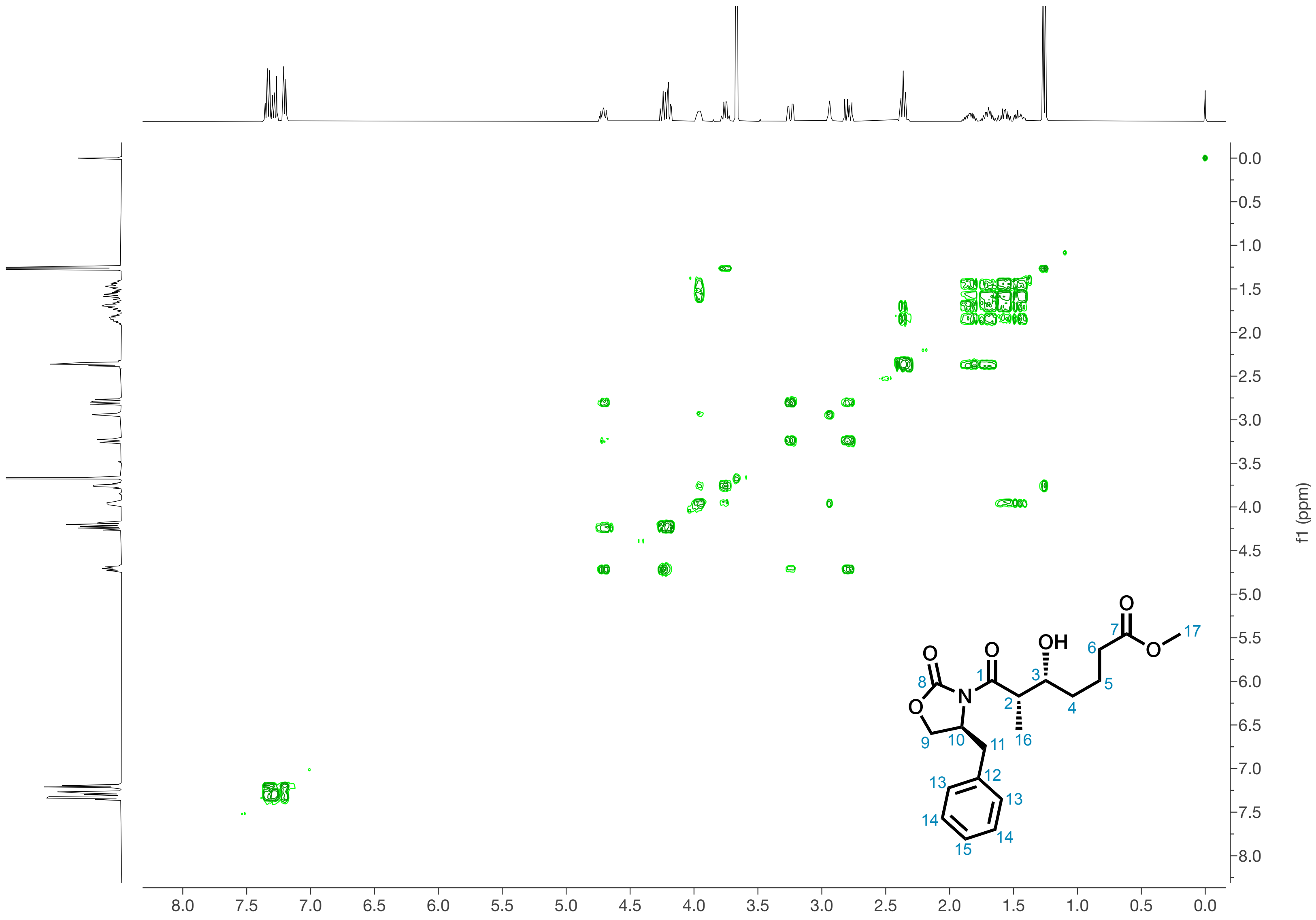

Figure S63. ^{1}H–^{1}H COSY (400 MHz, $CDCl_3$) of **alcohol 22**.

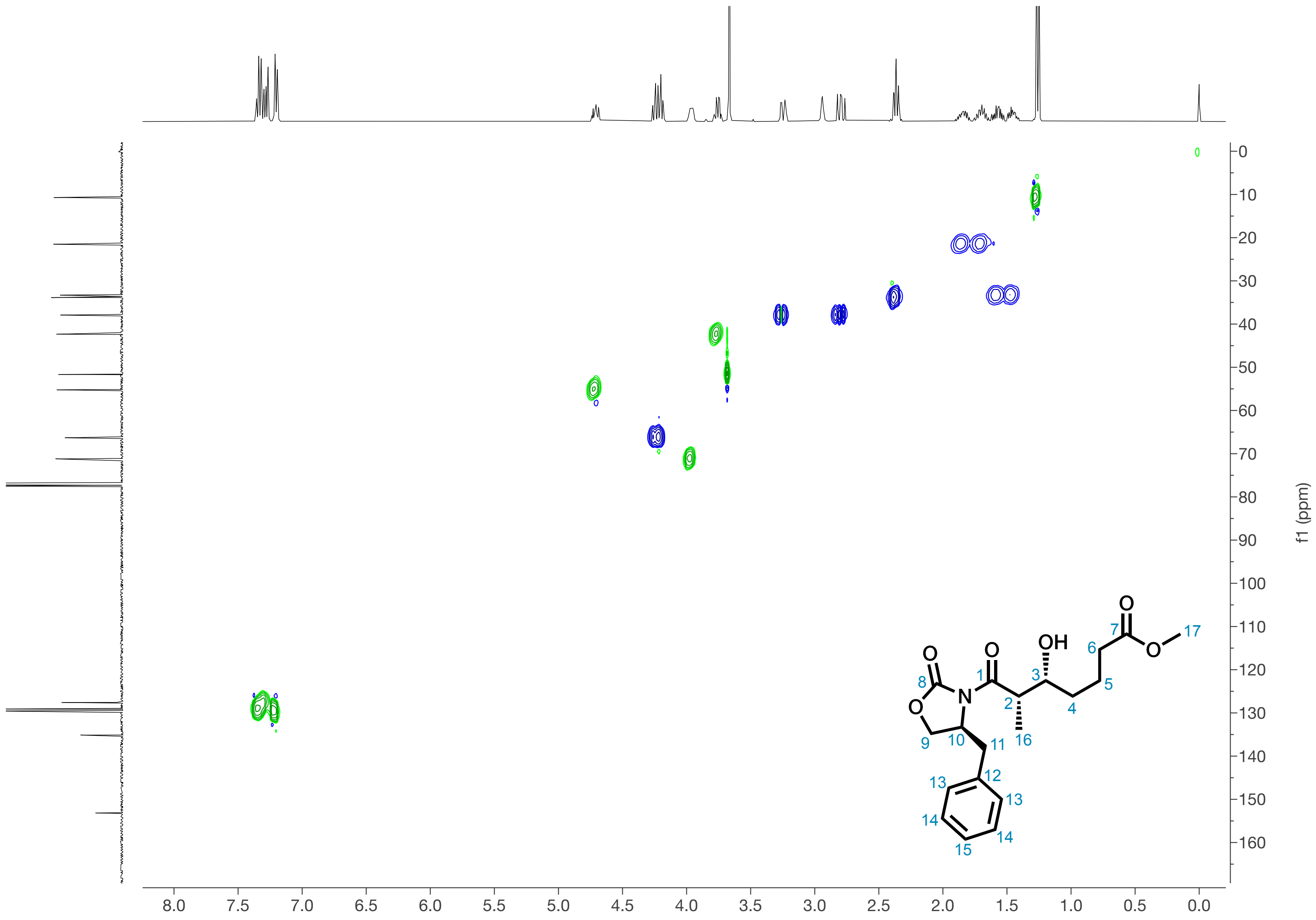

Figure S64. HSQC (400 MHz, $CDCl_3$) of **alcohol 22**.

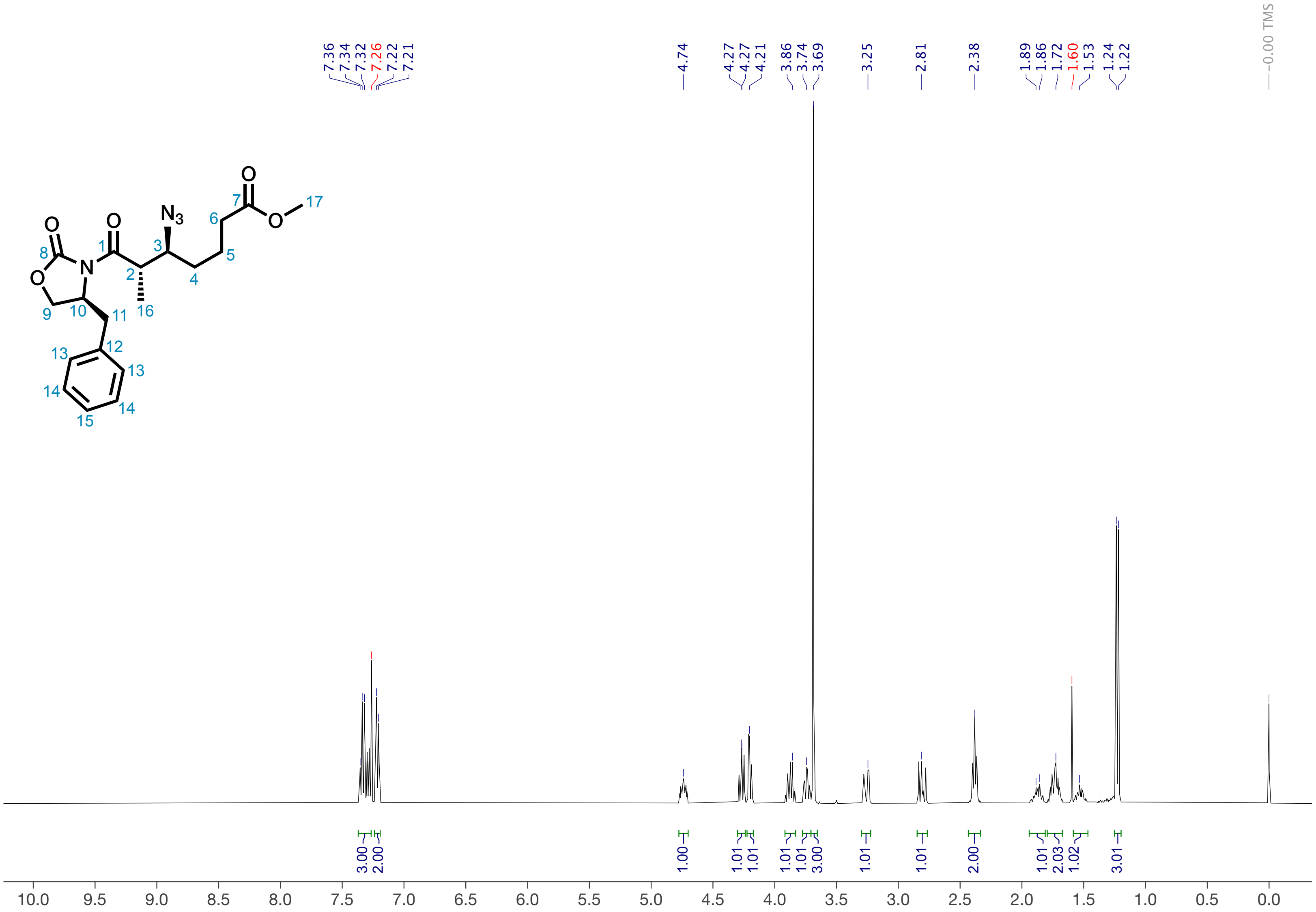

Figure S65. ^{1}H NMR (400 MHz, $CDCl_3$) of **azide SI-11**.

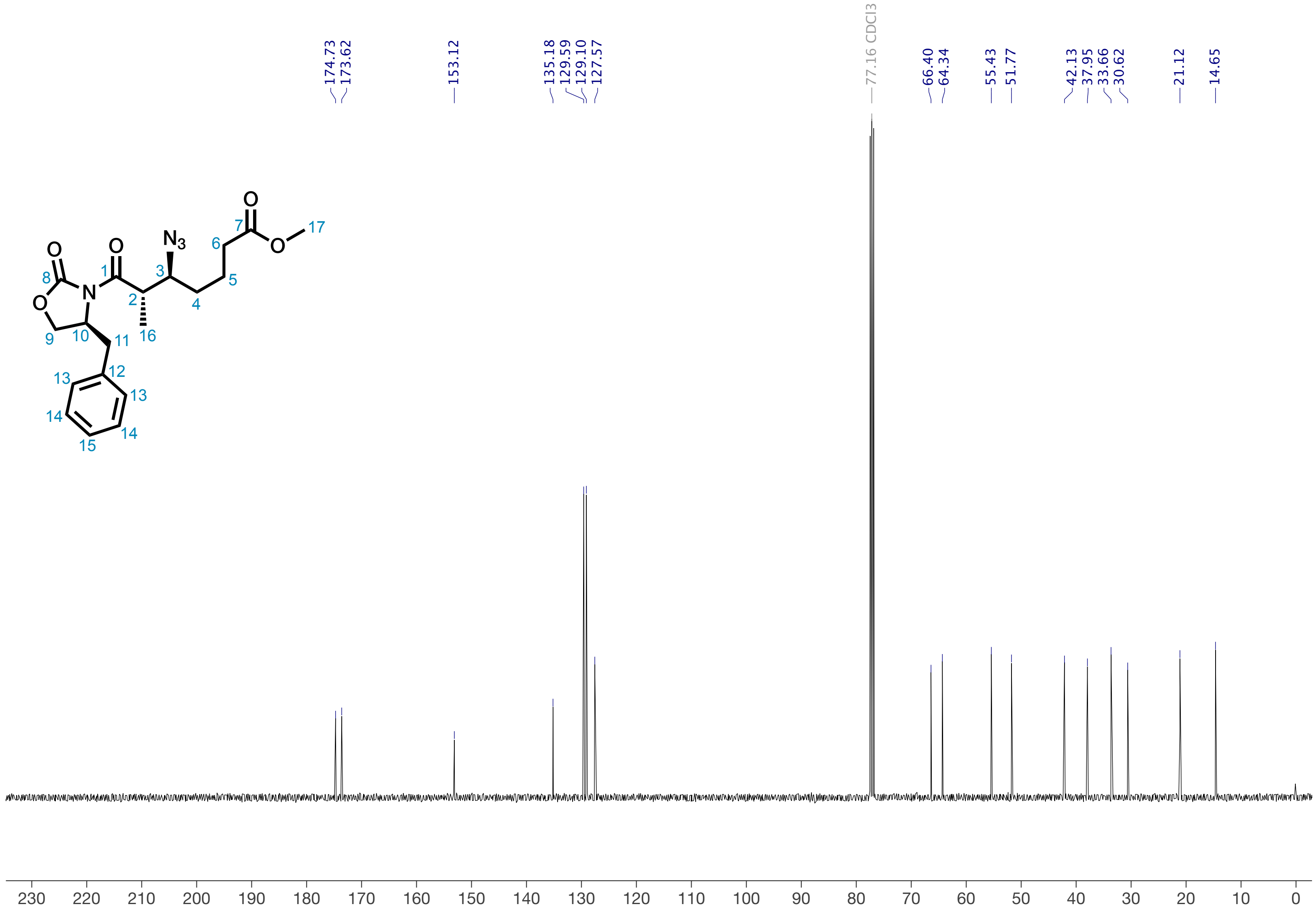

Figure S66. ^{13}C NMR (101 MHz, $CDCl_3$) of **azide SI-11**.

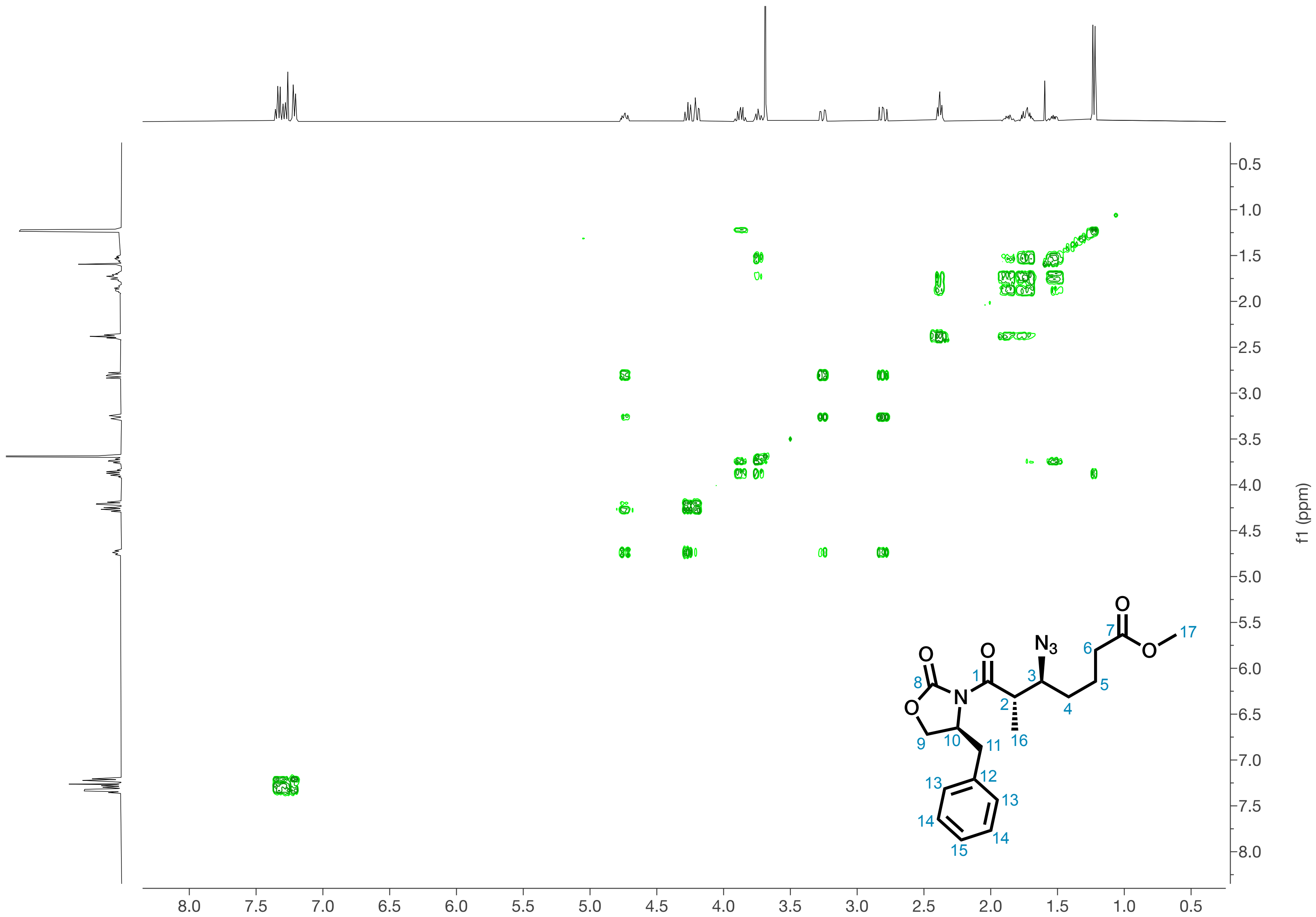

Figure S67. ^{1}H–^{1}H COSY (400 MHz, $CDCl_3$) of **azide SI-11**.

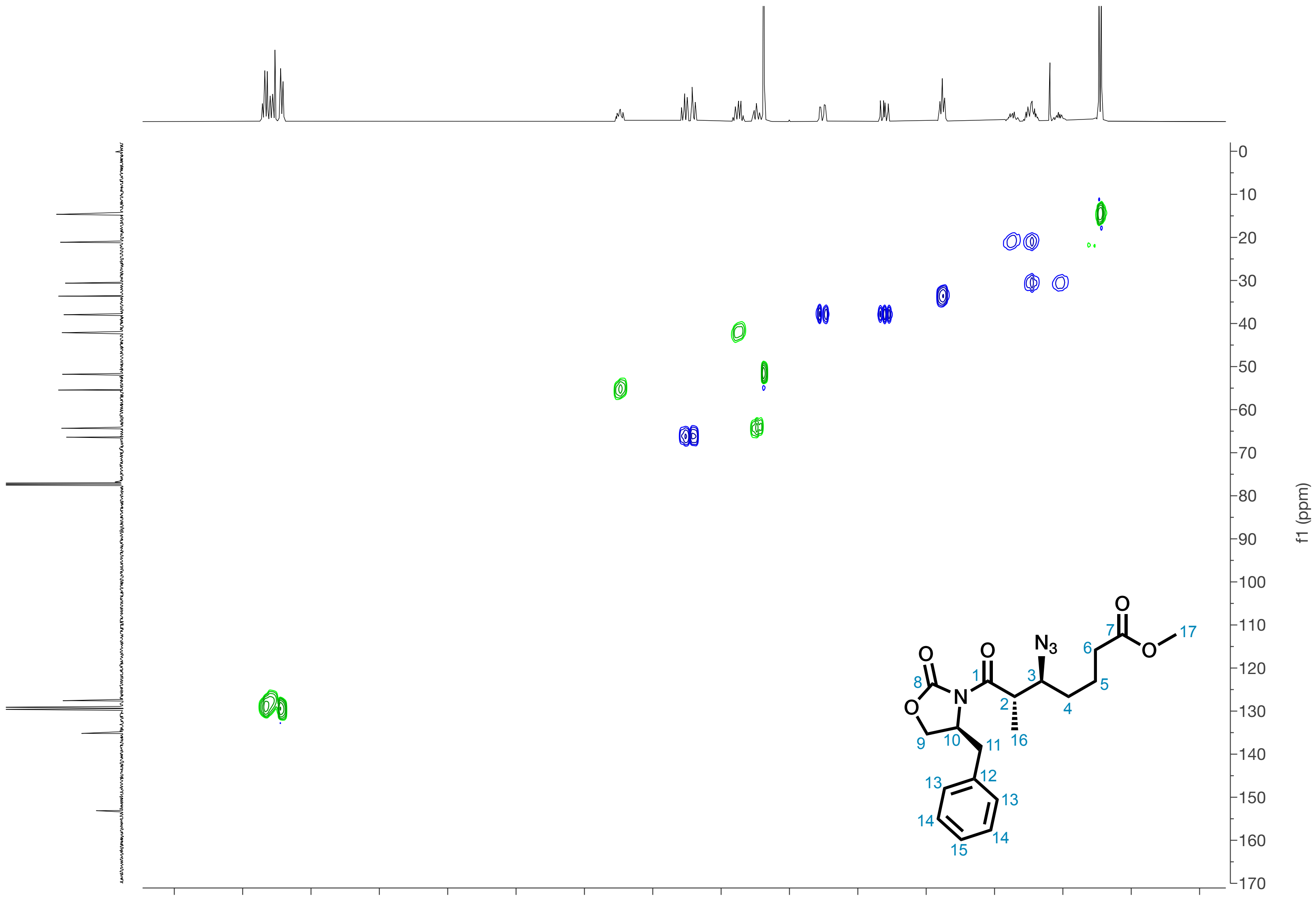

Figure S68. HSQC (400 MHz, $CDCl_3$) of **azide SI-11**.

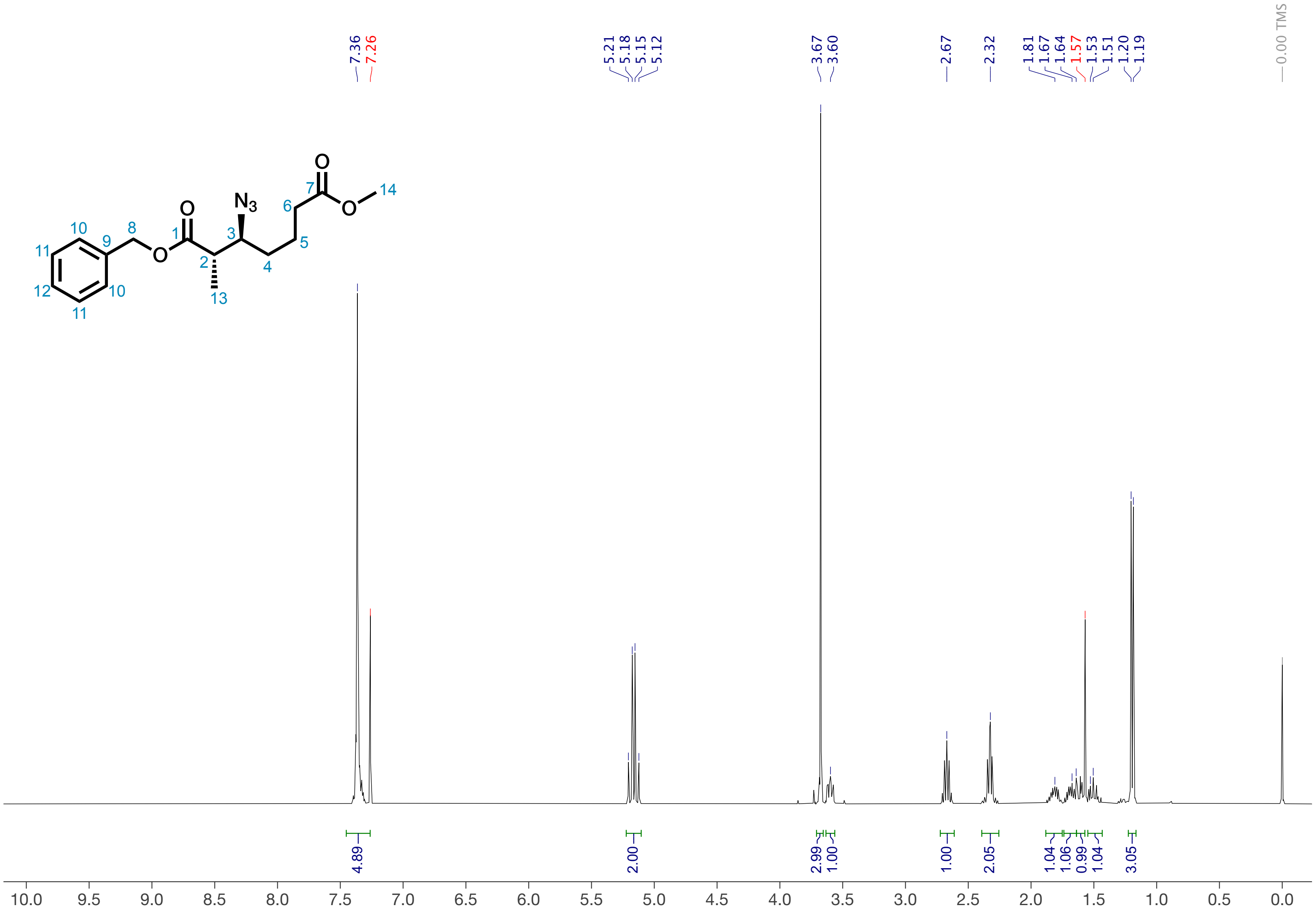

Figure S69. ^{1}H NMR (400 MHz, $CDCl_3$) of **benzyl ester 23**.

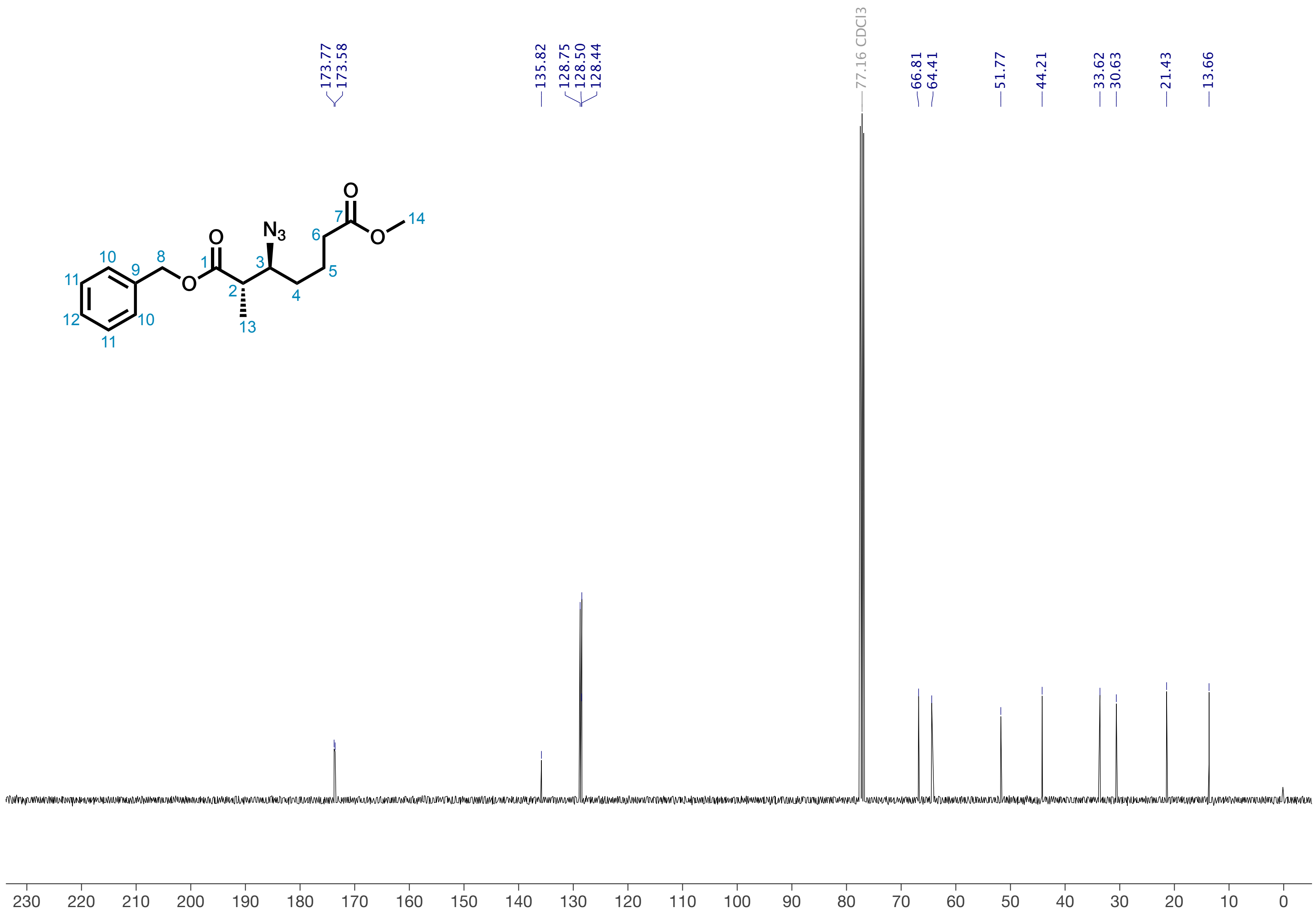

Figure S70. ^{13}C NMR (101 MHz, $CDCl_3$) of **benzyl ester 23**.

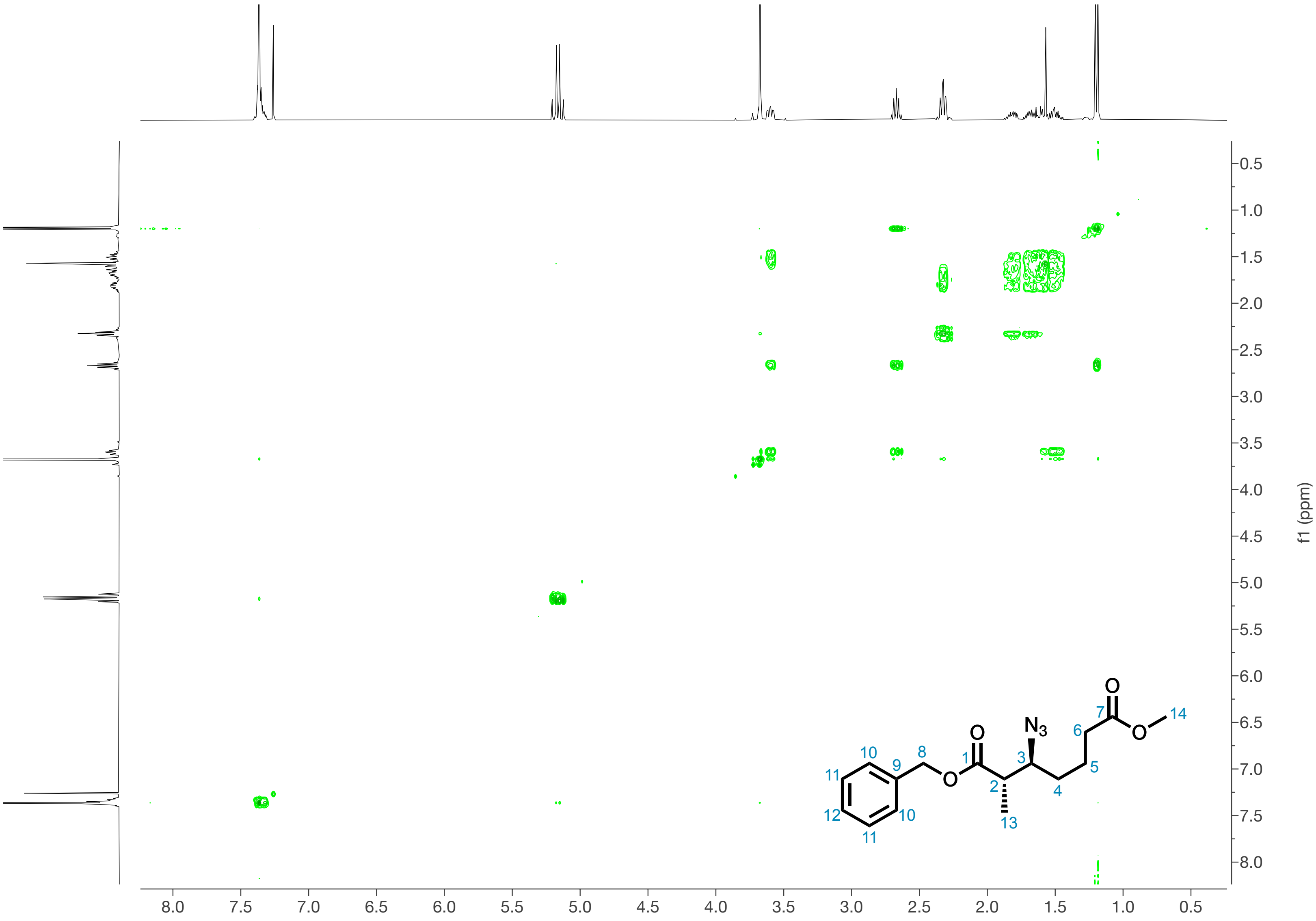

Figure S71. ¹H–¹H COSY (400 MHz, $CDCl_3$) of **benzyl ester 23**.

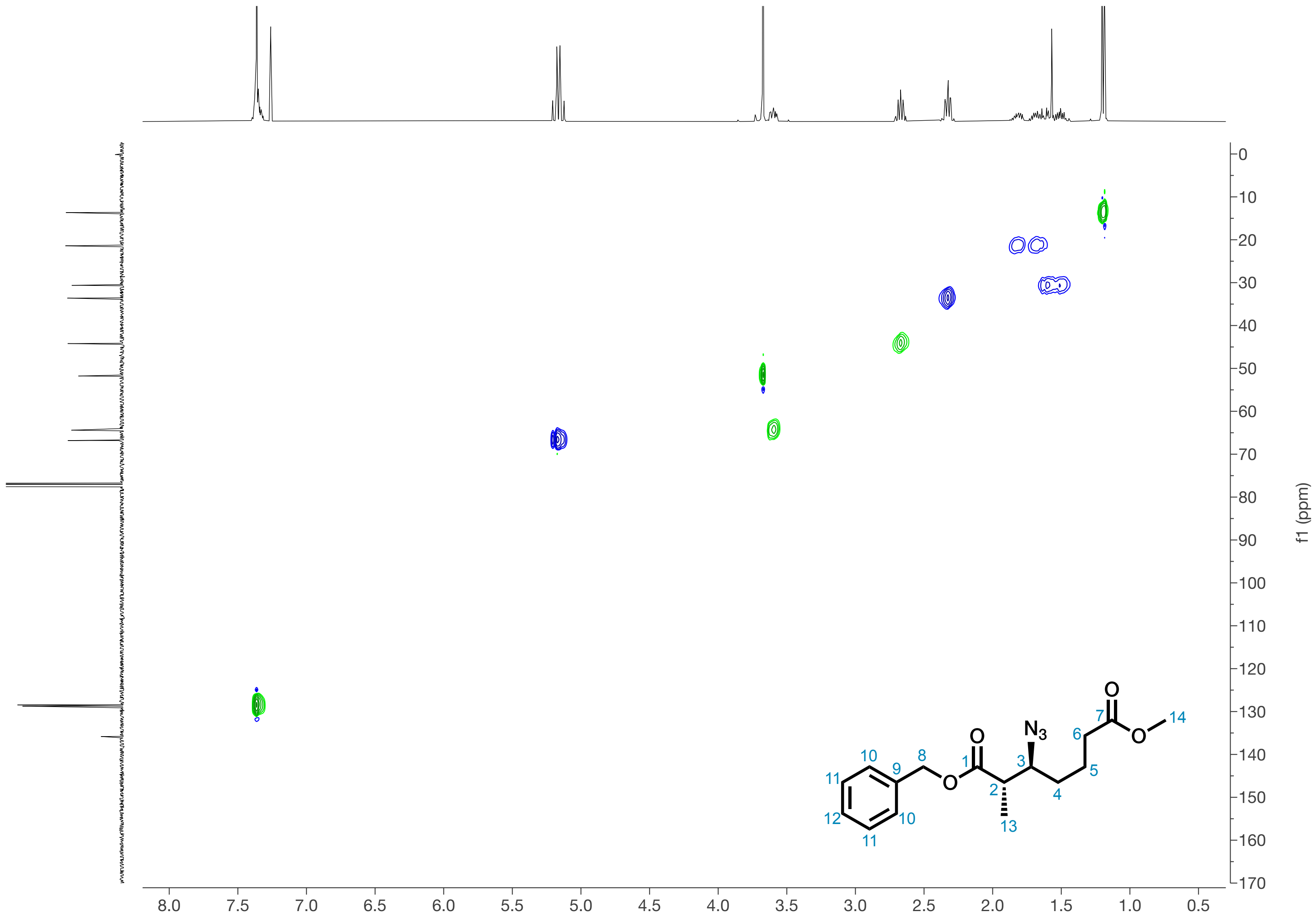

Figure S72. HSQC (400 MHz, $CDCl_3$) of **benzyl ester 23**.

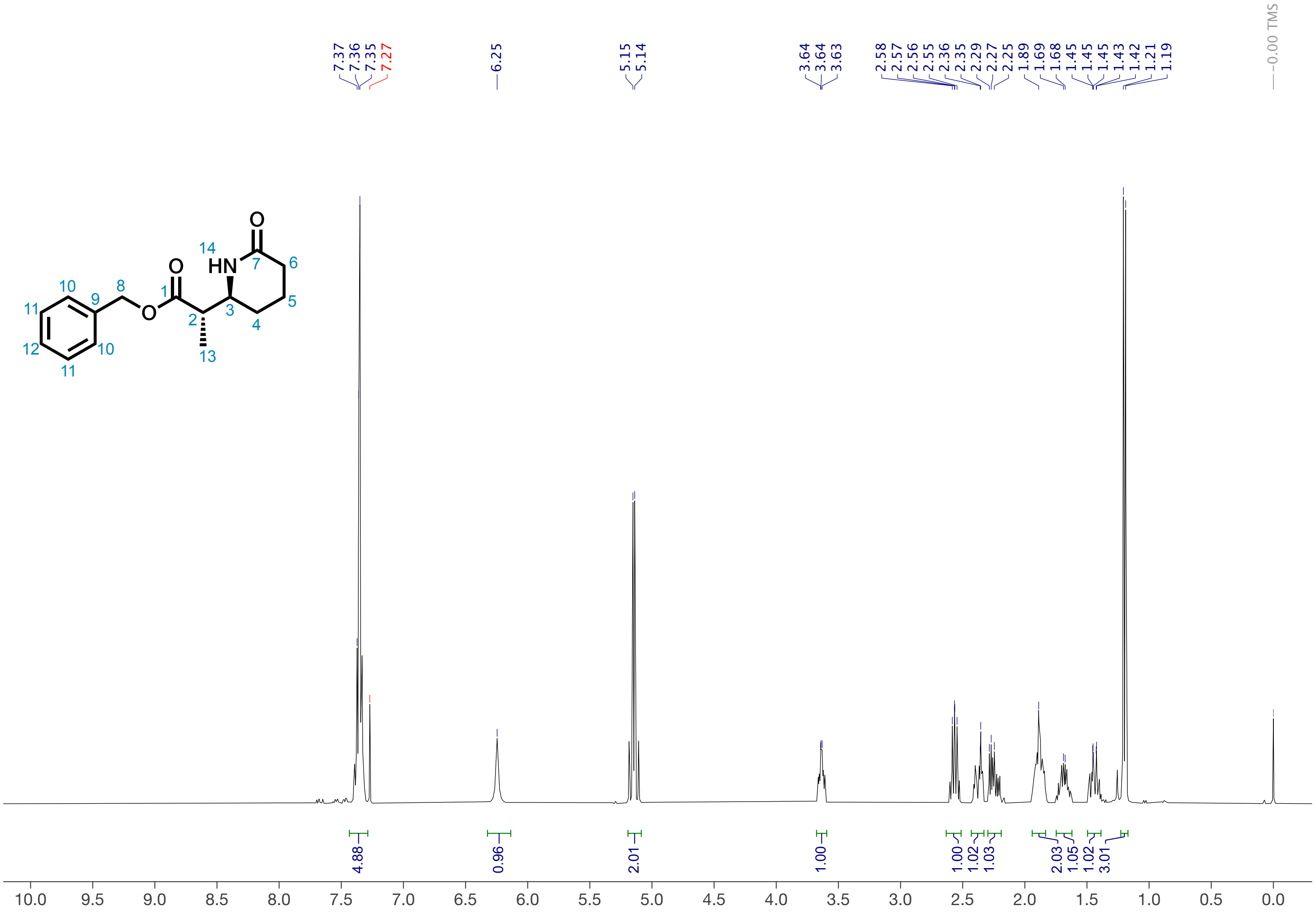

Figure S73. ^{1}H NMR (400 MHz, $CDCl_3$) of **lactam 24**.

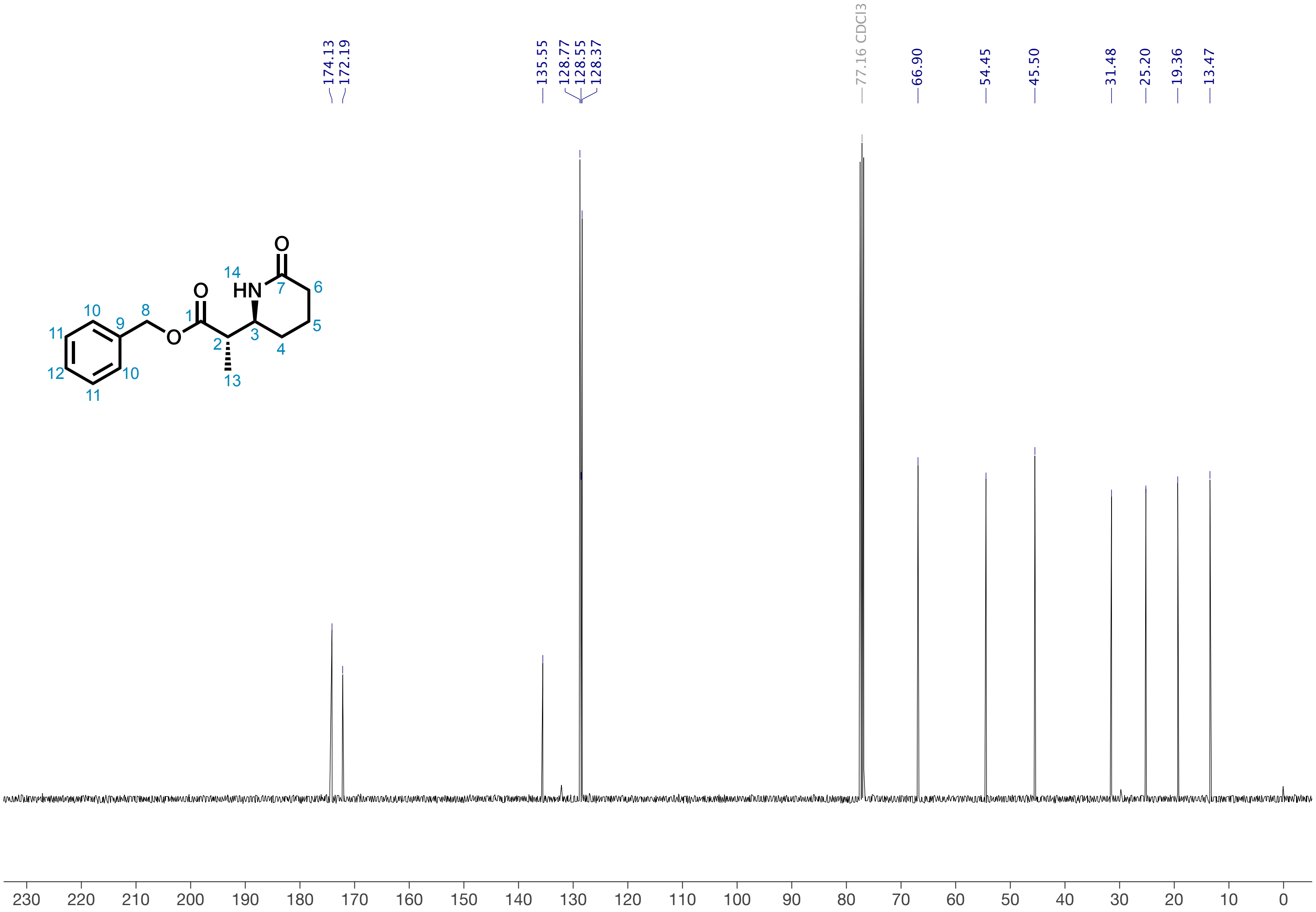

Figure S74. ^{13}C NMR (101 MHz, $CDCl_3$) of **lactam 24**.

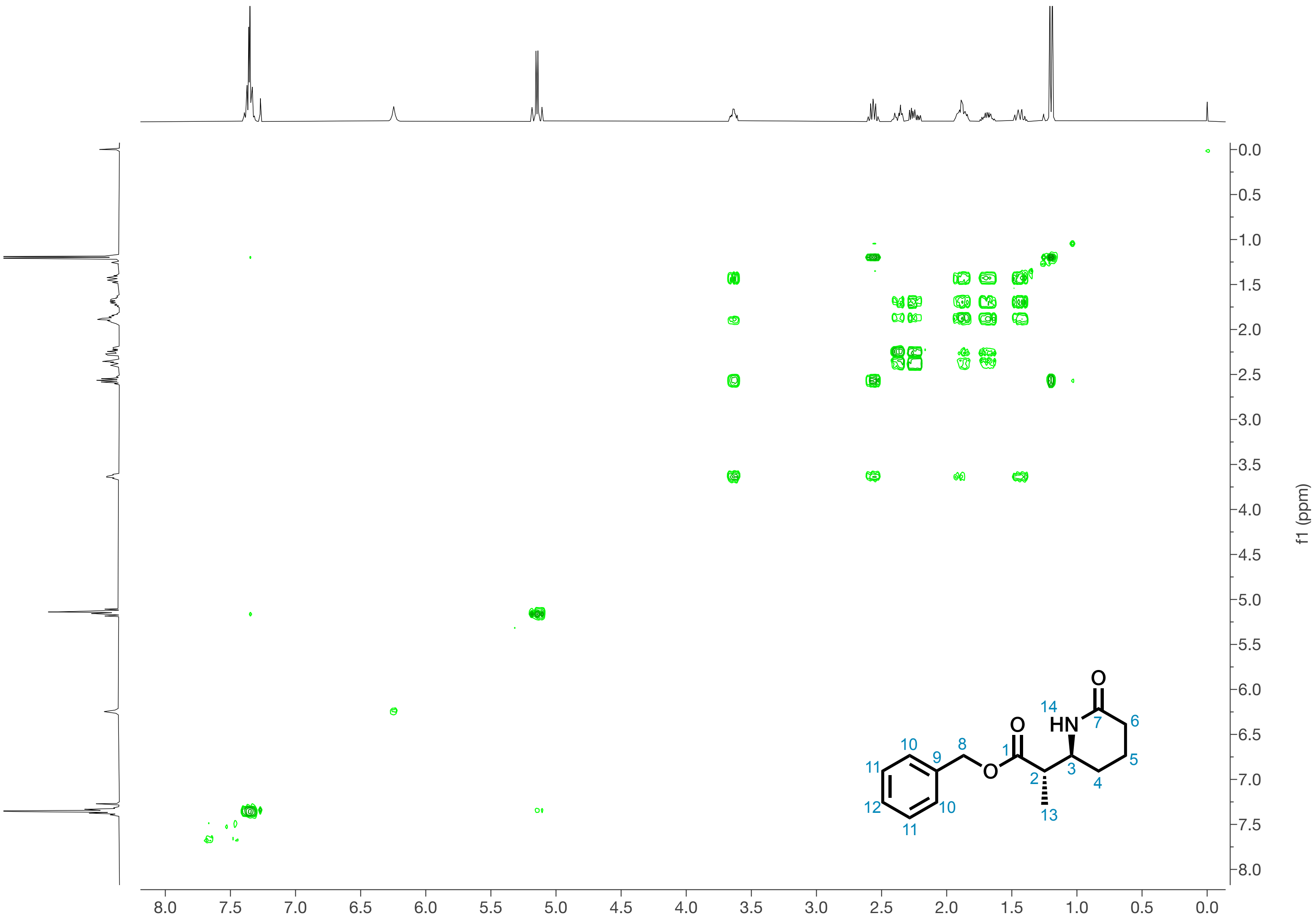

Figure S75. ^{1}H–^{1}H COSY (400 MHz, $CDCl_3$) of **lactam 24**.

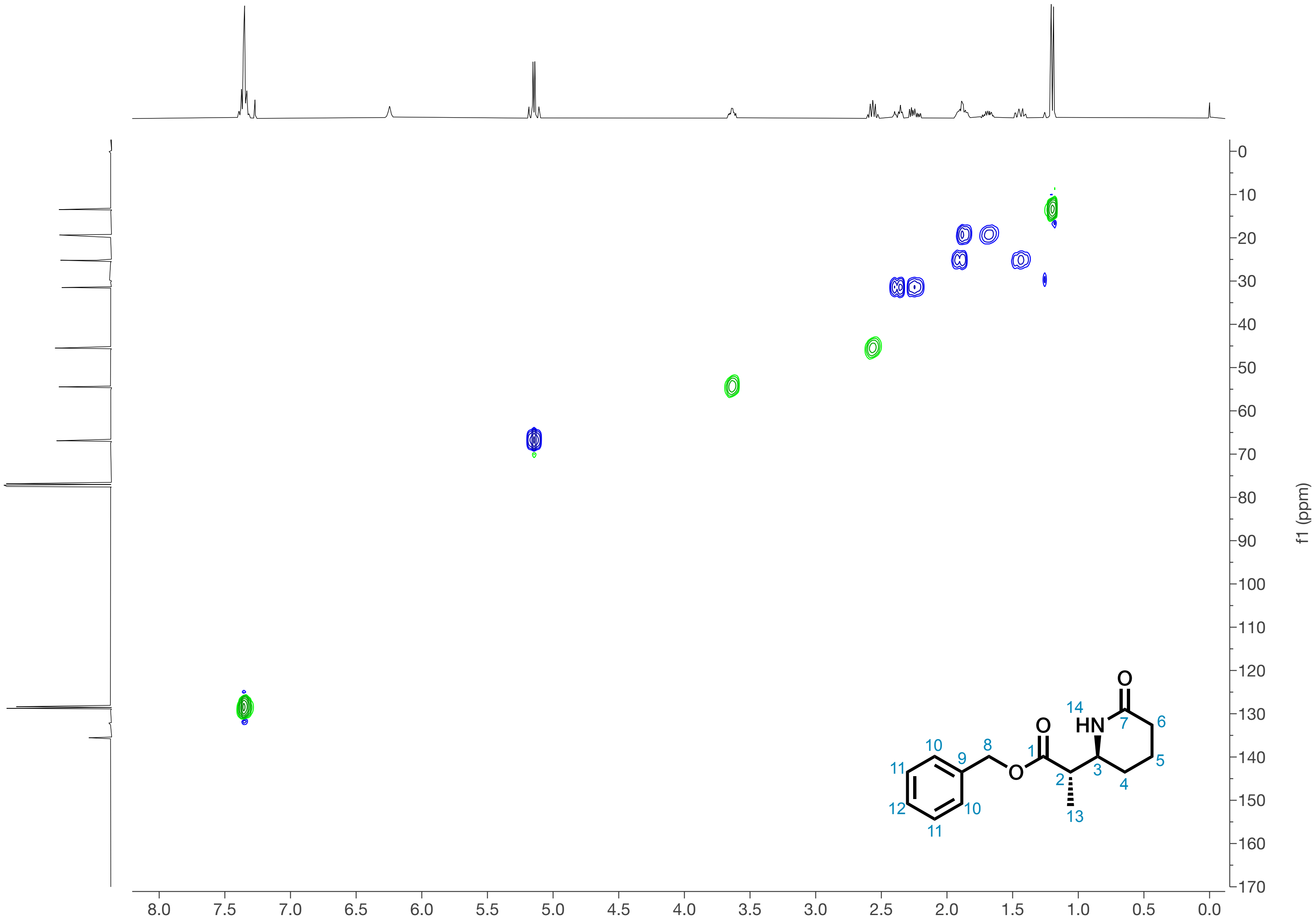

Figure S76. HSQC (400 MHz, $CDCl_3$) of **lactam 24**.

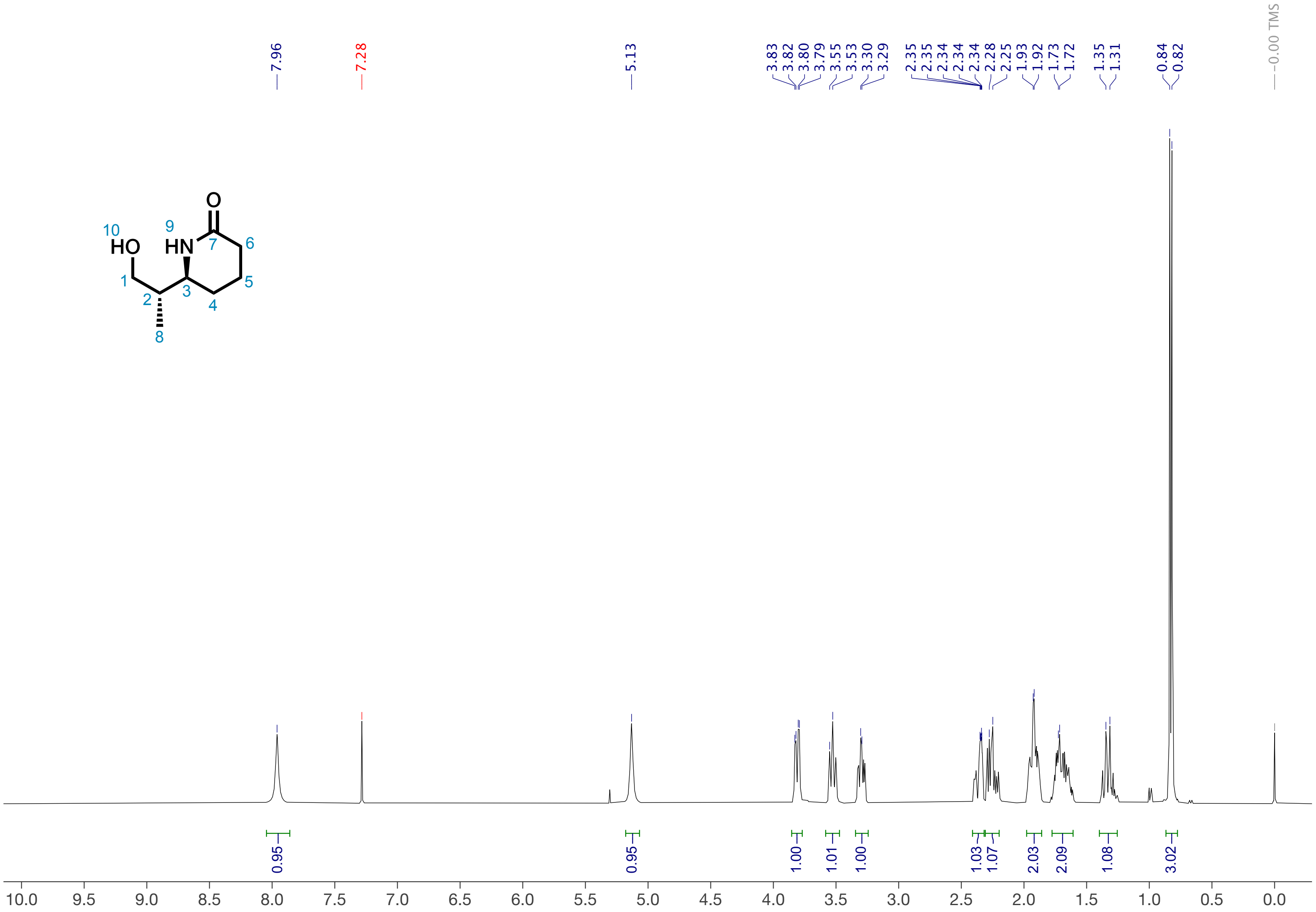

Figure S77. ^{1}H NMR (400 MHz, $CDCl_3$) of **alcohol SI-12**.

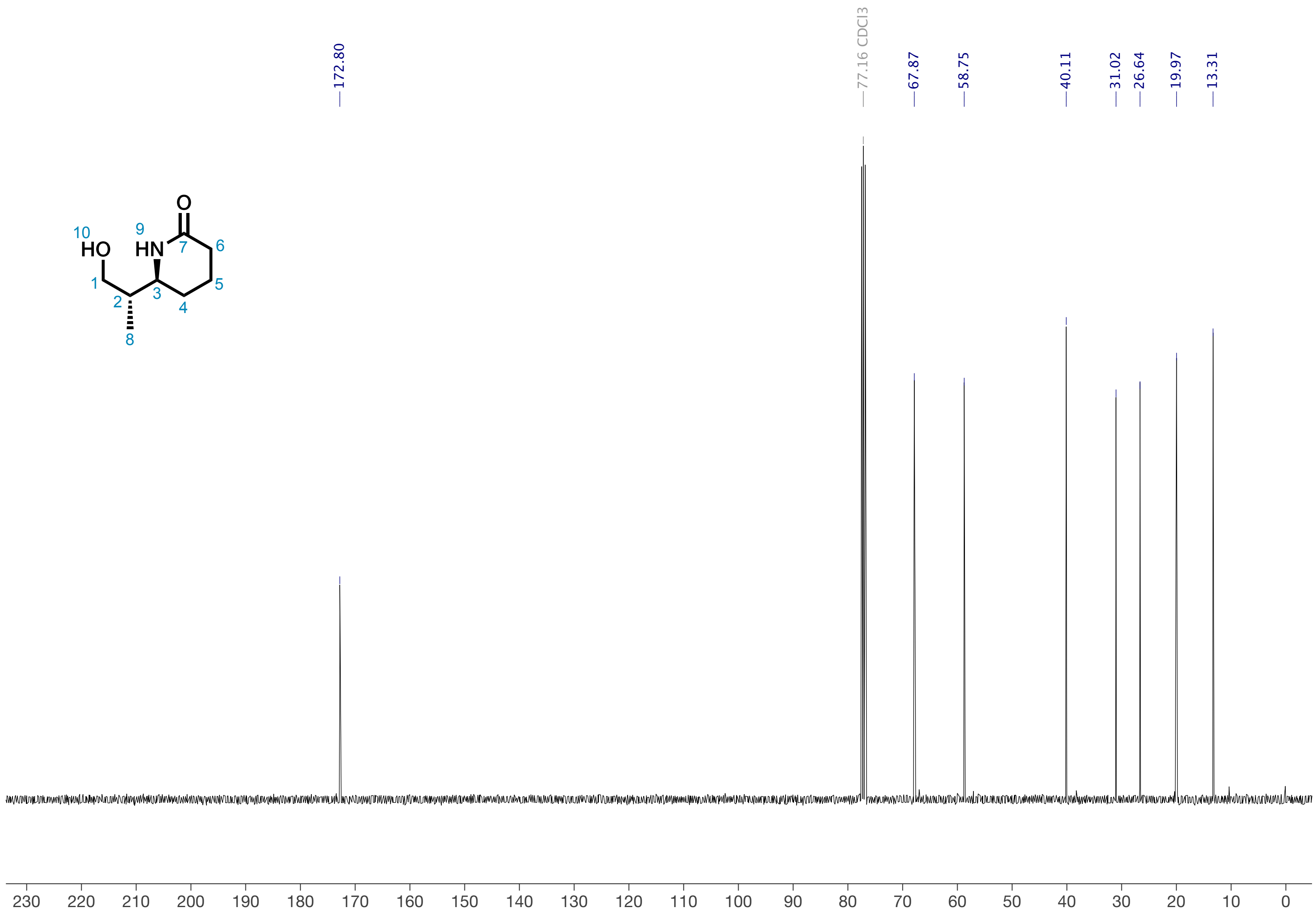

Figure S78. ^{13}C NMR (101 MHz, $CDCl_3$) of **alcohol SI-12**.

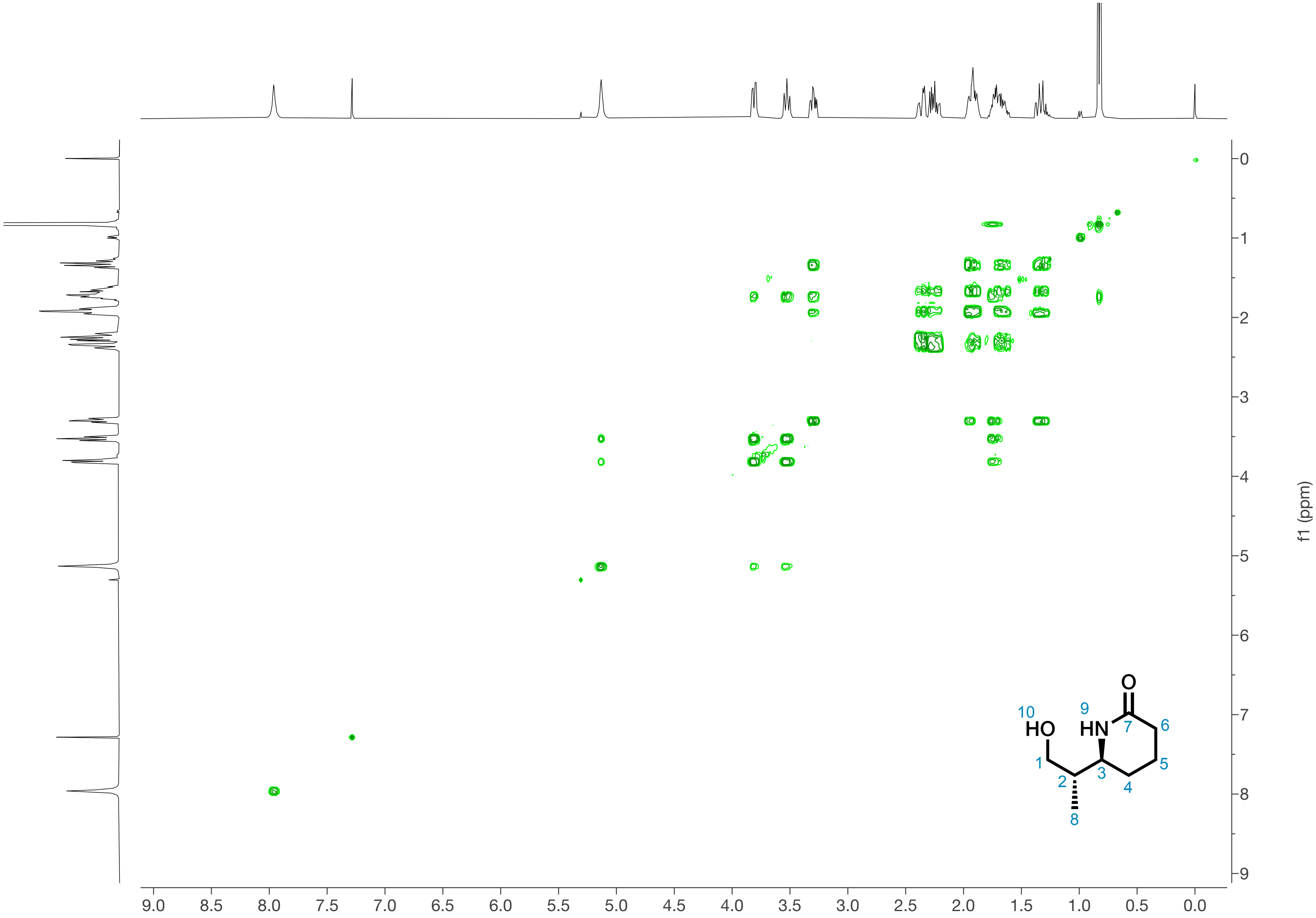

Figure S79. ^{1}H–^{1}H COSY (400 MHz, $CDCl_3$) of **alcohol SI-12**.

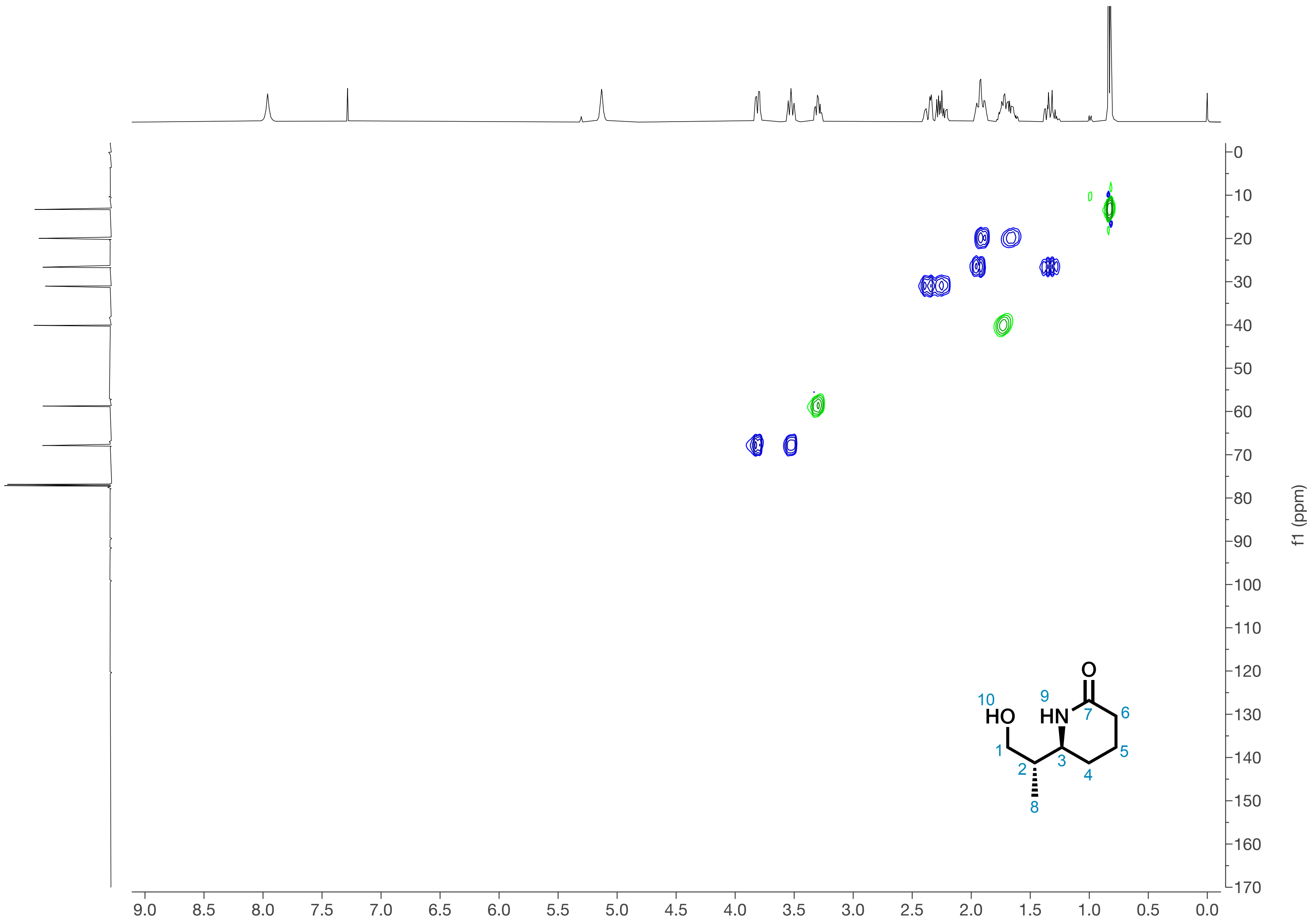

Figure S80. HSQC (400 MHz, $CDCl_3$) of **alcohol SI-12**.

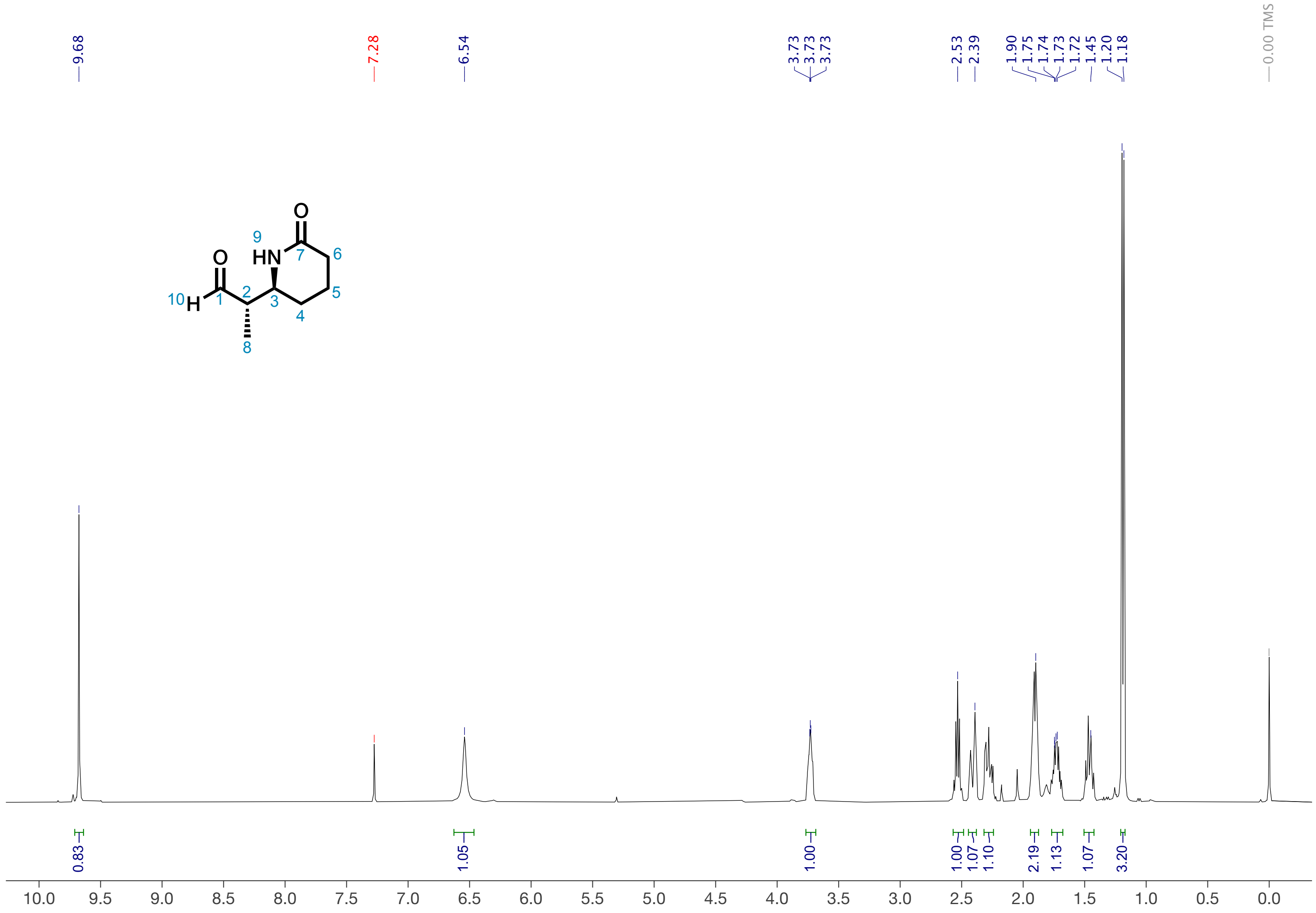

Figure S81. ^{1}H NMR (500 MHz, $CDCl_3$) of **aldehyde 25**.

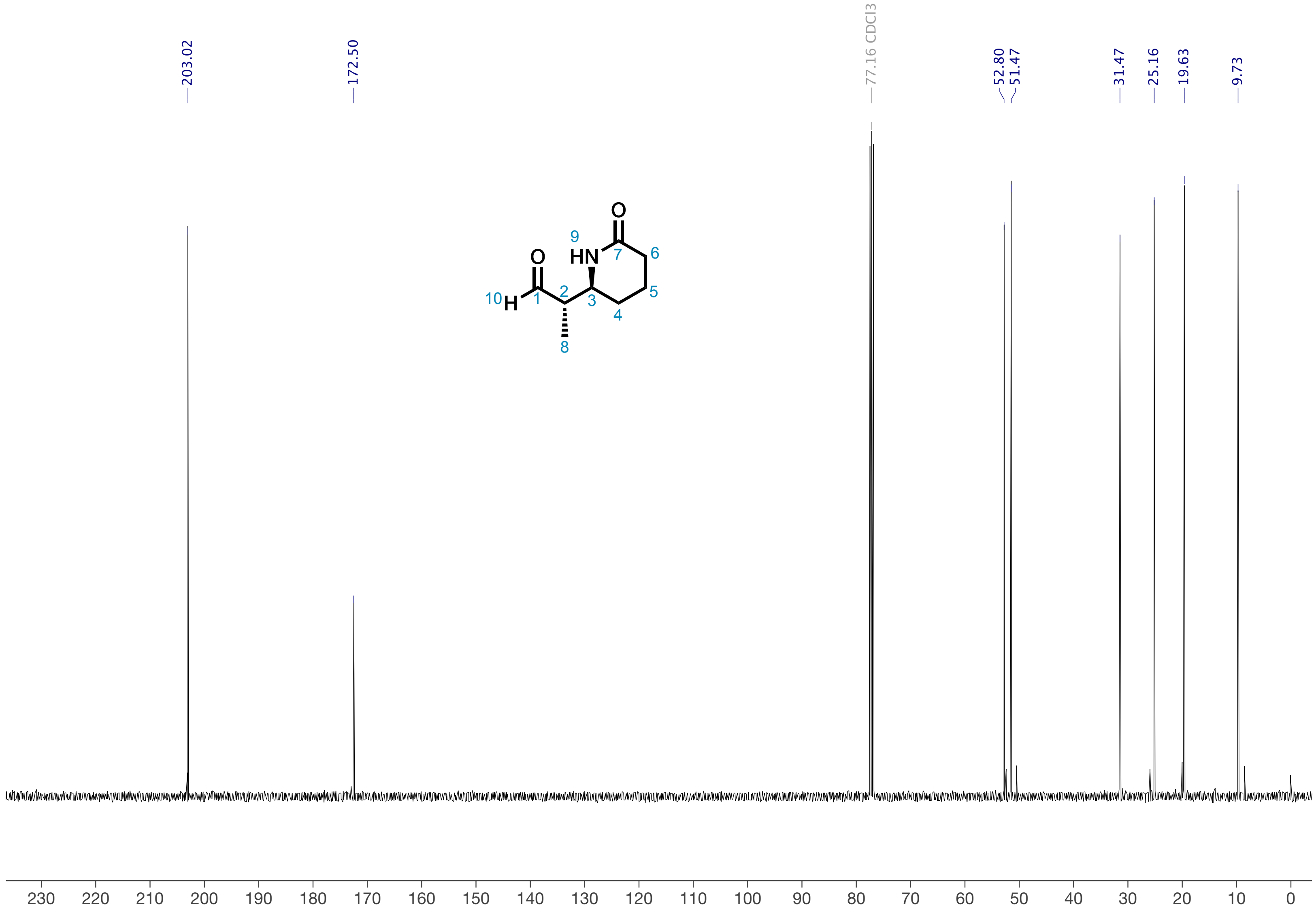

Figure S82. ^{13}C NMR (101 MHz, $CDCl_3$) of **aldehyde 25**.

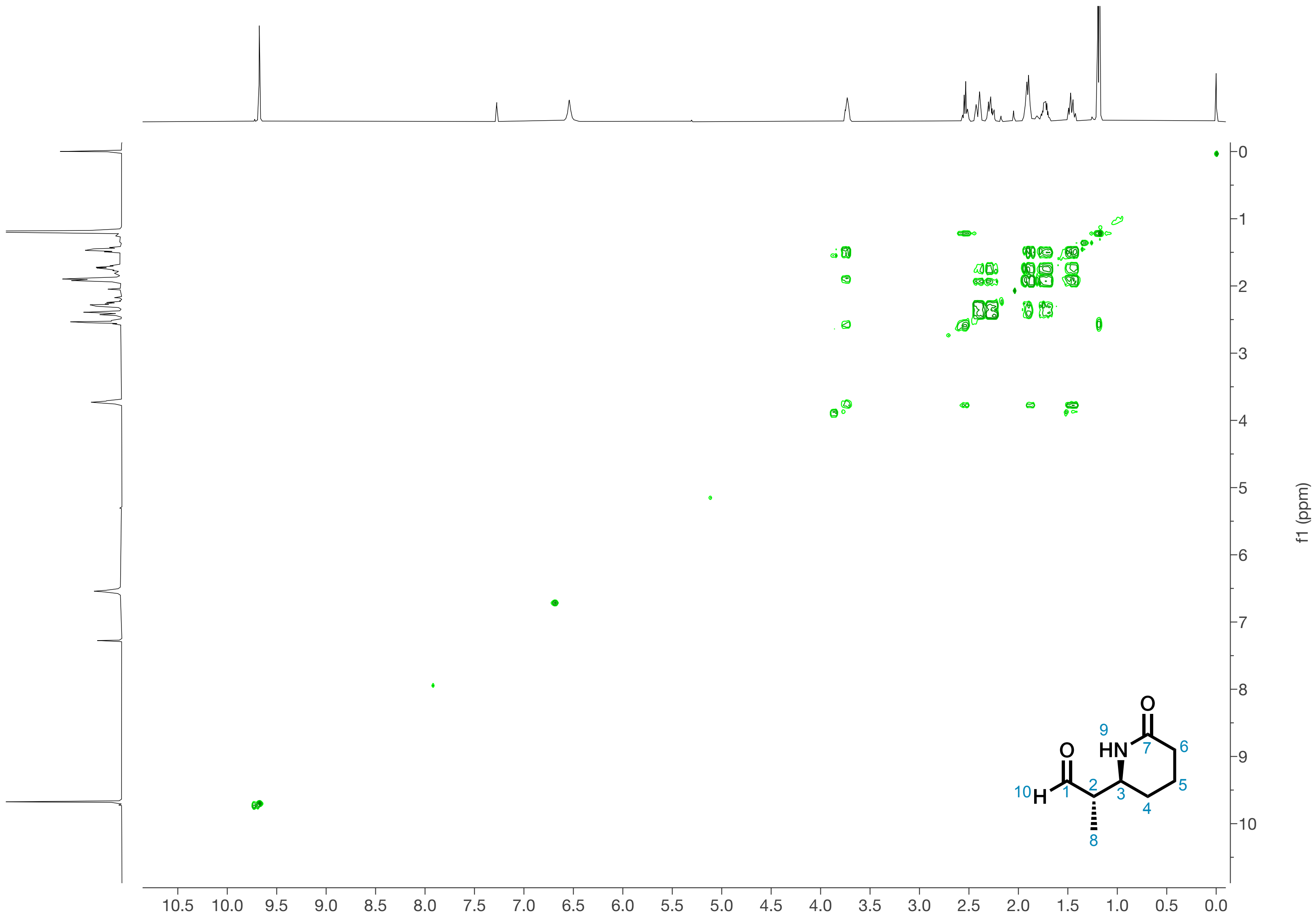

Figure S83. ^{1}H–^{1}H COSY (400 MHz, $CDCl_3$) of **aldehyde 25**.

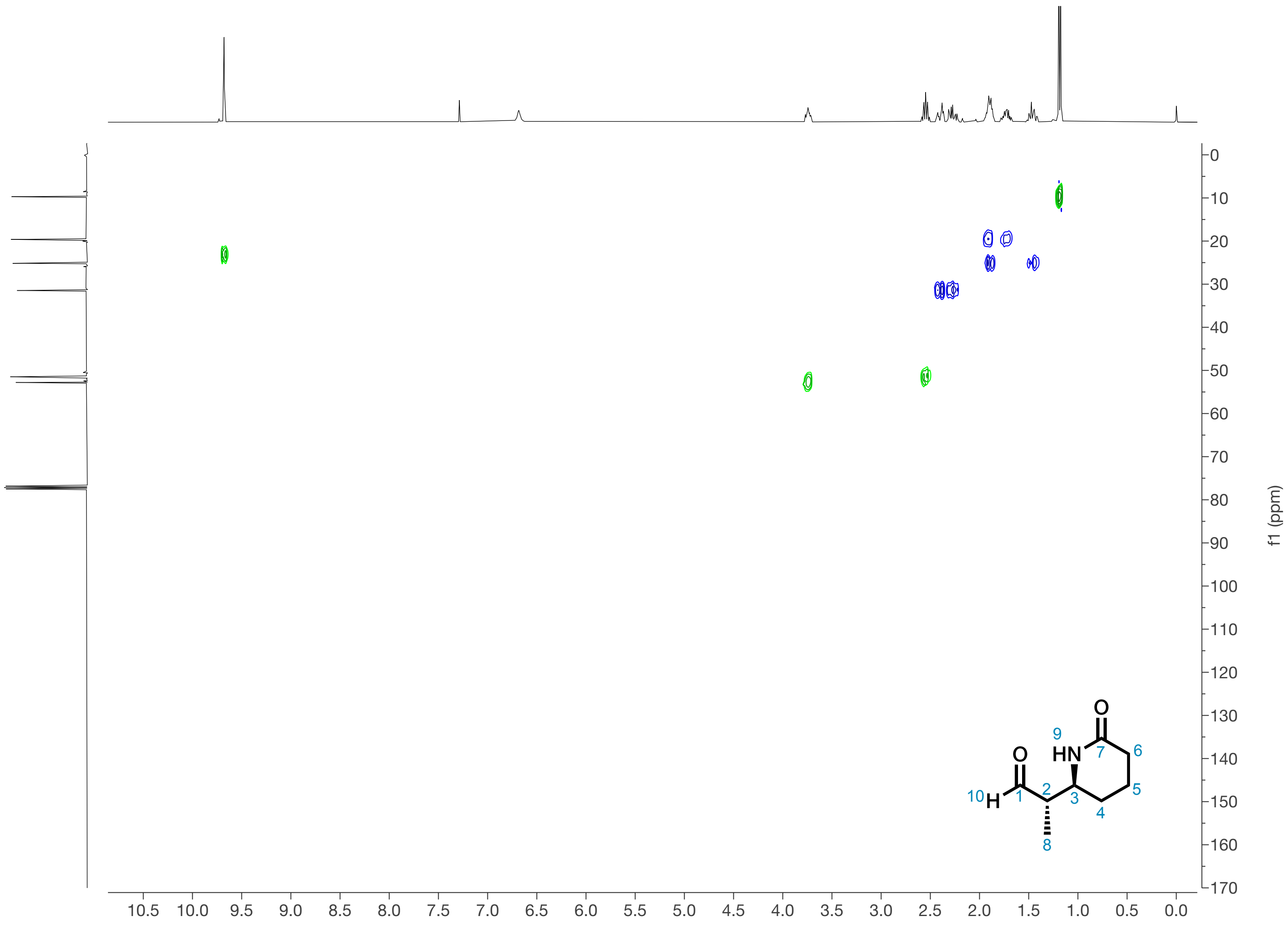

Figure S84. HSQC (400 MHz, $CDCl_3$) of **aldehyde 25**.

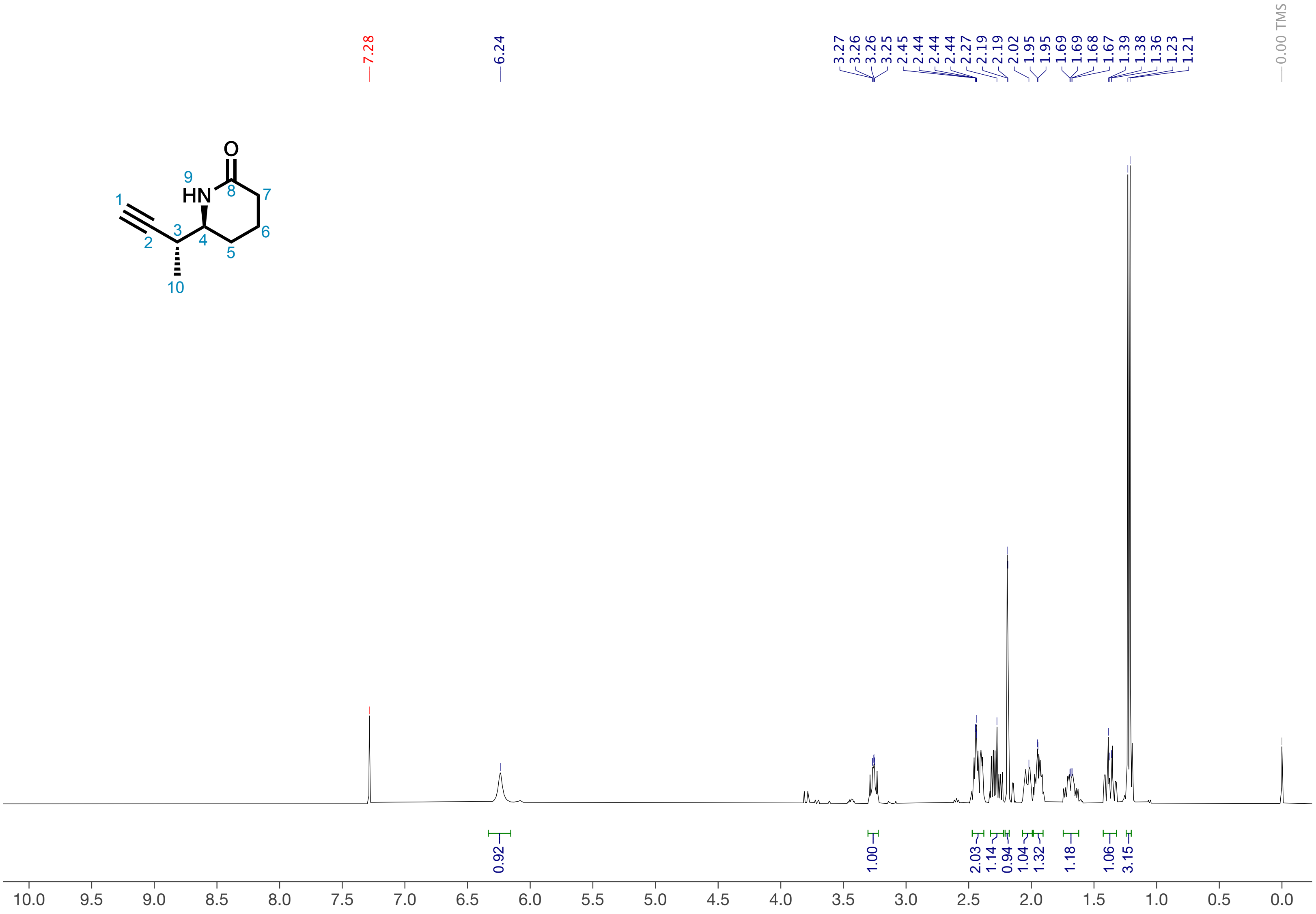

Figure S85. ^{1}H NMR (400 MHz, $CDCl_3$) of **alkyne 26**.

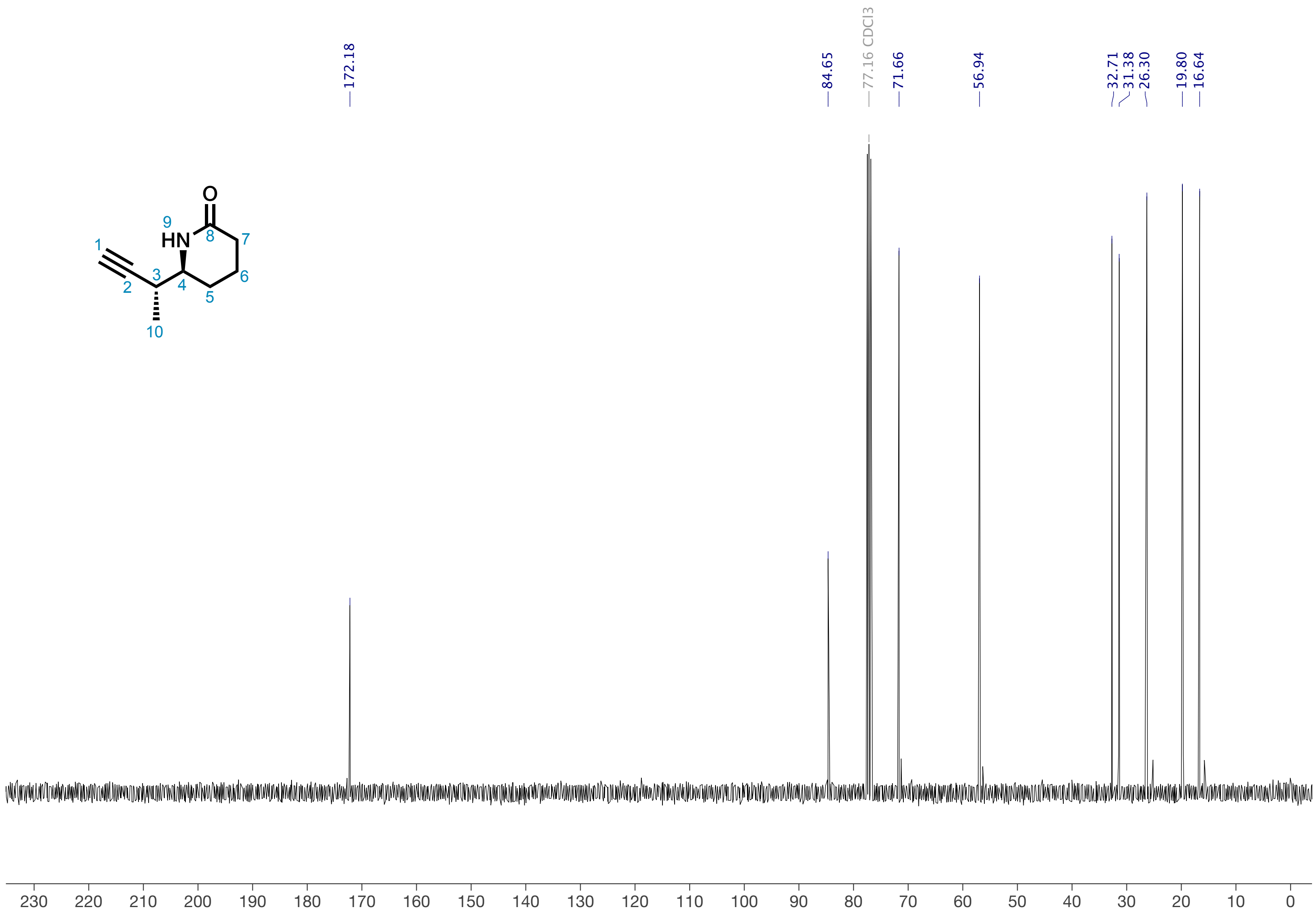

Figure S86. ^{13}C NMR (101 MHz, $CDCl_3$) of **alkyne 26**.

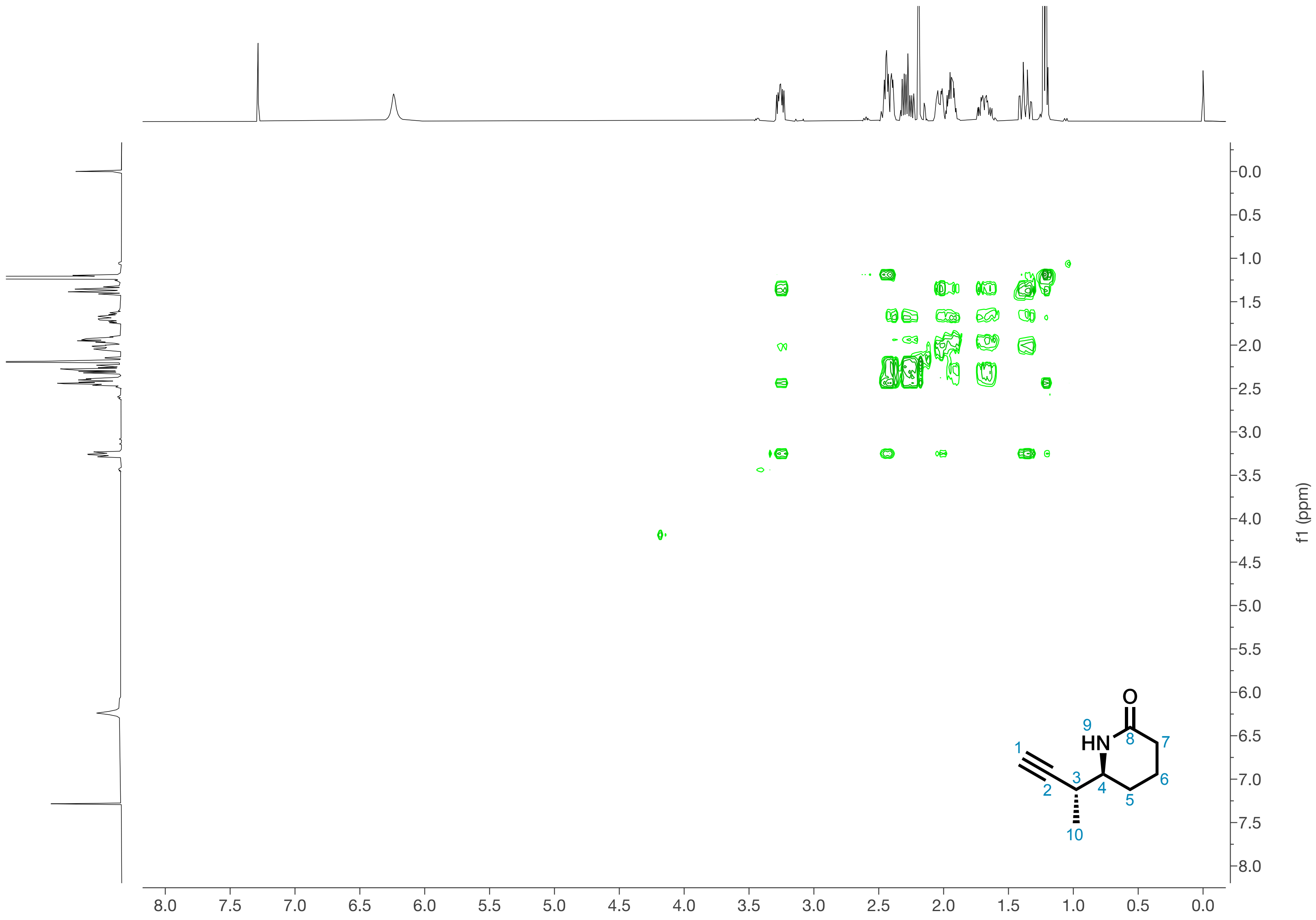

Figure S87. ^{1}H–^{1}H COSY (400 MHz, $CDCl_3$) of **alkyne 26**.

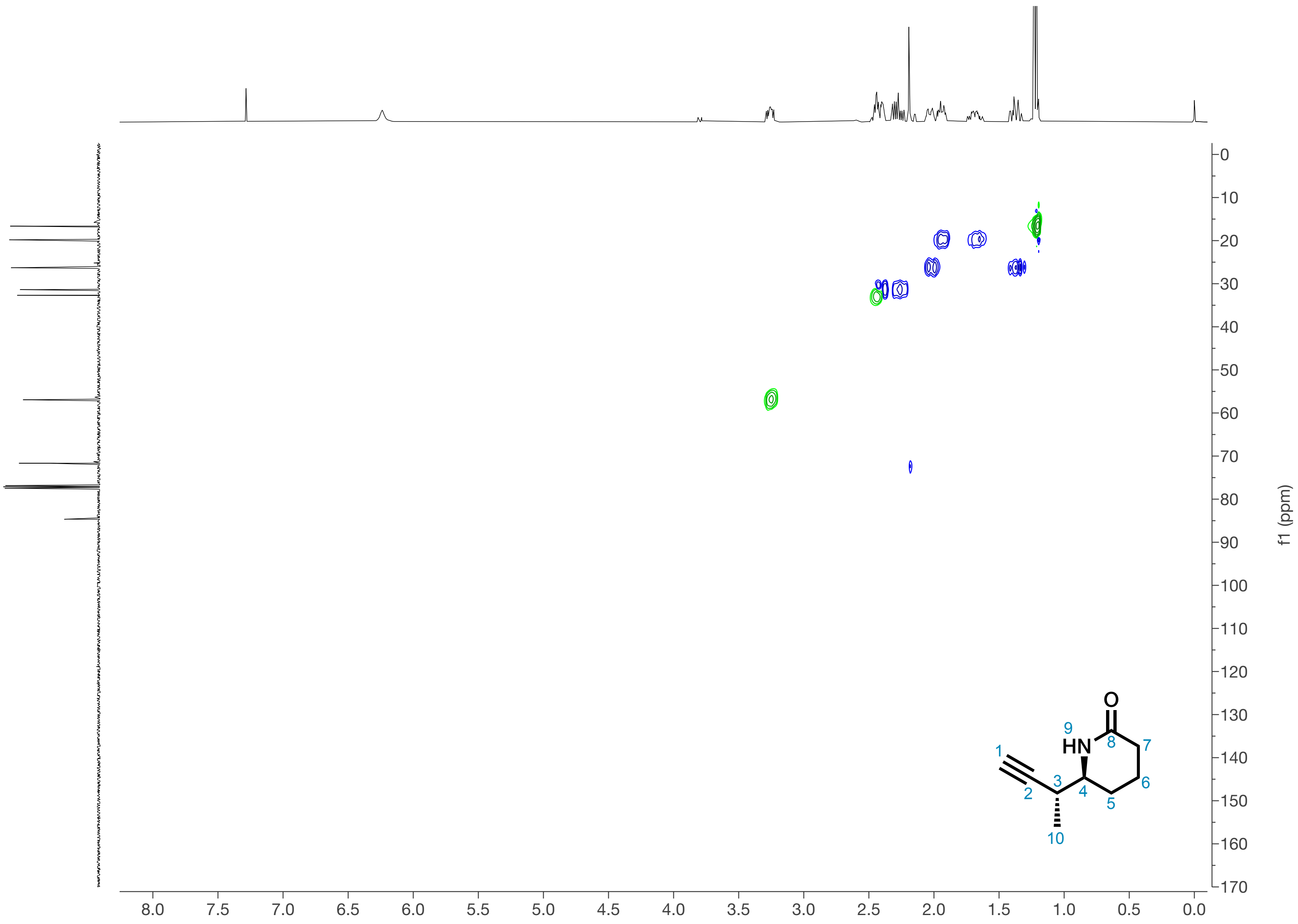

Figure S88. HSQC (400 MHz, $CDCl_3$) of **alkyne 26**.

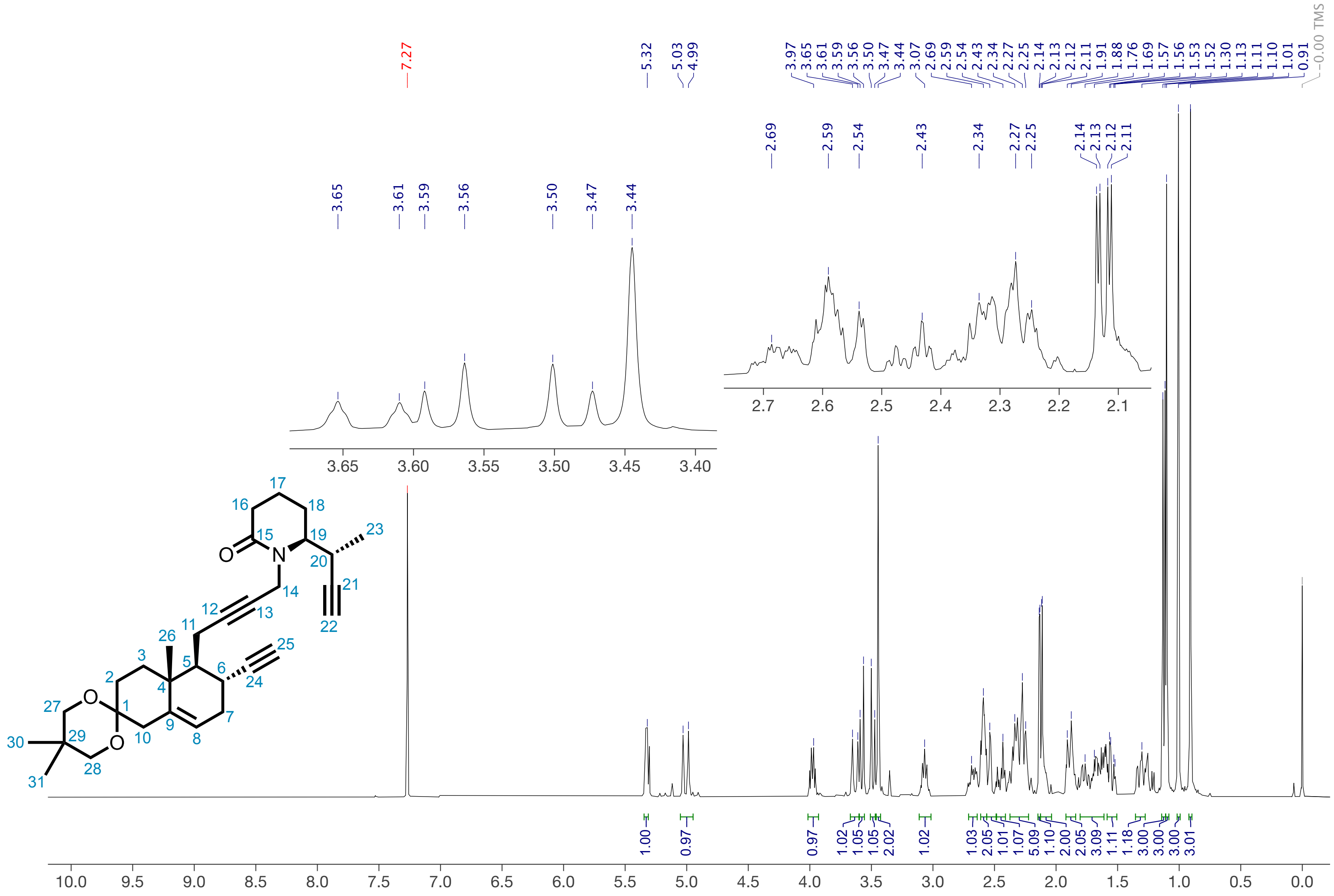

Figure S89. ^{1}H NMR (500 MHz, $CDCl_3$) of **triyne 27**.

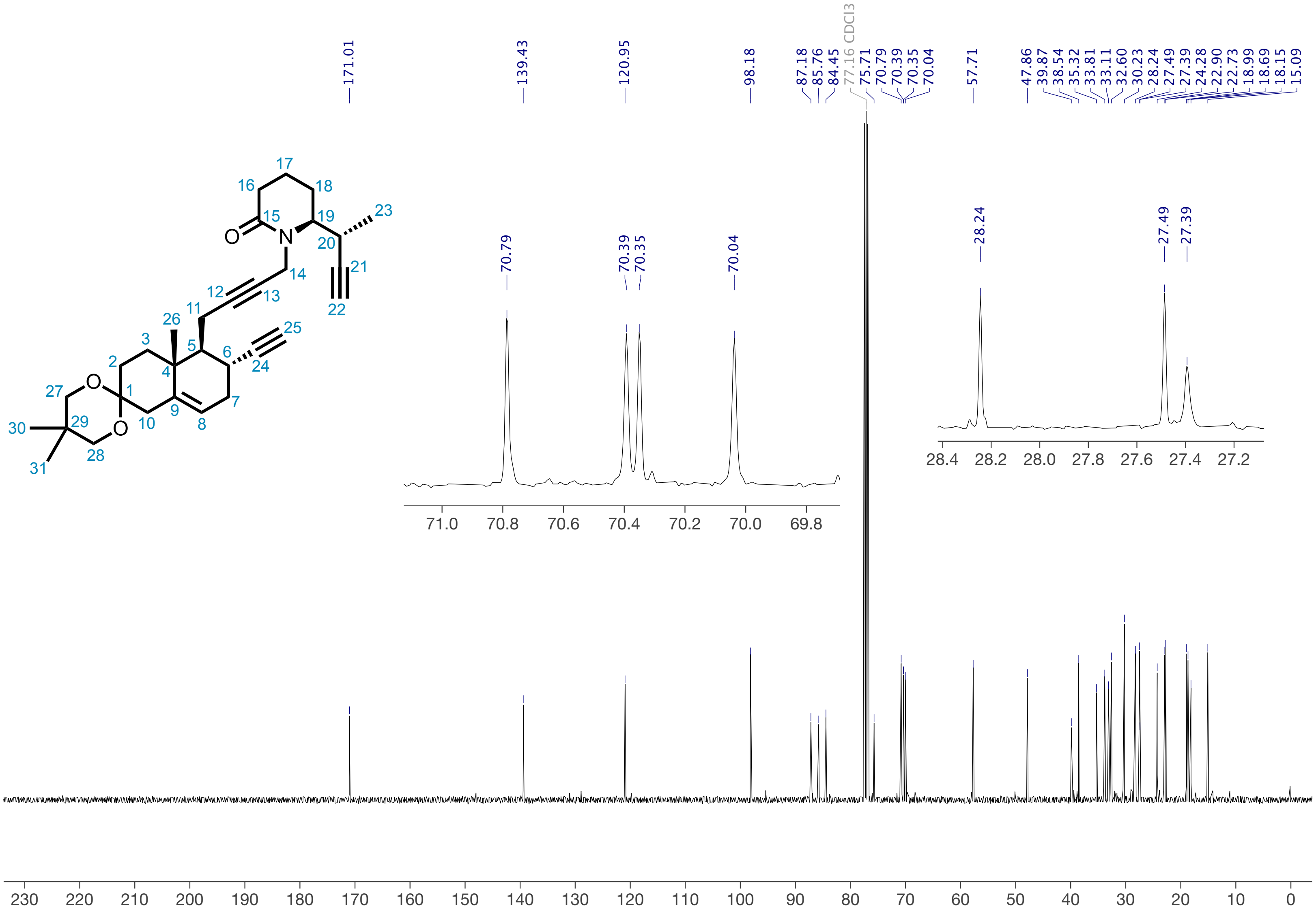

Figure S90. ^{13}C NMR (101 MHz, $CDCl_3$) of **triyne 27**.

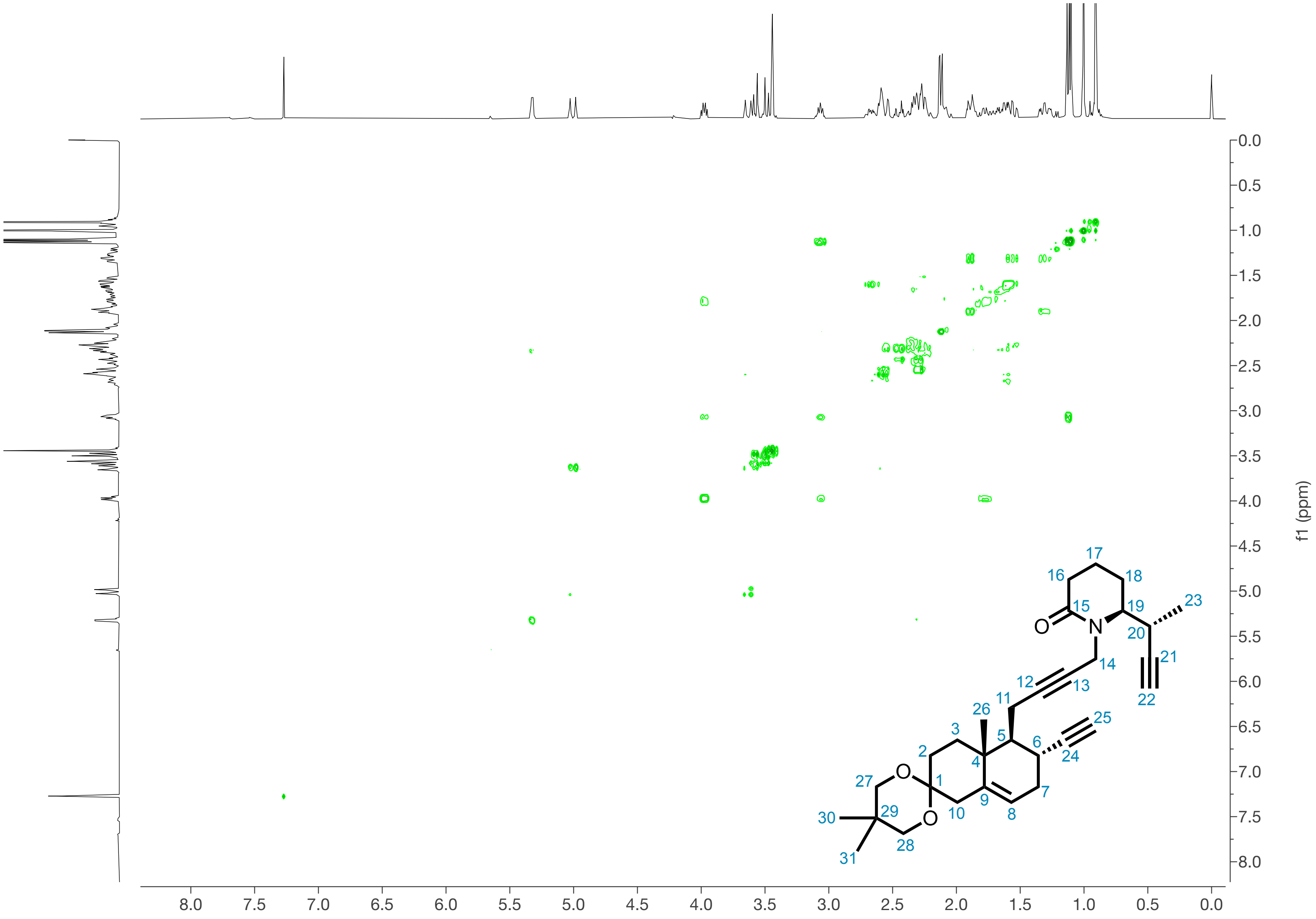

Figure S91. ^1H–^1H COSY (400 MHz, $CDCl_3$) of **triyne 27**.

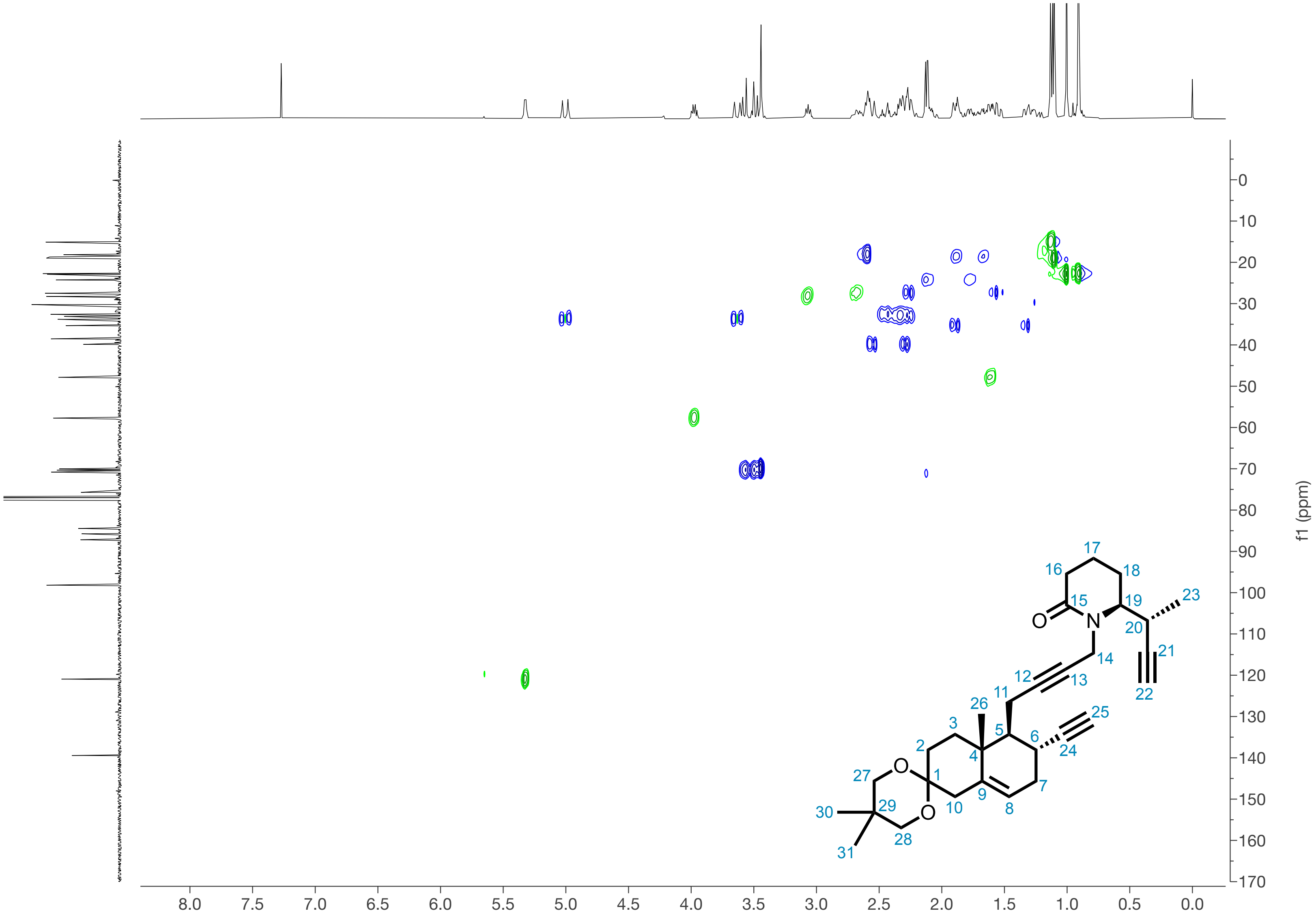

Figure S92. HSQC (400 MHz, $CDCl_3$) of **triyne 27**.

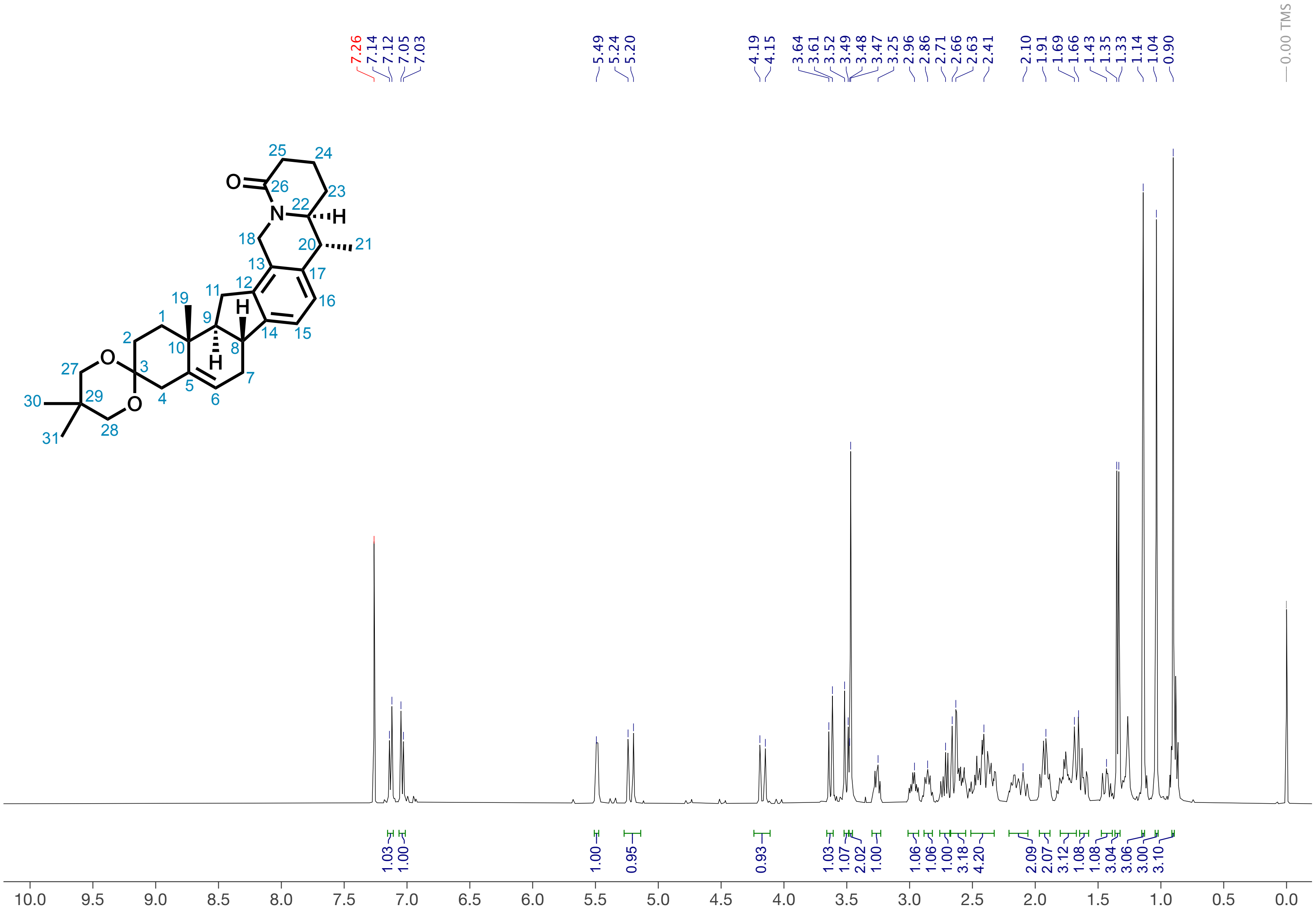

Figure S193. ^{1}H NMR (400 MHz, $CDCl_3$) of **hexacycle 28**.

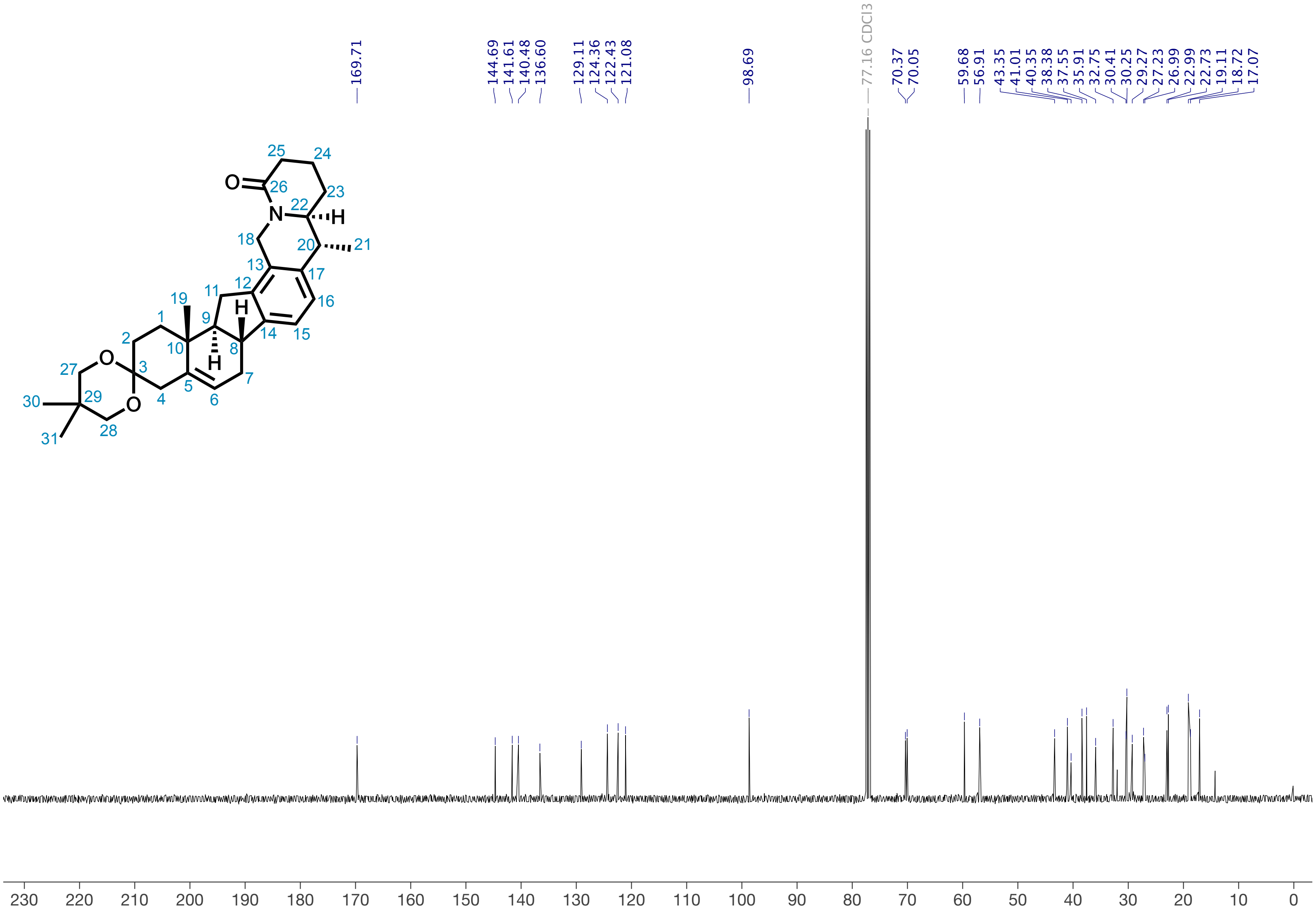

Figure S94. ^{13}C NMR (101 MHz, $CDCl_3$) of **hexacycle 28**.

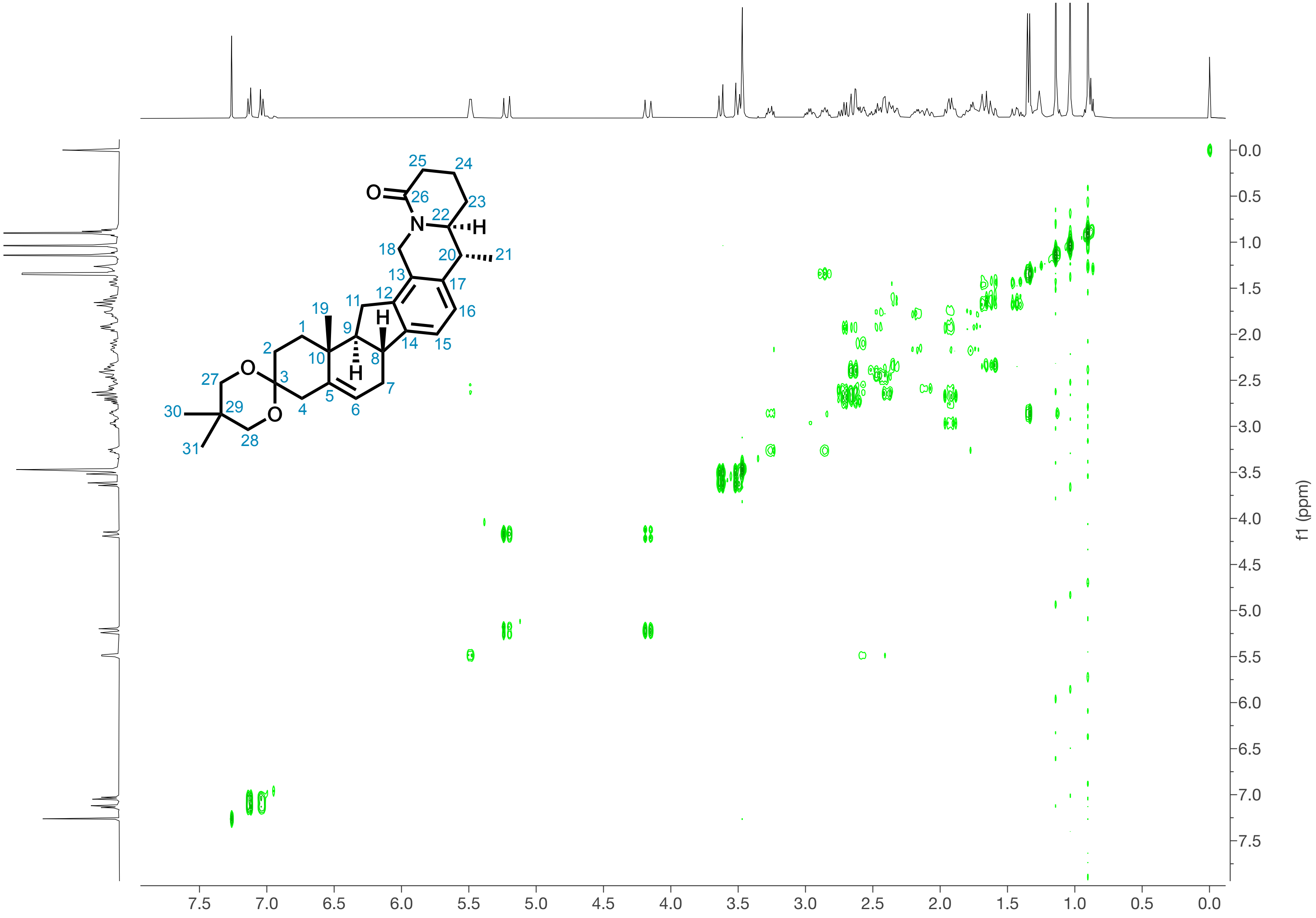

Figure S95. ^1H–^1H COSY (400 MHz, $CDCl_3$) of **hexacycle 28**.

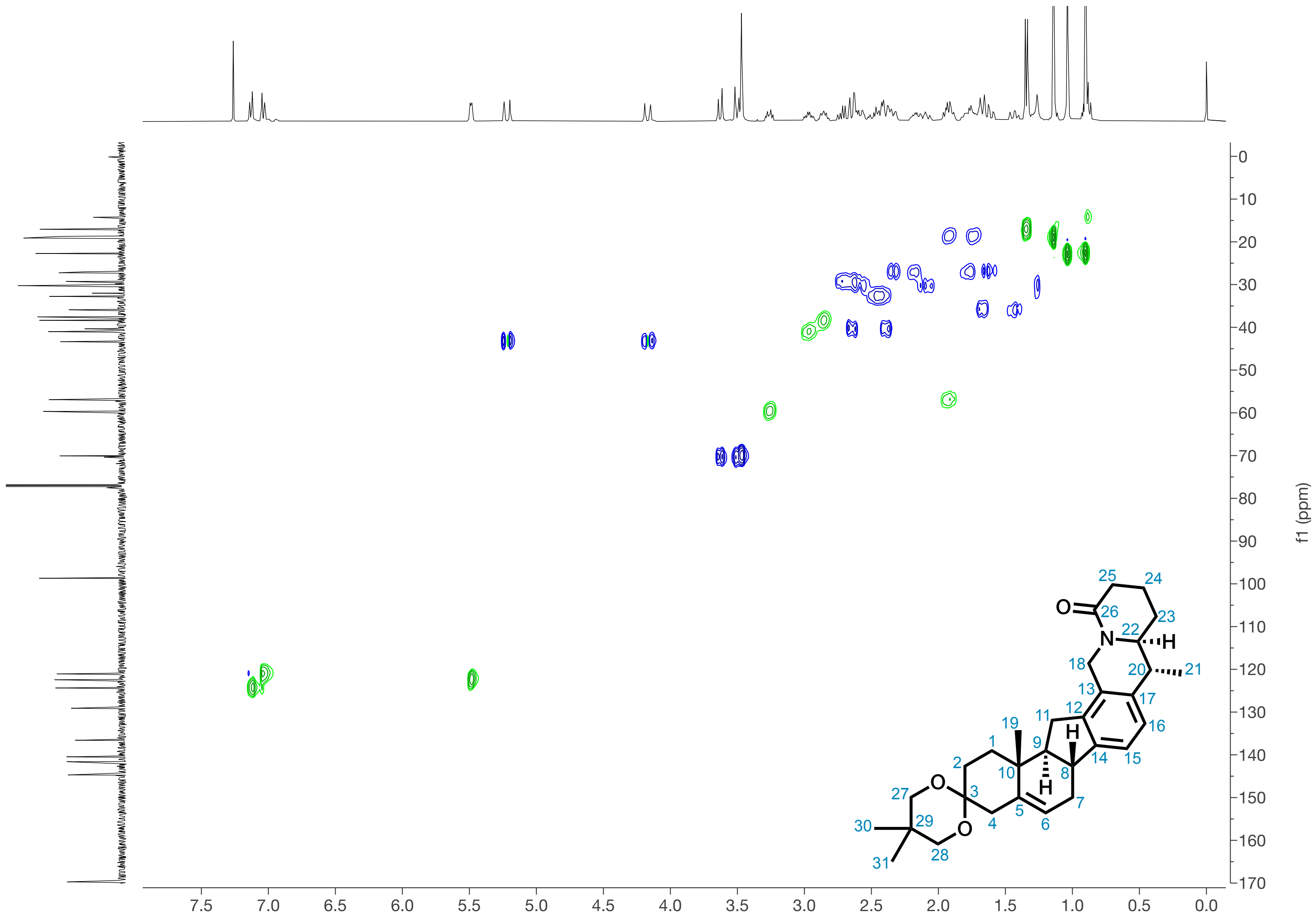

Figure S96. HSQC (400 MHz, $CDCl_3$) of **hexacycle 28**.

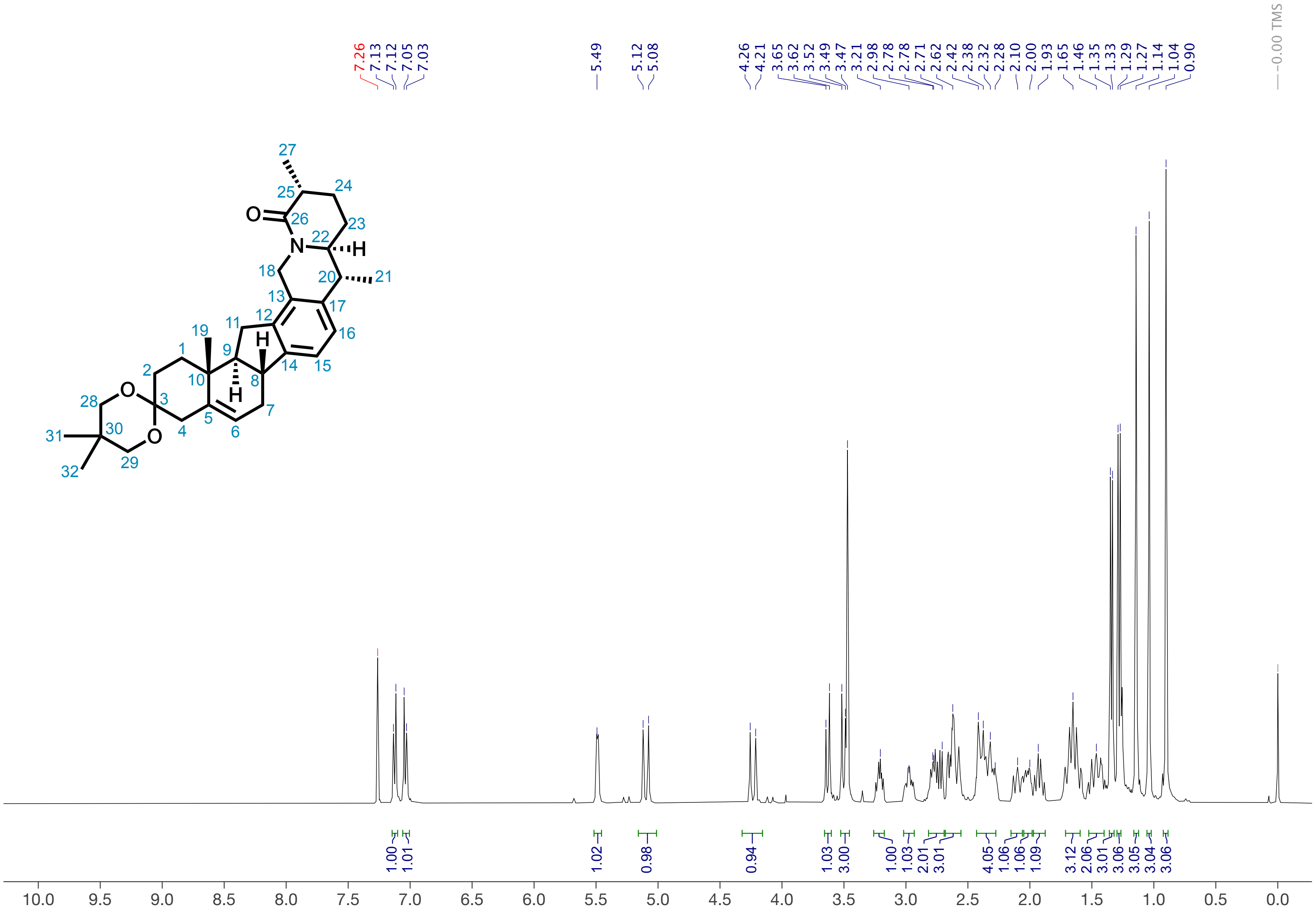

Figure S97. ^{1}H NMR (400 MHz, $CDCl_3$) of **α-methyl lactam 29**.

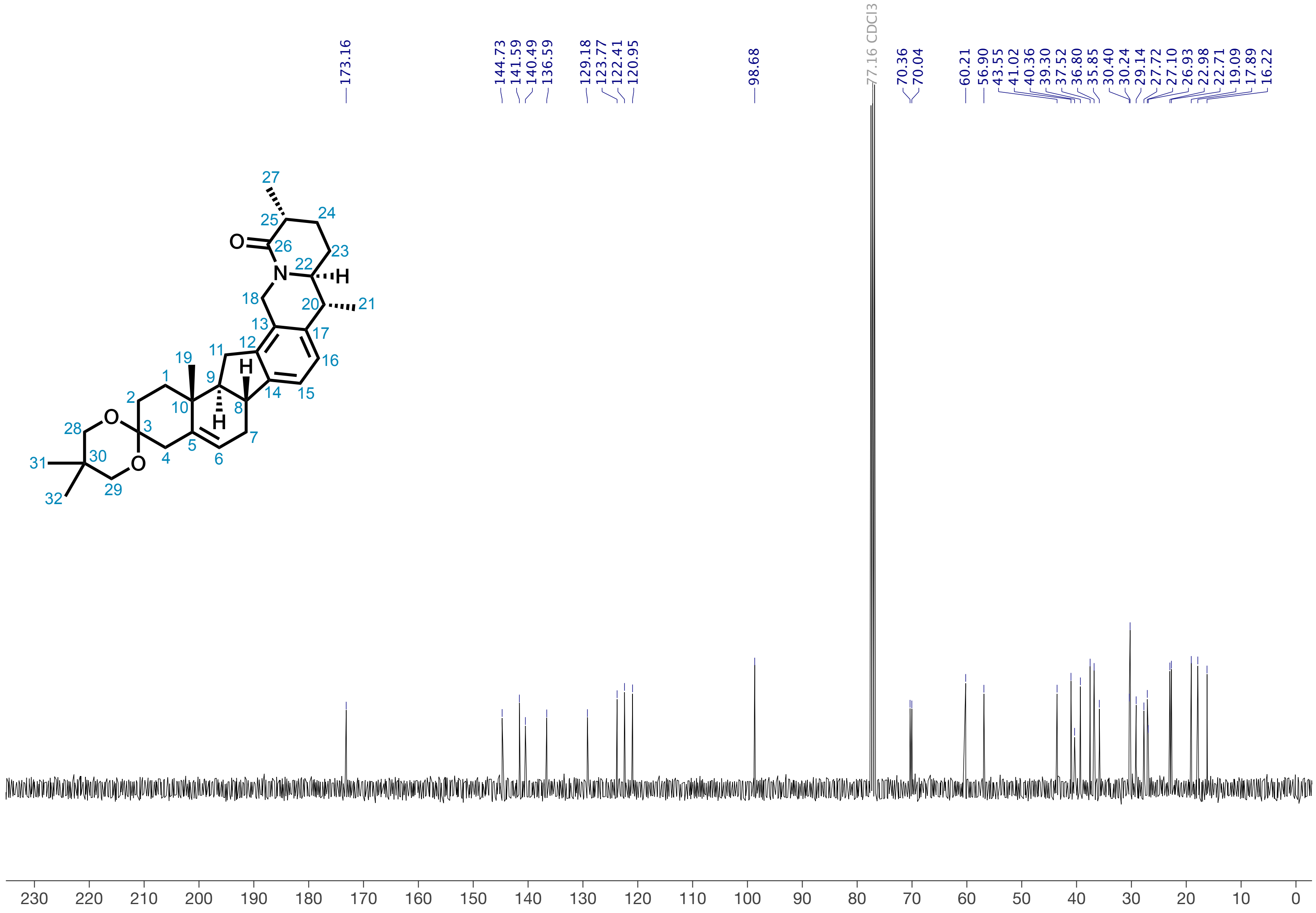

Figure S98. ^{13}C NMR (101 MHz, $CDCl_3$) of **α-methyl lactam 29**.

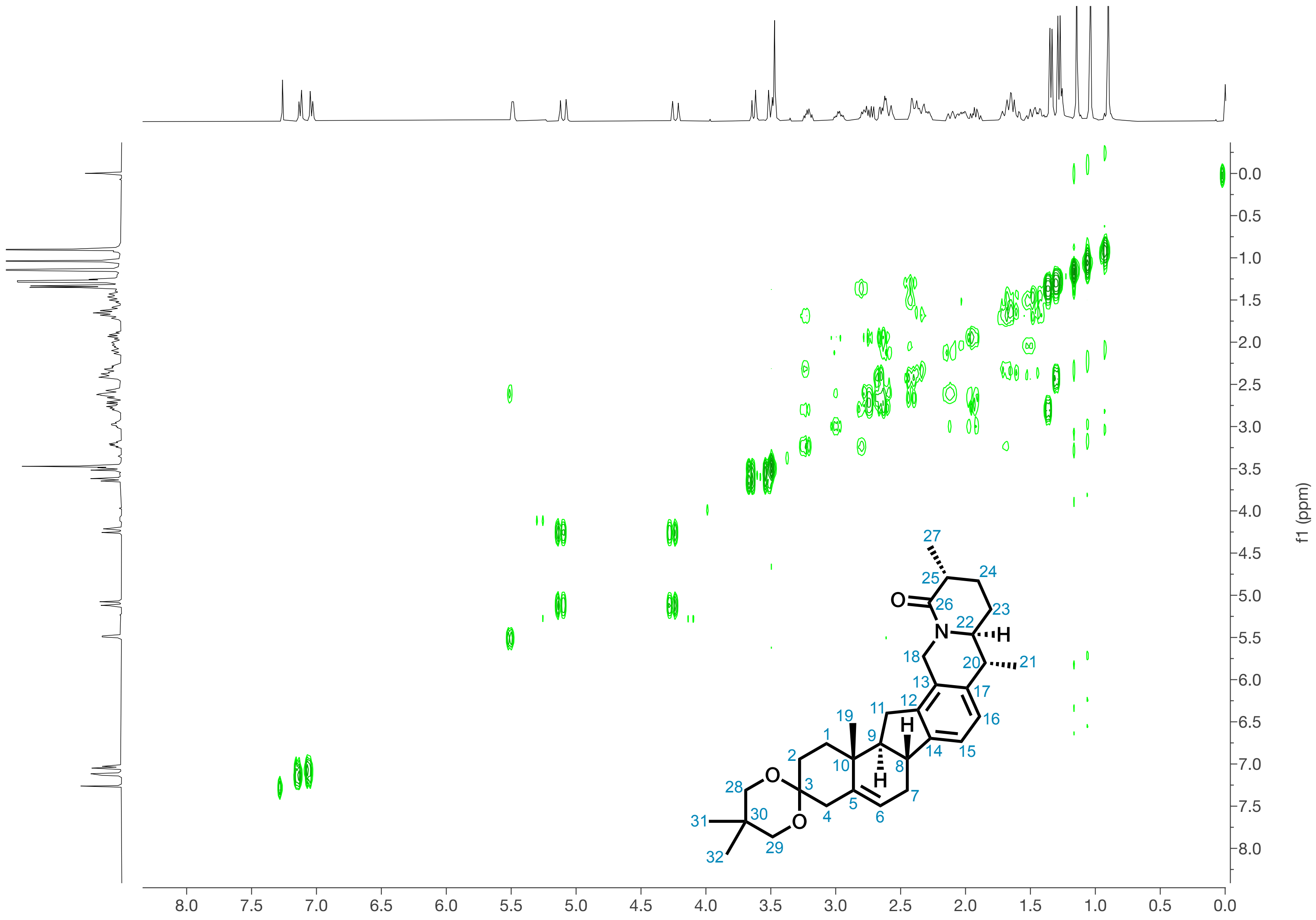

Figure S99. ^1H–^1H COSY (400 MHz, $CDCl_3$) of **α-methyl lactam 29**.

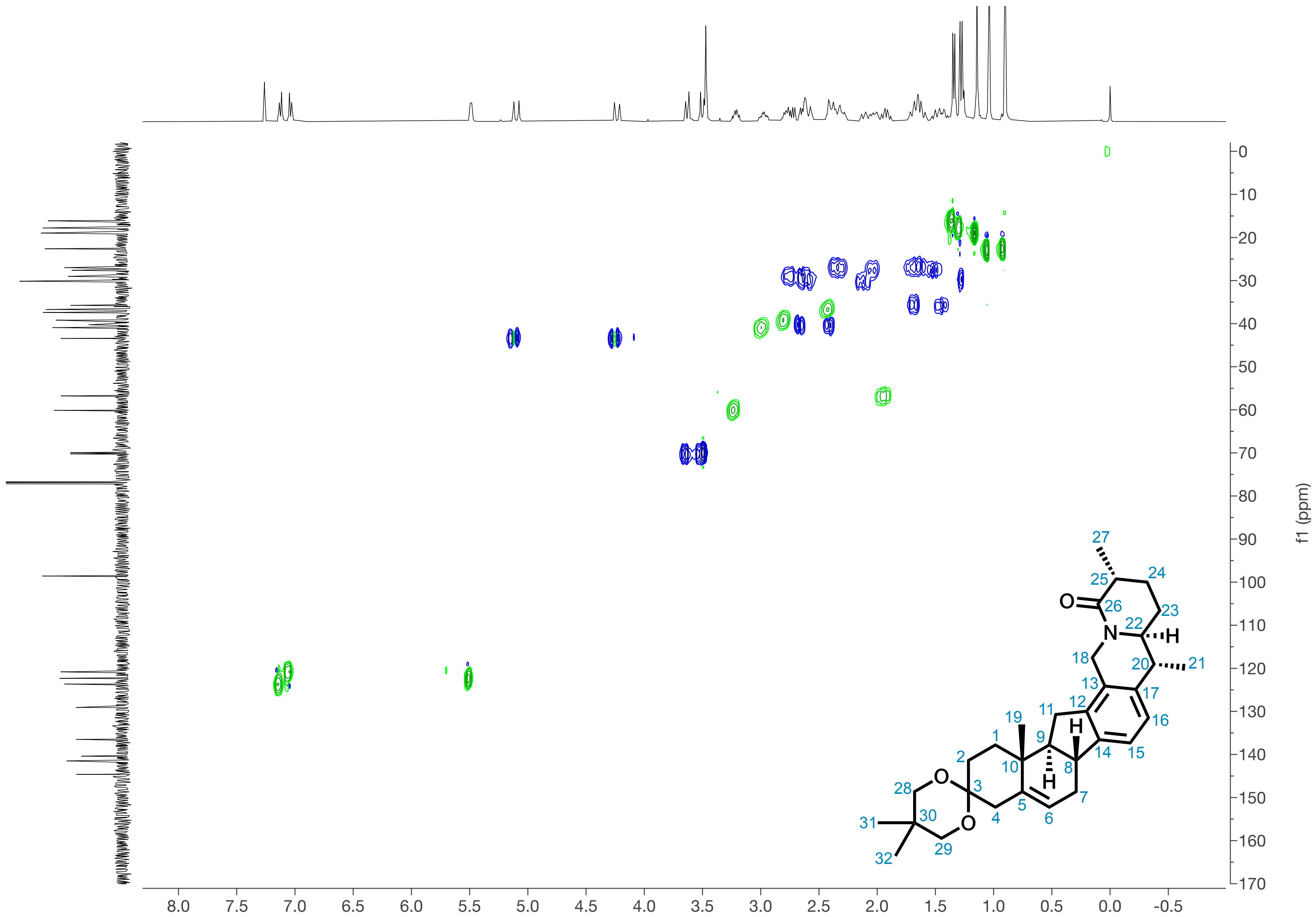

Figure S100. HSQC (400 MHz, $CDCl_3$) of **α-methyl lactam 29**.

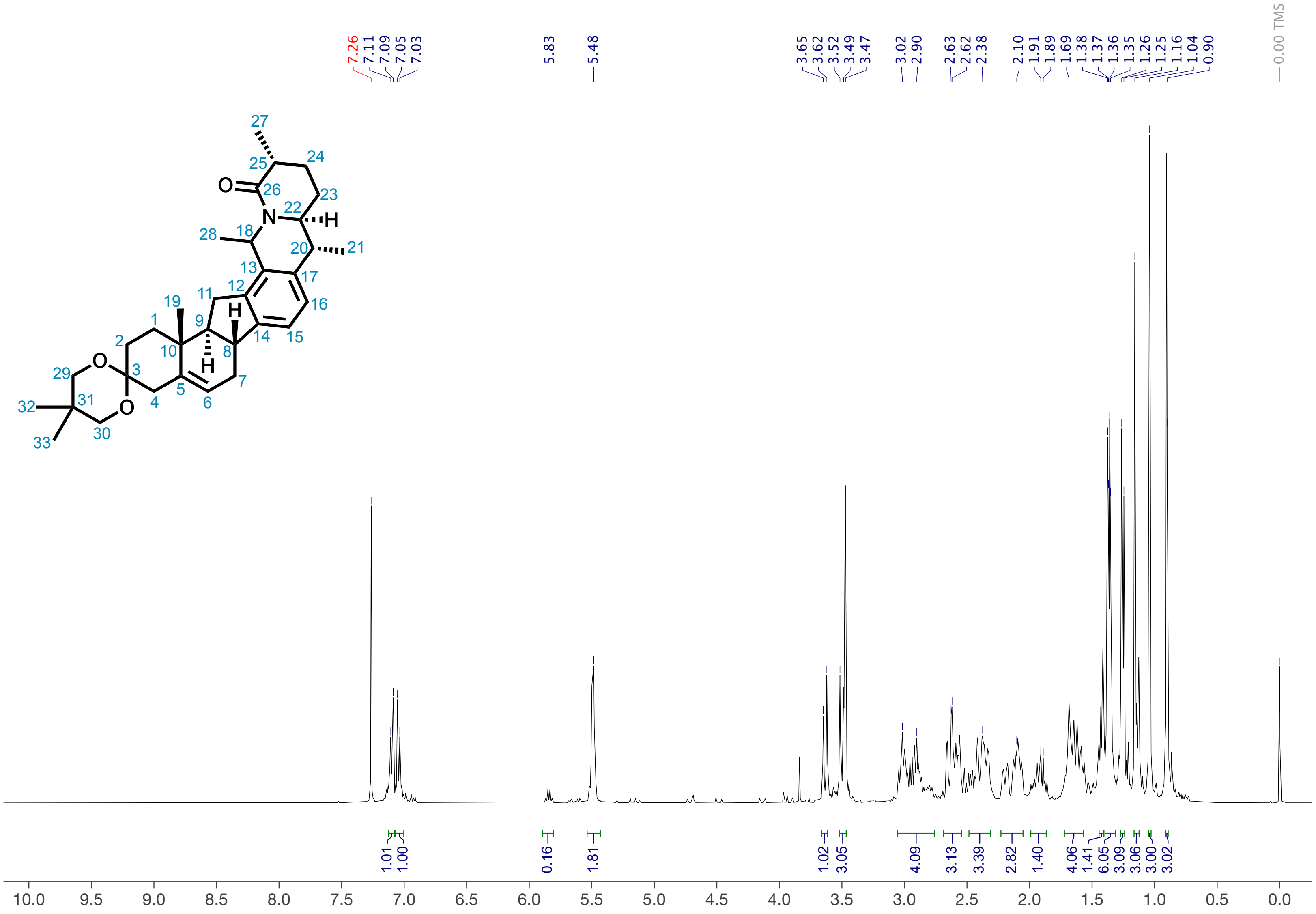

Figure S101. ^{1}H NMR (400 MHz, $CDCl_3$) of **dimethylated side product SI-13**.

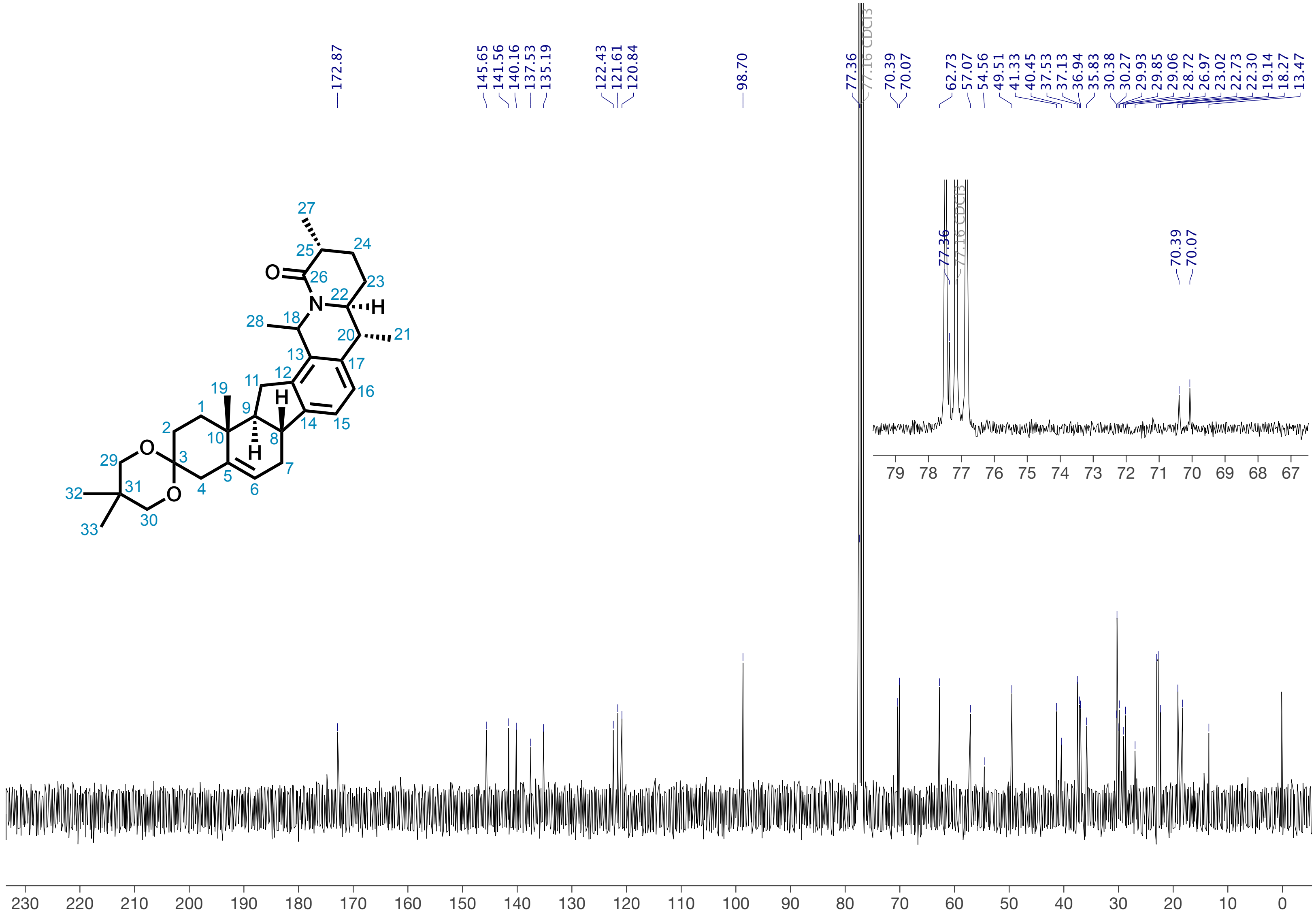

Figure S102. ^{13}C NMR (101 MHz, $CDCl_3$) of **dimethylated side product SI-13**.

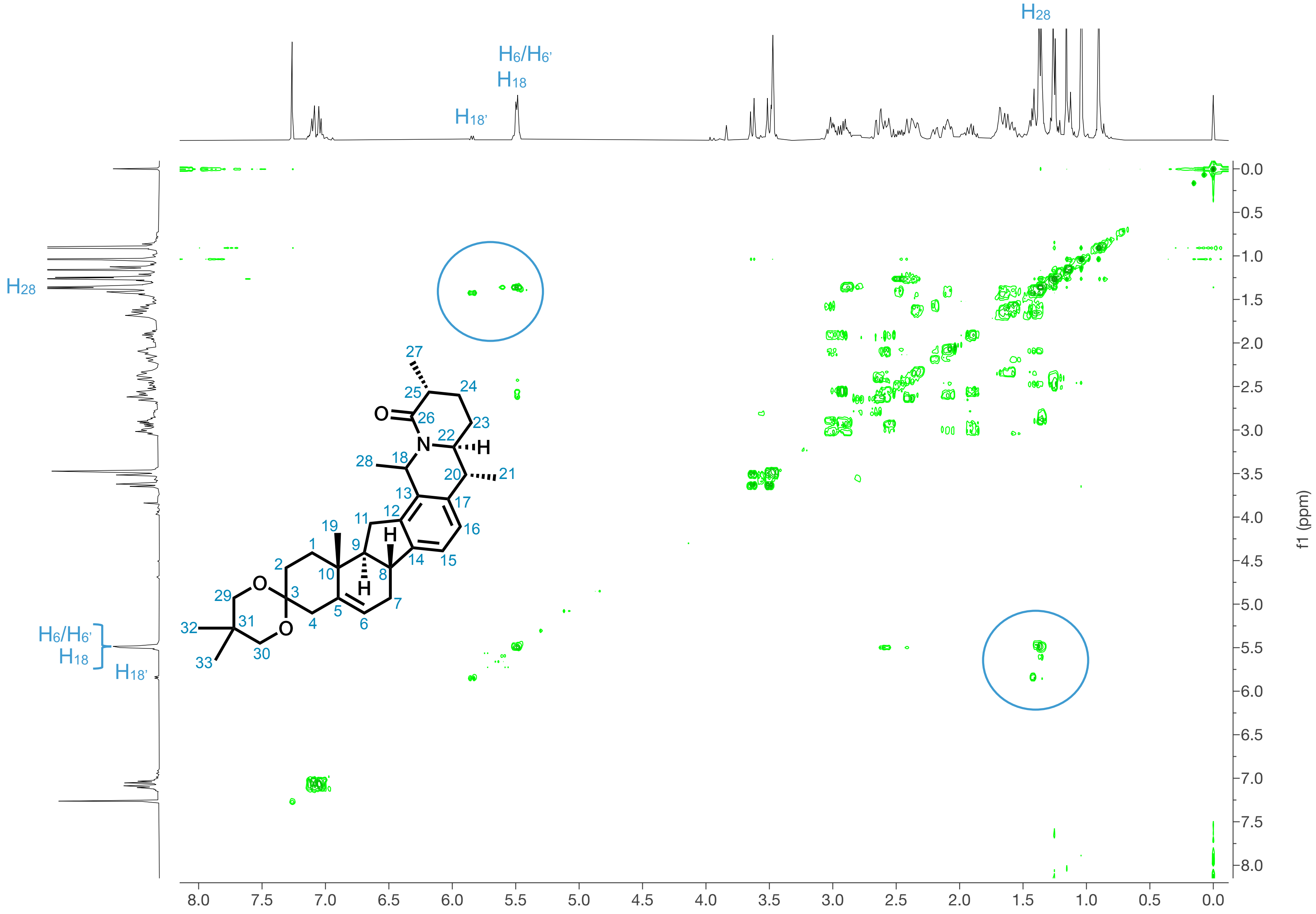

Figure S103. ^1H–^1H COSY (400 MHz, $CDCl_3$) of **dimethylated side product SI-13/SI-13'**.

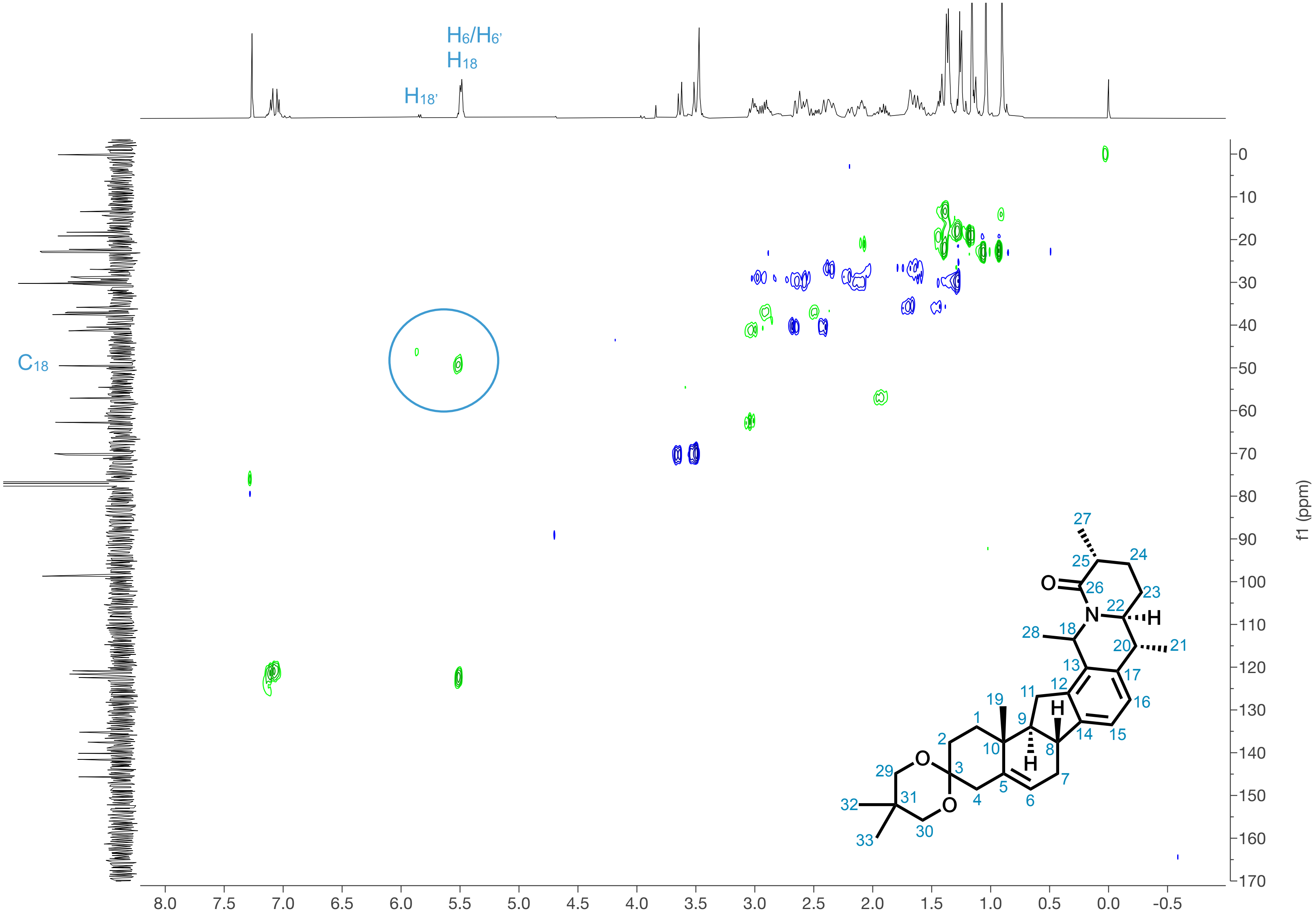

Figure S104. HSQC (400 MHz, $CDCl_3$) of **dimethylated side product SI-13**.

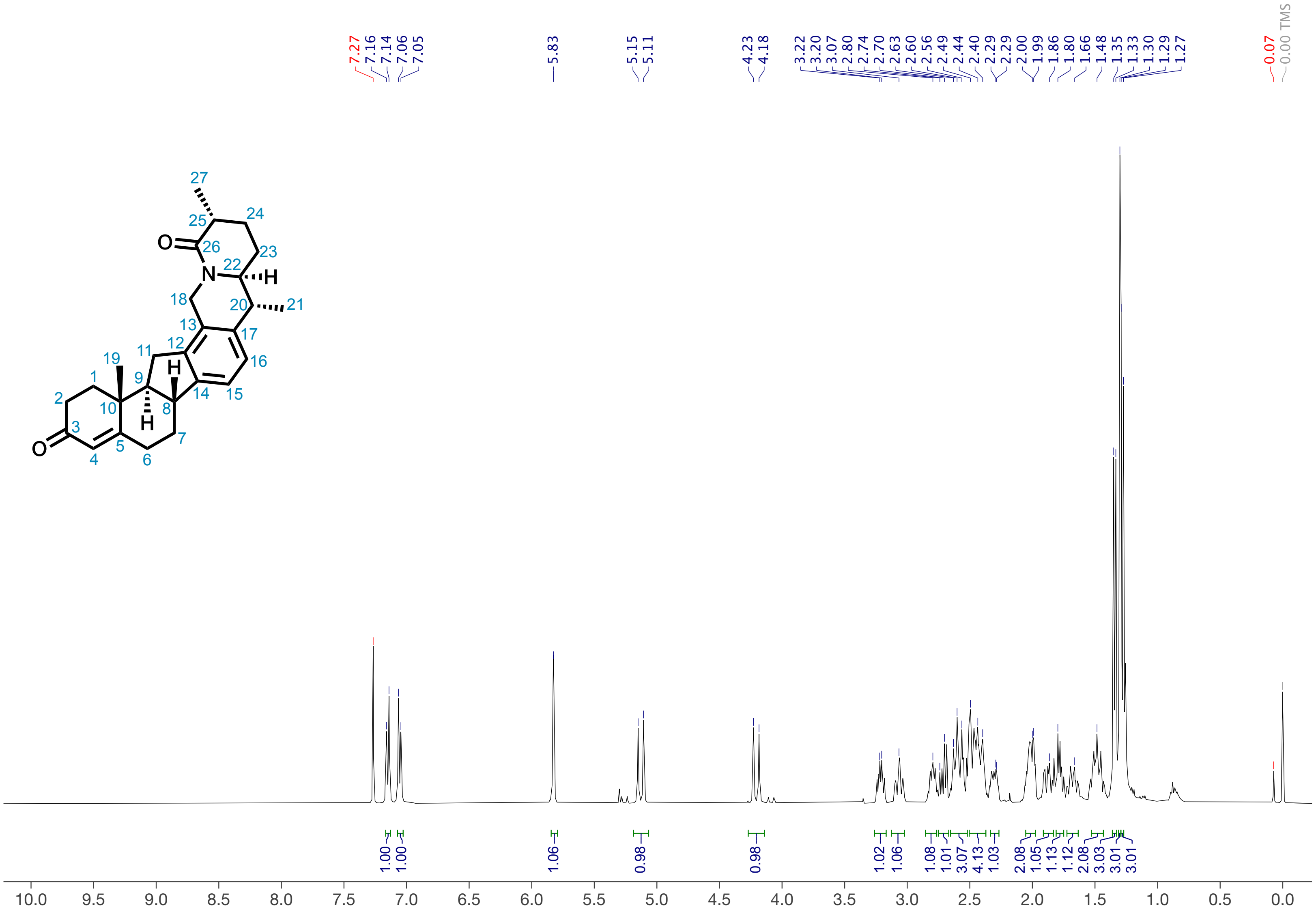

Figure S105. ^{1}H NMR (400 MHz, $CDCl_3$) of **enone 30**.

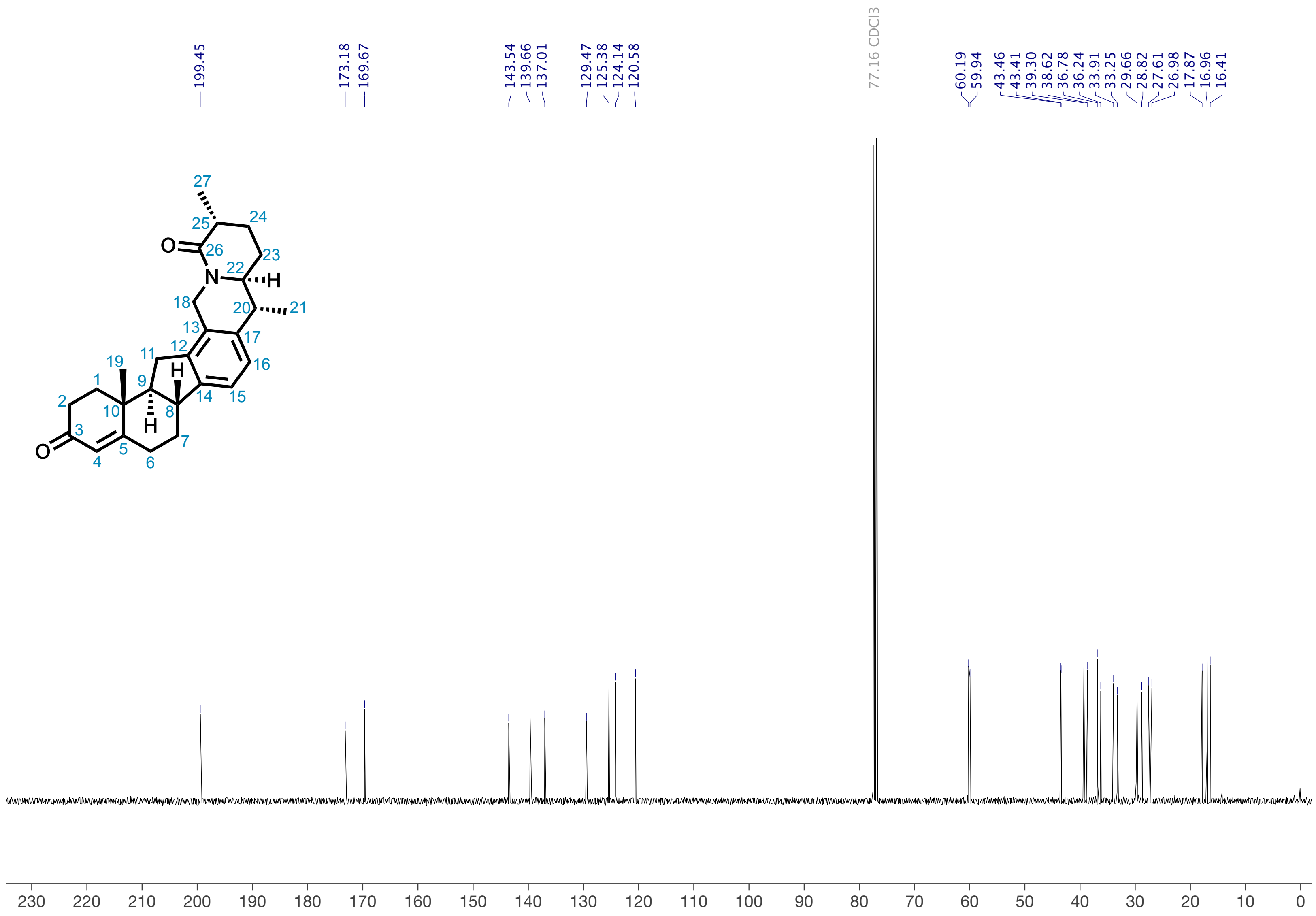

Figure S106. ^{13}C NMR (101 MHz, $CDCl_3$) of **enone 30**.

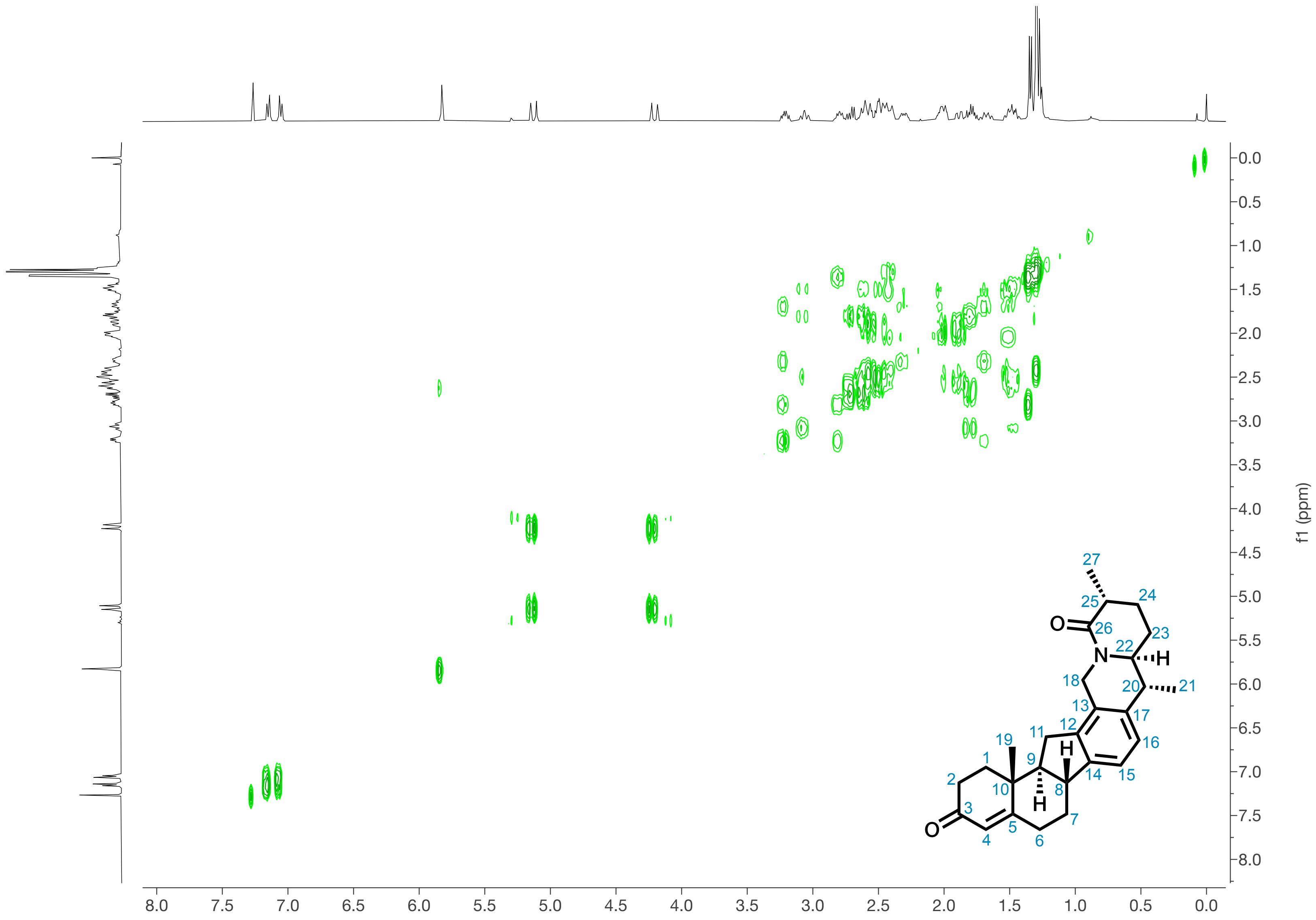

Figure S107. ^{1}H–^{1}H COSY (400 MHz, $CDCl_3$) of **enone 30**.

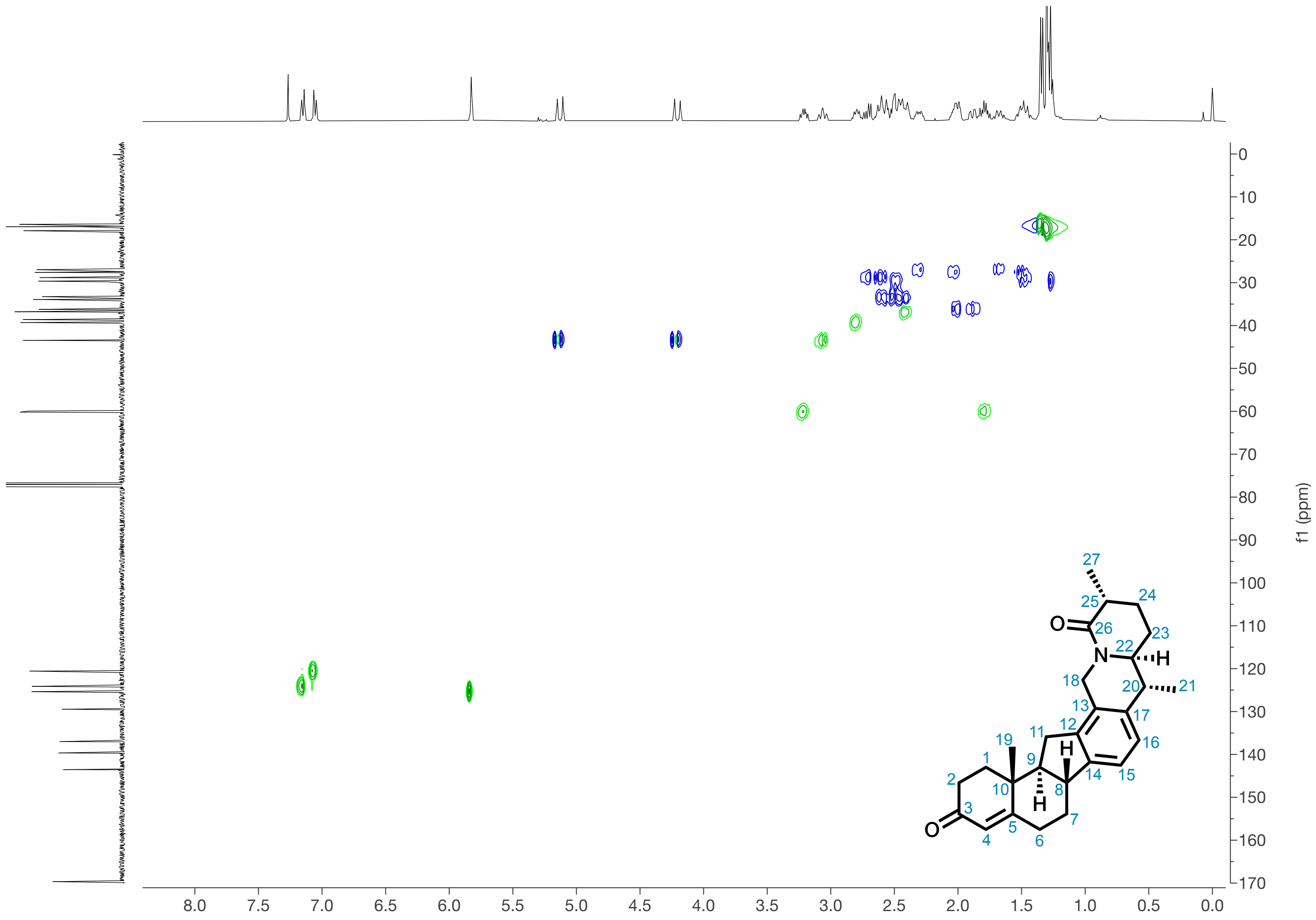

Figure S108. HSQC (400 MHz, $CDCl_3$) of **enone 30**.

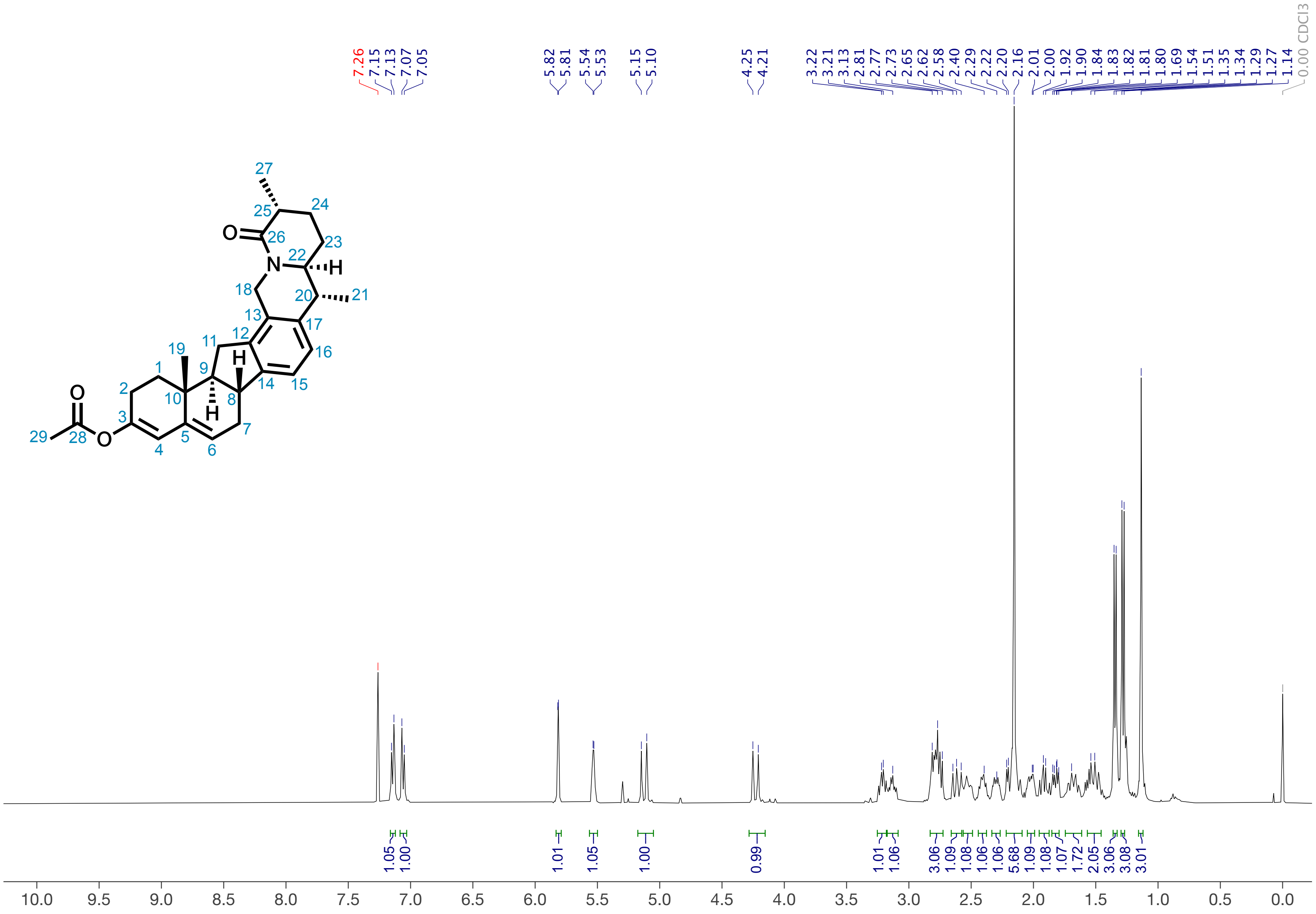

Figure S109. ^{1}H NMR (400 MHz, $CDCl_3$) of **dienol acetate 31**.

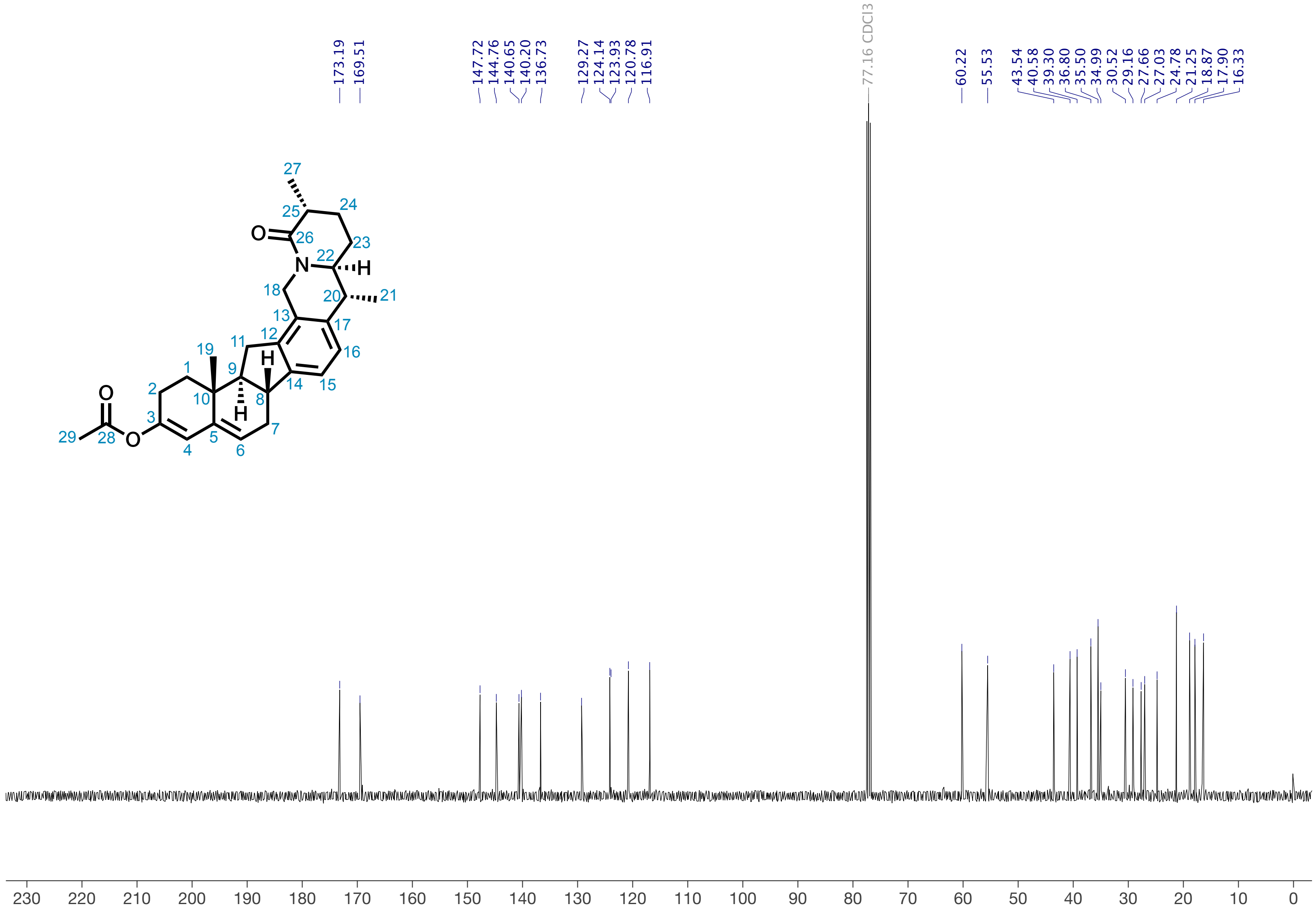

Figure S110. ^{13}C NMR (101 MHz, $CDCl_3$) of **dienol acetate 31**.

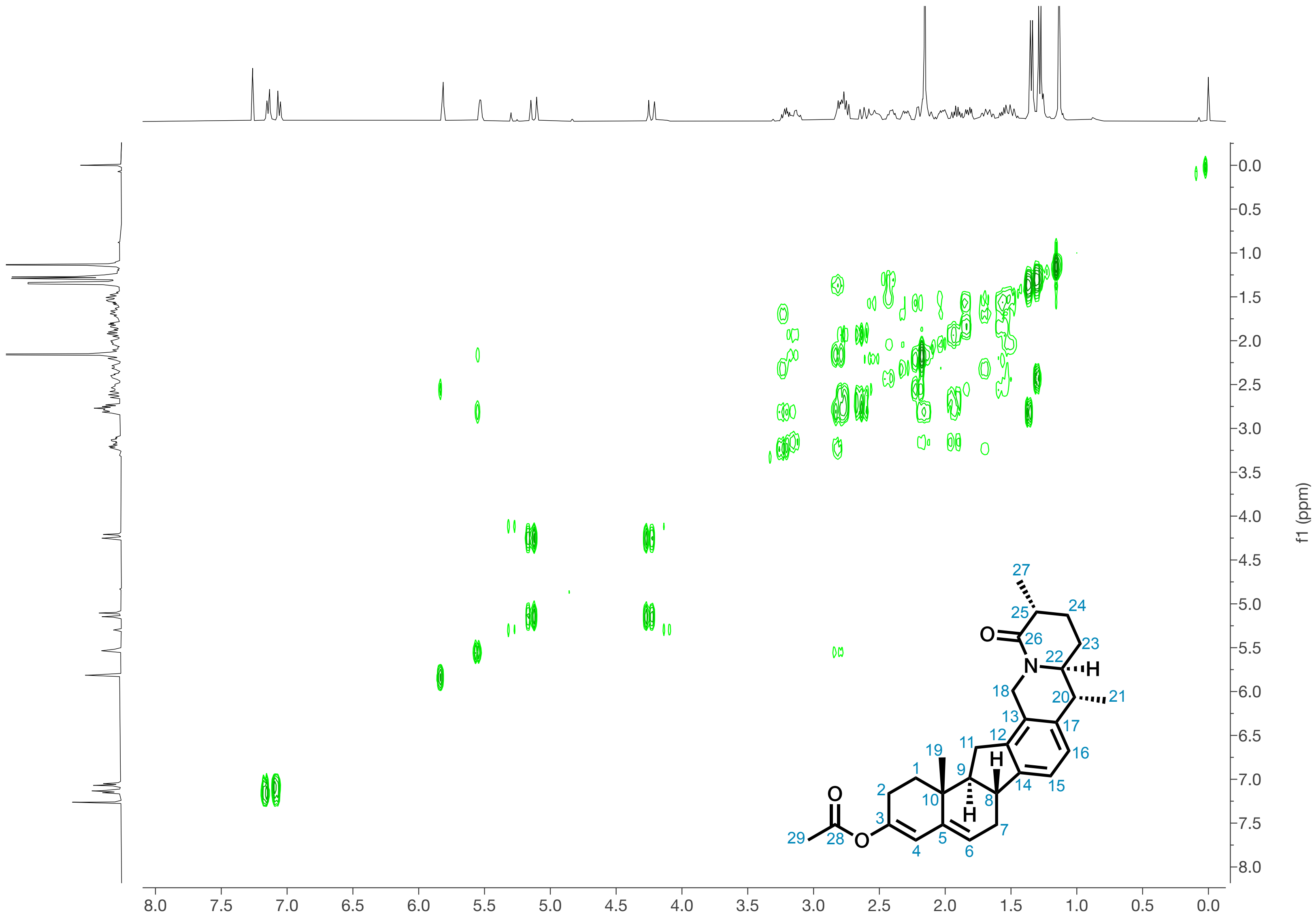

Figure S111. ^1H–^1H COSY (400 MHz, $CDCl_3$) of **dienol acetate 31**.

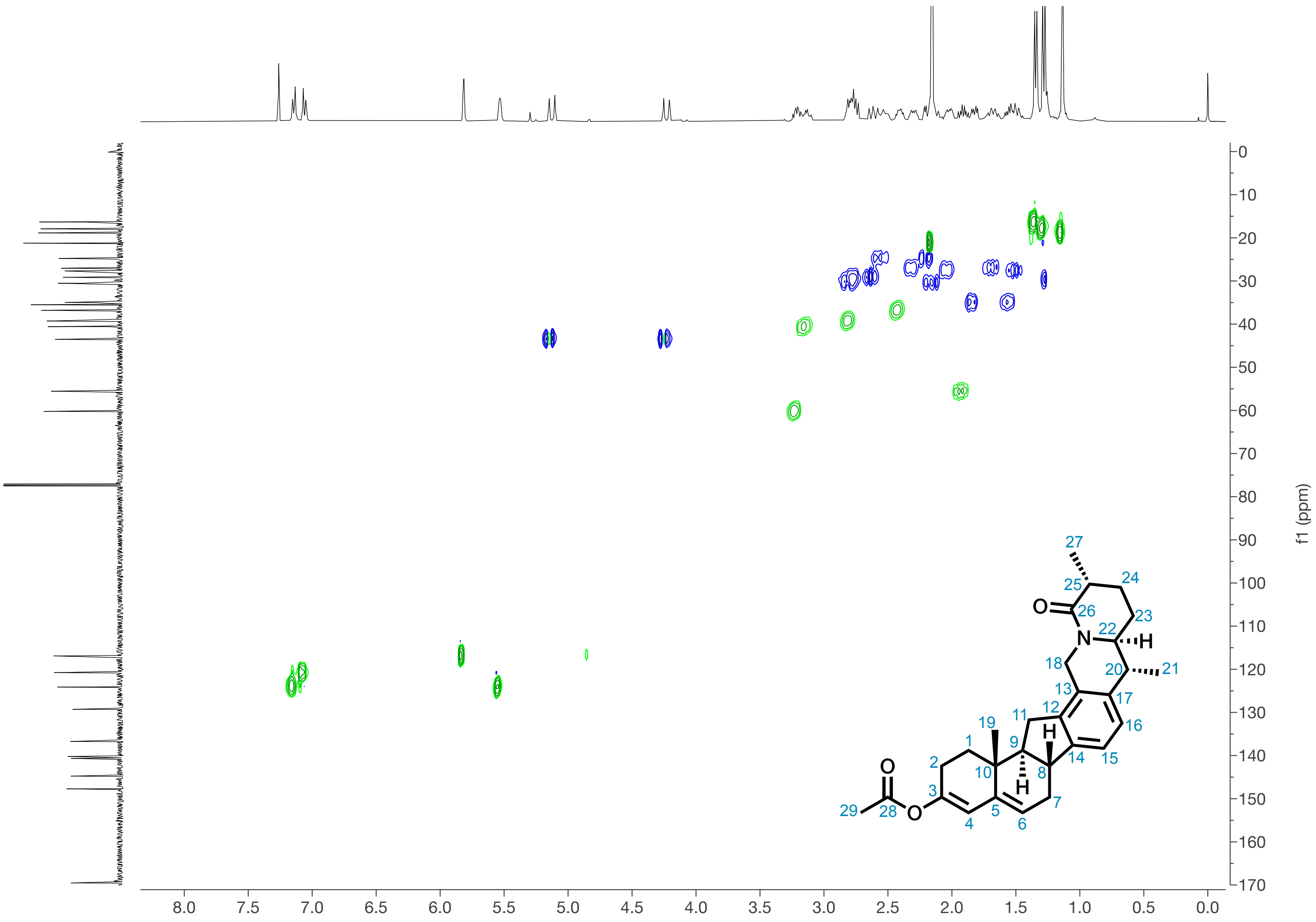

Figure S112. HSQC (400 MHz, $CDCl_3$) of **dienol acetate 31**.

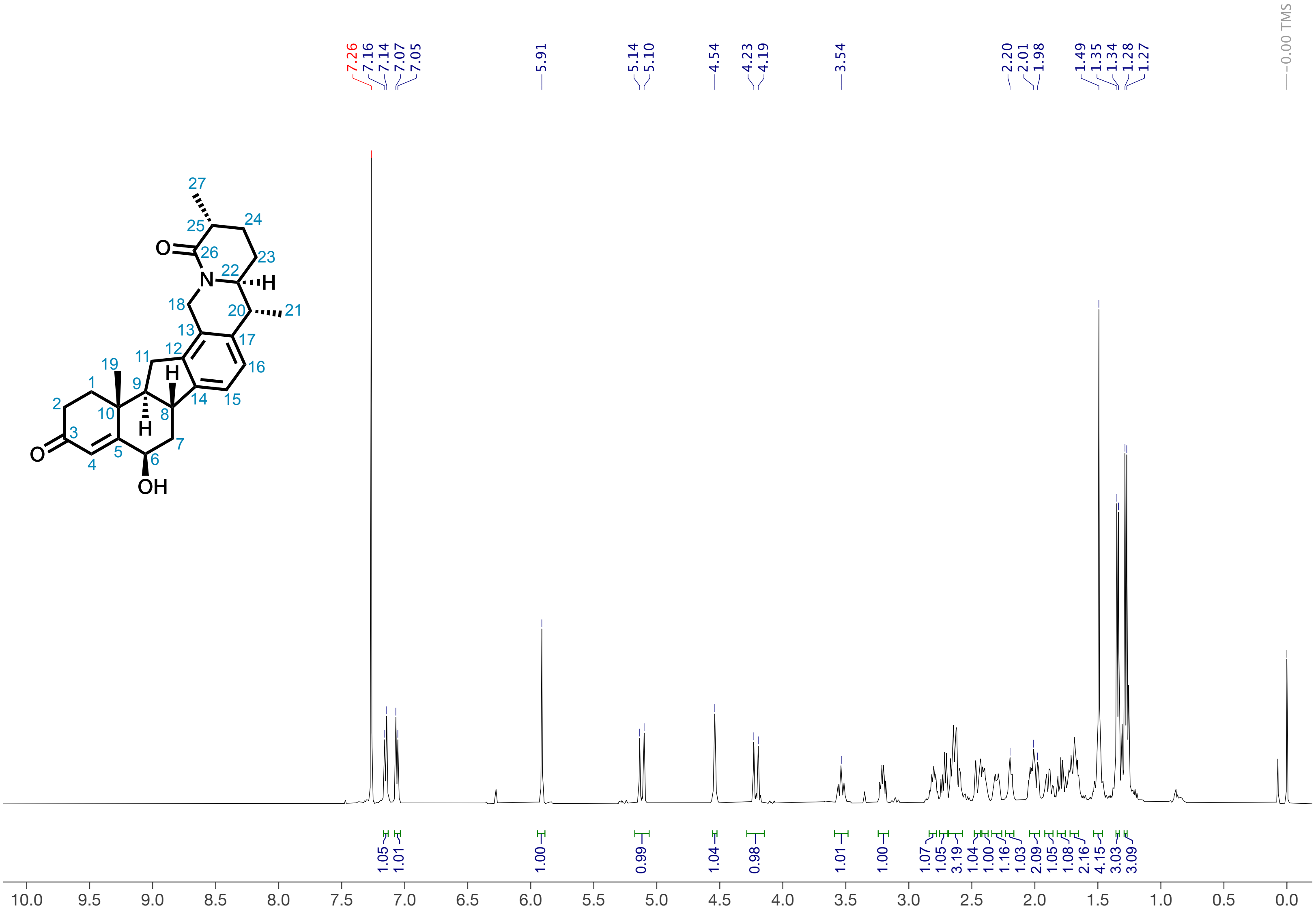

Figure S113. ^{1}H NMR (400 MHz, $CDCl_3$) of **γ-hydroxy enone 32**.

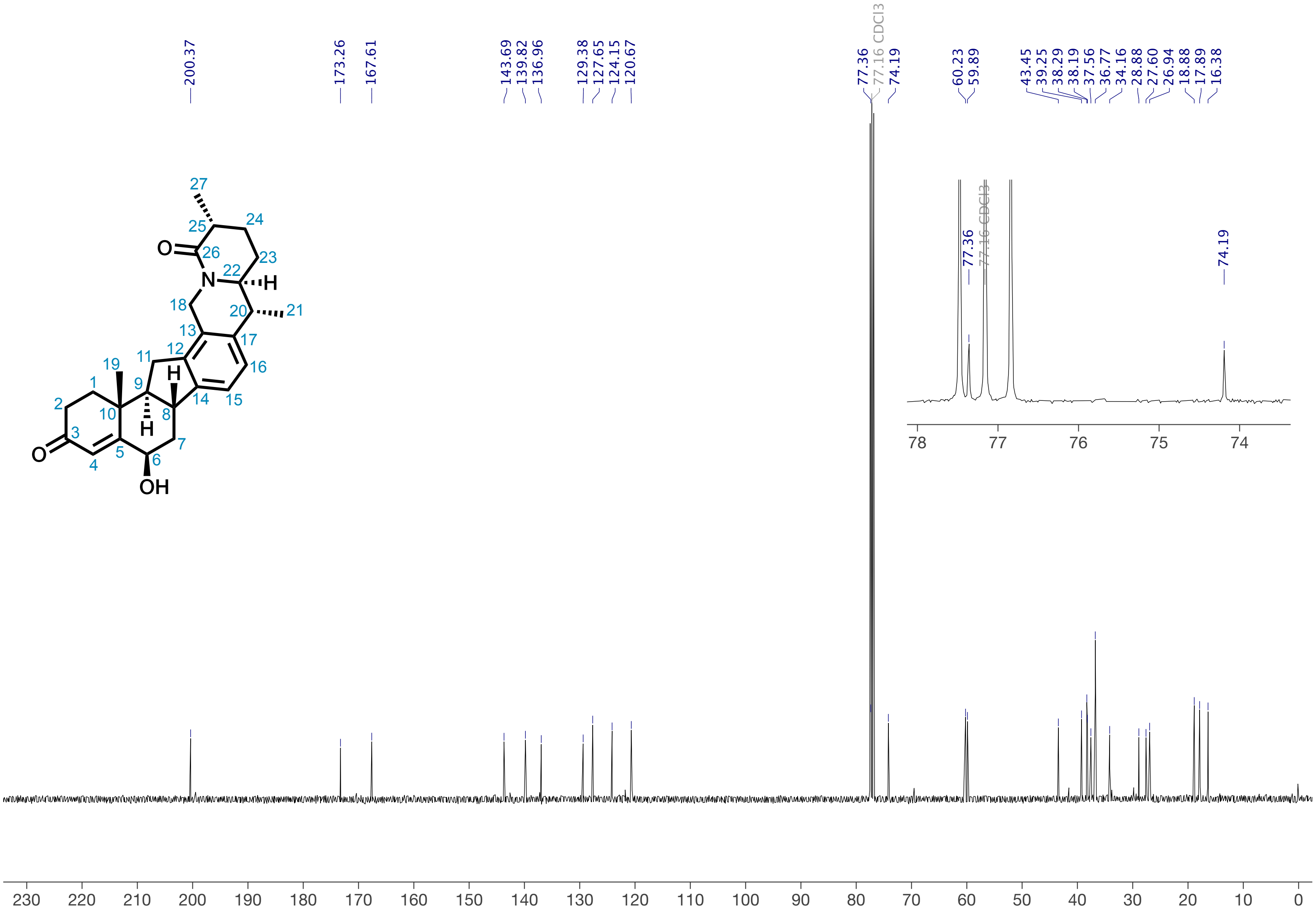

Figure S114. ^{13}C NMR (101 MHz, $CDCl_3$) of **γ-hydroxy enone 32**.

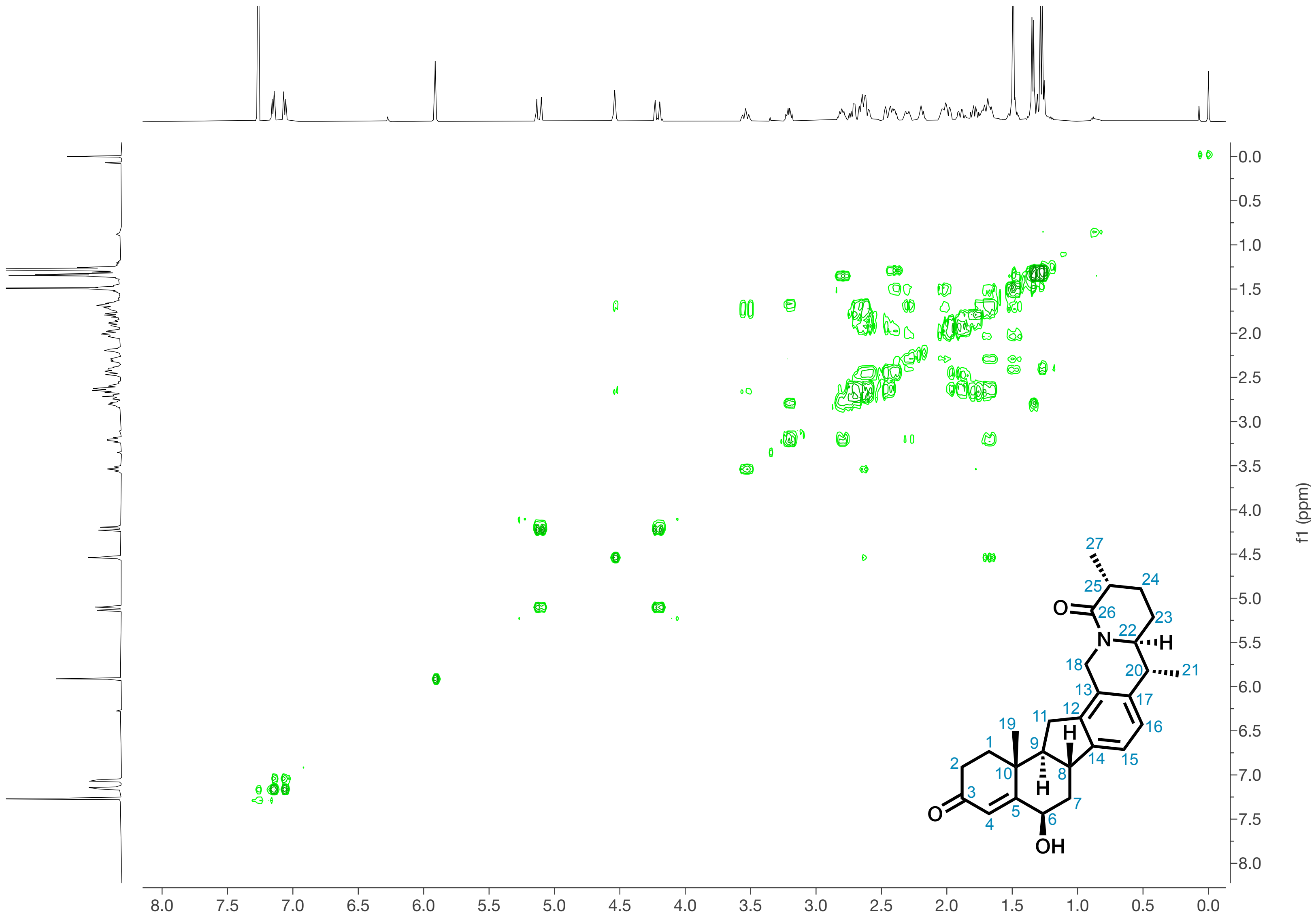

Figure S115. ^1H–^1H COSY (400 MHz, $CDCl_3$) of **γ-hydroxy enone 32**.

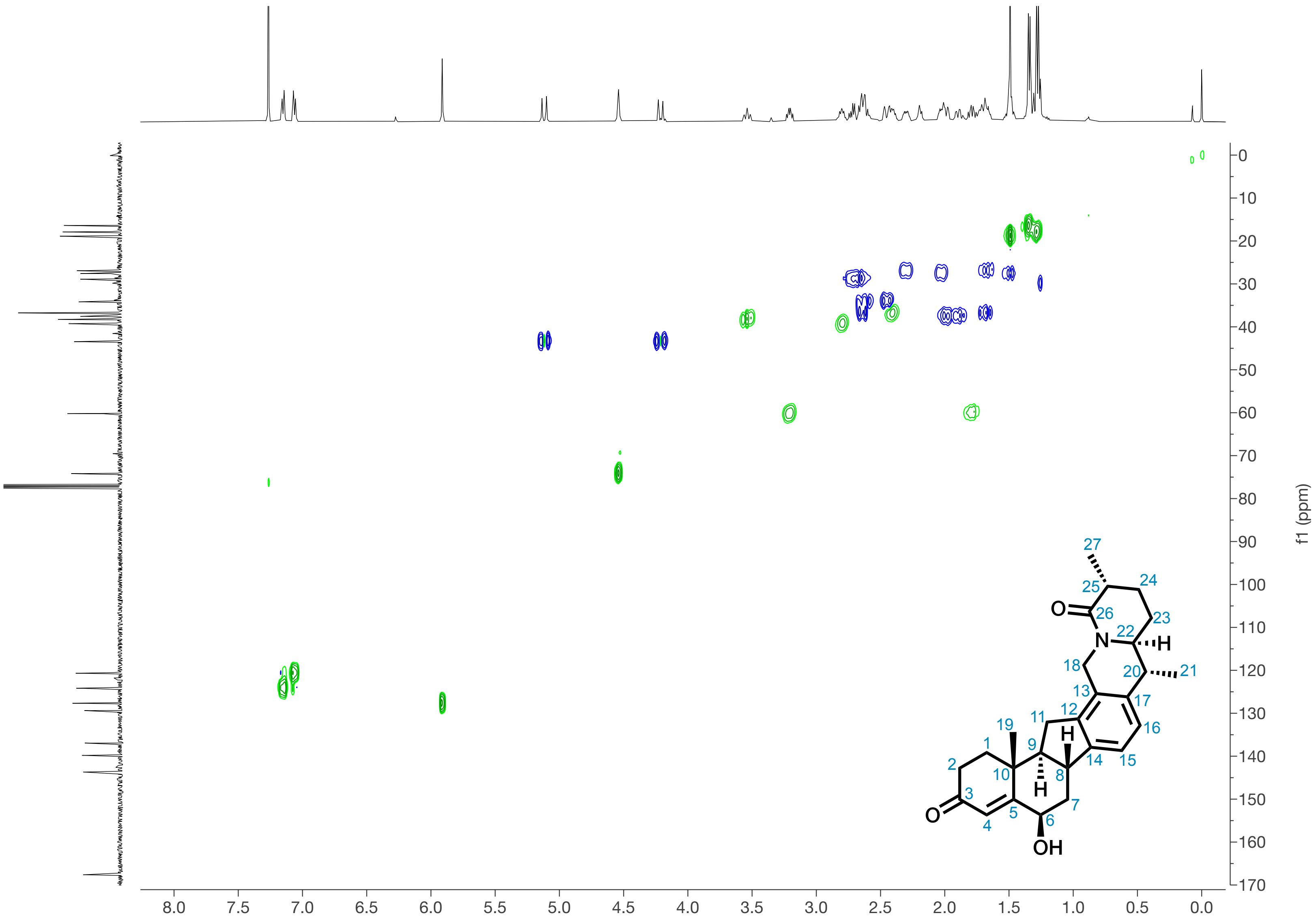

Figure S116. HSQC (400 MHz, $CDCl_3$) of **γ-hydroxy enone 32**.

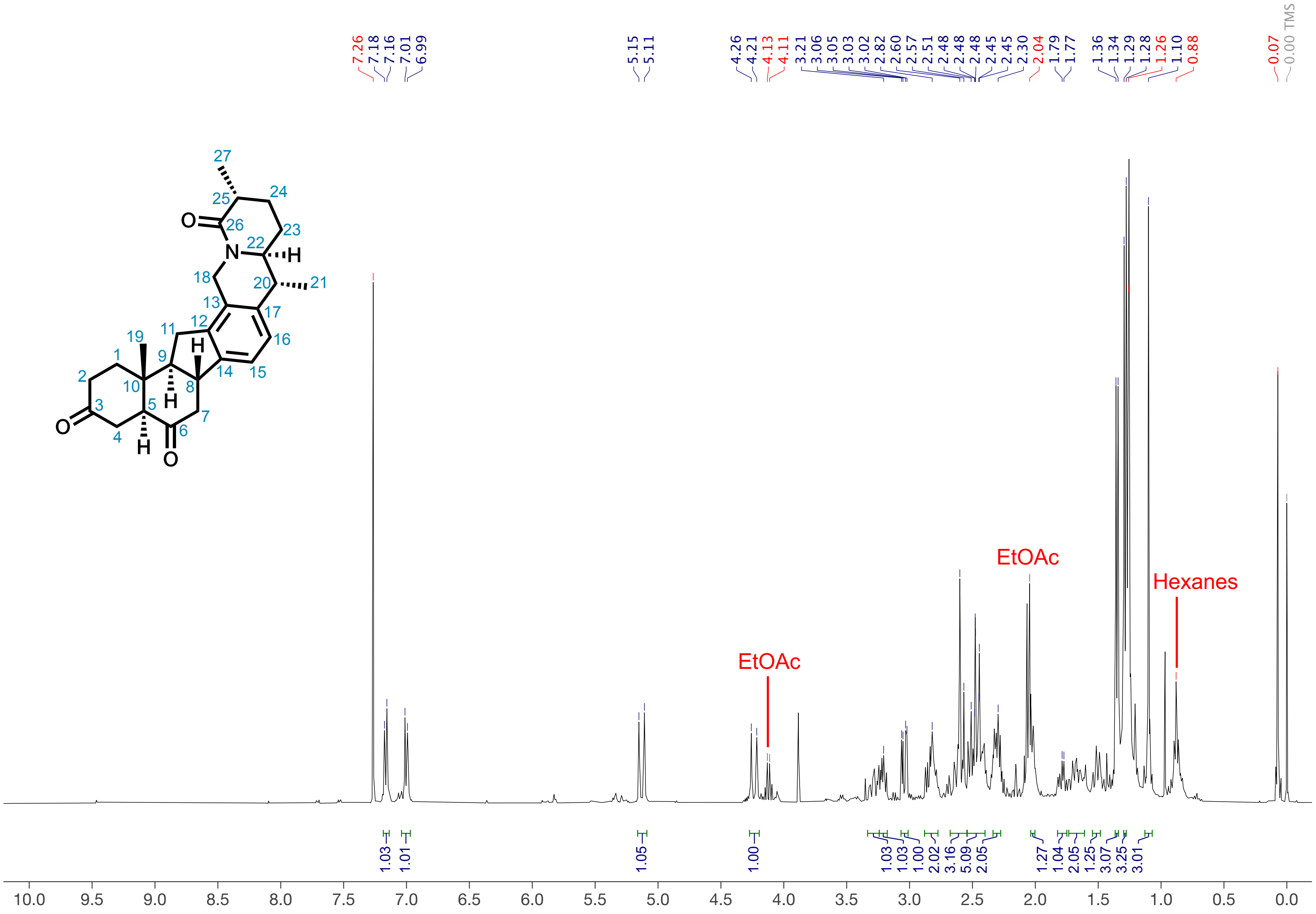

Figure S117. ^{1}H NMR (400 MHz, $CDCl_3$) of unpurified **γ-diketone SI-15**.

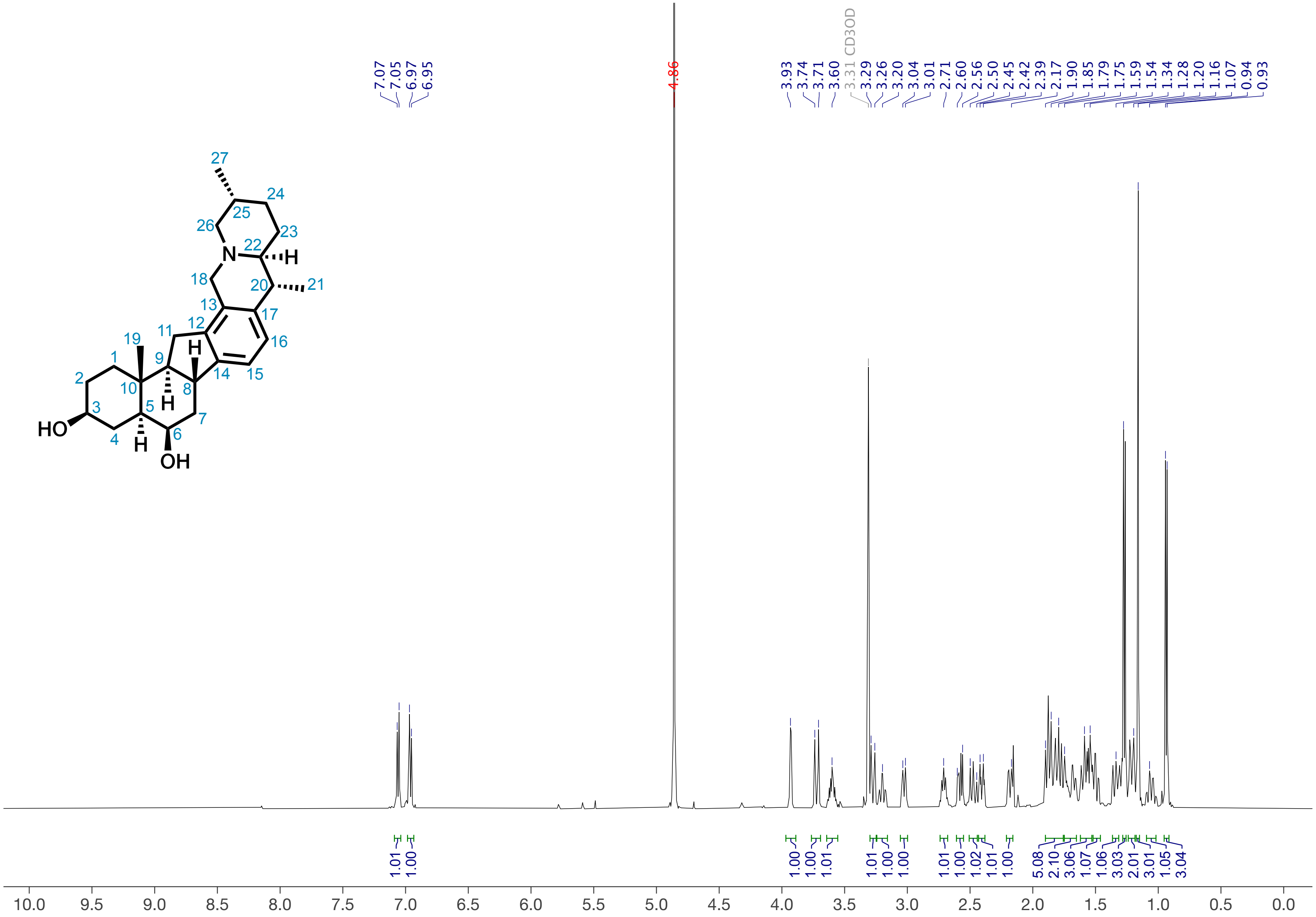

Figure S118. ^{1}H NMR (500 MHz, CD_3OD) of **heilonine**.

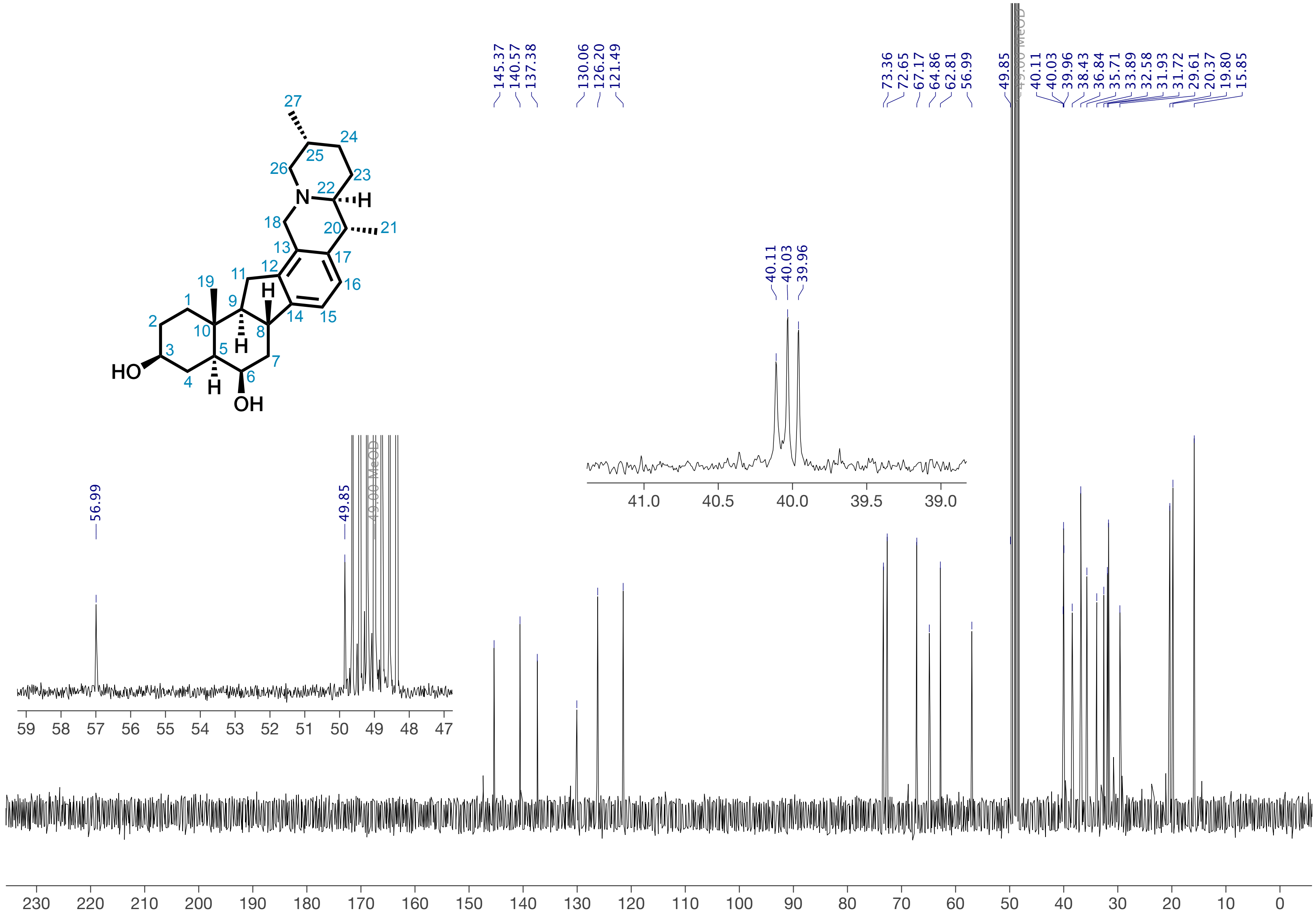

Figure S119. ^{13}C NMR (101 MHz, CD_3OD) of **heilonine**.

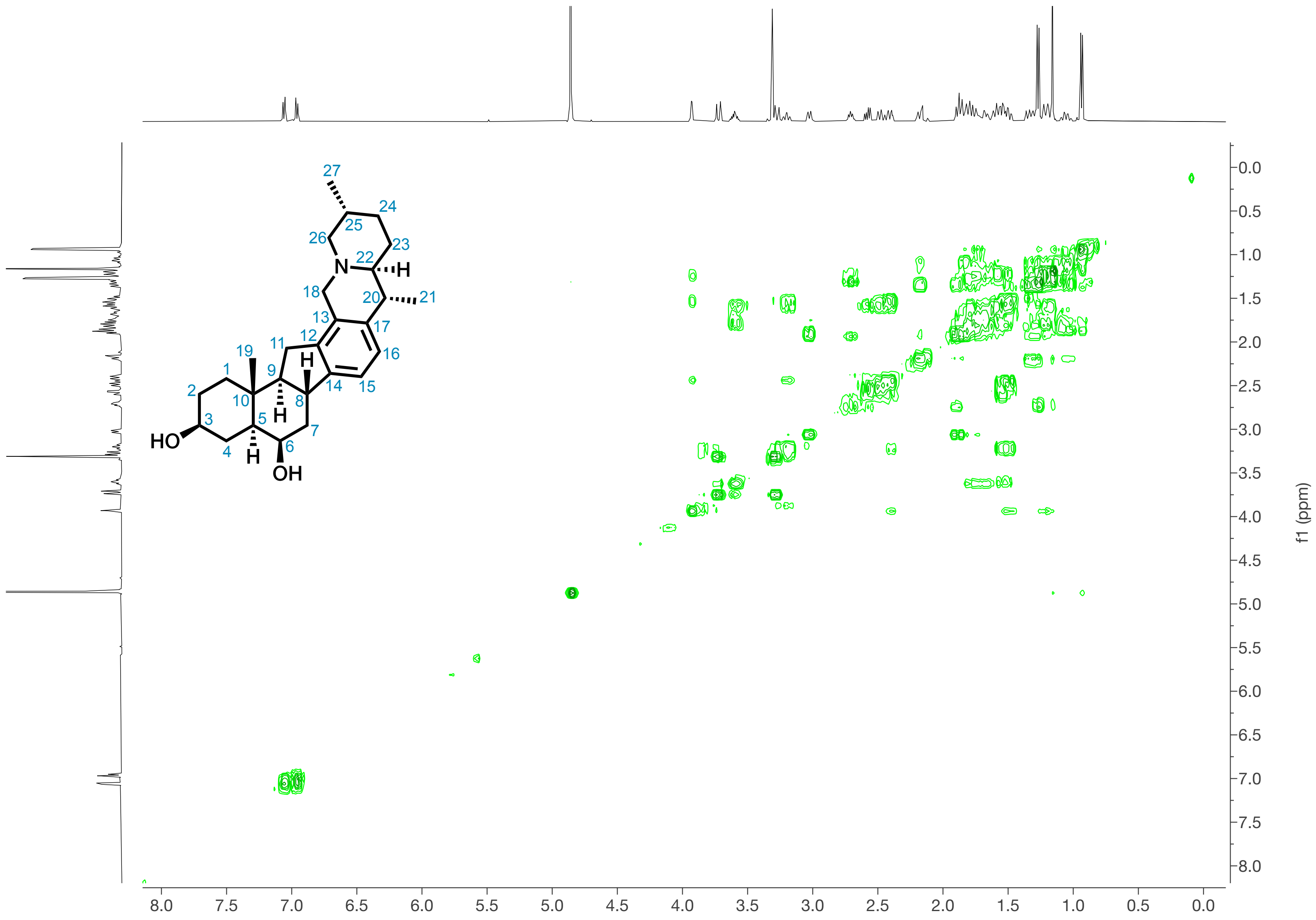

Figure S120. ¹H–¹H COSY (400 MHz, CD_3OD) of **heilonine**.

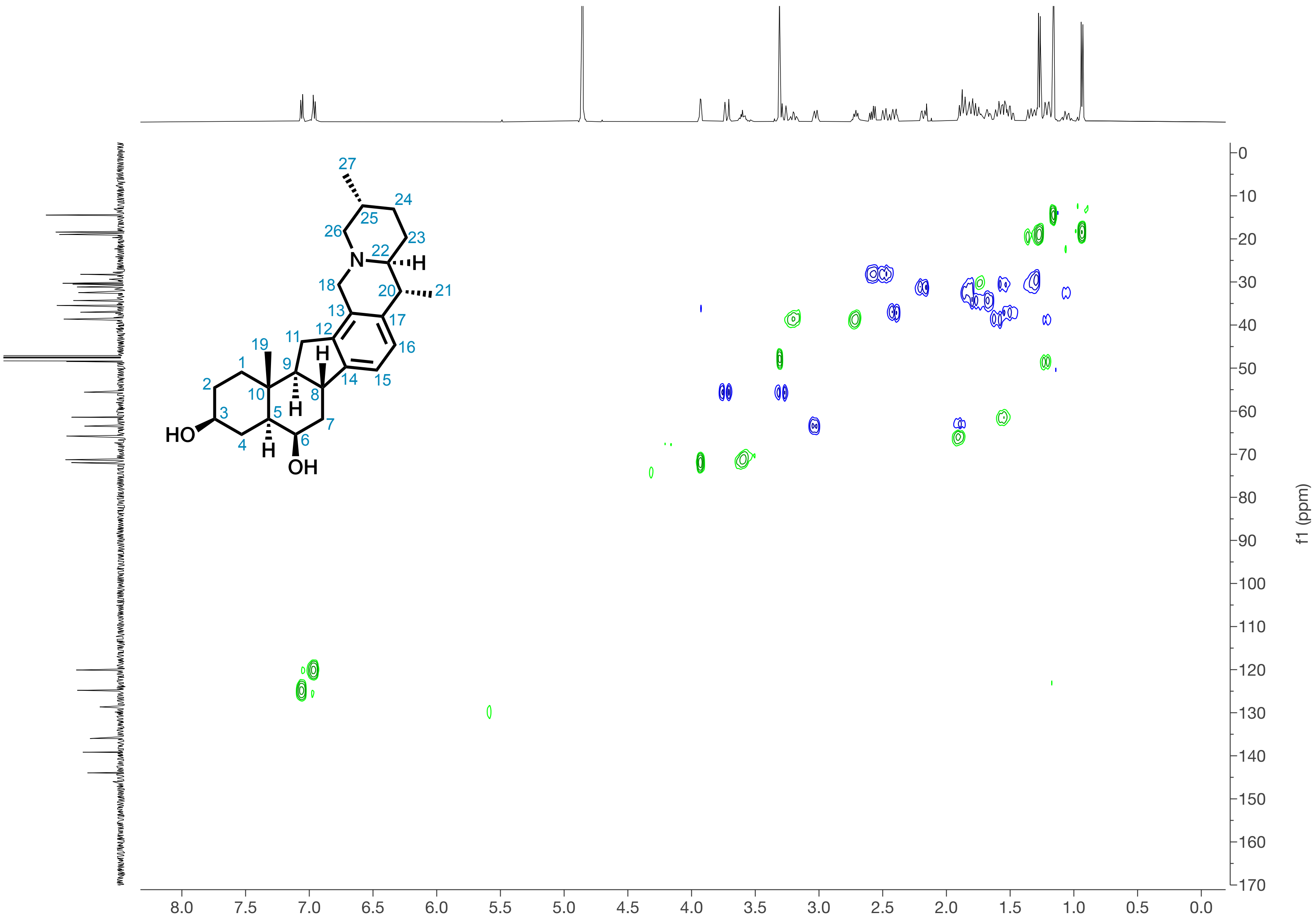

Figure S121. HSQC (400 MHz, CD_3OD) of **heilonine**.

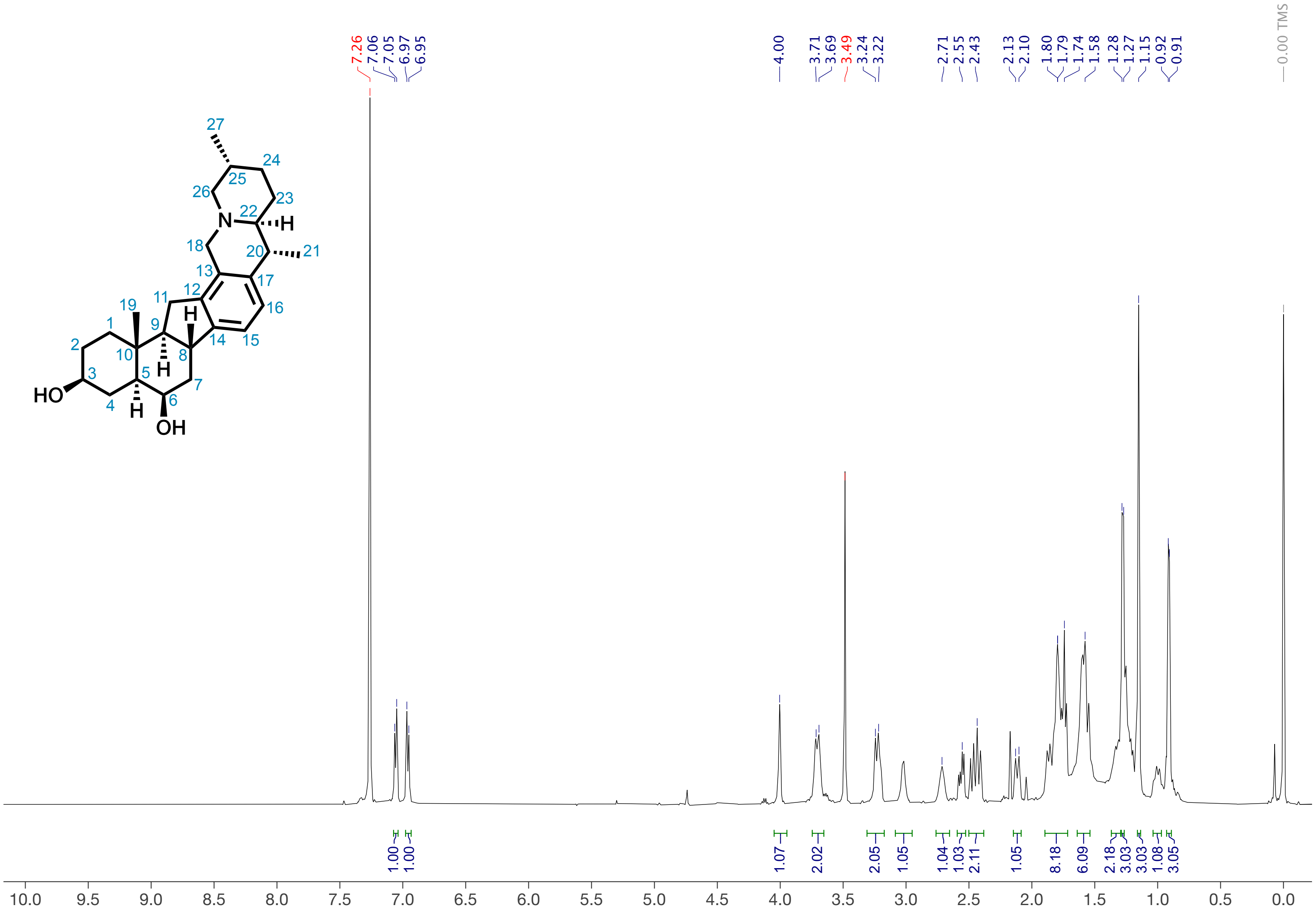

Figure S122. ^{1}H NMR (400 MHz, $CDCl_3$) of **heilonine**.

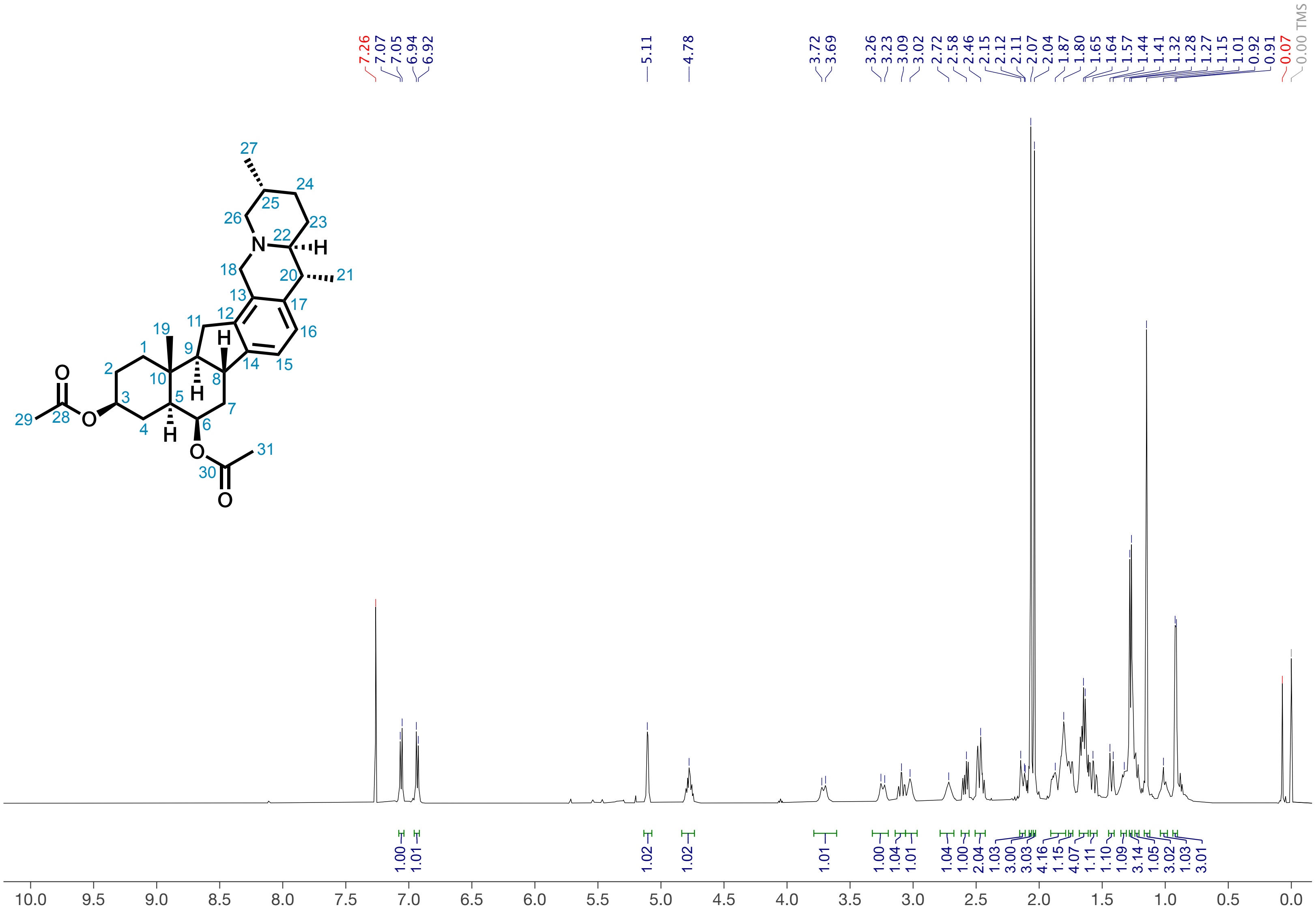

Figure S123. ^{1}H NMR (500 MHz, $CDCl_3$) of **heilonine diacetate**.

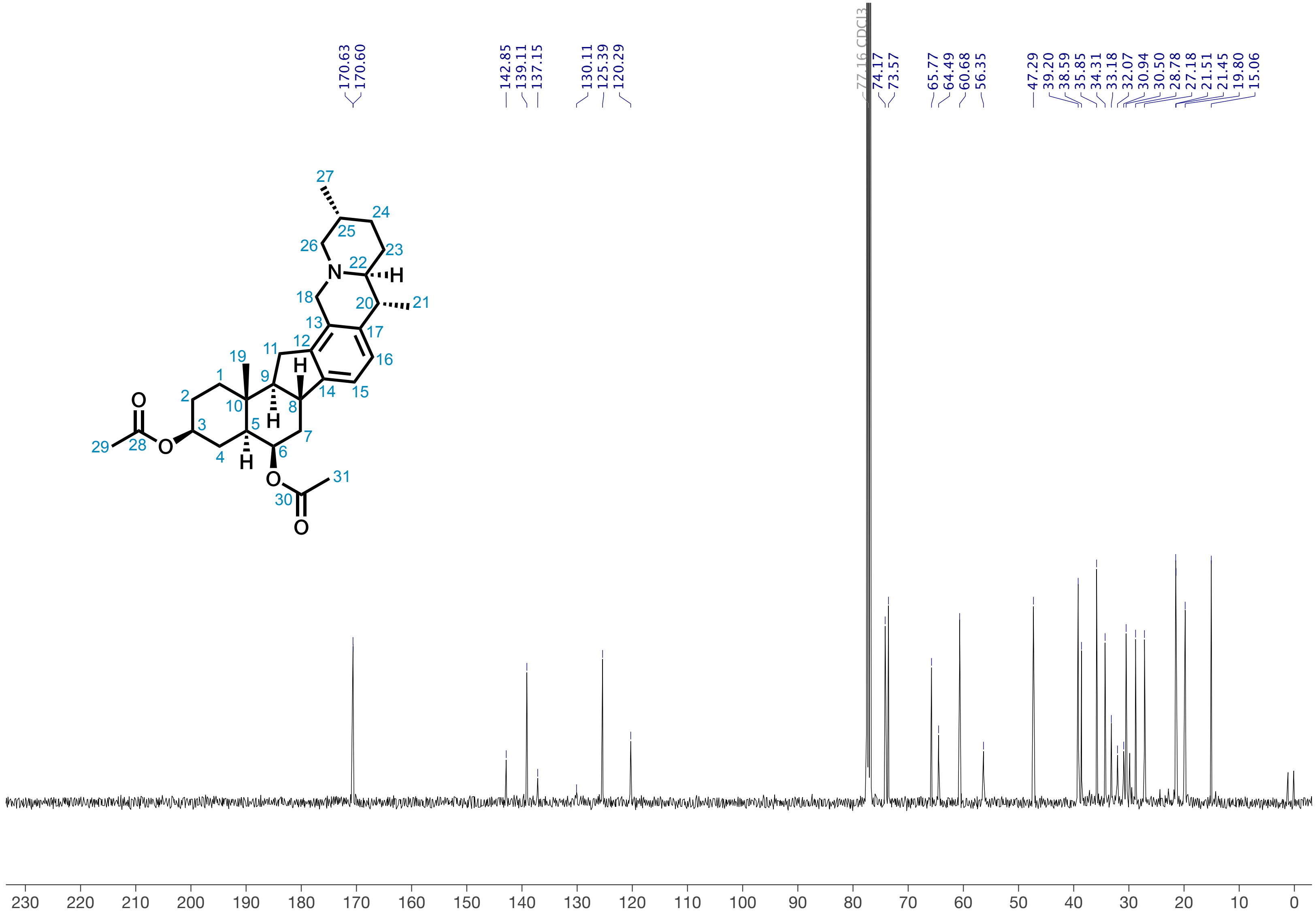

Figure S124. ^{13}C NMR (101 MHz, $CDCl_3$) of **heilonine diacetate**.

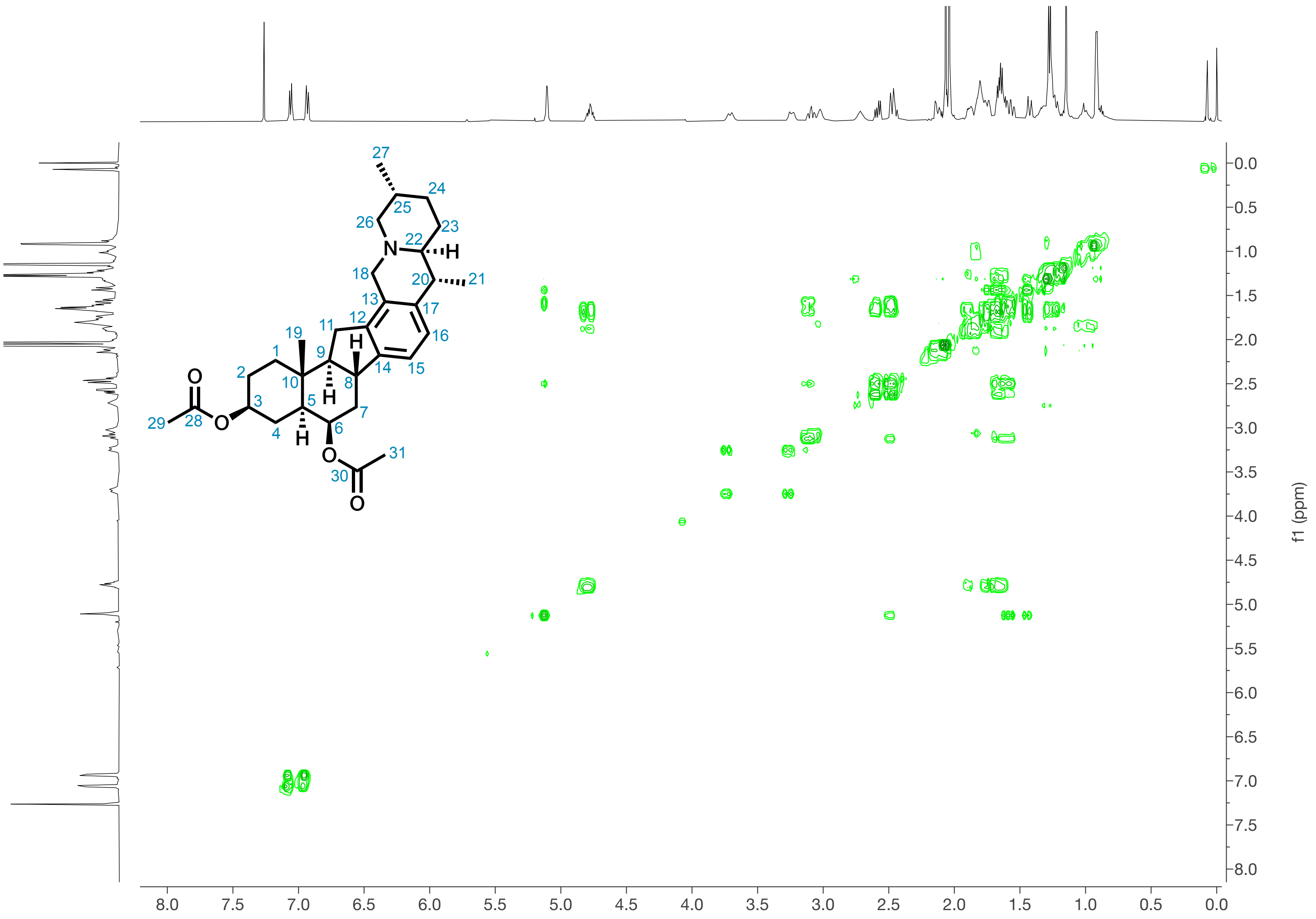

Figure S125. ^{1}H–^{1}H COSY (400 MHz, $CDCl_3$) of **heilonine diacetate**.

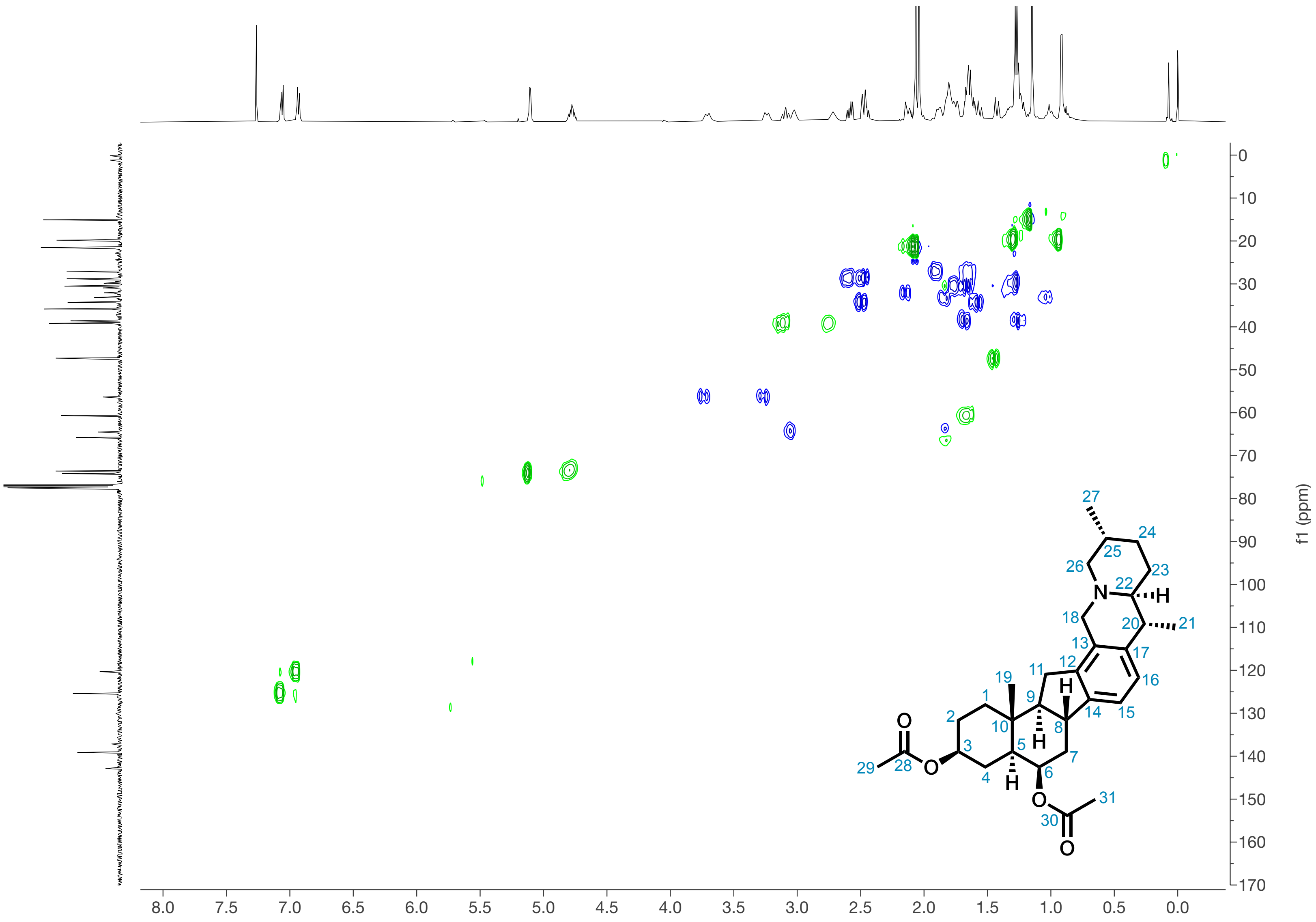

Figure S126. HSQC (400 MHz, $CDCl_3$) of **heilonine diacetate**.

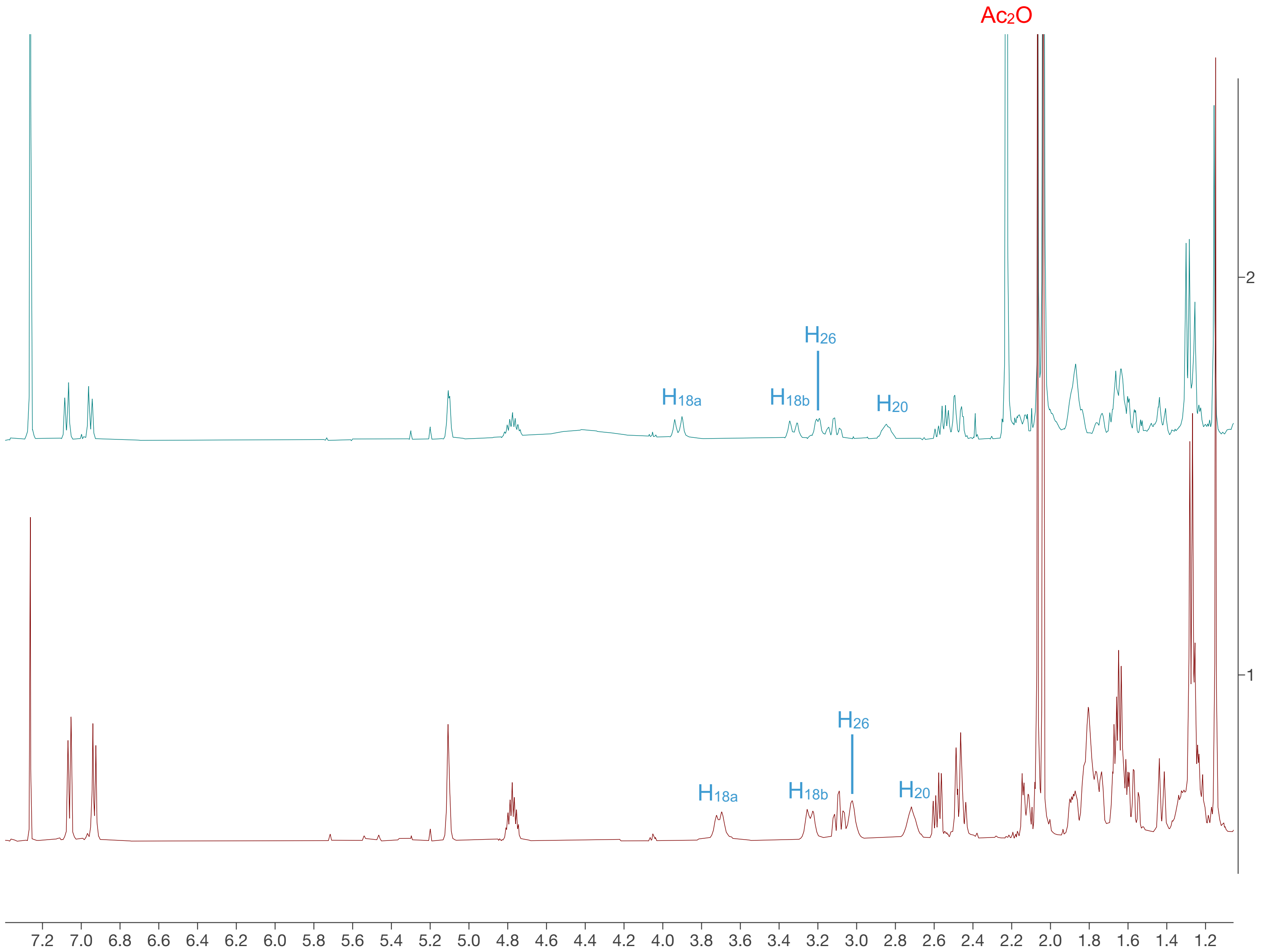

Figure S127. ^1H NMR (500 MHz, $CDCl_3$) of unpurified **heilonine diacetate** (top) and purified **heilonine diacetate**. The stacked spectra demonstrate the variability in chemical shifts due to residual acid.

Schemes of Previous Syntheses:[28]

Scheme S8. The Masamune synthesis of **jervine**.[29]

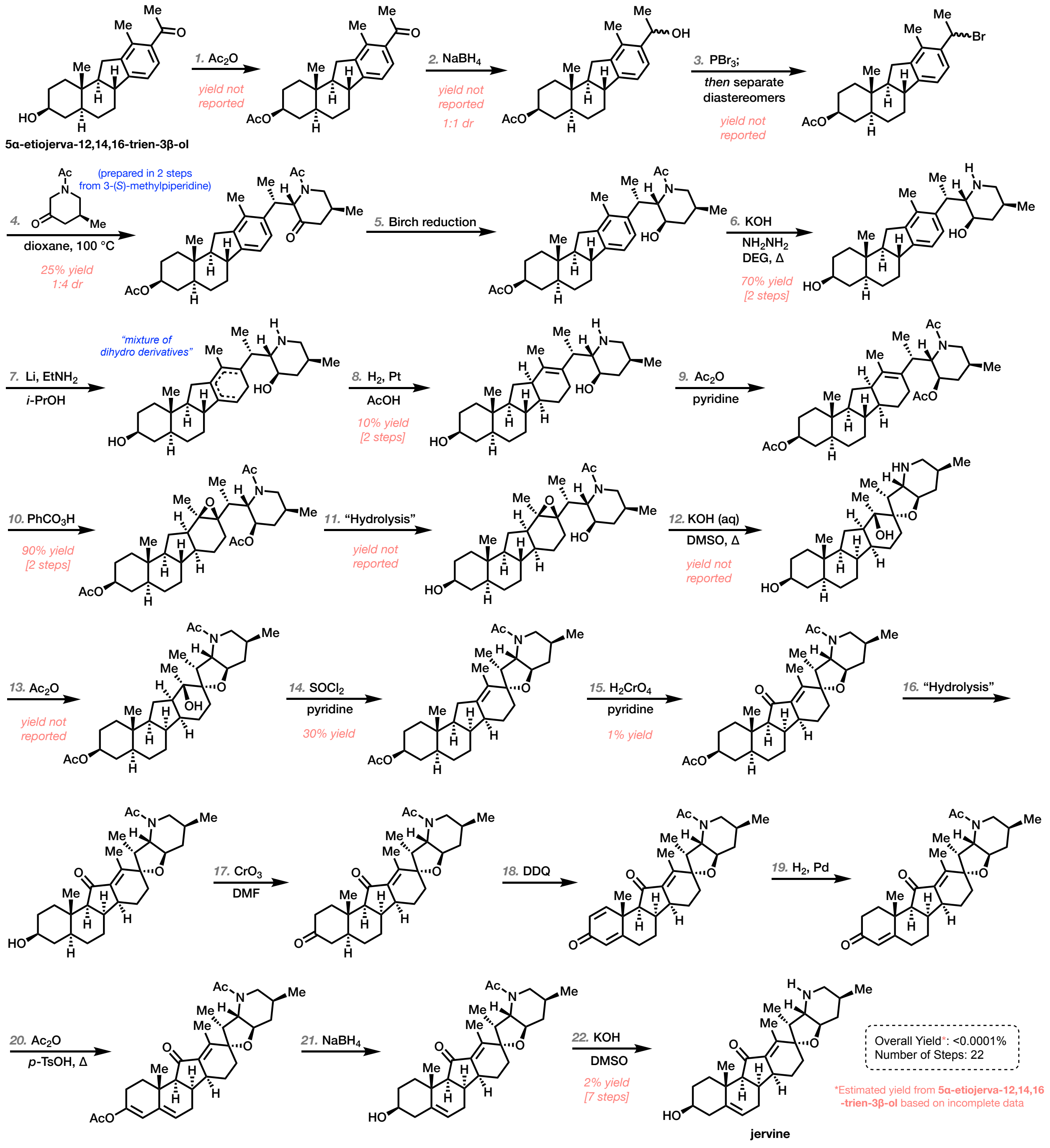

---

[28] The steps for the following syntheses were counted by us in a manner consistent with generally accepted practices. If procedures involved the sequential addition of reagents to a reaction, then it was considered one step. A work-up or solvent change, even if the intermediate was not further purified, was considered the end of one step and the beginning of a new one.

[29] Masamune, M.; Takasugi, M.; Murai, A.; Kobayashi, K. The Synthesis of Jervine and Related Alkaloids. *J. Am. Chem. Soc.* **1967**, *89*, 4521-4523.

Scheme S9. The Johnson synthesis of **veratramine**.[30]

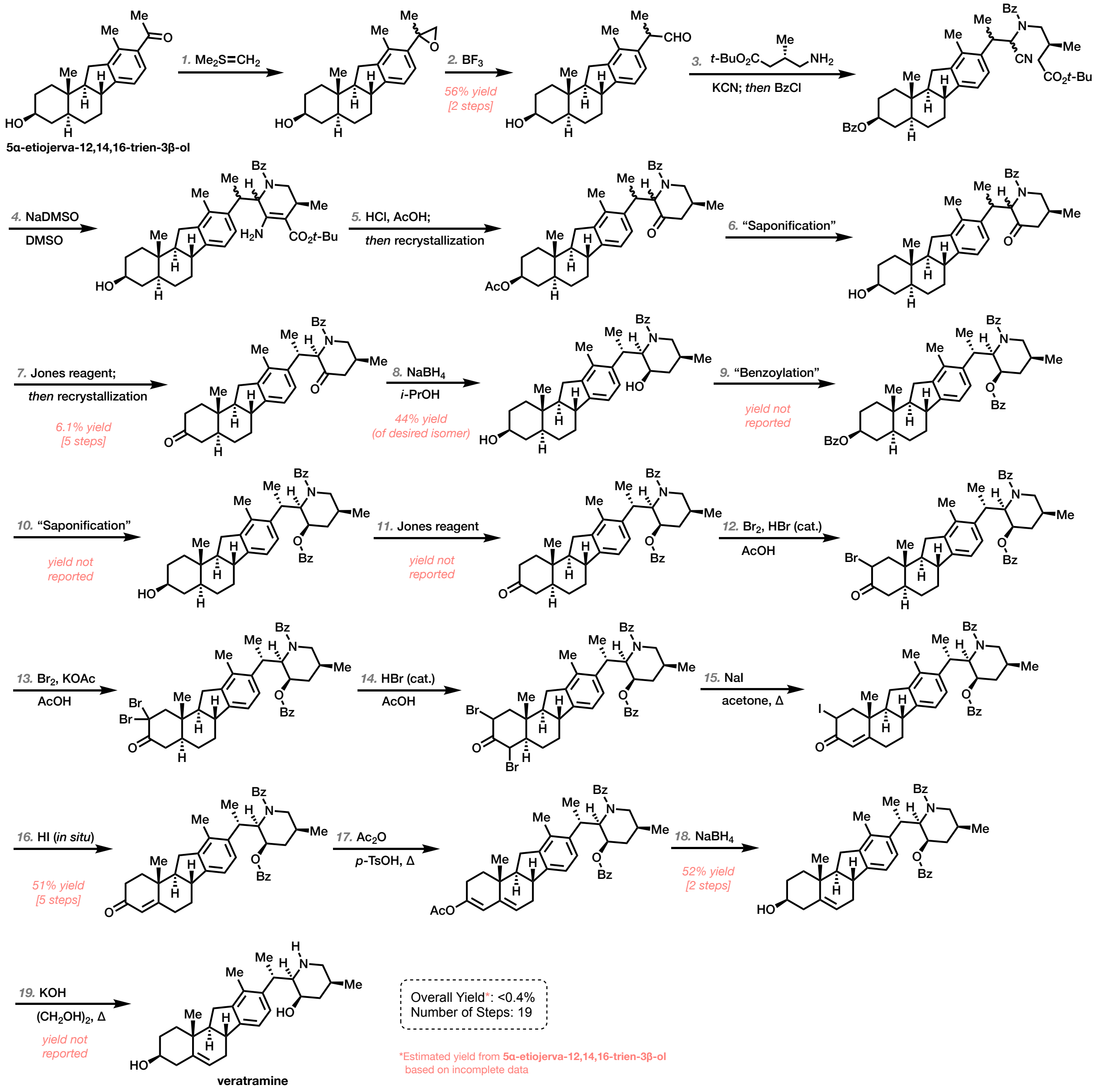

[30] Johnson, W. S.; deJongh, H. A. P.; Coverdale, C. E.; Scott, J. W.; Burckhardt, U. The Synthesis of Veratramine. *J. Am. Chem. Soc.* **1967**, *89*, 4523-4524.

Scheme S10. The Johnson synthesis of **5α-etiojerva-12,14,16-trien-3β-ol**.[31]

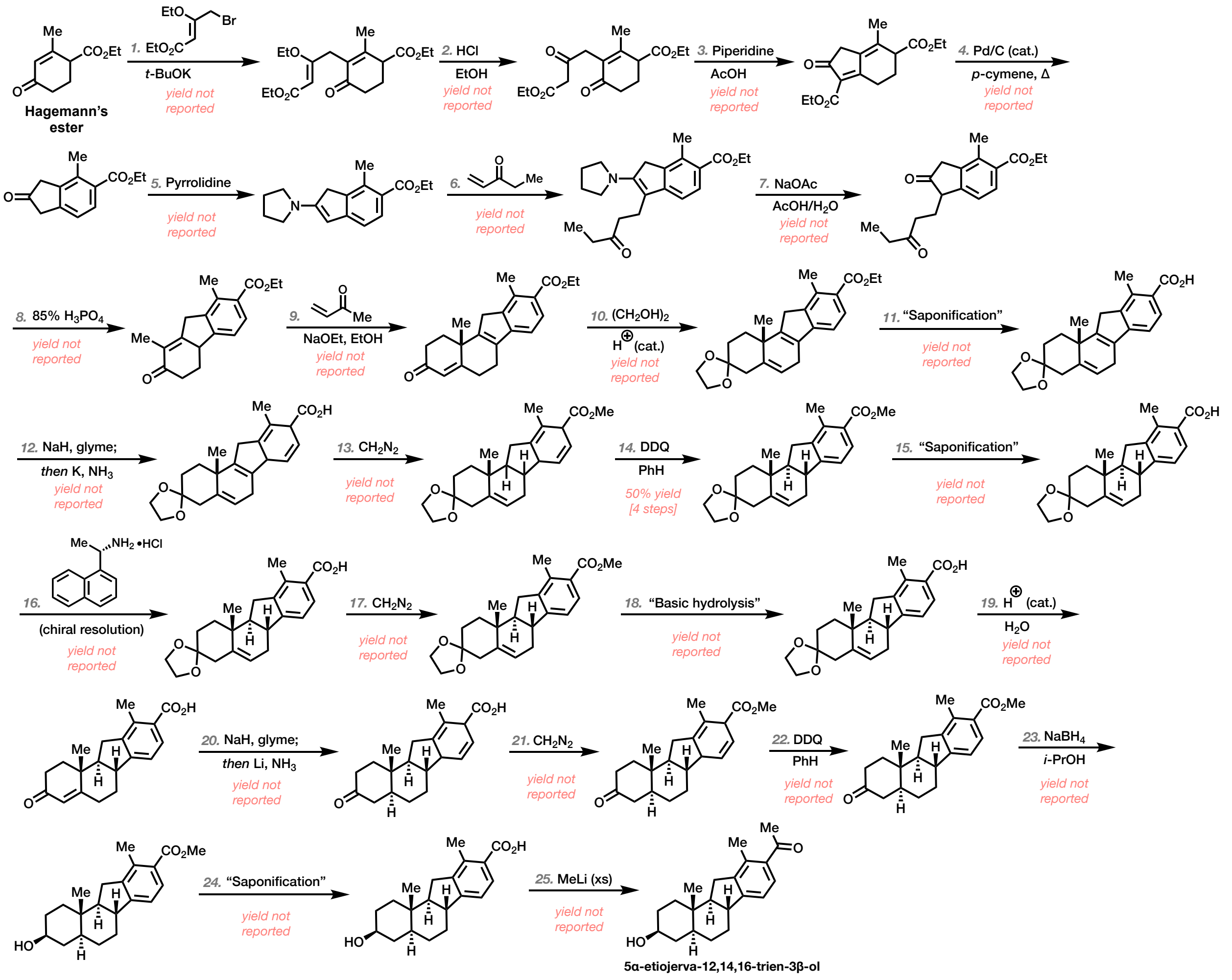

[31] Johnson, W. S.; Cox, J. M.; Graham, D. W.; Whitlock, Jr., H. W. The Total Synthesis of 17-acetyl-5α-etiojerva-12,14,16-trien-3β-ol. *J. Am. Chem. Soc.* **1967**, *89*, 4524-4526.

Scheme S11. The Kutney synthesis of **verticine**.[32]

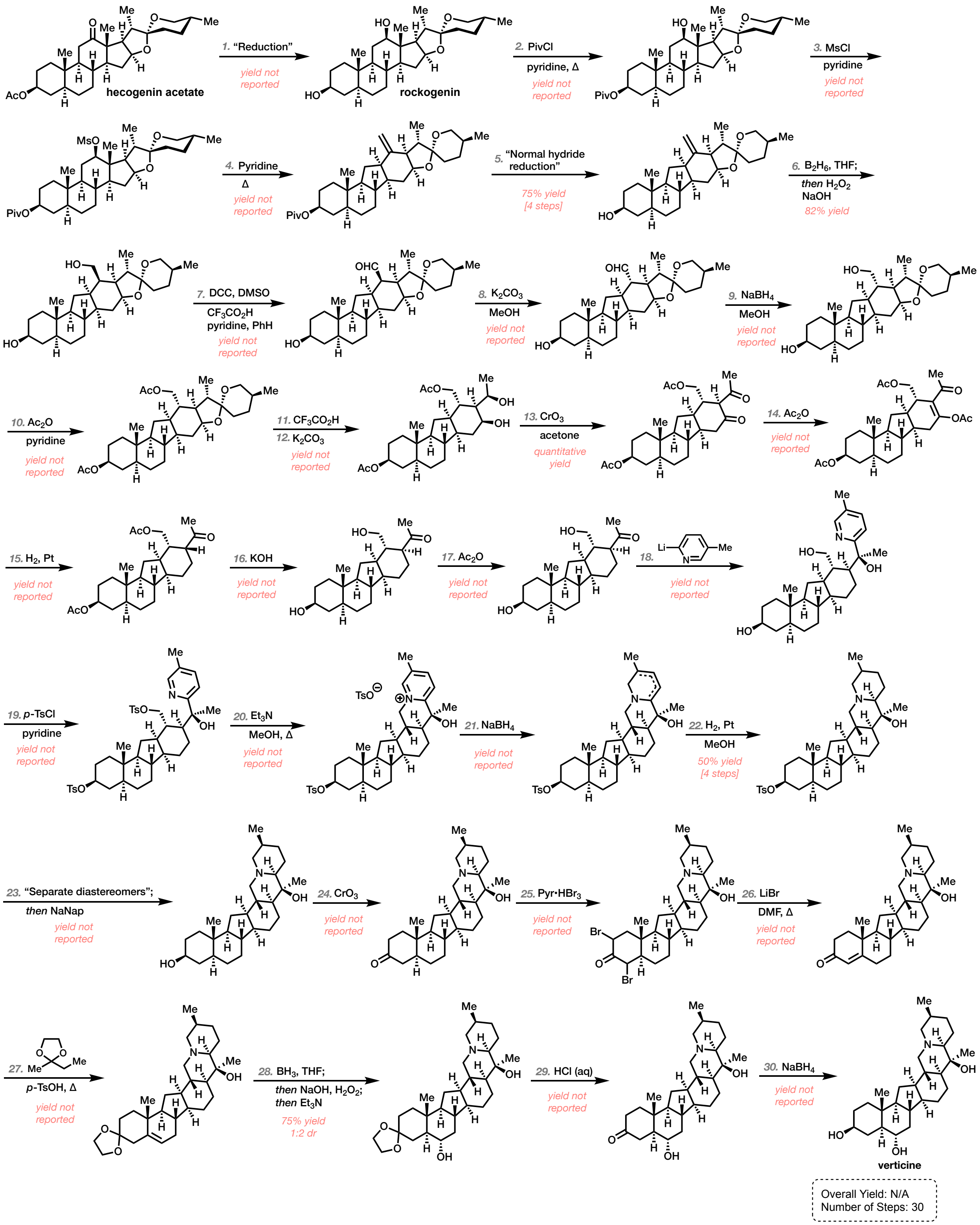

[32] Kutney, J. P.; Fortes, C. C.; Honda, T.; Murakami, Y.; Preston, A.; Ueda, Y. Synthetic Studies in the Veratrum Alkaloid Series. The Total Synthesis of Verticine. *J. Am. Chem. Soc.* **1977**, *99*, 964-966.

Scheme S12. The Giannis synthesis of **cyclopamine**.[33]

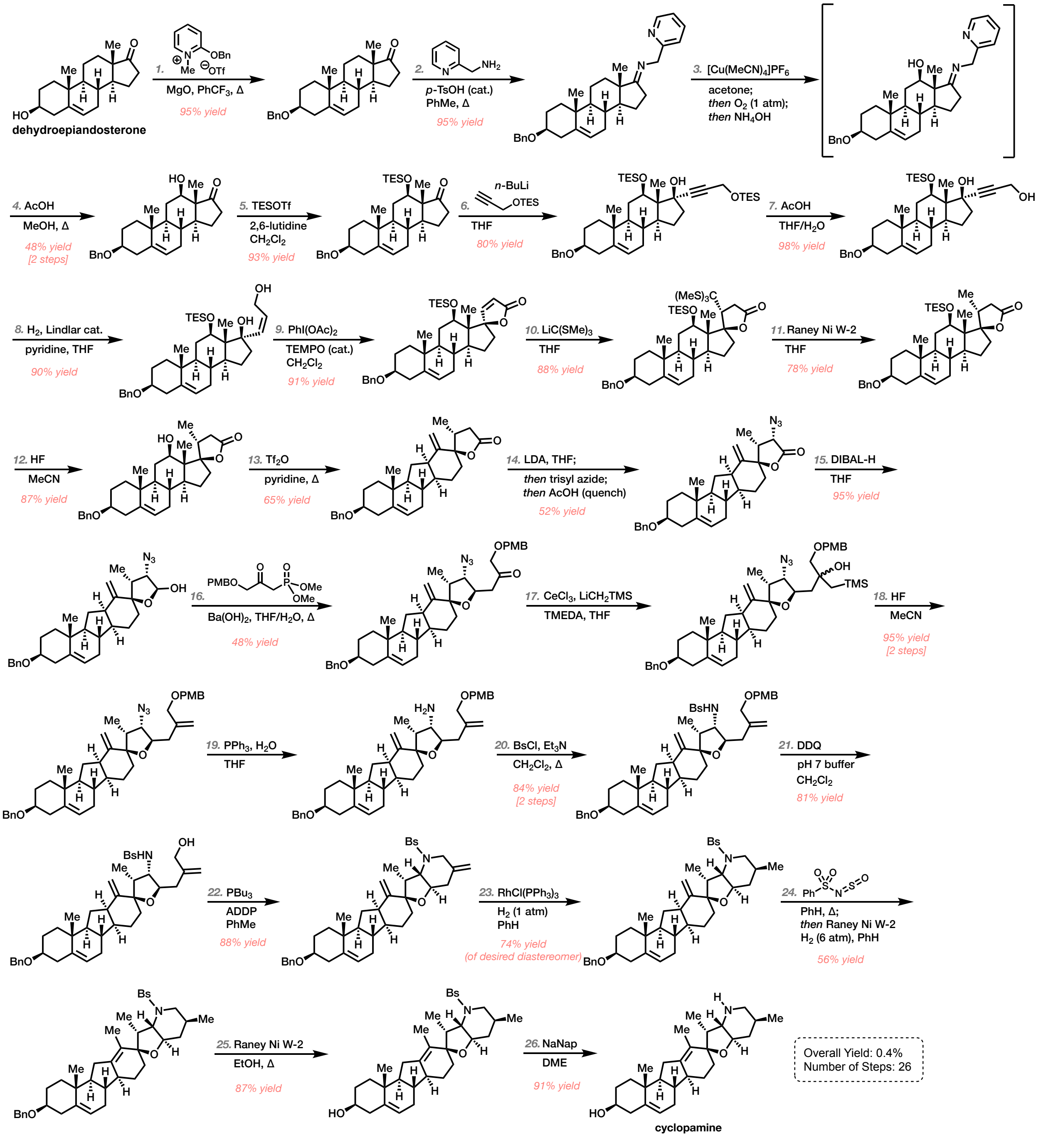

---

[33] Giannis, A.; Heretsch, P.; Sarli, V.; Stößel, A. Synthesis of Cyclopamine Using a Biomimetic and Diastereoselective Approach. *Angew. Chem. Int. Ed.* **2009**, *48*, 7911-7914.
